# Linking metabolic phenotypes to pathogenic traits among “*Candidatus* Liberibacter asiaticus” and its hosts

**DOI:** 10.1038/s41540-020-00142-w

**Published:** 2020-08-04

**Authors:** Cristal Zuñiga, Beth Peacock, Bo Liang, Greg McCollum, Sonia C. Irigoyen, Diego Tec-Campos, Clarisse Marotz, Nien-Chen Weng, Alejandro Zepeda, Georgios Vidalakis, Kranthi K. Mandadi, James Borneman, Karsten Zengler

**Affiliations:** 1 Department of Pediatrics, University of California, San Diego, 9500 Gilman Drive, La Jolla, CA 92093-0760 USA; 2 Department of Microbiology and Plant Pathology, University of California, Riverside, 900 University Avenue, Riverside, CA 92521 USA; 3 State Key Laboratory of Bioreactor Engineering and Institute of Applied Chemistry, East China University of Science and Technology, Shanghai, People’s Republic of China; 4 USDA, ARS, US Horticultural Research Laboratory, 2001 S. Rock Road, Fort Pierce, FL 34945 USA; 5 Texas A&M AgriLife Research and Extension Center, Texas A&M University System, Weslaco, TX USA; 6 Facultad de Ingeniería Química, Universidad Autónoma de Yucatán, Campus de Ciencias Exactas e Ingenierías, Mérida, 97203 Yucatán, México; 7 Department of Plant Pathology and Microbiology, Texas A&M University, College Station, TX USA; 8 Department of Bioengineering, University of California, San Diego, La Jolla, CA 92093-0412 USA; 9 Center for Microbiome Innovation, University of California, San Diego, 9500 Gilman Drive, La Jolla, CA 92093-0403 USA

**Keywords:** Biochemical networks, Plant sciences

## Abstract

*Candidatus* Liberibacter asiaticus (*C*Las) has been associated with Huanglongbing, a lethal vector-borne disease affecting citrus crops worldwide. While comparative genomics has provided preliminary insights into the metabolic capabilities of this uncultured microorganism, a comprehensive functional characterization is currently lacking. Here, we reconstructed and manually curated genome-scale metabolic models for the six *C*Las strains A4, FL17, gxpsy, Ishi-1, psy62, and YCPsy, in addition to a model of the closest related culturable microorganism, *L. crescens* BT-1. Predictions about nutrient requirements and changes in growth phenotypes of *C*Las were confirmed using in vitro hairy root-based assays, while the *L. crescens* BT-1 model was validated using cultivation assays. Host-dependent metabolic phenotypes were revealed using expression data obtained from *C*Las-infected citrus trees and from the *C*Las-harboring psyllid *Diaphorina citri* Kuwayama. These results identified conserved and unique metabolic traits, as well as strain-specific interactions between *C*Las and its hosts, laying the foundation for the development of model-driven Huanglongbing management strategies.

## Introduction

*Candidatus* Liberibacter asiaticus (*C*Las) has been associated with Huanglongbing (HLB), or citrus greening, a devastating vector-borne disease causing millions of dollars of agricultural damages every year^1^. *C*Las species infect the phloem of some plants in the family *Rutaceae* (e.g., citrus, *Murraya paniculata*) and *Solanaceae* (e.g., potato)^2^. HLB causes poor vegetative growth, fruit drop, diminished fruit quality, and tree decline^3–7^.

*C*Las infections have been documented across most citrus-producing areas in Asia, the Americas and Africa^5,8^, and are projected to spread further^9,10^. *C*Las is naturally spread in citrus through a psyllid host, *Diaphorina citri* Kuwayama^10^. The basic HLB management scheme is based on the use of HLB-free nursery stock, inoculum reduction by removal of HLB-affected trees and insecticide treatments for control of psyllid populations^11^. In addition, various combinations of citrus rootstocks and interstocks^11^, cocktails of antibiotics^12^ and small molecule bacterial inhibitors^13^, or brassinosteroids^14^, as well as thermotherapy and nanoemulsion technology^15^ have been deployed. However, none of these options has been proven to be very successful, economically viable, or environmentally sustainable, making HLB a major threat to the citrus industry worldwide.

Mathematical models have been critical in developing treatment options for infectious diseases^16^ and to understand complex metabolic capabilities^17^. These models could provide useful information for best practices to treat or prevent HLB. However, few detailed models of HLB currently exist^18^. To identify novel alternatives for combatting HLB, detailed knowledge about the metabolic dependencies and capabilities of the pathogen (*C*Las) is required. While *C*Las was identified as the likely infectious agent responsible for HLB in 1994 using molecular methods^19^, *C*Las has never been consistently cultivated axenically in vitro, limiting our ability to functionally characterize this pathogen. On the other hand, *Liberibacter crescens*, the closest culturable relative to *C*Las, was isolated and cultured in vitro from the phloem sap of defoliating mountain papaya in Puerto Rico^20,21^. Advances in metagenomic sequencing have enabled the assembly of genomes from several *C*Las strains obtained from HLB-infected citrus^22^. Genome sequences are the primary input used to reconstruct genome-scale metabolic models. These models have been successfully validated as a systems biology framework and deployed for a variety of uses. For example, models have been utilized for elucidating fundamental metabolic processes^23–26^, optimizing culture media and growth conditions^27,28^, and have been essential for metabolic engineering efforts^29^. These metabolic models are genome-scale knowledge databases, which contain manually curated annotation related to gene-protein-reaction associations for all possible metabolic reactions inside a cell. Reconstructed models have been used to understand and channel the metabolism of different pathogenic and non-pathogenic microorganisms^16,30^, as well as to contextualize metabolic states based on omics data^24^. Here, we reconstructed genome-scale models for seven *Liberibacter* strains and evaluated their physiology and metabolic response. Furthermore, we used in vivo expression data to determine strain-specific interactions of *C*Las while hosted by the psyllid *Diaphorina citri* Kuwayama or *Citrus* spp.

## Results

### Genome characteristics and model properties

**Fig. 1:**
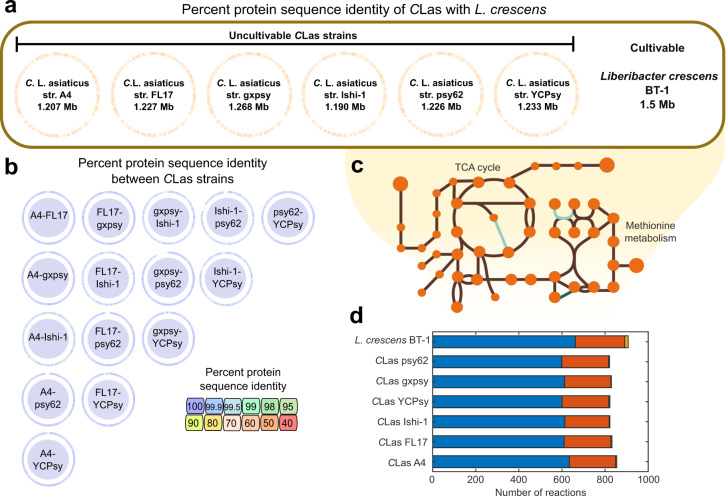
Properties of genomes and constraint-based metabolic models. **a** Percentage of protein sequence identity estimated using whole genome sequences of *Candidatus* Liberibacter asiaticus (*C*Las) strains and *L. crescens* BT-1. The identity between each *C*Las strain and BT-1 varied from 60–70%. **b** Protein-homology identity among *C*Las strains, which were over 99% identical. **c** Example of pathway completeness and gap filling for the TCA cycle and methionine metabolism in the *C*Las models. Circles represent metabolites, green represents gap filled reactions, and brown represents gene-associated reactions. **d** Reactions involved in central metabolism, including glycolysis/gluconeogenesis, the pentose phosphate pathway, and TCA cycle were categorized as pan reactions (highlighted in blue). The accessory reactions that include modeling reactions and other additional metabolic pathways (e.g., amino acid, carbohydrate and lipid metabolism) are shown in red. Unique reactions in each model are shown in orange.

Metabolic models were reconstructed based on complete genomes of the *C*Las strains gxpsy, Ishi-1, psy62, and almost complete genomes obtained by shotgun sequencing of strains A4, FL17, and YCPsy. Strains A4, FL17, Ishi-1, psy62, and YCPsy were obtained from citrus, while sequences for gxpsy were obtained from the psyllid. Additionally, we reconstructed a comparative metabolic model using the complete genome sequence of the microorganism *L. crescens* BT-1 (BT-1), the closest culturable microorganism to *C*Las (Supplementary Fig. 1a). All genome sequences were obtained from the PATRIC database^31^. In total, seven genome-scale metabolic models were reconstructed and the genomic and metabolic content of each strain was compared. Figure 1 details the main characteristics of the genomes and resulting models. The average number of annotated proteins was 1,185 across all *C*Las genomes and 1422 for BT-1. We calculated the percent protein sequence identity among the seven *Liberibacter* strains. The *C*Las genomes had around 75% identity to *L. crescens* BT-1 (Fig. 1a) and over 98% identity to each other (Fig. 1b).

A comprehensive organization of all available data and information on *Liberibacter* strains is crucial for overcoming the devastating impact of HLB. To this end, we created metabolic models that are also referred to as computational knowledge databases, which compile manually curated annotations for each *C*Las strain and *L. crescens* BT-1. After extensive curation of all models, a total of 1751 protein sequences (417 unique), previously annotated as hypothetical, were associated with a metabolic reaction or transport reaction in these models. A list of these reactions and their gene-protein-reaction associations is provided in Supplementary Table 1. Manual curation was followed by gap filling (Fig. 1c) and conversion of the reconstructions into mathematical models using the COBRA Toolbox^32^. The final model properties are shown in Table 1. The number of metabolites and reactions shared by all *C*Las models, defined as “Pan-capabilities”, were 447 and 601, respectively (Fig. 1d). Supplementary Fig. 2 shows a comparison of metabolites and reactions across the reconstructed models. The metabolic model of *L. crescens* BT-1 contained ~15% more metabolic reactions and metabolites than the *C*Las strains, hinting at a broader metabolic capability of *L. crescens*. Around 30% of reactions present only in BT-1 were associated with amino acid metabolism (e.g., methionine, lysine, glycine, serine and threonine metabolism). Another ~30% of those reactions were associated with the cell envelope (e.g., transporters and fatty acids). The rest of the reactions were divided among carbohydrate, glycan, and nucleotide metabolism (see Supplementary Table 2). We found that auxotrophies are *C*Las strain-specific, especially auxotrophies for l-proline, l-serine, and l-arginine (Supplementary Fig. 2). All models predicted auxotrophies for vitamins (e.g., riboflavin, biotin, thiamin, choline) and steroids (e.g., pantothenate, taurine, l-carnitine, quinate) (Supplementary Fig. 2).

**Table 1:** Properties of the genome-scale metabolic models.

Microorganism	Genome ID	Model ID	Genes	Reactions	Metabolites
*Candidatus* Liberibacter asiaticus					
A4	34021.4	A4	283	840	837
FL17	34021.11	FL17	272	818	807
gxpsy	1174529.3	gxpsy	276	815	807
Ishi-1	931202.3	Ishi-1	253	818	802
psy62	537021.9	psy62	285	818	807
YCPsy	34021.12	YCPsy	279	814	805
*Liberibacter crescens*					
BT-1	1215343.1	BT-1	372	892	887

### Validation of the *Liberibacter crescens* BT-1 model and analysis of *Candidatus* Liberibacter asiaticus models

Cruz-Munoz et al.^33^ recently reported citrate as the preferred carbon and energy source for *L. crescens* BT-1. The authors reported the growth of *L. crescens* BT-1 on the complex media BM-7 by measuring optical density. They also tested various media compositions (M13, M14, and M15), and developed an optimized defined medium (M15, containing citrate), which improved the growth rate^33^. Using OD measurements we determined the growth rates of *L. crescens* BT-1 while growing in BM-7, reaching a growth rate of 0.011 ± 0.007 1/h, which results in a 63 h generation time. Defined media compositions, such as M13, M14, and M15 can also support growth of this strain, resulting in growth rates of 0.009 ± 0.0006 1/h, 0.0081 ± 0.0004 1/h, and 0.012 ± 0.003 1/h, respectively.

**Fig. 2:**
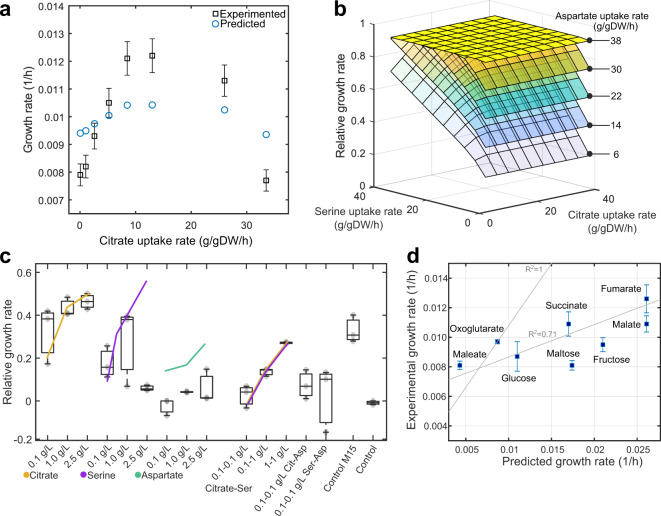
Validation of the metabolic model of *Liberibacter crescens* BT-1. **a** Simulated growth rate results using the culture medium M15 while varying the citrate concentration (blue circles). Squares with error bars (standard deviation) represent the average growth rate calculated using OD measurements. Citrate constraints used for simulation as well as experimental and predicted growth rates are given in Supplementary Table 3. Increase in the growth rate at citrate uptake rates higher than 10 g/gDW/h was associated with an increase of alanine uptake rate. **b** Relative growth rate predictions across varying citrate, serine, and aspartate uptake rates. Growth rate in M15 was used as control **c** Experimentally determined growth phenotypes. Box plots show *L. crescens* BT-1 average growth under 14 treatments using several concentrations of citrate, serine, and aspartate and their interaction, the central mark indicates the media, and the bottom and top edges of the box indicate the 25th and 75th percentiles, respectively. Experiments were carried out using three independent replicates. Growth rates were calculated using 25 consecutive time points (sampled every 12 h) measured over the course of 12 days. The culture medium M15 and the base media (without nutrients) were used as controls. **d** Comparison among predicted and experimental growth rates of BT-1 while using the culture media M14 (Supplementary Table 3) in combination with glucose, fructose, fumarate, malate, maleate, maltose, oxoglutarate, and succinate as additional carbon sources. Predicted and observed growth phenotypes were correlated up to 71% (Pearson correlation, *p*-value < 0.0041, *n* = 3). A theoretical *R*^2^ = 1 is also shown in the figure for comparison purposes. Squares represent the average growth and its standard deviation.

These growth rate phenotypes observed experimentally were reproduced *in silico* by the BT-1 model, obtaining growth rate predictions of 0.011, 0.009, 0.011, and 0.015 1/h for the culture media BG-7, M13, M14, and M15, respectively. Complete data and calculations about constraints are shown in Supplementary Table 3. Growth rate predictions using M15 media with varying citrate concentrations are shown in Fig. 2a, along with the corroborating experimental results^33^. To determine the impact of amino acids and intermediaries of the TCA cycle on growth rate, we interrogated the model for metabolites most affecting the growth rate of *L. crescens*. We found that citrate, aspartate, and serine have an interwoven growth effect. Simulations performed while varying serine, aspartate, and citrate uptake rates are shown in Fig. 2b, highlighting a proportional increase among predicted growth rates and constrained uptake rates. Predicted trends were confirmed experimentally for citrate and serine individually and in combination (Fig. 2c); however, increased aspartate additions reduced the growth of BT-1 experimentally. These aspartate phenotypes can be attributed to high-metabolic regulation since aspartate is link to nine operons in BT-1^34^.

*C*Las models were evaluated using different culture media compositions to unravel the most important metabolites contributing to *C*Las growth. The culture media compositions tested for *L. crescens* BT-1 were used as constraints to simulate *C*Las growth as shown in Supplementary Table 3. Simulations using single carbon sources (uptake rate of 15 mmol/gDWh) demonstrated that none of the *C*Las strains were able to grow, suggesting that co-metabolism with the host could play an important role for these bacteria. Modeling results showed that arginine, glucose, glutamate, glutamine, proline, ornithine, citrate, and alpha-ketoglutarate could each support the growth of *L. crescens* BT-1 individually. However, we found that *C*Las strains were highly dependent on co-metabolism to stimulate growth, necessitating the combination of two or more carbon sources at the same time (e.g., glucose and glycine, or aspartate, or serine, or succinate). The growth rate improvement due to the addition of single metabolites in the culture media containing glucose was evaluated for each metabolite (Supplementary Table 3). Co-metabolism simulations were performed by assessing the growth rate predictions while varying the uptake rates of serine, aspartate, and citrate in a continuous gradient from 0 to 45 g/gDW/h. Experimental results from *L. crescens* suggest that citrate and serine are the main drivers of its growth, followed by aspartate (Fig. 2c).

The role of each metabolite in the metabolic model was evaluated across all models (Supplementary Fig. 3). We found that all *C*Las strains have similar simulated growth rates across all media compositions (i.e., BM-7, M13, M14, and M15). However, when the effect of each metabolite was analyzed independently, we found that media composition affected *C*Las growth rates differentially (Supplementary Fig. 3), suggesting strain-specific phenotypes. Also, the growth rates predicted for *L. crescens* BT-1 differed from those predicted for the six *C*Las strains (Supplementary Fig. 3).

**Fig. 3:**
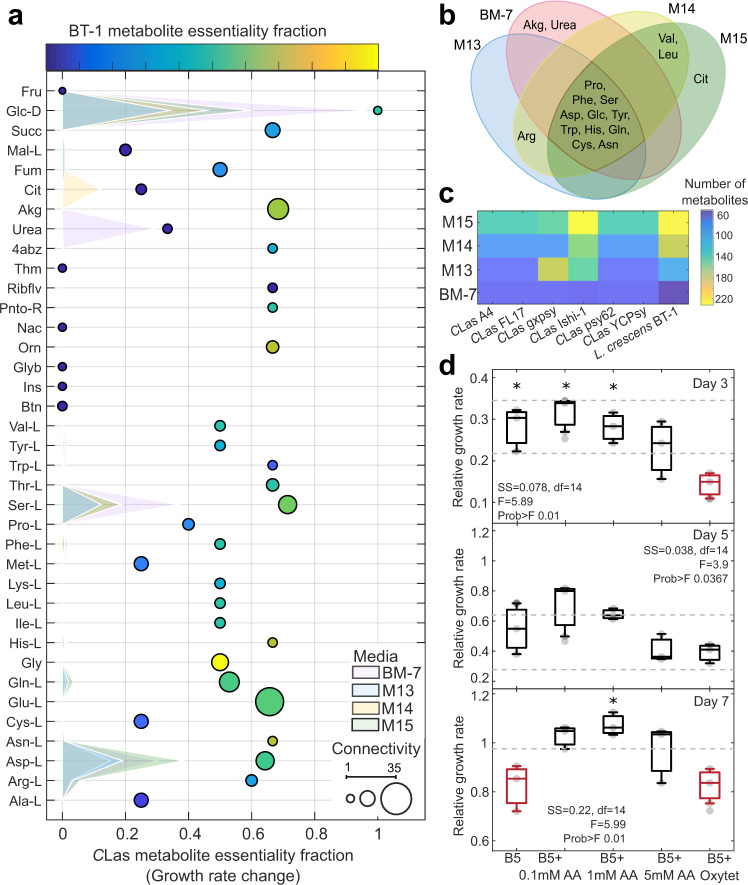
Model evaluation by culture media. **a** Metabolic connectivity and essentiality. The essentiality fraction (change in growth rate) was determined by identifying the percentage of reactions in which each metabolite in the culture media participates. Metabolite essentiality in *Candidatus* Liberibacter asiaticus strains and *Liberibacter crescens* BT-1 are shown by bubble location and bubble color (non-essential in dark blue to essential in yellow), respectively. Bubble size indicates connectivity. Shaded surfaces represent the metabolite contribution to the growth rate by culture media. **b** Metabolites predicted to improve *C*Las growth rates are compared across media compositions in the Venn diagram. **c** Biosynthetic capacity under maximal *Liberibacter* biomass growth rate by culture media. The color scale represents the number of metabolites predicted to be synthesized. **d** In vitro measurements of *C*Las growth rates over time in hairy root-assays. Panels show *C*Las titers for day 3, 5, and 7. Experimental measurements were normalized to one for day 0. Each panel shows the one-way ANOVA for the five assayed treatments (B5, culture medium suitable for the hairy root system; B5 + 0.1 mM AA, the hairy root system using B5 medium in addition with 0.1 mM of a cocktail of glycine, serine, proline, aspartate, glutamate, and glutamine; subsequently the amino acid cocktail was added in concentrations 1 mM and 5 mM for the treatments, B5 + 1 mM AA; B5 and B5 + 5 mM AA; the treatment B5 + Oxytet contained 500 mg/L of the antibiotic oxytetracycline). Measurements were obtained using at least four and maximum five replicates of independent samples, each sample was measured four times (0, 3, 5, and 7 days). The ANOVA function tests the hypothesis that the samples (4–5 total) are drawn from populations with the same mean against the alternative hypothesis that the population means are not all the same. Standard ANOVA stats are given in each panel. Box plots, the central mark indicates the media and the bottom and top edges of the box indicate the 25th and 75th percentiles, respectively, marked with asterisk (*) are significantly different from the red boxplot(s) of the same panel.

Part of the well established modeling protocols is the determination of the average metabolite connectivity^32,35^ and respective contributions to growth. We evaluated the variation of connectivity using our seven reconstructed models, determining potential metabolites with strong role in the metabolism. Metabolite connectivity highlighted differences among the models for the six *C*Las strains (Fig. 3a). For example, proline was connected (based on bubble size) to six reactions in the gxspy model, but to only five reactions in the other *C*Las models, while the opposite was observed for methionine and malate (Supplementary Table 4). The connectivity network enabled us to estimate the contribution of each metabolite to growth rate, and thereby calculate the essentiality fraction (relative change in growth rate). Figure 3a summarizes modeling predictions of the individual carbon sources for the metabolic models in different culture media compositions, highlighting the differences in predicted growth rate by the presence of specific metabolites (shaded areas in Fig. 3a). Highly connected metabolites, such as glutamine, glutamate, serine, and alpha-ketoglutarate, had high essentiality fractions, around 0.5-0.7 in both the *C*Las and *L. crescens* BT-1 models. However, the only metabolite with the same essentiality fraction as glucose was glycine for *L. crescens* BT-1. The essentiality fraction of metabolites, such as succinate, fumarate, citrate, urea, tryptophan, arginine, and riboflavin, were dramatically lower for *L. crescens* compared to the *C*Las strains. The metabolites predicted to increase growth rate for each of the four media compositions are shown in a Venn diagram (Fig. 3b). Glucose and nine amino acids improved the simulated *C*Las growth rates in all culture media, whereas alpha-ketoglutarate (akg) and urea were predicted to improve growth rate only for the BM-7 media. Overall, modeling predictions showed that serine, malate, fumarate, and aspartate will improve the growth rate of *C*Las strains in culture medium M13, M14, and, M15. On the other hand, predicted metabolites limiting the growth rate are nicotinate, pantothenate, riboflavin, and aminobenzoate when BM-7 culture media was used.

While these results hint at specific metabolites that could improve *C*Las growth rates, they also suggest that phenotypic outcomes depend on the media composition to which each metabolic model is being subjected. The heatmap in Fig. 3c shows the predicted number of metabolites produced when biomass production is optimized. For microorganisms such as *C*Las gxpsy and Ishi-1 the media composition can strongly affect the ability of a strain to synthesize metabolites, varying from 60 to over 220 metabolites. The M15 media composition resulted in maximal metabolite production for every model, except for *C*Las gxpsy. The culture medium BM-7 resulted in a similar number of produced metabolites for all seven models. We also evaluated the growth phenotype under eight different carbon sources assayed experimentally, predicting accurately the growth rate for oxoglutarate, glucose, and malate and an overall growth increase for fructose, fumarate, malate, maltose, and succinate (Fig. 2d). The BT-1 model could explain up to 71% (Pearson correlation, *p*-value < 0.0041) of the changes in the observed growth rate for the different carbon sources^33^.

To test this experimentally with *C*Las, a cocktail of predicted metabolites was added to in vitro citrus hairy root cultures infected with *C*Las. Culture media B5, which is used to propagate the hairy root cultures, was supplemented with three concentrations (0.1, 1, or 5 mM) of an amino acid cocktail containing glycine, serine, proline, aspartate, glutamine, and glutamate. Measurements of *C*Las in the hairy root cultures obtained over the course of seven days showed that the treatment of 1 mM significantly improved *C*Las growth rate compared to 0.1 and 5 mM treatments, as well as controls (*F* = 5.99, Prob > *F* 0.01, df = 14) (Fig. 3d).

### Host-dependent constraints reveal activation of *Candidatus* Liberibacter asiaticus pathways associated with pathogenic phenotypes

After validating the *L. crescens* BT-1 genome-scale metabolic model, we evaluated the response of *C*Las models using host-specific RNA-sequencing data. Expression data was collected from phloem-enriched samples (referred to as phloem in the rest of the paper) from three Citrus cultivars (Valencia orange, Washington navel orange, and Tango mandarin) and from the alimentary canals of *Diaphorina citri* (psyllid). Using RNA-sequencing reads we generated strain-specific counts that were used to constrain the boundaries of the reactions in each model. Supplementary Table 5 shows statistics about data preprocessing of RNA-seq data such as number of raw reads and total counts generated after trimming reads aligned to the citrus or psyllid genomes. Supplementary Figs 4 and 5 show the expression profiles for all *C*Las strains and the differentially expressed genes using a cut off of *p*-value < 0.05 (*t*-test).

**Fig. 4:**
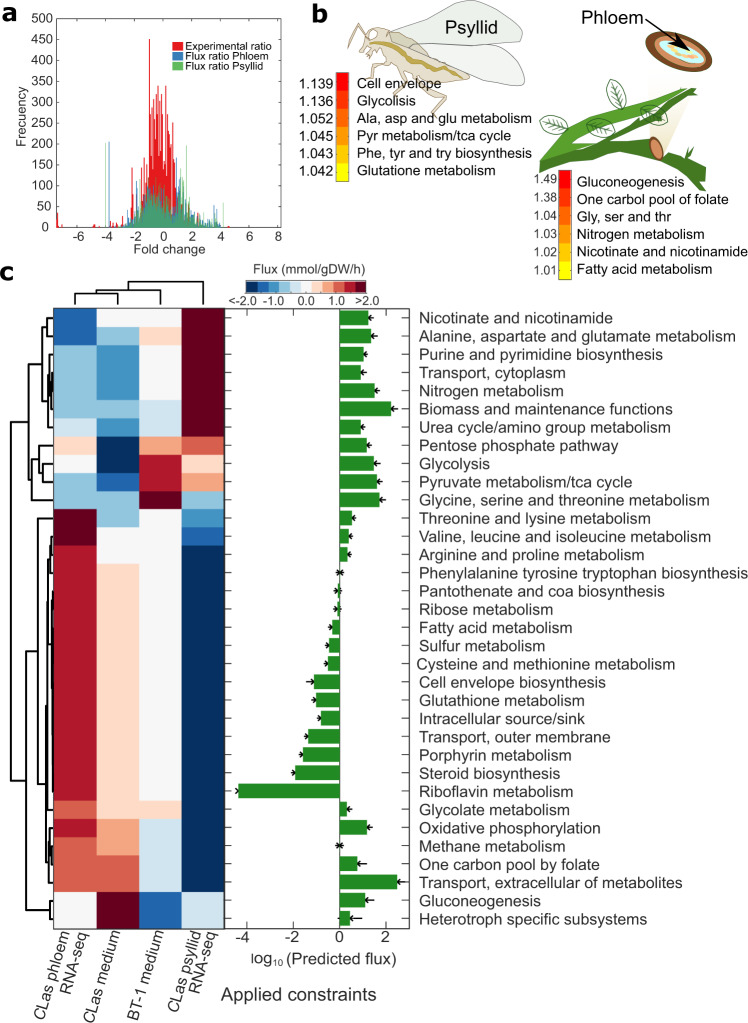
Model-driven analysis of *Candidatus* Liberibacter asiaticus (*C*Las) RNA-sequencing data. **a** The histogram shows the distribution of the average fold change of *C*Las expression for samples obtained from the psyllid alimentary canals and citrus phloem. In red the fold change between phloem and psyllid samples is shown using the entire RNA-sequencing dataset. Plotted in blue and green is the average ratio of predicted flux distribution from the *C*Las model (A4, FL17, gxpsy, Ishi-1, psy62, and YCPsy) using M15 culture medium and RNA-sequencing data from psyllid and phloem samples, respectively. **b** Heatmap highlighting *C*Las subsystems with highest flux activity in psyllid and phloem samples. Numbers represent the fold change increase of predicted flux. **c** Analysis of predicted flux distributions from constraining the *C*Las models with RNA-sequencing data (phloem and psyllid), or culture medium M15 (Medium) compared to the predicted flux distribution for *L. crescens* BT-1 using M15 medium. Barplot shows the total flux carried by pathway by all the simulated flux distributions for all *C*Las and *L. crescens* strains. Correlation matrix shows that flux distribution clusters by constraint conditions independently of *C*Las strain (Supplementary Fig. 6), enabling to averaging of the flux distribution by strain.

As expected, gene expression fold change between phloem and psyllid samples was normally distributed across genes. The maximum fold change observed was 28. Figure 4a shows the distribution of the flux ratio used to constrain the *C*Las models from each host, as well as the fold change between samples from the two hosts obtained from RNA-sequencing data. Data from the individual phloem and psyllid samples were combined into two separate datasets that were used to constrain the models, giving insight into specific metabolic traits operated by *C*Las under each host. Constrained models are provided in at https://github.com/cristalzucsd/Liberibacter.

Host-specific simulations performed using the *C*Las models (A4, FL17, gxpsy, Ishi-1, psy62, and YCPsy) resulted in a growth rate decrease of up to 68% and 74% for phloem and psyllid, respectively, in comparison to media-constrained conditions in which the predicted and experimental growth rate was around 0.013 ± 0.0009 1/h. Phloem-constraint flux distributions revealed increased amino acid metabolism, fatty acid metabolism, gluconeogenesis, nitrogen metabolism, one-carbon pool of folate, nicotinamide and nicotinate metabolism (Fig. 4b). Network evaluation showed that these pathways are interconnected by metabolites, such as formate, glycine, and 5,10-methylenetetrahydrofolate, linking these subsystems with possible pathogenic traits.

On the other hand, psyllid-constraint flux distributions revealed increased amino acid metabolism, cell envelope, glycolysis, pyruvate metabolism, and TCA cycle (Fig. 4b). The reactions associated with the cell envelope catalyze the synthesis of membrane lipids (enoyl reductase, EC 1.3.1.9) and oligosaccharides (DTDP-4-dehydrorhamnose 3,5-epimerase, EC 5.1.3.13, DTDPglucose 4,6-dehydratase, EC 4.2.1.46, and Glucose-1-phosphate thymidylyltransferase, EC 2.7.7.24). These oligosaccharide enzymes also participate in the metabolism of nucleotide sugars, streptomycin biosynthesis, and polyketide sugar biosynthesis. Polyketide biosynthesis is closely related to the additional availability of sugars and organic acids (e.g., pyruvate, succinate, malate), which are products of *C*Las metabolism.

Figure 4c shows the predicted flux distributions when BT-1 and *C*Las models are differentially constrained (media and expression data of phloem or psyllid). The overall flux distributions across all constraints showed that subsystems, including glycolysis, transporters, TCA cycle, pyruvate metabolism, and nitrogen metabolism were highly activated, which was the opposite of riboflavin metabolism, steroid biosynthesis, and cysteine and methionine metabolism. Furthermore, the pentose phosphate pathway was predicted to be active only under host-constraints while glycine, serine, and threonine metabolism were reduced in flux.

Broadly, *C*Las models constrained with phloem expression data clustered with models constrained with culture media M15 and *C*Las models constrained with psyllid expression data clustered with the flux distribution of BT-1 (Fig. 4c). These findings provide insight into potential metabolic stages that could facilitate *C*Las cultivation in vitro when obtained from the psyllid host.

### Predicting genetic targets for HLB management

**Fig. 5:**
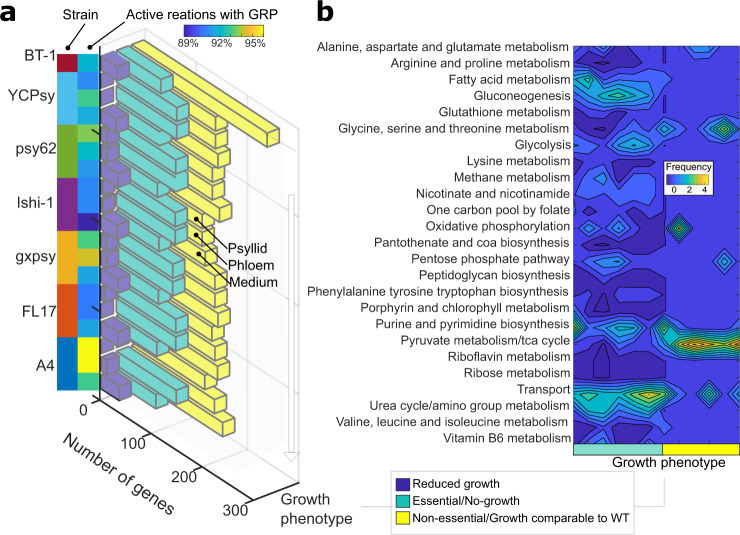
Predicted gene essentiality by strain and host. **a** Breakdown of phenotypes of all the genes in the metabolic models. Gene essentiality predictions are shown for all six *C*Las strains and BT-1. Phenotypes are binned into three categories based on predicted growth rate after individual gene knock-out; no growth (essential genes-green), reduced growth rate (blue), and comparable growth rate to wild-type conditions (non-essential-yellow). The gene essentiality results are plotted for each strain under the three different constraint datasets (psyllid RNA-sequencing data, psyllid; phloem RNA-sequencing data, phloem; and culture medium M15, medium). Note that *L. crescens* BT-1 only contains predictions for the culture medium, because it is not a pathogen found in either the psyllid or the citrus phloem. Percentage of active reactions with gene-protein-reaction association during the simulation of non-essential genes is shown in the blue-to-yellow heatmap. **b** The contour plot shows the number of *C*Las genes that have unique phenotypes across the three constraint datasets, meaning that some genes that are non-essential in culture medium became essential when constrained by host conditions. We found that genes reducing the growth rate were consistent among growth conditions, while categorizing genes as essential or non-essential depended on the host. The number of genes that changed between essential and non-essential categorization under different host conditions is binned by subsystem, where yellow means maximum six genes changed in the subsystem, and dark blue is no genes changed in the subsystem. The complete dataset is provided in Supplementary Tables 6 and 7.

Identification of potential *C*Las essential genes can lead to the development of HLB management strategies by designing molecules that specifically block or inhibit these gene products. We simulated single-gene knockouts, changing the reaction bounds of all reaction(s) associated to each gene, while maintaining the host-dependent constraints for the rest of the network. Figure 5 shows the phenotypic changes when media, psyllid RNA-seq data and phloem RNA-seq data constraints were applied to each of the six *C*Las models and the BT-1 model.

We found that host-dependent constraints not only affect growth phenotypes in different environments, but also affect gene essentiality by strain and activated number of reactions. The *C*Las strain YCPsy was the most sensitive, with 94 essential genes in comparison with the strains gxpsy, Ishi-1, psy62, FL17, and A4, which had 93, 91, 90, 89, and 87 essential genes, respectively (see Supplementary Fig. 7). Overall, the number of essential genes increased around 27 ± 2% because of the host-constraints imposed on the models compared to media constraints (Fig. 5a, green bars). Most of the essential genes common across the six *C*Las strains are involved in purine and pyrimidine metabolism, panthothenate and CoA biosynthesis, fatty acid metabolism and gluconeogenesis (Supplementary Fig. 7). However, when we evaluated the unique differences by subsystem across the six *C*Las strains, we found that genes involved in fatty acid metabolism, gluconeogenesis, glycine, isoleucine, leucine, serine, threonine, and valine metabolism, the TCA cycle, transport reactions, and urea cycle are the most sensitive and provide potential targets for the development of HLB management strategies (Fig. 5b). A full list of genes by subsystem is given in Supplementary Tables 6–8.

Previously, a study of random transposon mutagenesis of *L. crescens* suggested that 105 metabolic genes were essential^36^. Genome-scale models contain 71 of those genes and predict that 18 of those genes are essential, 12 reduce the growth rate, and 41 are not essential (Supplementary Table 8). We then compared the 18 essential genes that were identified both experimentally and *in silico* with genes overexpressed during *C*Las infection to pinpoint potential targets for HLB mitigation. During *C*Las infection of *C. sinensis* the enzymes DTMP kinase (EC 2.7.4.9), inorganic diphosphatase (3.6.1.1), coproporphyrinogen oxidase (EC 1.3.3.3), and protoporphyrinogen oxidase (EC 1.3.3.4) were overexpressed (*t*-test, *p*-value < 0.05, fold change > 3) and identified as essential. Additionally, we compared all predicted essential genes (91) with genes overexpressed (*t*-test, *p*-value < 0.05 and fold change > 10, *n* = 3) in the citrus cultivars Valencia and Washington navel orange (*C. sinensis* L. Osb.) and Tango mandarin (*C. reticulata* Blanco) and the *C*Las enzymes phosphoglycerate mutase (EC 5.4.2.12), dihydroorotic acid (menaquinone-8) (EC 1.3.5.2), ribonucleoside-diphosphate reductase (UDP) (glutaredoxin) (EC 1.17.4.1), and glutaredoxin reductase (EC 1.20.4.1) were selected. Together, these results suggest eight distinct enzymes, whose inhibition could reduce *C*Las pathogenicity. The full dataset of metabolic reactions that are potential genetic targets in the *C*Las strains studied here are shown in Supplementary Table 9 and Supplementary Fig. 6.

## Discussion

Constraint-based modeling allowed us to elucidate metabolic changes in *Candidatus* Liberibacter asiaticus (*C*Las) during growth in the psyllid host *Diaphorina citri* Kuwayama and infection of the plant host *Citrus* spp. This work represents a systems biology modeling approach to understand the metabolic role of *C*Las, the putative vector-borne causal pathogen of HLB, in citrus infection. We generated high-quality, manually curated genome-scale metabolic models of the six *C*Las strains A4, FL17, gxpsy, Ishi-1, psy62, and YCPsy (Fig. 1). All models combine genomic and biochemical information with available literature resources to date. Manually curated models are characterized by an unprecedented quality in annotation^16,17,29^, since they dramatically reduce the amount of possible misannotation caused by automated tools. In metabolic models, annotation is referred to as gene-protein-reaction associations. Compared to the genome annotations, ~24–28% of the gene-protein-reaction associations in the models were improved during the manual curation process, increasing the accuracy of predicted metabolic phenotypes (Supplementary Table 1).

Metabolic models are broadly used because they can simulate the metabolism of organisms with minimal experimental data, such as substrate uptake rates^32^. When such data is not available, it can be generalized using experimental data from closely related organisms^37^. For this purpose, we reconstructed a model of *L. crescens* BT-1, a closely related, culturable microorganism. We generated constraints to simulate growth phenotypes based on BT-1 experimental data^33^. The BT-1 model was validated by accurately predicting growth rates across four culture media compositions (i.e., BM-7, M13, M14, and M15) and multiple substrates (e.g., fumarate, glucose, oxoglutarate) (Fig. 2). In confirmation of these findings, citrate was recently discovered to improve the growth rate of BT-1 experimentally^33^. In addition, we experimentally confirmed our serine and other amino acid predictions in *L. crescens* and in the *C*Las-hairy root-assays.

After successful validation of the BT-1 model we performed simulations to understand *C*Las metabolism. We found an interwoven effect of media composition on phenotypic traits, such as growth rate and metabolic production capabilities, which identified citrate and amino acids, such as glycine, serine, proline, glutamine, and glutamate, as metabolites with a significant effect on *C*Las and *L. crescens* BT-1 growth (Figs 2d and 3d). Furthermore, it has been observed that metabolites, such as glycine, serine, citrate, glycine, glutamic acid, inositol, and malate, significantly change their concentration during *C*Las habitation in the psyllid host^38^ and citrate, histidine, phenylalanine, and sucrose during infection of *C. sinensis*^39^.

Genes essential for *L. crescens* BT-1 growth in vitro that are absent in *C*Las may be responsible for the failure of maintaining *C*Las strains in culture^40^. The lack of these genes suggests that *C*Las acquires aromatic amino acids, vitamins, saccharides, and fatty acids from their hosts, as previously shown in other microbial communities^28^. We identified over 109 metabolic reactions that are present in *L. crescens* BT-1 but missing across all *C*Las strains (Supplementary Table 2). Previous studies have also suggested that *C*Las species lost the ability to synthesize proline, phenylalanine, tryptophan, cysteine, tyrosine, and histidine in addition to other translation components that may compromise regulatory systems^36,41^. We confirmed all of these auxotrophies and found that the proline, aspartate, arginine and serine auxotrophies are *C*Las strain-specific. Additionally, we predicted auxotrophies for steroids, cofactors and vitamins such as biotin, carnitine, choline, coniferol, riboflavin, and thiamin (Supplementary Fig. 2).

Using genome-scale metabolic models, we focused on understanding the metabolic behavior of *C*Las when it inhabits its two hosts. Application of modeling constraints based on *C*Las expression data enabled simulation and identification of metabolic changes at various functional stages, for example when *C*Las inhabits the psyllid or the plant. In vivo data (i.e., metagenomics and metatranscriptomics) are reliable sources of information for modeling uncultivable microorganisms. Host-specific (psyllid or plant expression data) constrained models predicted growth rates ~70% slower than media-constrained models. The predicted growth rate in the psyllid was higher than in the plant, as was previously observed experimentally^42^. These findings suggest different behaviors of *C*Las are dependent on its host (Fig. 4). *C*Las grows faster while inhabiting the psyllid, activating pathways related to nucleotide sugar metabolism, streptomycin biosynthesis, polyketide sugar unit biosynthesis, and cell envelope synthesis. Among these pathways, enzymes related to cell wall oligosaccharide enzymes were identified by screening all predicted flux distributions. It has been observed that *C*Las uses these enzymes to synthesize polysaccharides and thrive under different environments, especially in the presence of competitive bacterial biological agents^43–45^. These results suggest that in the psyllid host, *C*Las may activate the synthesis of antibiotics and antimicrobial precursors to compete with endogenous bacteria in the psyllid gut. On the other hand, in the citrus phloem, *C*Las may activate pathways that counteract plant defense mechanisms, such as the production of reactive oxygen species by NADPH oxidase^46,47^, or the synthesis of antimicrobial peptides and long chain fatty acids^48,49^ by activating reactions that depletes intermediaries of these toxic metabolites (e.g., orotic acid dehydrogenase, l-aspartate and glycolate oxidases). We also found that fatty acid metabolism was highly activated in *C*Las from citrus phloem samples, including enoyl-acyl reductase, which has been associated with antibiotic resistance^50^.

Significant progress has been made toward understanding the interactions between *C*Las and its hosts, and systems biology and omics tools can help to further unravel metabolic mechanisms associated with HLB initiation and progression, as well as to identify targets in *C*Las that can be used to develop HLB management strategies. Our results are consistent with and expand on prior findings. For example, other studies in *L. cresce*ns have shown that supplementation of amino acids to the culture media increases growth rates^36^. Gene essentiality simulations (Fig. 5) agree with previous findings, revealing ABC transporters, cell envelope biosynthesis, and fatty acid metabolism to be crucial subsystems for *C*Las^36,49,51^. Additionally, we found genetic targets in metabolic pathways whose inhibition may block the growth of *C*Las, thus preventing spread of this destructive disease (Supplementary Fig. 7). The systems biology tools presented here allow for the simulation of thousands of conditions, by applying environmental and/or genetic constraints, which reveal the vulnerabilities of *C*Las across various environments and improve our ability to guide future research and management efforts to combat this pathogen.

## Methods

### Draft model reconstruction and manual curation

Reconstructions are biochemically and genomically structured networks that contain information about associations among genes, reaction stoichiometry, and reaction reversibility. Here, we used a semi-automated process to reconstruct high-quality metabolic models, which comprises four fundamental steps: (i) creation of an automated draft reconstruction, (ii) draft refinement by manual curation, (iii) conversion from reconstruction to mathematical model, and (iv) model evaluation.

Semi-automatic reconstruction methods reduce building time, while maintaining high-quality architecture and prediction accuracy^52^. This method results in draft models, which require refinement through manual curation. Draft models are generated based on protein-homology comparison between each protein sequence in the genome of the target microorganism (e.g., *C*Las) and the protein sequence of a manually curated reference model(s).

The reference models used here were chosen from the BiGG Database^53^. Supplementary Fig. 1 shows the phylogenetic relationships between *Liberibacter* strains and bacteria with available reference models in the repository. *Pseudomonas putida* KT2440, *i*JN746^54^ was the closest related microorganism to *Liberibacter*, followed by *Yersinia pestis* CO92, *i*PC815^55^ and the model of *Escherichia coli* str. K-12 substr. MG1655, *i*ML1515^56^. Table 1 shows the genome IDs of the protein sequences of *C*Las strains A4, FL17, gxpsy, psy62, YCPsy and *L. crescens* BT-1, which were used as input to The COBRA^32^ and RAVEN Toolboxes^57^ for MATLAB (The MathWorks Inc., Natick, MA).

Each metabolic reaction in the reconstructed models was manually curated for their correct gene-protein-reaction association (GPR) using protein BLAST^58^ to compare protein sequences between each strain of *Liberibacter* in the multi-strain model with sequences of *E. coli*, *P. putida*, and *Y. pestis* using UniProtKB/Swiss-Prot databases^59^. Transporter protein sequences were identified and compared using the TCBD database^60^. Metabolic reactions where no gene association could be found underwent another round of curation, where literature was reviewed to find evidence for the presence/absence of these proteins. Reactions with no supportive literature or matching sequences were included in the model for gap filling to ensure the completeness of relevant pathways^37^. The Supplementary Table 10 shows the reaction without GPR included in the models and a pie chart of the distribution of reactions across subsystems.

The manual curation process was followed by model evaluation and validation. The reconstructions were analyzed for connectivity, mass and charge balance and converted into a functional mathematical model for simulation using The COBRA Toolbox^32^. Metabolic models were shared following the standard protocols for computational analysis^61^.

### Constraints and growth simulations

The seven *C*Las and BT-1 metabolic model reconstructions were constrained identically using the culture media BM-7, M13, M14, and M15^33^. All media compositions were simulated by setting a lower bound of −100 (allowing unlimited uptake) on the exchange reactions for Co^2+^, Fe^2+^, H^+^, H_2_O, Na^+^, NH_4_, PO_4_, SO_4_. Supplementary Table 3 shows the media compositions and applied constraints for each culture media. Conversion from optical density to dry weight was based on the BIONUMB3R5 database^62^. Growth simulations were performed using the flux balance analysis procedure^32^. Constraints on biomass composition were imported from the reference model of *P. putida* KT2440, *i*JN746^54^. Stoichiometry of the biomass composition was estimated to be 1 g of dry weight of biomass.

The model topology was evaluated following the constraint-based modeling standard protocol^32,35^. Methods listed under growth simulations of alternate substrates of Orth et al. protocol were used to calculate the degree of metabolite connectivity (D, Eq. 1)^35^, identifying metabolites with remarkable effect on the growth rate across the entire network. The matrix S is a feature in constraint-based modeling and its size is determined by the number of metabolites (rows) and reactions (columns) in the model. Reaction essentiality by metabolite was calculated by scanning the matrix S across all reactions. For the reactions in which each metabolite was found to participate the boundaries were set to zero and compound growth rate was estimated (*µ*_comp,metabolite_). The essentiality fraction was determined by the ratio between compound growth rate and the growth rate determined without any modification to the boundaries (wild-type growth rate, *µ*_WT_).

1 $$\begin{array}{l}D_{{\mathrm{connectivity}},i} = \mathop{\sum}\limits_{S_{{\mathrm{bin}},i,:}} \\ \left( {A_{{\mathrm{comp}}}} \right)\!\!:A_{{\mathrm{comp}}} = S_{{\mathrm{bin}}} \ast S_{{\mathrm{bin}}}^T\\ \gamma _{{\mathrm{benefitial}},i} = \frac{{\mu _{{\mathrm{comp}},i}}}{{\mu _{{\mathrm{WT}}}}}\end{array}$$

### Phenotypic experimental data

#### *Liberibacter crescens* cultivation

M15 media consists of CaCl_2_•2H_2_O (1320 mg/L), MgCl_2_ (1068.2 mg/L), MgSO_4_•7H_2_O (2778 mg/L), KCl (2240 mg/L), NaH_2_PO_4_•H_2_O (1007 mg/L), L-alanine (447.24 mg/L), L-arginine-HCl (1777 mg/L), L-asparagine monohydrate (1075.45 mg/L), L-cysteine-2HCl (56.38 mg/L), L-glutamic acid (1502.2 mg/L), glycine (859.51 mg/L), L-histidine hydrochloride monohydrate (2366.11 mg/L), L-isoleucine (687.36 mg/L), L-leucine (592.89 mg/L), L-lysine-HCl (1464.85 mg/L), L-methionine (678.9 mg/L), L-phenylalanine (789.62 mg/L), L-proline (940.61 mg/L), l-threonine (459.8 mg/L), L-tryptophan (373.73 mg/L), L-tyrosine disodium salt (391.37 mg/L), L-valine (644.31 mg/L), betaine monohydrate (0.36 mg/L), dl-ornithine hydrochloride (293.39 mg/L), methionine sulfoxide (18.2 mg/L), D-biotin (0.1 mg/L), choline chloride (1000 mg/L), folic acid (0.2 mg/L), myo-inositol (0.2 mg/L), niacin (0.2 mg/L), D-calcium pantothenate (0.2 mg/L), para-aminobenzoic acid (0.2 mg/L), pyridoxine-HCl (0.2 mg/L), riboflavin (0.2 mg/L), thiamine-HCl (0.2 mg/L), L-aspartic acid (2500 mg/L), dl-serine (2500 mg/L), L-glutamine (358.04 mg/L), and citric acid (2500 mg/L)^33^. All ingredients were combined with the exception of tyrosine, which was first dissolved in 1 M HCl before being added. Once all ingredients were dissolved, the medium was adjusted to pH 5.92 with 5 M KOH and filter sterilized. Other derivatives of M15 (the different treatment media types) were prepared in the same way, but with differing concentrations of the components being examined (i.e., l-aspartic acid, dl-serine, l-glutamine, and citric acid). The concentrations of all other components were kept the same as the original M15 recipe. M15-basic media was made with minimal (0.1 mg/L) amounts of the treatment components (i.e., citrate, serine, and aspartate or citrate, serine, and glutamine) but was otherwise kept the same as the original M15 recipe.

*Liberibacter crescens* (type strain BT-1^T^, 5ATCC BAA-2481^T^5DSM T 26877) was used for all experiments^21^. Glycerol stocks of *L. crescens* strain BT-1 in BM-7 complex media were used to inoculate M15 media, which was then shaken at 150 r.p.m. and 28 °C for 3–5 days to grow sufficient quantities for the experiments. Bacteria were pelleted via centrifugation at 6000 rcf for 10 min, re-suspended in M15-basic medium, which does not contain the treatment components (citrate, serine, and aspartate or citrate, serine, and glutamine) and shaken at 150 r.p.m. and 28 °C for 1 h to remove any large traces of the treatment components. OD_600_ was measured, and bacteria were re-pelleted using the same conditions described above. Pelleted bacteria were re-suspended in sterile DI water and used to inoculate treatment media for growth to stationary phase: OD_600_ = 0.8. Treatment media tubes were grown in 5 mL volumes in 16 × 100 mm tubes at 150 r.p.m. and 28 °C. Growth was measured every 12–24 h for 300 h using a Spectronic-20 (Milton Roy, Houston, TX) spectrophotometer and OD_600_.

#### *C*Las-Citrus hairy root culturing and in vitro assays

The ex vivo *C*Las-citrus hairy root cultures were generated using methods described previously^63^, with *C*Las-infected sour orange tissues (*Citrusxaurantium* L.) as ex-plant/inoculum source for *C*Las. Briefly, quantitative polymerase chain reaction (qPCR) validated CLas containing sour orange were identified and 5–10 cm shoots were excised for hairy root induction. The cut-end of the ex-plant was inoculated with fresh culture of *Rhizobium rhizogenes* (American Type Culture Collection strain 15834, OD 0.5) under gentle vacuum infiltration (~200 kPa). *R. rhizogenes* is a soil bacterium that naturally transforms plant cells to induce hairy roots at the point of contact by reprogramming plant hormone signaling^64^. In citrus, hairy root formation typically occurs in ~90 days, and because of the vascular connectivity between the shoot ex-plant, CLas naturally migrates into the newly formed hairy roots. Presence of *C*Las in the hairy root cultures was further confirmed by qPCR, using *C*Las-specific primers as described below. To determine the effect of amino acids on *C*Las titers, in vitro assays were set up using the validated *C*Las-citrus hairy roots^63^. Briefly, the *C*Las-citrus hairy roots were surface sterilized with 70% ethanol and 2.5% sodium hypochlorite for five minutes followed by six washes with sterile water. Approximately 100 mg of *C*Las-citrus hairy roots were added to a multi-well culture plates and supplemented with B5 + amino acid cocktail (glycine, serine, proline, aspartate, glutamine, and glutamate) concentrations (0, 0.1, 1, and 5 µM). An oxytetracycline (500 p.p.m. = 500 mg/L) treatment was included as a *C*Las-inhibitor control for the in vitro assay. Four to five independent biological replicates were included for all treatments. The samples were vacuum infiltrated at 200 kPa for ~15 min to facilitate penetration of the media and nutrients into the hairy root cultures. The assay plates were placed on an orbital shaker at 40 r.p.m. at~25 °C and in dark. Fresh B5 medium was replaced at 3 and 5 days after incubation. Samples were collected at 0, 3, 5, and 7 days after treatment and flash-frozen in liquid nitrogen and stored at −80 °C until further use.

#### DNA extraction and qPCR analysis

All control and treated *C*Las-citrus hairy root cultures were lyophilized and homogenized in a MiniG 1600 (Spex Sample Prep) homogenizer at 1500 x r.p.m. for 30 s, with a single steel bead (two times, re-freezing samples at −80 °C in between). Total DNA extraction was carried out according to Almeyda et al.^65^. qPCR reactions were carried out in a CFX-384 Real-Time PCR Detection System (BioRad, Hercules, CA) with 25 ng of DNA as template, using Sso Advanced Universal SYBR Green Supermix (BioRad, Hercules, CA), and the following primers for citrus GAPC2 (CsiGAPC2-F 5′-TCTTGCCTGCTTTGAATGGA-3′and CsiGAPC2-R 5′-TGTGAGGTCAACCACTGCGACAT-3′) and for the β-subunit od *nrd*B gene from *C*Las, RNR (RNRf 5′-CATGCTCCATGAAGCTACCC-3′ and RNRr 5′-GGAGCATTTAACCCCACGAA-3′)^66^. The reactions were carried out under the following conditions: Initial denaturation 95 °C for 3 min, followed by 95 °C for 15 s and 55 °C for 30 s for 40 cycles, and a final extension at 65 °C for 5 s. Relative *C*Las titers were estimated using the ^ΔΔ^Ct method^67^. Briefly, the *C*Las Ct was first normalized to the housekeeping gene (GAPC2) to account for DNA template differences, and then to the 0 days Ct, which was set to 1 (or 100%). Growth rates at each time point were calculated using the initial *C*Las titer as a reference point. The minimal and maximal data were discarded before the analysis of variance (ANOVA) analysis. Calculations were performed using The Preprocessing Data and The Statistics and Machine Learning Toolboxes of MATLAB (The MathWorks Inc.).

### Expression data

RNA-sequencing data collected from environmental samples was used to constrain the *C*Las models. The samples were obtained from the phloem-enriched samples from different citrus cultivars and from Asian citrus psyllid (ACP) alimentary canals as described below. For each growth condition, the storage and consumption of starch, calculated using experimental data, were taken into account (Supplementary Table 6).

#### RNA extraction from citrus

Samples were harvested from 12 *C*Las-infected citrus trees grown in a greenhouse at the U.S. Horticultural Research Facility in Fort Pierce, Florida. Three trees were selected each from three different *Citrus* cultivars: Valencia orange (*Citrus sinensis* [L.] Osbeck) on Swingle citrumelo (*C. paradisi* Macf. X Poncirus trifoliate [L.] Raf.) rootstock, Tango mandarin (*Citrus reticulata* Blanco) on Sour orange rootstock, and Washington navel orange (*Citrus sinensis* [L.] Osbeck) on Sour orange rootstock (Supplementary Table 6). One to 2 years prior to sampling, the greenhouse trees were exposed to *C*Las-positive ACP for varying lengths of time between one week and one month, and *C*Las infection was verified using qPCR at or near the time of harvest.

Four pieces of budwood that were roughly one year old and ~15 cm long were harvested from each tree and immediately placed on ice. Within 15 min, they were sampled from as follows: budwood was removed from ice and sprayed with CVS brand Health Alcohol Free Liquid Bandage Spray (CVS, Woonsocket, RI) to prevent surface contamination. After drying for three minutes, the bark was peeled from each piece and the inside surface located away from the cut ends was quickly scraped with a razor blade twice—first to remove surface contamination and potential xylem and second to collect a phloem-enriched sample. Samples were immediately placed in PowerBead Tubes filled with Solution MBL and the Phenolic Separation Solution from an RNeasy PowerPlant Kit (Qiagen, Valencia, CA), which were held in a CoolRack (BioCision, San Rafael, CA) on dry ice. RNA was extracted using the RNeasy PowerPlant Kit following the kit protocol with two minutes of bead beating, eluted in 50 µL of RNase-free water, and stored at −80 °C for library preparation.

#### RNA extraction from Asian citrus psyllids

Approximately 100 adult ACP were collected from *C*Las-exposed colonies maintained at the U.S. Horticultural Research Facility in Fort Pierce, Florida using an aspirator. Alimentary canals were dissected from ACP in a weight-boat containing Solution PM1 from an RNeasy PowerMicrobiome Kit (Qiagen, Valencia, CA) placed on ice, and deposited in two PowerBead Tubes containing Solution PM1 (50 canals each) held in a CoolRack (BioCision) on dry ice. RNA was extracted using the RNeasy PowerMicrobiome Kit following the kit protocol with one minute of bead beating, eluted in 50 µL of RNase-free water, and stored at −80 °C for library preparation.

#### Library preparation and sequencing

Alimentary canal sample complementary DNA (cDNA) libraries were prepared using the ScriptSeq Complete Gold Kit (Yeast) (Illumina, San Diego, CA), following kit protocols and performing ribo-depletion. Citrus sample cDNA libraries were prepared using the ScriptSeq Complete Kit (Plant Leaf) (Illumina, San Diego, CA), again using provided protocols and ribo-depletion. In both cases, ScriptSeq Index PCR Primers (Illumina, San Diego, CA) were used for barcoding samples. RNA Sequencing was performed using Illumina’s HiSeq2500 platform. Raw RNA reads were trimmed using TrimGalore (version 0.4.4) including adapter removal and quality control: low-quality ends from reads (Phred score < 20) were trimmed and reads less than 20 bp were discarded. Next, read quality was checked using FastQC (version 0.11.7). To discard host and 16S rRNA reads, *C. maxima* (Burm.) Merr. genome (NCBI_Assembly: GCA_002006925.1) and bacterial 16S rRNA sequences (SILVA database: https://www.arb-silva.de/) were chosen as reference templates. Valid reads were aligned to reference templates using bowtie2 (version 2.3.4.1) with parameters set by the flag very-sensitive. Unmatched reads were picked out and converted to fastq format using samtools (version 1.8) and bam2fastq (http://www.hudsonalpha.org/gsl/information/software/bam2fastq), respectively. To count the FPKM (fragment per kilobase per million mapped reads), reads were mapped to 7 Liberibacter strains: *C*Las strains A4 (GCF_000590865.2), FL17 (GCF_000820625.1), psy62 (GCF_000023765.2), YCPsy (GCF_001296945.1), gxpsy (GCF_000346595.1), Ishi-1 (GCF_000829355.1) and *L. crescens* BT-1 (GCF_000325745.1).

## Supplementary material

Supplementary Information

Dataset 1

## Data Availability

The Liberibacter models are available at https://github.com/cristalzucsd/Liberibacter. Models were constrained using the traditional culture medium BG-7 and the optimized culture media M13, M14 and M15. All sequencing reads were deposited in the Sequence Read Archive under BioProject PRJNA509215, with specific numbers listed in Supplementary Table 5. Additionally, all supplemental materials are available at https://github.com/cristalzucsd/Liberibacter.
