# Supplementary material for: Linking metabolic phenotypes to pathogenic traits among “Candidatus Liberibacter asiaticus” and its hosts
Source: NPJ Syst Biol Appl. 2020 Aug 4;6:24. doi: 10.1038/s41540-020-00142-w (PMC7403731; doi:10.1038/s41540-020-00142-w)

Supplementary Table 1. Manually curated gene-protein-reactions associations

Reaction ID	Reaction Name	GPR	Subsystem
2DHGLCNkt_tpp	2DHGLCNkt tpp	(VBICanLib130745_0773)	Transport
4HTHRS	4-Hydroxy-L-threonine synthase	(VBICanLib130745_0382)	Vitamin B6 Metabolism
ACCOAC	Acetyl-CoA carboxylase	(VBICanLib130745_0420) and (VBICanLib130745_0160) and (VBICanLib130745_0661) and (VBICanLib130745_0161)	Fatty Acid Biosynthesis
ACGS	N-acetylglutamate synthase	(VBICanLib130745_0402) or (VBICanLib130745_0225)	Urea cycle/amino group metabolism
ACHBS	2-aceto-2-hydroxybutanoate synthase	(VBICanLib130745_0020)	Valine, Leucine, and Isoleucine Metabolism
ACOATA	Acetyl-CoA ACP transacylase	(VBICanLib130745_0719 and VBICanLib130745_0753)	Fatty Acid Biosynthesis
ACONT	Aconitate hydratase	(VBICanLib130745_0366)	TCA Cycle
ACONTa	Aconitase (half-reaction A, Citrate hydro-lyase)	(VBICanLib130745_0366)	TCA Cycle
ACONTb	Aconitase (half-reaction B, Isocitrate hydro-lyase)	(VBICanLib130745_0366)	TCA Cycle
ADSL1r	Adenylsuccinate lyase	(VBICanLib130745_0916)	Purine Metabolism
ADSL2r	Adenylosuccinate lyase	(VBICanLib130745_0916) or (VBICanLib130745_0077) or (VBICanLib130745_0280)	Purine Metabolism
AGPAT120	1-tetradecanoyl-sn-glycerol 3-phosphate O-acyltransferase (n-C12:0)	(VBICanLib130745_0669)	Glycerophospholipid Metabolism
AGPAT160	1-hexadecanoyl-sn-glycerol 3-phosphate O-acyltransferase (n-C16:0)	(VBICanLib130745_0669)	Glycerophospholipid Metabolism
AGPAT161	1-hexadec-7-enoyl-sn-glycerol 3-phosphate O-acyltransferase (n-C16:1)	(VBICanLib130745_0669)	Glycerophospholipid Metabolism
AGPAT180	1-octadecanoyl-sn-glycerol 3-phosphate O-acyltransferase (n-C18:0)	(VBICanLib130745_0669)	Glycerophospholipid Metabolism
AGPAT181	1-octadec-7-enoyl-sn-glycerol 3-phosphate O-acyltransferase (n-C18:1)	(VBICanLib130745_0669)	Glycerophospholipid Metabolism
AKGDH	2-Oxogluterate dehydrogenase	(VBICanLib130745_1002 and VBICanLib130745_1000 and VBICanLib130745_1001)	TCA Cycle
ALAALAr	D-alanine-D-alanine ligase (reversible)	(VBICanLib130745_1136)	Peptidoglycan Biosynthesis

ANPRT	Anthranilate phosphoribosyltransferase	(VBICanLib130745_0325)	Phenylalanine Tyrosine Tryptophan Biosynthesis
ANS	Anthranilate synthase	(VBICanLib130745_0325)	Phenylalanine Tyrosine Tryptophan Biosynthesis
ARGSS	Argininosuccinate synthase	(VBICanLib130745_0126)	Arginine and Proline Metabolism
ARGabcpp	L-arginine transport via ABC system (periplasm)	((VBICanLib130745_1064) and (VBICanLib130745_0058)) or ((VBICanLib130745_1063) and (VBICanLib130745_1064) and (VBICanLib130745_0058) and (VBICanLib130745_0057))	Transport
ASAD	Aspartate-semialdehyde dehydrogenase	(VBICanLib130745_0204)	Lysine Biosynthesis
ASNS1	Asparagine synthase (glutamine-hydrolysing)	(VBICanLib130745_0238)	Alanine and Aspartate Metabolism
ASPabcpp	L-aspartate transport via ABC system (periplasm)	(VBICanLib130745_0057 and VBICanLib130745_0056 and VBICanLib130745_1063) or (VBICanLib130745_1064)	Transport
ATPPRT	ATP phosphoribosyltransferase	(VBICanLib130745_0382)	Histidine Metabolism
ATPS4rpp	ATP synthase (four protons for one ATP) (periplasm)	(VBICanLib130745_0631 and VBICanLib130745_0634 and VBICanLib130745_1039 and VBICanLib130745_0632 and VBICanLib130745_0630 and VBICanLib130745_0633)	Oxidative Phosphorylation
BZt1pp	Benzoate transport in via proton symport (periplasm)	(VBICanLib130745_0297)	B-Ketoadipate pathway
CHLabcpp	Choline transport via ABC system (periplasm)	(VBICanLib130745_0244 and VBICanLib130745_0243) or (VBICanLib130745_0541) or (VBICanLib130745_0653) or (VBICanLib130745_0691)	Transport
CHOLS_ex	CHOLS LPAREN ex RPAREN	(VBICanLib130745_0382)	Transport
DAPE	Diaminopimelate epimerase	(VBICanLib130745_0534)	Lysine Biosynthesis

CHOLSabc	CHOLSabc	(VBICanLib130745_0245) or (VBICanLib130745_0244 and VBICanLib130745_0243) or (VBICanLib130745_0541) or (VBICanLib130745_0653) or (VBICanLib130745_0691)	Glycine, Serine and threonine metabolism
CITtex	Citrate transport via diffusion (extracellular to periplasm)	(VBICanLib130745_0840)	Transport
COBALT2tex	Cobalt (Co+2) transport via diffusion (extracellular to periplasm)	(VBICanLib130745_0652)	Transport
COBALT2tpp	Cobalt transport in via permease (no H+)	(VBICanLib130745_0652)	Transport
CS	Citrate synthase	(VBICanLib130745_0243 and VBICanLib130745_0244)	TCA Cycle
CTPS2	CTP synthase (glutamine)	(VBICanLib130745_0243 and VBICanLib130745_0244)	Pyrimidine Metabolism
CYO1b	CYO1b	(VBICanLib130745_0391 and VBICanLib130745_0392)	Oxidative Phosphorylation
CYSabcpp	L-cysteine uptake via ABC system (periplasm)	(VBICanLib130745_0719 and VBICanLib130745_0753)	Transport
DAPDC	Diaminopimelate decarboxylase	(VBICanLib130745_0281)	Lysine Biosynthesis
DASYN120	CDP-diacylglycerol synthetase (n-C12:0)	(VBICanLib130745_0467)	Glycerophospholipid Metabolism
DASYN160	CDP-diacylglycerol synthetase (n-C16:0)	(VBICanLib130745_0467)	Glycerophospholipid Metabolism
DASYN161	CDP-diacylglycerol synthetase (n-C16:1)	(VBICanLib130745_0467)	Glycerophospholipid Metabolism
DASYN180	CDP-diacylglycerol synthetase (n-C18:0)	(VBICanLib130745_0467)	Glycerophospholipid Metabolism
DASYN181	CDP-diacylglycerol synthetase (n-C18:1)	(VBICanLib130745_0467)	Glycerophospholipid Metabolism
DB4PS	3,4-Dihydroxy-2-butanone-4-phosphate synthase	(VBICanLib130745_0266)	Riboflavin Metabolism
DDPA	3-deoxy-D-arabino-heptulosonate 7-phosphate synthetase	(VBICanLib130745_0575)	Phenylalanine Tyrosine Tryptophan Biosynthesis
DHPM1	Dihydropyrimidinase (5,6-dihydrouracil)	(VBICanLib130745_0198)	Pantothenate and CoA Biosynthesis

DMATT	Dimethylallyltranstransferase	(VBICanLib130745_1062) or (VBICanLib130745_0490)	Steroid biosynthesis
DNAMTn2r	DNA (cytosine-5-)-methyltransferase	(VBICanLib130745_0736) or (VBICanLib130745_0735)	Methionine Metabolism
DNTPPA	Dihydroneopterin triphosphate pyrophosphatase	(VBICanLib130745_0224)	Folate Biosynthesis
DPCOAK	Dephospho-CoA kinase	(VBICanLib130745_0422)	Pantothenate and CoA Biosynthesis
DPR	2-dehydropantoate 2-reductase	(VBICanLib130745_1050)	Pantothenate and CoA Biosynthesis
DTMPK	DTMP kinase	(VBICanLib130745_0110)	Pyrimidine Metabolism
DURAD	Dihydrouracil dehydrogenase (NADP)	(VBICanLib130745_0347)	Pantothenate and CoA Biosynthesis
DUTPDP	DUTP diphosphatase	(VBICanLib130745_0975)	Pyrimidine Metabolism
DXPS	1-deoxy-D-xylulose 5-phosphate synthase	(VBICanLib130745_0571)	Steroid biosynthesis
EAR100y	Enoyl-[acyl-carrier-protein] reductase (NADPH) (n-C10:0)	(VBICanLib130745_0379) or (VBICanLib130745_1123)	Fatty Acid Biosynthesis
EAR120y	Enoyl-[acyl-carrier-protein] reductase (NADPH) (n-C12:0)	(VBICanLib130745_1123) or (VBICanLib130745_0379) or (VBICanLib130745_0718)	Fatty Acid Biosynthesis
EAR121y	Enoyl-[acyl-carrier-protein] reductase (NADPH) (n-C12:1)	(VBICanLib130745_0244 and VBICanLib130745_0243)	Fatty Acid Biosynthesis
EAR160y	Enoyl-[acyl-carrier-protein] reductase (NADPH) (n-C16:0)	(VBICanLib130745_0379) or (VBICanLib130745_1123)	Fatty Acid Biosynthesis
EAR161y	Enoyl-[acyl-carrier-protein] reductase (NADPH) (n-C16:1)	(VBICanLib130745_0541 and VBICanLib130745_0244) or (VBICanLib130745_0243)	Fatty Acid Biosynthesis
EAR180y	Enoyl-[acyl-carrier-protein] reductase (NADPH) (n-C18:0)	(VBICanLib130745_0379) or (VBICanLib130745_1123)	Fatty Acid Biosynthesis
EAR181y	Enoyl-[acyl-carrier-protein] reductase (NADPH) (n-C18:1)	(VBICanLib130745_0379) or (VBICanLib130745_1123)	Fatty Acid Biosynthesis
EAR40y	Enoyl-[acyl-carrier-protein] reductase (NADPH) (n-C4:0)	(VBICanLib130745_0379) or (VBICanLib130745_1123)	Fatty Acid Biosynthesis
EAR60y	Enoyl-[acyl-carrier-protein] reductase (NADPH) (n-C6:0)	(VBICanLib130745_0392 and VBICanLib130745_0391 and VBICanLib130745_0389 and VBICanLib130745_0390)	Fatty Acid Biosynthesis

EAR80y	Enoyl-[acyl-carrier-protein] reductase (NADPH) (n-C8:0)	(VBICanLib130745_0243 and VBICanLib130745_0244)	Fatty Acid Biosynthesis
ETHSO3abcpp	Ethanesulfonate transport via ABC system (periplasm)	(VBICanLib130745_0653 and VBICanLib130745_0652 and VBICanLib130745_0787)	Transport
FDH	Formate dehydrogenase	(VBICanLib130745_0787 and VBICanLib130745_0786 and VBICanLib130745_0785)	Methane Metabolism
FE2abc	Iron (II) transport via ABC system	(VBICanLib130745_0243) or (VBICanLib130745_0691 and VBICanLib130745_0690 and VBICanLib130745_0687)	Transport
FERtex	FERtex	(VBICanLib130745_0501)	Transport
FERtpp	FERtpp	(VBICanLib130745_0501)	Transport
FTHFD	Formyltetrahydrofolate deformylase	(VBICanLib130745_0591)	One Carbon pool by folate
FUM	Fumarase	(VBICanLib130745_0132 or VBICanLib130745_0299)	TCA Cycle
FUMtex	Fumarate transport via diffusion (extracellular to periplasm)	(VBICanLib130745_0077)	Transport
G1SAT	Glutamate-1-semialdehyde aminotransferase	(VBICanLib130745_0345)	Porphyrin and Chlorophyll Metabolism
G3PAT120	Glycerol-3-phosphate acyltransferase (C12:0)	(VBICanLib130745_0719)	Glycerophospholipid Metabolism
G3PAT160	Glycerol-3-phosphate acyltransferase (C16:0)	(VBICanLib130745_0719)	Glycerophospholipid Metabolism
G3PAT161	Glycerol-3-phosphate acyltransferase (C16:1)	(VBICanLib130745_0719)	Glycerophospholipid Metabolism
G3PAT180	Glycerol-3-phosphate acyltransferase (C18:0)	(VBICanLib130745_0719)	Glycerophospholipid Metabolism
G3PAT181	Glycerol-3-phosphate acyltransferase (C18:1)	(VBICanLib130745_0719)	Glycerophospholipid Metabolism
GART	GAR transformylase-T	(VBICanLib130745_0041)	Purine Metabolism
GLCNt2rpp	D-gluconate transport via proton symport, reversible (periplasm)	(VBICanLib130745_0254)	Transport

GLCabcpp	D-glucose transport via ABC system (periplasm)	(VBICanLib130745_0058) or (VBICanLib130745_0243) or (VBICanLib130745_0691)	Transport
GLNS	Glutamine synthetase	((VBICanLib130745_1063) and (VBICanLib130745_1064) and (VBICanLib130745_0058)) or (VBICanLib130745_0653)	Glutamate Metabolism
GLNTRAT	Glutamyl-tRNA(Gln) amidotransferase	(VBICanLib130745_0920 and VBICanLib130745_0922 and VBICanLib130745_0921)	Glutamate Metabolism
GLUSy	Glutamate synthase (NADPH)	(VBICanLib130745_0178)	Glutamate Metabolism
GLUt4pp	Na+/glutamate symport (periplasm)	(VBICanLib130745_0907)	Transport
GLXCL	Glyoxalate carboligase	(VBICanLib130745_0192)	Glycolate metabolism
GLYALDtex	Glyceraldehyde transport via diffusion (extracellular to periplasm)	(VBICanLib130745_0410) or (VBICanLib130745_0057)	Transport
GLYBabcpp	Glycine betaine transport via ABC system (periplasm)	(VBICanLib130745_0297)	Arginine and Proline Metabolism
GLYCK	Glycerate kinase	(VBICanLib130745_0850)	Glycine, Serine and threonine metabolism
GLYCL	Glycine Cleavage System	(VBICanLib130745_0569) or (VBICanLib130745_1002) or (VBICanLib130745_1004) or (VBICanLib130745_0569) or (VBICanLib130745_1002) or (VBICanLib130745_1004)	Glycine, Serine and threonine metabolism
GLYCLTt2rpp	Glycolate transport via proton symport, reversible (periplasm)	(VBICanLib130745_0569) or (VBICanLib130745_1002) or (VBICanLib130745_1004) or (VBICanLib130745_0569) or (VBICanLib130745_1002) or (VBICanLib130745_1004)	Transport
GLYCLTtex	Glycolate transport via diffusion (extracellular to periplasm)	(VBICanLib130745_0569) or (VBICanLib130745_1002) or (VBICanLib130745_1004) or (VBICanLib130745_0569) or (VBICanLib130745_1002) or (VBICanLib130745_1004)	Transport

GLYCL_2	Glycine cleavage system, cytosol	(VBICanLib130745_0569) or (VBICanLib130745_1002) or (VBICanLib130745_1004) or (VBICanLib130745_0569) or (VBICanLib130745_1002) or (VBICanLib130745_1004)	Nitrogen Metabolism
GLYCTO1	Glycolate oxidase	(VBICanLib130745_0484)	Glycolate metabolism
GLYTRS	Glycyl-tRNA synthetase	(VBICanLib130745_1045 and VBICanLib130745_0488)	Glycine, Serine and threonine metabolism
GMPS2	GMP synthase	(VBICanLib130745_1045 and VBICanLib130745_0488)	Purine Metabolism
GND	Phosphogluconate dehydrogenase	(VBICanLib130745_0528) or (VBICanLib130745_0299)	Pentose Phosphate Pathway
GRTT	Geranyltranstransferase	(VBICanLib130745_1062) or (VBICanLib130745_0490)	Steroid biosynthesis
GTHS	Glutathione synthetase	(VBICanLib130745_0549)	Glutamate Metabolism
GTPCII	GTP cyclohydrolase II	(VBICanLib130745_0266)	Riboflavin Metabolism
HCO3_NAt	Bicarbonate transport (Na/HCO3 cotransport)	(VBICanLib130745_0858)	Transport
HEMEOS	Heme O synthase	(VBICanLib130745_0267)	Porphyrin and Chlorophyll Metabolism
HMBS	Hydroxymethylbilane synthase	(VBICanLib130745_0986)	Porphyrin and Chlorophyll Metabolism
HMGL	Hydroxymethylglutaryl-CoA lyase	(VBICanLib130745_0081)	Synthesis and degradation of Ketones Bodies
HPPK	2-amino-4-hydroxy-6-hydroxymethyldihydropteridine diphosphokinase	(VBICanLib130745_1106)	Folate Biosynthesis
HXAtex	Hexanoate transport via diffusion (extracellular to periplasm)	(VBICanLib130745_0858)	Transport
ICDHyr	Isocitrate dehydrogenase (NADP)	(VBICanLib130745_0834)	TCA Cycle
ICITtex	Citrate transport via diffusion extracellular to periplasm	(VBICanLib130745_0909)	Transport
ICL	Isocitrate lyase	(VBICanLib130745_0396)	TCA Cycle
IGPS	Indole-3-glycerol-phosphate synthase	(VBICanLib130745_0020)	Phenylalanine Tyrosine Tryptophan Biosynthesis
ILEabcpp	L-isoleucine transport via ABC system (periplasm)	(VBICanLib130745_0909)	Transport
IPDPS	1-hydroxy-2-methyl-2-(E)-butenyl 4-diphosphate reductase (ipdp)	(VBICanLib130745_0979)	Steroid biosynthesis

IPMD	3-isopropylmalate dehydrogenase	(VBICanLib130745_0834)	Valine, Leucine, and Isoleucine Metabolism
IPPMIa	3-isopropylmalate dehydratase	(VBICanLib130745_0366) or (VBICanLib130745_0750)	Valine, Leucine, and Isoleucine Metabolism
IPPMIb	2-isopropylmalate hydratase	(VBICanLib130745_0366) or (VBICanLib130745_0750)	Valine, Leucine, and Isoleucine Metabolism
ISETACtex	Isethionate transport via diffusion (extracellular to periplasm)	(VBICanLib130745_0653)	Transport
KARA1	Ketol-acid reductoisomerase (2,3-dihydroxy-3-methylbutanoate)	(VBICanLib130745_1170)	Valine, Leucine, and Isoleucine Metabolism
KARA2	Ketol-acid reductoisomerase (2-Acetolactate)	(VBICanLib130745_1170)	Valine, Leucine, and Isoleucine Metabolism
KAS14	Beta-ketoacyl-ACP synthase	(VBICanLib130745_0378)	Fatty Acid Biosynthesis
LEUTRS	Leucyl-tRNA synthetase	(VBICanLib130745_0439)	Valine, Leucine, and Isoleucine Metabolism
LEUabcpp	L-leucine transport via ABC system (periplasm)	(VBICanLib130745_0909 and VBICanLib130745_1112)	Transport
LYSTRS	Lysyl-tRNA synthetase	(VBICanLib130745_1069)	Lysine Biosynthesis
LYSabcpp	L-lysine transport via ABC system (periplasm)	(VBICanLib130745_0889)	Lysine Metabolism
MACPD	Malonyl-ACP decarboxylase	(VBICanLib130745_0378)	Fatty Acid Biosynthesis
MSO3abcpp	Methanesulfonate transport via ABC system (periplasm)	(VBICanLib130745_0652)	Transport
MTHFC	Methenyltetrahydrofolate cyclohydrolase	(VBICanLib130745_0745)	One Carbon pool by folate
MTHFD	Methylenetetrahydrofolate dehydrogenase (NADP)	(VBICanLib130745_0745)	One Carbon pool by folate
MHPGLUT	5 methyltetrahydropteroyltriglutamate homocysteine S methyltransferase	(VBICanLib130745_1034)	Methionine Metabolism

NADH16pp	NADH dehydrogenase (ubiquinone-8 & 3 protons) (periplasm)	(VBICanLib130745_0781 and VBICanLib130745_0782 and VBICanLib130745_0783 and VBICanLib130745_0784 and VBICanLib130745_0785 and VBICanLib130745_0786 and VBICanLib130745_0787 and VBICanLib130745_0788 and VBICanLib130745_0789 and VBICanLib130745_0790 and VBICanLib130745_0791 and VBICanLib130745_0792 and VBICanLib130745_0793 and VBICanLib130745_0794)	Oxidative Phosphorylation
NADK	NAD kinase	(VBICanLib130745_0491)	Nicotinate and Nicotinamide
NDPK1	Nucleoside-diphosphate kinase (ATP:GDP)	(VBICanLib130745_0299)	Purine Metabolism
NDPK2	Nucleoside-diphosphate kinase (ATP:UDP)	(VBICanLib130745_0299)	Pyrimidine Metabolism
NDPK3	Nucleoside-diphosphate kinase (ATP:CDP)	(VBICanLib130745_0299)	Pyrimidine Metabolism
NDPK4	Nucleoside-diphosphate kinase (ATP:dTDP)	(VBICanLib130745_0299)	Pyrimidine Metabolism
NDPK5	Nucleoside-diphosphate kinase (ATP:dGDP)	(VBICanLib130745_0299)	Purine Metabolism
NDPK6	Nucleoside-diphosphate kinase (ATP:dUDP)	(VBICanLib130745_0299)	Pyrimidine Metabolism
NDPK7	Nucleoside-diphosphate kinase (ATP:dCDP)	(VBICanLib130745_0299)	Pyrimidine Metabolism
NDPK8	Nucleoside-diphosphate kinase (ATP:dADP)	(VBICanLib130745_0299)	Purine Metabolism
NNATr	Nicotinate-nucleotide adenylyltransferase	(VBICanLib130745_0598)	Nicotinate and Nicotinamide
NO2t2rpp	Nitrite transport in via proton symport, reversible (periplasm)	(VBICanLib130745_0965)	Transport
OIVD1r	2-oxoisovalerate dehydrogenase (acylating; 4-methyl-2-oxopentaoate)	(VBICanLib130745_1000)	Valine, leucine and isoleucine degradation

OIVD2	2-oxoisovalerate dehydrogenase (acylating; 3-methyl-2-oxobutanoate)	(VBICanLib130745_1000)	Valine, leucine and isoleucine degradation
OIVD3	2-oxoisovalerate dehydrogenase (acylating; 3-methyl-2-oxopentanoate)	(VBICanLib130745_1000)	Valine, leucine and isoleucine degradation
ORNCD	Ornithine cyclodeaminase	(VBICanLib130745_0989)	Arginine and Proline Metabolism
ORNabcpp	Ornithine transport via ABC system (periplasm)	(VBICanLib130745_0058)	Lysine Metabolism
ORPT	Orotate phosphoribosyltransferase	(VBICanLib130745_0961)	Pyrimidine Metabolism
PANTS	Pantothenate synthase	(VBICanLib130745_0626)	Pantothenate and CoA Biosynthesis
PDH	Pyruvate dehydrogenase	(VBICanLib130745_0569)	Glycolysis
PDX5POi	Pyridoxine 5-phosphate oxidase	(VBICanLib130745_1122)	Vitamin B6 Metabolism
PGM	Phosphoglycerate mutase	(VBICanLib130745_0173)	Gluconeogenesis
PGMT	Phosphoglucomutase	(VBICanLib130745_1058)	Glycolysis
PMDPHT	Pyrimidine phosphatase	(VBICanLib130745_0050)	Riboflavin Metabolism
PPA	Inorganic diphosphatase	(VBICanLib130745_0124)	Oxidative Phosphorylation
PPBNGS	Porphobilinogen synthase	(VBICanLib130745_0257)	Porphyrin and Chlorophyll Metabolism
PPPGO	Protoporphyrinogen oxidase (aerobic)	(VBICanLib130745_1023)	Porphyrin and Chlorophyll Metabolism
PRAGSr	Phosphoribosylglycinamide synthase	(VBICanLib130745_0096)	Purine Metabolism
PRAIi	Phosphoribosylanthranilate isomerase (irreversible)	(VBICanLib130745_0020)	Phenylalanine Tyrosine Tryptophan Biosynthesis
PRFGS	Phosphoribosylformylglycinamidine synthase	(VBICanLib130745_0912)	Purine Metabolism
PSP_L	Phosphoserine phosphatase (L-serine)	(VBICanLib130745_0900)	Glycine, Serine and threonine metabolism
RIBabcpp	D-ribose transport via ABC system (periplasm)	(VBICanLib130745_0691) or (VBICanLib130745_0653)	Transport
RPE	Ribulose 5-phosphate 3-epimerase	(VBICanLib130745_0917)	Pentose Phosphate Pathway
RPI	Ribose-5-phosphate isomerase	(VBICanLib130745_1003)	Pentose Phosphate Pathway
SDPTA	Succinyldiaminopimelate transaminase	(VBICanLib130745_0345)	Lysine Biosynthesis
SERAT	Serine O-acetyltransferase	(VBICanLib130745_0562)	Cysteine Metabolism
SERTRS	Seryl-tRNA synthetase	(VBICanLib130745_0564)	Glycine, Serine and threonine metabolism
SHCHF	Sirohydrochlorin ferrochelatase	(VBICanLib130745_0636)	Porphyrin and Chlorophyll Metabolism
SOTA	Succinylornithine transaminase	(VBICanLib130745_0345)	Arginine and Proline Metabolism
SUCD1	Succinate dehydrogenase	(VBICanLib130745_0992) and (VBICanLib130745_0993) and (VBICanLib130745_0991)	TCA Cycle

SULR	Sulfite reductase (NADPH2)	(VBICanLib130745_0902)	Sulfur Metabolism
SULabcpp	Sulfate transport via ABC system (periplasm)	(VBICanLib130745_0822)	Sulfur Metabolism
TOLtpp	TOLtpp	(VBICanLib130745_0058)	Transport
TRSARr	Tartronate semialdehyde reductase	(VBICanLib130745_0528)	Glycolate metabolism
UPPN	B-ureidopropionase	(VBICanLib130745_1048)	Pantothenate and CoA Biosynthesis
UREA	Urease	(VBICanLib130745_0814)	Urea cycle/amino group metabolism
VANKpp	VANKpp	(VBICanLib130745_0909)	Transport
VANLNtex	VANLNtex	(VBICanLib130745_0528)	Transport
VANLTtex	VANLTtex	(VBICanLib130745_0691) or (VBICanLib130745_0243)	Transport
pentso3abcpp	Pentso3abcpp	(VBICanLib130745_0653)	Transport
ACOLIPAabctex	Arabinose modified core oligosaccharide lipid A transport via ABC system (periplasm to extracellular)	(VBICanLib130745_0302 and VBICanLib130745_0501) or (VBICanLib130745_0715) or (VBICanLib130745_0019) or (VBICanLib130745_0541)	Lipopolysaccharide Biosynthesis / Recycling
ALATA_D2	D-alanine transaminase	(VBICanLib130745_0253)	Cofactor and Prosthetic Group Biosynthesis
ALATA_L2	Alanine transaminase	(VBICanLib130745_0253)	Cofactor and Prosthetic Group Biosynthesis
AMPTASECG	Alanyl aminopeptidase (cys-gly)	(VBICanLib130745_0301)	Cysteine Metabolism
AMPTASEPG	Aminopeptidase (pro-gly)	(VBICanLib130745_0301)	Arginine and Proline Metabolism
ASCBptspp	L-ascorbate transport via PEP:Pyr PTS (periplasm)	(VBICanLib130745_0057 and VBICanLib130745_0056 and VBICanLib130745_1063 and VBICanLib130745_1063) or (VBICanLib130745_1125)	Transport, Inner Membrane
BUTSO3abcpp	Butanesulfonate transport via ABC system (periplasm)	(VBICanLib130745_0653 and VBICanLib130745_0652 and VBICanLib130745_0244)	Transport, Inner Membrane
CLIPAabctex	Cold lipid A transport via ABC system (periplasm to extracellular)	(VBICanLib130745_0517 and VBICanLib130745_0302 and VBICanLib130745_0501 and VBICanLib130745_0500 and VBICanLib130745_0304 and VBICanLib130745_0361)	Lipopolysaccharide Biosynthesis / Recycling

COLIPAPabctex	Core oligosaccharide lipid A diphosphate transport via ABC system (periplasm to extracellular)	(VBICanLib130745_0302 and VBICanLib130745_0500 and VBICanLib130745_0501)	Lipopolysaccharide Biosynthesis / Recycling
COLIPAabctex	Core oligosaccharide lipid A transport via ABC system (periplasm to extracellular)	(VBICanLib130745_0302 and VBICanLib130745_0500 and VBICanLib130745_0501)	Lipopolysaccharide Biosynthesis / Recycling
CRNDabcpp	D-carnitine transport via ABC system (periplasm)	(VBICanLib130745_0243 and VBICanLib130745_0244)	Transport, Inner Membrane
CYSSADS	L-cysteine sulfinic acid desulfurase	(VBICanLib130745_1043) or (VBICanLib130745_1012)	Cysteine Metabolism
ECA4COLIPAabctex	Enterobacterial common antigen (x4) core oligosaccharide lipid A transport via ABC system (periplasm to extracellular)	(VBICanLib130745_0500 and VBICanLib130745_0517 and VBICanLib130745_0501 and VBICanLib130745_0302)	Lipopolysaccharide Biosynthesis / Recycling
ENLIPAabctex	Phosphoethanolamine lipid A transport via ABC system (periplasm to extracellular)	(VBICanLib130745_0501 and VBICanLib130745_0302 and VBICanLib130745_0500)	Lipopolysaccharide Biosynthesis / Recycling
GRXR	Glutaredoxin reductase	(VBICanLib130745_0910)	Unassigned
K2L4Aabcpp	KDO(2)-lipid IV A transport via ABC system (periplasm)	(VBICanLib130745_0850)	Transport, Inner Membrane
K2L4Aabctex	KDO(2)-lipid IV A transport via ABC system (periplasm to extracellular)	(VBICanLib130745_0850)	Lipopolysaccharide Biosynthesis / Recycling
KAS15	Beta-ketoacyl-ACP synthase (2)	(VBICanLib130745_0720)	Membrane Lipid Metabolism
KDOPS	3-deoxy -D-manno-octulosonic -acid 8-phosphate synthase	(VBICanLib130745_0575)	Lipopolysaccharide Biosynthesis / Recycling
LIPAabctex	Lipid A transport via ABC system (periplasm to extracellular)	(VBICanLib130745_0850)	Lipopolysaccharide Biosynthesis / Recycling

NADH17pp	NADH dehydrogenase (menaquinone-8 & 3 protons) (periplasm)	(VBICanLib130745_0781 and VBICanLib130745_0782 and VBICanLib130745_0783 and VBICanLib130745_0784 and VBICanLib130745_0785 and VBICanLib130745_0786 and VBICanLib130745_0787 and VBICanLib130745_0788 and VBICanLib130745_0789 and VBICanLib130745_0790 and VBICanLib130745_0791 and VBICanLib130745_0792 and VBICanLib130745_0793 and VBICanLib130745_0794)	Oxidative Phosphorylation
NADH18pp	NADH dehydrogenase (demethylmenaquinone-8 & 3 protons) (periplasm)	(VBICanLib130745_0781 and VBICanLib130745_0782 and VBICanLib130745_0783 and VBICanLib130745_0784 and VBICanLib130745_0785 and VBICanLib130745_0786 and VBICanLib130745_0787 and VBICanLib130745_0788 and VBICanLib130745_0789 and VBICanLib130745_0790 and VBICanLib130745_0791 and VBICanLib130745_0792 and VBICanLib130745_0793 and VBICanLib130745_0794)	Oxidative Phosphorylation
NADHHR	NADH hydratase	(VBICanLib130745_0592)	Metabolite Repair
NADHHS	NADH hydratase	(VBICanLib130745_0592)	Metabolite Repair
NADPHHR	NADPH hydratase	(VBICanLib130745_0592)	Metabolite Repair
NADPHHS	NADPH hydratase	(VBICanLib130745_0592)	Metabolite Repair
O16A4COLIPAabctex	O16 antigen (x4) core oligosaccharide lipid A transport via ABC system (periplasm to extracellular)	(VBICanLib130745_0499 and VBICanLib130745_0500 and VBICanLib130745_0501)	Lipopolysaccharide Biosynthesis / Recycling
OCTDPS	Octaprenyl pyrophosphate synthase	(VBICanLib130745_0490)	Cofactor and Prosthetic Group Biosynthesis

OMBZLM	2-Octaprenyl-6-methoxy-benzoquinol methylase	(VBICanLib130745_0490)	Cofactor and Prosthetic Group Biosynthesis
PA140abcpp	Phosphatidate transport via ABC system (n-C14:0, periplasm)	(VBICanLib130745_0850)	Transport, Inner Membrane
PE141abcpp	Phosphatidylethanolamine transport via ABC system (n-C14:1, periplasm)	(VBICanLib130745_0850)	Transport, Inner Membrane
RNDR3b	Ribonucleoside-diphosphate reductase (CDP) (glutaredoxin)	(VBICanLib130745_0749 and VBICanLib130745_0016) or (VBICanLib130745_0074) or (VBICanLib130745_0075) or (VBICanLib130745_0775) or (VBICanLib130745_0906) or (VBICanLib130745_0956)	Nucleotide Salvage Pathway
RNDR4b	Ribonucleoside-diphosphate reductase (UDP) (glutaredoxin)	(VBICanLib130745_0477)	Nucleotide Salvage Pathway
S2FE2SR	SUF [2Fe-2S] regeneration	(VBICanLib130745_0945 and VBICanLib130745_1012 and VBICanLib130745_1011 and VBICanLib130745_1010)	Cofactor and Prosthetic Group Biosynthesis
S2FE2SS	SUF [2Fe-2S] Synthesis	(VBICanLib130745_0945 and VBICanLib130745_1012 and VBICanLib130745_1011 and VBICanLib130745_1010)	Cofactor and Prosthetic Group Biosynthesis
S2FE2SS2	SUF [2Fe-2S] Synthesis II	(VBICanLib130745_0945 and VBICanLib130745_1012 and VBICanLib130745_1011 and VBICanLib130745_1010)	Cofactor and Prosthetic Group Biosynthesis
S2FE2ST	SUF [2Fe-2S] Transfer	(VBICanLib130745_0945 and VBICanLib130745_1012 and VBICanLib130745_1011 and VBICanLib130745_1010)	Cofactor and Prosthetic Group Biosynthesis
S4FE4SR	SUF [4Fe-4S] Reduction	(VBICanLib130745_0945 and VBICanLib130745_1012 and VBICanLib130745_1011 and VBICanLib130745_1010)	Cofactor and Prosthetic Group Biosynthesis

S4FE4ST	SUF [4Fe-4S] Transfer	(VBICanLib130745_0945 and VBICanLib130745_1012 and VBICanLib130745_1011 and VBICanLib130745_1010)	Cofactor and Prosthetic Group Biosynthesis
SCYSDS	SUF Cysteine desulfuration	(VBICanLib130745_1012)	Cofactor and Prosthetic Group Biosynthesis
FEex	Iron transporter	(VBICanLib130745_0850 and VBICanLib130745_0673) or (VBICanLib130745_0850)	iron metabolism
FEpp	Iron transporter	(VBICanLib130745_0673 and VBICanLib130745_0850)	iron metabolism
Kt3pp	Potassium transport out via proton antiport (periplasm)	(VBICanLib130745_0857)	Inorganic Ion Transport and Metabolism
2DHGLCNkt_tpp	2DHGLCNkt tpp	(RH08_03650)	Transport
3OAR141	3-oxoacyl-[acyl-carrier-protein] reductase (n-C14:1)	(RH08_03350) or (RH08_01075) or (RH08_01695)	Fatty Acid Biosynthesis
3OAS120	3-oxoacyl-[acyl-carrier-protein] synthase (n-C12:0)	(RH08_01690 and RH08_03360)	Fatty Acid Biosynthesis
4HTHRS	4-Hydroxy-L-threonine synthase	(RH08_01710)	Vitamin B6 Metabolism
ACCOAC	Acetyl-CoA carboxylase	(RH08_00715 and RH08_01930 and RH08_03100 and RH08_00720)	Fatty Acid Biosynthesis
ACGS	N-acetylglutamate synthase	(RH08_01810) or (RH08_01020)	Urea cycle/amino group metabolism
ACHBS	2-aceto-2-hydroxybutanoate synthase	(RH08_00090)	Valine, Leucine, and Isoleucine Metabolism
ACOATA	Acetyl-CoA ACP transacylase	(RH08_03530))	Fatty Acid Biosynthesis
ACONT	Aconitate hydratase	(RH08_01630)	TCA Cycle
ACONTa	Aconitase (half-reaction A, Citrate hydro-lyase)	((RH08_01630)	TCA Cycle
ACONTb	Aconitase (half-reaction B, Isocitrate hydro-lyase)	(RH08_01630))	TCA Cycle
ADSL1r	Adenylsuccinate lyase	(RH08_04320)	Purine Metabolism
ADSL2r	Adenylosuccinate lyase	(RH08_04320) or (RH08_00340) or (RH08_01235)	Purine Metabolism
AGPAT120	1-tetradecanoyl-sn-glycerol 3-phosphate O-acyltransferase (n-C12:0)	(RH08_03135)	Glycerophospholipid Metabolism
AGPAT160	1-hexadecanoyl-sn-glycerol 3-phosphate O-acyltransferase (n-C16:0)	(RH08_03135)	Glycerophospholipid Metabolism

AGPAT161	1-hexadec-7-enoyl-sn-glycerol 3-phosphate O-acyltransferase (n-C16:1)	(RH08_03135)	Glycerophospholipid Metabolism
AGPAT180	1-octadecanoyl-sn-glycerol 3-phosphate O-acyltransferase (n-C18:0)	(RH08_03135)	Glycerophospholipid Metabolism
AGPAT181	1-octadec-7-enoyl-sn-glycerol 3-phosphate O-acyltransferase (n-C18:1)	(RH08_03135)	Glycerophospholipid Metabolism
AICART	Phosphoribosylaminoimidazolecarboxamide formyltransferase	(RH08_02700)	Purine Metabolism
AKGDH	2-Oxogluterate dehydrogenase	(RH08_03355 and RH08_03345)	TCA Cycle
ALAALAr	D-alanine-D-alanine ligase (reversible)	(RH08_05265)	Peptidoglycan Biosynthesis
ALATRS	Alanyl-tRNA synthetase	(RH08_04695 and RH08_04685 and RH08_04690)	Alanine and Aspartate Metabolism
ANPRT	Anthranilate phosphoribosyltransferase	(RH08_01450)	Phenylalanine Tyrosine Tryptophan Biosynthesis
ANS	Anthranilate synthase	(RH08_01450)	Phenylalanine Tyrosine Tryptophan Biosynthesis
ARGabcpp	L-arginine transport via ABC system (periplasm)	(RH08_01320)	Transport
ASAD	Aspartate-semialdehyde dehydrogenase	(RH08_00915)	Lysine Biosynthesis
ASNS1	Asparagine synthase (glutamine-hydrolysing)	(RH08_01070)	Alanine and Aspartate Metabolism
ASPabcpp	L-aspartate transport via ABC system (periplasm)	(RH08_00245 and RH08_00240 and RH08_04980) or (RH08_04985)	Transport
ASPt2_2pp	Aspartate transport via proton symport (2 H) (periplasm)	(RH08_01320) or (RH08_05225)	Transport
ATPPRT	ATP phosphoribosyltransferase	(RH08_01710)	Histidine Metabolism
ATPS4rpp	ATP synthase (four protons for one ATP) (periplasm)	(RH08_02940 and RH08_02955 and RH08_04855 and RH08_02945 and RH08_02935 and RH08_02950)	Oxidative Phosphorylation
BZt1pp	Benzoate transport in via proton symport (periplasm)	(RH08_01320)	B-Ketoadipate pathway
CHLabcpp	Choline transport via ABC system (periplasm)	(RH08_01095 and RH08_01090) or (RH08_02520) or (RH08_03060) or (RH08_03235)	Transport
CHOLS_ex	CHOLS LPAREN ex RPAREN	(RH08_01710)	Transport
DAPE	Diaminopimelate epimerase	(RH08_02485)	Lysine Biosynthesis

CHOLSabc	CHOLSabc	(RH08_01100 and RH08_01095 and RH08_01090) or (RH08_02520) or (RH08_03060) or (RH08_03235)	Glycine, Serine and threonine metabolism
CITtex	Citrate transport via diffusion (extracellular to periplasm)	(RH08_04000)	Transport
COBALT2tex	Cobalt (Co+2) transport via diffusion (extracellular to periplasm)	(RH08_03055)	Transport
COBALT2tpp	Cobalt transport in via permease (no H+)	(RH08_03055)	Transport
CRNabcpp	L-carnitine transport via ABC system (periplasm)	(RH08_01090 and RH08_03060 and RH08_01095)	Transport
CS	Citrate synthase	((RH08_01090) and (RH08_01095)	TCA Cycle
CTPS2	CTP synthase (glutamine)	((RH08_01090) and (RH08_01095)	Pyrimidine Metabolism
CYO1b	CYO1b	(RH08_01755 and RH08_01760)	Oxidative Phosphorylation
CYSabcpp	L-cysteine uptake via ABC system (periplasm)	(RH08_03530))	Transport
DAPDC	Diaminopimelate decarboxylase	(RH08_01240)	Lysine Biosynthesis
DASYN120	CDP-diacylglycerol synthetase (n-C12:0)	(RH08_02160)	Glycerophospholipid Metabolism
DASYN160	CDP-diacylglycerol synthetase (n-C16:0)	(RH08_02160)	Glycerophospholipid Metabolism
DASYN161	CDP-diacylglycerol synthetase (n-C16:1)	(RH08_02160)	Glycerophospholipid Metabolism
DASYN180	CDP-diacylglycerol synthetase (n-C18:0)	(RH08_02160)	Glycerophospholipid Metabolism
DASYN181	CDP-diacylglycerol synthetase (n-C18:1)	(RH08_02160)	Glycerophospholipid Metabolism
DB4PS	3,4-Dihydroxy-2-butanone-4-phosphate synthase	(RH08_01180)	Riboflavin Metabolism
DDPA	3-deoxy-D-arabino-heptulosonate 7-phosphate synthetase	(RH08_02685)	Phenylalanine Tyrosine Tryptophan Biosynthesis
DHDPS	Dihydrodipicolinate synthase	(RH08_01090 and RH08_01095)	Lysine Biosynthesis
DHPM1	Dihydropyrimidinase (5,6-dihydrouracil)	(RH08_00890)	Pantothenate and CoA Biosynthesis
DMATT	Dimethylallyltranstransferase	(RH08_04975) or (RH08_02275)	Steroid biosynthesis
DNAMTn2r	DNA (cytosine-5-)-methyltransferase	(RH08_03445) or (RH08_03440)	Methionine Metabolism

DNTPPA	Dihydroneopterin triphosphate pyrophosphatase	(RH08_01015)	Folate Biosynthesis
DPCOAK	Dephospho-CoA kinase	(RH08_01935)	Pantothenate and CoA Biosynthesis
DPR	2-dehydropantoate 2-reductase	(RH08_04910)	Pantothenate and CoA Biosynthesis
DTMPK	DTMP kinase	(RH08_00475)	Pyrimidine Metabolism
DURAD	Dihydrouracil dehydrogenase (NADP)	(RH08_01550)	Pantothenate and CoA Biosynthesis
DUTPDP	DUTP diphosphatase	(RH08_04565)	Pyrimidine Metabolism
DXPS	1-deoxy-D-xylulose 5-phosphate synthase	(RH08_02665)	Steroid biosynthesis
E4PD	Erythrose 4-phosphate dehydrogenase	(RH08_02745)	Vitamin B6 Metabolism
EAR100y	Enoyl-[acyl-carrier-protein] reductase (NADPH) (n-C10:0)	(RH08_01695) or (RH08_05205)	Fatty Acid Biosynthesis
EAR120y	Enoyl-[acyl-carrier-protein] reductase (NADPH) (n-C12:0)	((RH08_01090) and (RH08_01095)	Fatty Acid Biosynthesis
EAR121y	Enoyl-[acyl-carrier-protein] reductase (NADPH) (n-C12:1)	(RH08_01090) and (RH08_01095))	Fatty Acid Biosynthesis
EAR160y	Enoyl-[acyl-carrier-protein] reductase (NADPH) (n-C16:0)	((RH08_01090) and (RH08_01095))	Fatty Acid Biosynthesis
EAR161y	Enoyl-[acyl-carrier-protein] reductase (NADPH) (n-C16:1)	(RH08_00250))	Fatty Acid Biosynthesis
EAR180y	Enoyl-[acyl-carrier-protein] reductase (NADPH) (n-C18:0)	(RH08_01695) or (RH08_05205)	Fatty Acid Biosynthesis
EAR181y	Enoyl-[acyl-carrier-protein] reductase (NADPH) (n-C18:1)	(RH08_01695) or (RH08_05205)	Fatty Acid Biosynthesis
EAR40y	Enoyl-[acyl-carrier-protein] reductase (NADPH) (n-C4:0)	(RH08_01695) or (RH08_05205)	Fatty Acid Biosynthesis
EAR60y	Enoyl-[acyl-carrier-protein] reductase (NADPH) (n-C6:0)	(RH08_01695) or (RH08_05205)	Fatty Acid Biosynthesis
EAR80y	Enoyl-[acyl-carrier-protein] reductase (NADPH) (n-C8:0)	(RH08_01760 and RH08_01755 and RH08_01745 and RH08_01750)	Fatty Acid Biosynthesis
ETHSO3abcpp	Ethanesulfonate transport via ABC system (periplasm)	(RH08_03060 and RH08_03055 and RH08_03735)	Transport
FDH	Formate dehydrogenase	(RH08_03735 and RH08_03730 and RH08_03725)	Methane Metabolism

FE2abc	Iron (II) transport via ABC system	(RH08_01090 and RH08_03230 and RH08_03215) or (RH08_03235 and RH08_03230 and RH08_03215)	Transport
FERtex	FERtex	(RH08_02330)	Transport
FERtpp	FERtpp	(RH08_02330)	Transport
FTHFD	Formyltetrahydrofolate deformylase	(RH08_04010)	One Carbon pool by folate
FUM	Fumarase	(RH08_00340) or (RH08_04320) or (RH08_01235)	TCA Cycle
FUMt2_2pp	Fumarate transport via proton symport (2 H) (periplasm)	(RH08_01320) or (RH08_05225)	Transport
FUMtex	Fumarate transport via diffusion (extracellular to periplasm)	(RH08_00340)	Transport
G1SAT	Glutamate-1-semialdehyde aminotransferase	(RH08_01540)	Porphyrin and Chlorophyll Metabolism
G3PAT120	Glycerol-3-phosphate acyltransferase (C12:0)	(RH08_03355)	Glycerophospholipid Metabolism
G3PAT160	Glycerol-3-phosphate acyltransferase (C16:0)	(RH08_03355)	Glycerophospholipid Metabolism
G3PAT161	Glycerol-3-phosphate acyltransferase (C16:1)	(RH08_03355)	Glycerophospholipid Metabolism
G3PAT180	Glycerol-3-phosphate acyltransferase (C18:0)	(RH08_03355)	Glycerophospholipid Metabolism
G3PAT181	Glycerol-3-phosphate acyltransferase (C18:1)	(RH08_03355)	Glycerophospholipid Metabolism
G6PDH2r	Glucose 6-phosphate dehydrogenase	(RH08_03500)	Glycolysis
GART	GAR transformylase-T	(RH08_00185)	Purine Metabolism
GF6PTA	Glutamine-fructose-6-phosphate transaminase	(RH08_04175) or (RH08_01070)	Glutamate Metabolism
GLCNt2rpp	D-gluconate transport via proton symport, reversible (periplasm)	(RH08_01140)	Transport
GLCabcpp	D-glucose transport via ABC system (periplasm)	(RH08_00250) or (RH08_01090) or (RH08_03235)	Transport
GLNS	Glutamine synthetase	(RH08_00320) or (RH08_03505)	Glutamate Metabolism
GLNTRAT	Glutamyl-tRNA(Gln) amidotransferase	(RH08_04335 and RH08_04345 and RH08_04340)	Glutamate Metabolism
GLUSy	Glutamate synthase (NADPH)	(RH08_00800)	Glutamate Metabolism

GLUabcpp	L-glutamate transport via ABC system (periplasm)	((RH08_00240 and RH08_04980 and RH08_00250 and RH08_00245)) or (RH08_00245) or (RH08_04985) or (RH08_00235)	Transport
GLUt4pp	Na+/glutamate symport (periplasm)	(RH08_04280)	Transport
GLXCL	Glyoxalate carboligase	(RH08_00865) or (RH08_02670)	Glycolate metabolism
GLYALDtex	Glyceraldehyde transport via diffusion (extracellular to periplasm)	(RH08_01880) or (RH08_00245)	Transport
GLYBabcpp	Glycine betaine transport via ABC system (periplasm)	((RH08_01090 and RH08_01095))	Arginine and Proline Metabolism
GLYCK	Glycerate kinase	(RH08_04040)	Glycine, Serine and threonine metabolism
GLYCL	Glycine Cleavage System	(RH08_02655) or (RH08_04695) or (RH08_04705) or (RH08_02655) or (RH08_04695) or (RH08_04705)	Glycine, Serine and threonine metabolism
GLYCLTt2rpp	Glycolate transport via proton symport, reversible (periplasm)	(RH08_02655) or (RH08_04695) or (RH08_04705) or (RH08_02655) or (RH08_04695) or (RH08_04705)	Transport
GLYCLTtex	Glycolate transport via diffusion (extracellular to periplasm)	(RH08_02655) or (RH08_04695) or (RH08_04705) or (RH08_02655) or (RH08_04695) or (RH08_04705)	Transport
GLYCL_2	Glycine cleavage system, cytosol	(RH08_02655) or (RH08_04695) or (RH08_04705) or (RH08_02655) or (RH08_04695) or (RH08_04705)	Nitrogen Metabolism
GLYCTO1	Glycolate oxidase	(RH08_02240)	Glycolate metabolism
GLYTRS	Glycyl-tRNA synthetase	(RH08_00250))	Glycine, Serine and threonine metabolism
GMPS2	GMP synthase	((RH08_00580) or (RH08_01330))	Purine Metabolism
GND	Phosphogluconate dehydrogenase	(RH08_02455)	Pentose Phosphate Pathway
GRTT	Geranyltranstransferase	(RH08_04975) or (RH08_02275)	Steroid biosynthesis
GTHPi	Glutathione peridoxase	(RH08_05440)	Glutathione Metabolism
GTHS	Glutathione synthetase	(RH08_02560)	Glutamate Metabolism
GTPCII	GTP cyclohydrolase II	(RH08_01180)	Riboflavin Metabolism
HCO3_NAt	Bicarbonate transport (Na/HCO3 cotransport)	(RH08_04080)	Transport
HEMEOS	Heme O synthase	(RH08_01185)	Porphyrin and Chlorophyll Metabolism
HISabcpp	L-histidine transport via ABC system (periplasm)	(RH08_01095)	Transport

HMBS	Hydroxymethylbilane synthase	(RH08_04620)	Porphyrin and Chlorophyll Metabolism
HMGL	Hydroxymethylglutaryl-CoA lyase	(RH08_00355)	Synthesis and degradation of Ketones Bodies
HPPK	2-amino-4-hydroxy-6-hydroxymethyldihydropteridine diphosphokinase	(RH08_05510)	Folate Biosynthesis
HXAtex	Hexanoate transport via diffusion (extracellular to periplasm)	(RH08_04080)	Transport
ICDHyr	Isocitrate dehydrogenase (NADP)	(RH08_03970)	TCA Cycle
ICITtex	Citrate transport via diffusion extracellular to periplasm	(RH08_04285)	Transport
ICL	Isocitrate lyase	(RH08_01780)	TCA Cycle
IGPS	Indole-3-glycerol-phosphate synthase	(RH08_00090)	Phenylalanine Tyrosine Tryptophan Biosynthesis
ILEabcpp	L-isoleucine transport via ABC system (periplasm)	(RH08_04285)	Transport
IPDDI	Isopentenyl-diphosphate D-isomerase	(RH08_04790)	Steroid biosynthesis
IPDPS	1-hydroxy-2-methyl-2-(E)-butenyl 4-diphosphate reductase (ipdp)	(RH08_04585)	Steroid biosynthesis
IPMD	3-isopropylmalate dehydrogenase	(RH08_03970)	Valine, Leucine, and Isoleucine Metabolism
IPPMIa	3-isopropylmalate dehydratase	(RH08_01630) or (RH08_03515)	Valine, Leucine, and Isoleucine Metabolism
IPPMIb	2-isopropylmalate hydratase	(RH08_01630) or (RH08_03515)	Valine, Leucine, and Isoleucine Metabolism
KARA1	Ketol-acid reductoisomerase (2,3-dihydroxy-3-methylbutanoate)	(RH08_05500)	Valine, Leucine, and Isoleucine Metabolism
KARA2	Ketol-acid reductoisomerase (2-Acetolactate)	(RH08_05500)	Valine, Leucine, and Isoleucine Metabolism
KAS14	Beta-ketoacyl-ACP synthase	(RH08_01690)	Fatty Acid Biosynthesis
LEUTRS	Leucyl-tRNA synthetase	(RH08_02020)	Valine, Leucine, and Isoleucine Metabolism
LEUabcpp	L-leucine transport via ABC system (periplasm)	(RH08_04285 and RH08_05165)	Transport
LYSTRS	Lysyl-tRNA synthetase	(RH08_05010)	Lysine Biosynthesis
LYSabcpp	L-lysine transport via ABC system (periplasm)	(RH08_05390)	Lysine Metabolism
MACPD	Malonyl-ACP decarboxylase	(RH08_00250)	Fatty Acid Biosynthesis
MALt2_2pp	Malate transport via proton symport (2 H) (periplasm)	(RH08_01320)	Transport
MSO3abcpp	Methanesulfonate transport via ABC system (periplasm)	(RH08_03055)	Transport

MTHFC	Methenyltetrahydrofolate cyclohydrolase	(RH08_03490)	One Carbon pool by folate
MTHFD	Methylenetetrahydrofolate dehydrogenase (NADP)	(RH08_03490)	One Carbon pool by folate
MHPGLUT	5 methyltetrahydropteroyltriglutamate homocysteine S methyltransferase	(RH08_04830)	Methionine Metabolism
NADH16pp	NADH dehydrogenase (ubiquinone-8 & 3 protons) (periplasm)	(RH08_03705 and RH08_03710 and RH08_03715 and RH08_03720 and RH08_03725 and RH08_03730 and RH08_03735 and RH08_03740 and RH08_03745 and RH08_03750 and RH08_03755 and RH08_03760 and RH08_03765 and RH08_03770)	Oxidative Phosphorylation
NADK	NAD kinase	(RH08_02280)	Nicotinate and Nicotinamide
NDPK1	Nucleoside-diphosphate kinase (ATP:GDP)	(RH08_01330)	Purine Metabolism
NDPK2	Nucleoside-diphosphate kinase (ATP:UDP)	(RH08_01330)	Pyrimidine Metabolism
NDPK3	Nucleoside-diphosphate kinase (ATP:CDP)	(RH08_01330)	Pyrimidine Metabolism
NDPK4	Nucleoside-diphosphate kinase (ATP:dTDP)	(RH08_01330)	Pyrimidine Metabolism
NDPK5	Nucleoside-diphosphate kinase (ATP:dGDP)	(RH08_01330)	Purine Metabolism
NDPK6	Nucleoside-diphosphate kinase (ATP:dUDP)	(RH08_01330)	Pyrimidine Metabolism
NDPK7	Nucleoside-diphosphate kinase (ATP:dCDP)	(RH08_01330)	Pyrimidine Metabolism
NDPK8	Nucleoside-diphosphate kinase (ATP:dADP)	(RH08_01330)	Purine Metabolism
NNATr	Nicotinate-nucleotide adenylyltransferase	(RH08_02770)	Nicotinate and Nicotinamide
OIVD1r	2-oxoisovalerate dehydrogenase (acylating; 4-methyl-2-oxopentaoate)	(RH08_04685)	Valine, leucine and isoleucine degradation
OIVD2	2-oxoisovalerate dehydrogenase (acylating; 3-methyl-2-oxobutanoate)	(RH08_04685)	Valine, leucine and isoleucine degradation
OIVD3	2-oxoisovalerate dehydrogenase (acylating; 3-methyl-2-oxopentanoate)	(RH08_04685)	Valine, leucine and isoleucine degradation

ORNCD	Ornithine cyclodeaminase	(RH08_05465) or (RH08_04635)	Arginine and Proline Metabolism
ORNabcpp	Ornithine transport via ABC system (periplasm)	(RH08_00250)	Lysine Metabolism
ORPT	Orotate phosphoribosyltransferase	(RH08_04500)	Pyrimidine Metabolism
PANTS	Pantothenate synthase	(RH08_02910)	Pantothenate and CoA Biosynthesis
PAPPT3	Phospho-N-acetylmuramoyl-pentapeptide-transferase (meso-2,6-diaminopimelate)	(RH08_04915)	Peptidoglycan Biosynthesis
PDH	Pyruvate dehydrogenase	(RH08_02655)	Glycolysis
PDX5POi	Pyridoxine 5-phosphate oxidase	(RH08_04290)	Vitamin B6 Metabolism
PGM	Phosphoglycerate mutase	(RH08_00775)	Gluconeogenesis
PGMT	Phosphoglucomutase	(RH08_04950)	Glycolysis
PMDPHT	Pyrimidine phosphatase	(RH08_00220)	Riboflavin Metabolism
PNTK	Pantothenate kinase	(RH08_01890)	Pantothenate and CoA Biosynthesis
PPA	Inorganic diphosphatase	(RH08_00540)	Oxidative Phosphorylation
PPBNGS	Porphobilinogen synthase	(RH08_02520)	Porphyrin and Chlorophyll Metabolism
PPPGO	Protoporphyrinogen oxidase (aerobic)	(RH08_04780)	Porphyrin and Chlorophyll Metabolism
PRAGSr	Phosphoribosylglycinamide synthase	((RH08_00540)	Purine Metabolism
PRAIi	Phosphoribosylanthranilate isomerase (irreversible)	(RH08_00090)	Phenylalanine Tyrosine Tryptophan Biosynthesis
PRAIS	Phosphoribosylaminoimidazole synthase	(RH08_04315)	Purine Metabolism
PRFGS	Phosphoribosylformylglycinamidine synthase	(RH08_04300)	Purine Metabolism
PROabcpp	L-proline transport via ABC system (periplasm)	(RH08_00250)	Arginine and Proline Metabolism
PRPPS	Phosphoribosylpyrophosphate synthetase	(RH08_01420)	Ribose Metabolism
PSP_L	Phosphoserine phosphatase (L-serine)	(RH08_04255)	Glycine, Serine and threonine metabolism
PTRCabcpp	Putrescine transport via ABC system (periplasm)	(RH08_03060) or (RH08_03795) or (RH08_01090)	Transport
RIBabcpp	D-ribose transport via ABC system (periplasm)	(RH08_03060) or (RH08_03795)	Transport
RPE	Ribulose 5-phosphate 3-epimerase	(RH08_04290)	Pentose Phosphate Pathway
RPI	Ribose-5-phosphate isomerase	(RH08_04700)	Pentose Phosphate Pathway
SDPTA	Succinyldiaminopimelate transaminase	(RH08_02145))	Lysine Biosynthesis

SERAT	Serine O-acetyltransferase	(RH08_02620)	Cysteine Metabolism
SERTRS	Seryl-tRNA synthetase	(RH08_02630)	Glycine, Serine and threonine metabolism
SHCHF	Sirohydrochlorin ferrochelatase	(RH08_02970)	Porphyrin and Chlorophyll Metabolism
SOTA	Succinylornithine transaminase	(RH08_01540)	Arginine and Proline Metabolism
SPMDabcpp	Spermidine transport via ABC system (periplasm)	(RH08_01380)	Transport
SUCD1	Succinate dehydrogenase	(RH08_04650 and RH08_04655 and RH08_04645)	
SUCDi	Succinate dehydrogenase (irreversible)	(RH08_04650 and RH08_04655 and RH08_04645)	
SUCOAS	Succinyl-CoA synthetase (ADP-forming)	(RH08_04675)	TCA Cycle
SULabcpp	Sulfate transport via ABC system (periplasm)	(RH08_03930)	Sulfur Metabolism
TRSARr	Tartronate semialdehyde reductase	(RH08_02455)	Glycolate metabolism
TSULabcpp	Thiosulfate transport via ABC system (periplasm)	(RH08_03235) or (RH08_01090)	Sulfur Metabolism
UAMAS	UDP-N-acetylmuramoyl-L-alanine synthetase	(RH08_04895)	Glutamate metabolism
UMPK	UMP kinase	(RH08_02145)	Pyrimidine Metabolism
UPPN	B-ureidopropionase	(RH08_04900)	Pantothenate and CoA Biosynthesis
UREA	Urease	(RH08_03885)	Urea cycle/amino group metabolism
VANKpp	VANKpp	(RH08_04285)	Transport
VANLNtex	VANLNtex	(RH08_04285)	Transport
VANLTtex	VANLTtex	(RH08_04285)	Transport
VNLNpp	VNLNpp	(RH08_04285)	Transport
pentso3abcpp	Pentso3abcpp	(RH08_03060)	Transport
ACOLIPAabctex	Arabinose modified core oligosaccharide lipid A transport via ABC system (periplasm to extracellular)	(RH08_02330 and RH08_01345) or (RH08_03335) or (RH08_03235) or (RH08_00085) or (RH08_02520)	Lipopolysaccharide Biosynthesis / Recycling
ALATA_D2	D-alanine transaminase	(RH08_01135 or RH08_04735)	Cofactor and Prosthetic Group Biosynthesis
ALATA_L2	Alanine transaminase	(RH08_01135 or RH08_04735)	Cofactor and Prosthetic Group Biosynthesis
AMPTASECG	Alanyl aminopeptidase (cys-gly)	(RH08_01340)	Cysteine Metabolism
AMPTASEPG	Aminopeptidase (pro-gly)	(RH08_04870 or RH08_04735)	Arginine and Proline Metabolism

ASCBptspp	L-ascorbate transport via PEP:Pyr PTS (periplasm)	(RH08_00245 and RH08_00240 and RH08_04980 and RH08_04980) or (RH08_05215)	Transport, Inner Membrane
BUTSO3abcpp	Butanesulfonate transport via ABC system (periplasm)	(RH08_03060 and RH08_03055 and RH08_01095)	Transport, Inner Membrane
CLIPAabctex	Cold lipid A transport via ABC system (periplasm to extracellular)	(RH08_02405 and RH08_01345 and RH08_02330 and RH08_02325 and RH08_01355 and RH08_01605)	Lipopolysaccharide Biosynthesis / Recycling
COLIPAPabctex	Core oligosaccharide lipid A diphosphate transport via ABC system (periplasm to extracellular)	(RH08_01345 and RH08_02325 and RH08_02330)	Lipopolysaccharide Biosynthesis / Recycling
COLIPAabctex	Core oligosaccharide lipid A transport via ABC system (periplasm to extracellular)	(RH08_01345 and RH08_02325 and RH08_02330)	Lipopolysaccharide Biosynthesis / Recycling
CRNDabcpp	D-carnitine transport via ABC system (periplasm)	(RH08_01095 and RH08_01090) or (RH08_02520) or (RH08_03060) or (RH08_03235)	Transport, Inner Membrane
CYSSADS	L-cysteine sulfinic acid desulfurase	(RH08_04870 or RH08_04735)	Cysteine Metabolism
ECA4COLIPAabctex	Enterobacterial common antigen (x4) core oligosaccharide lipid A transport via ABC system (periplasm to extracellular)	(RH08_02325 and RH08_02405 and RH08_02330 and RH08_01345)	Lipopolysaccharide Biosynthesis / Recycling
ENLIPAabctex	Phosphoethanolamine lipid A transport via ABC system (periplasm to extracellular)	(RH08_02330 and RH08_01345 and RH08_02325)	Lipopolysaccharide Biosynthesis / Recycling
GRXR	Glutaredoxin reductase	(RH08_04290)	Unassigned
K2L4Aabctex	KDO(2)-lipid IV A transport via ABC system (periplasm to extracellular)	(RH08_04040)	Lipopolysaccharide Biosynthesis / Recycling
KAS15	Beta-ketoacyl-ACP synthase (2)	(RH08_03360)	Membrane Lipid Metabolism
KDOPS	3-deoxy -D-manno-octulosonic -acid 8-phosphate synthase	(RH08_02685)	Lipopolysaccharide Biosynthesis / Recycling
LIPAabctex	Lipid A transport via ABC system (periplasm to extracellular)	(RH08_04040)	Lipopolysaccharide Biosynthesis / Recycling

NADH17pp	NADH dehydrogenase (menaquinone-8 & 3 protons) (periplasm)	(RH08_03705 and RH08_03710 and RH08_03715 and RH08_03720 and RH08_03725 and RH08_03730 and RH08_03735 and RH08_03740 and RH08_03745 and RH08_03750 and RH08_03755 and RH08_03760 and RH08_03765 and RH08_03770)	Oxidative Phosphorylation
NADH18pp	NADH dehydrogenase (demethylmenaquinone-8 & 3 protons) (periplasm)	(RH08_03705 and RH08_03710 and RH08_03715 and RH08_03720 and RH08_03725 and RH08_03730 and RH08_03735 and RH08_03740 and RH08_03745 and RH08_03750 and RH08_03755 and RH08_03760 and RH08_03765 and RH08_03770)	Oxidative Phosphorylation
NADHHR	NADH hydratase	(RH08_02745)	Metabolite Repair
NADHHS	NADH hydratase	(RH08_02745)	Metabolite Repair
NADPHHR	NADPH hydratase	(RH08_02745)	Metabolite Repair
NADPHHS	NADPH hydratase	(RH08_02745)	Metabolite Repair
O16A4COLIPAabctex	O16 antigen (x4) core oligosaccharide lipid A transport via ABC system (periplasm to extracellular)	(RH08_02320 and RH08_02325 and RH08_02330)	Lipopolysaccharide Biosynthesis / Recycling
OCTDPS	Octaprenyl pyrophosphate synthase	(RH08_01330))	Cofactor and Prosthetic Group Biosynthesis
OMBZLM	2-Octaprenyl-6-methoxy-benzoquinol methylase	(RH08_01330))	Cofactor and Prosthetic Group Biosynthesis
RNDR3b	Ribonucleoside-diphosphate reductase (CDP) (glutaredoxin)	(RH08_03510 and RH08_03665) or (RH08_04275)	Nucleotide Salvage Pathway
RNDR4b	Ribonucleoside-diphosphate reductase (UDP) (glutaredoxin)	(RH08_02210)	Nucleotide Salvage Pathway
S2FE2SR	SUF [2Fe-2S] regeneration	(RH08_04430 and RH08_04735 and RH08_04730 and RH08_04725)	Cofactor and Prosthetic Group Biosynthesis
S2FE2SS	SUF [2Fe-2S] Synthesis	(RH08_04430 and RH08_04735 and RH08_04730 and RH08_04725)	Cofactor and Prosthetic Group Biosynthesis
S2FE2SS2	SUF [2Fe-2S] Synthesis II	(RH08_04430 and RH08_04735 and RH08_04730 and RH08_04725)	Cofactor and Prosthetic Group Biosynthesis
S2FE2ST	SUF [2Fe-2S] Transfer	(RH08_04430 and RH08_04735 and RH08_04730 and RH08_04725)	Cofactor and Prosthetic Group Biosynthesis
S4FE4SR	SUF [4Fe-4S] Reduction	(RH08_04430 and RH08_04735 and RH08_04730 and RH08_04725)	Cofactor and Prosthetic Group Biosynthesis

S4FE4ST	SUF [4Fe-4S] Transfer	(RH08_04430 and RH08_04735 and RH08_04730 and RH08_04725)	Cofactor and Prosthetic Group Biosynthesis
SCYSDS	SUF Cysteine desulfuration	(RH08_04735)	Cofactor and Prosthetic Group Biosynthesis
FEex	Iron transporter	((RH08_04040 and RH08_03155) or (RH08_00355)	iron metabolism
FEpp	Iron transporter	(RH08_03155 and RH08_04040)	iron metabolism
Kt3pp	Potassium transport out via proton antiport (periplasm)	(RH08_04075)	Inorganic Ion Transport and Metabolism
2DHGLCNkt_tpp	2DHGLCNkt tpp	(VBICanLib238577_0746)	Transport
3OAR141	3-oxoacyl-[acyl-carrier-protein] reductase (n-C14:1)	(VBICanLib238577_0692) or (VBICanLib238577_0223) or (VBICanLib238577_0358)	Fatty Acid Biosynthesis
3OAS120	3-oxoacyl-[acyl-carrier-protein] synthase (n-C12:0)	(VBICanLib238577_0357 and VBICanLib238577_0694)	Fatty Acid Biosynthesis
4HTHRS	4-Hydroxy-L-threonine synthase	(VBICanLib238577_0361)	Vitamin B6 Metabolism
ACCOAC	Acetyl-CoA carboxylase	(VBICanLib238577_0399 and VBICanLib238577_0145 and VBICanLib238577_0637 and VBICanLib238577_0146)	Fatty Acid Biosynthesis
ACGS	N-acetylglutamate synthase	(VBICanLib238577_0381) or (VBICanLib238577_0209)	Urea cycle/amino group metabolism
ACHBS	2-aceto-2-hydroxybutanoate synthase	(VBICanLib238577_0003)	Valine, Leucine, and Isoleucine Metabolism
ACOATA	Acetyl-CoA ACP transacylase	(VBICanLib238577_0727)	Fatty Acid Biosynthesis
ACONT	Aconitate hydratase	(VBICanLib238577_0345)	TCA Cycle
ACONTa	Aconitase (half-reaction A, Citrate hydro-lyase)	((VBICanLib238577_0345)	TCA Cycle
ACONTb	Aconitase (half-reaction B, Isocitrate hydro-lyase)	(VBICanLib238577_0345))	TCA Cycle
ADSL1r	Adenylsuccinate lyase	(VBICanLib238577_0886)	Purine Metabolism
ADSL2r	Adenylosuccinate lyase	(VBICanLib238577_0886) or (VBICanLib238577_0061) or (VBICanLib238577_0260)	Purine Metabolism
AGPAT120	1-tetradecanoyl-sn-glycerol 3-phosphate O-acyltransferase (n-C12:0)	(VBICanLib238577_0645)	Glycerophospholipid Metabolism
AGPAT160	1-hexadecanoyl-sn-glycerol 3-phosphate O-acyltransferase (n-C16:0)	(VBICanLib238577_0645)	Glycerophospholipid Metabolism

AGPAT161	1-hexadec-7-enoyl-sn-glycerol 3-phosphate O-acyltransferase (n-C16:1)	(VBICanLib238577_0645)	Glycerophospholipid Metabolism
AGPAT180	1-octadecanoyl-sn-glycerol 3-phosphate O-acyltransferase (n-C18:0)	(VBICanLib238577_0645)	Glycerophospholipid Metabolism
AGPAT181	1-octadec-7-enoyl-sn-glycerol 3-phosphate O-acyltransferase (n-C18:1)	(VBICanLib238577_0645)	Glycerophospholipid Metabolism
AICART	Phosphoribosylaminoimidazolecarboxamide formyltransferase	(VBICanLib238577_0559)	Purine Metabolism
AKGDH	2-Oxogluterate dehydrogenase	((VBICanLib238577_0693) and (VBICanLib238577_0691))	TCA Cycle
ALAALAr	D-alanine-D-alanine ligase (reversible)	(VBICanLib238577_1111)	Peptidoglycan Biosynthesis
ALATRS	Alanyl-tRNA synthetase	((VBICanLib238577_0975) and (VBICanLib238577_0973) and (VBICanLib238577_0974))	Alanine and Aspartate Metabolism
ANPRT	Anthranilate phosphoribosyltransferase	(VBICanLib238577_0305)	Phenylalanine Tyrosine Tryptophan Biosynthesis
ANS	Anthranilate synthase	(VBICanLib238577_0305)	Phenylalanine Tyrosine Tryptophan Biosynthesis
ARGSS	Argininosuccinate synthase	(VBICanLib238577_0110)	Arginine and Proline Metabolism
ARGabcpp	L-arginine transport via ABC system (periplasm)	(VBICanLib238577_0277)	Transport
ASAD	Aspartate-semialdehyde dehydrogenase	(VBICanLib238577_0189)	Lysine Biosynthesis
ASNS1	Asparagine synthase (glutamine-hydrolysing)	(VBICanLib238577_0222)	Alanine and Aspartate Metabolism
ASPabcpp	L-aspartate transport via ABC system (periplasm)	((VBICanLib238577_0041 and VBICanLib238577_0040 and VBICanLib238577_1037) or (VBICanLib238577_1038)	Transport
ASPt2_2pp	Aspartate transport via proton symport (2 H) (periplasm)	(VBICanLib238577_0277) or (VBICanLib238577_1103)	Transport
ATPPRT	ATP phosphoribosyltransferase	(VBICanLib238577_0361)	Histidine Metabolism
ATPS4rpp	ATP synthase (four protons for one ATP) (periplasm)	(VBICanLib238577_0608 and VBICanLib238577_0611 and VBICanLib238577_1012 and VBICanLib238577_0609 and VBICanLib238577_0607 and VBICanLib238577_0610)	Oxidative Phosphorylation

BZt1pp	Benzoate transport in via proton symport (periplasm)	(VBICanLib238577_0277)	B-Ketoadipate pathway
CHLabcpp	Choline transport via ABC system (periplasm)	(VBICanLib238577_0228 and VBICanLib238577_0227) or (VBICanLib238577_0520) or (VBICanLib238577_0629) or (VBICanLib238577_0667)	Transport
CHOLS_ex	CHOLS LPAREN ex RPAREN	(VBICanLib238577_0361)	Transport
DAPE	Diaminopimelate epimerase	(VBICanLib238577_0513)	Lysine Biosynthesis
CHOLSabc	CHOLSabc	(VBICanLib238577_02299) or (VBICanLib238577_0228 and VBICanLib238577_0227) or (VBICanLib238577_0520) or (VBICanLib238577_0629) or (VBICanLib238577_0667)	Glycine, Serine and threonine metabolism
CITtex	Citrate transport via diffusion (extracellular to periplasm)	(VBICanLib238577_0813)	Transport
COBALT2tex	Cobalt (Co+2) transport via diffusion (extracellular to periplasm)	(VBICanLib238577_0628)	Transport
COBALT2tpp	Cobalt transport in via permease (no H+)	(VBICanLib238577_0628)	Transport
CRNabcpp	L-carnitine transport via ABC system (periplasm)	(VBICanLib238577_0227 and VBICanLib238577_0629 and VBICanLib238577_0228)	Transport
CS	Citrate synthase	((VBICanLib238577_0227) and (VBICanLib238577_0228)	TCA Cycle
CTPS2	CTP synthase (glutamine)	((VBICanLib238577_0227) and (VBICanLib238577_0228)	Pyrimidine Metabolism
CYO1b	CYO1b	(VBICanLib238577_0370 and VBICanLib238577_0371)	Oxidative Phosphorylation
CYSabcpp	L-cysteine uptake via ABC system (periplasm)	((VBICanLib238577_0729) or (VBICanLib238577_0729))	Transport
DAPDC	Diaminopimelate decarboxylase	(VBICanLib238577_0261)	Lysine Biosynthesis
DASYN120	CDP-diacylglycerol synthetase (n-C12:0)	(VBICanLib238577_0447)	Glycerophospholipid Metabolism
DASYN160	CDP-diacylglycerol synthetase (n-C16:0)	(VBICanLib238577_0447)	Glycerophospholipid Metabolism

DASYN161	CDP-diacylglycerol synthetase (n-C16:1)	(VBICanLib238577_0447)	Glycerophospholipid Metabolism
DASYN180	CDP-diacylglycerol synthetase (n-C18:0)	(VBICanLib238577_0447)	Glycerophospholipid Metabolism
DASYN181	CDP-diacylglycerol synthetase (n-C18:1)	(VBICanLib238577_0447)	Glycerophospholipid Metabolism
DB4PS	3,4-Dihydroxy-2-butanone-4-phosphate synthase	(VBICanLib238577_0249)	Riboflavin Metabolism
DDPA	3-deoxy-D-arabino-heptulosonate 7-phosphate synthetase	(VBICanLib238577_0553)	Phenylalanine Tyrosine Tryptophan Biosynthesis
DHDPS	Dihydrodipicolinate synthase	(VBICanLib238577_0227 and VBICanLib238577_0228)	Lysine Biosynthesis
DHPM1	Dihydropyrimidinase (5,6-dihydrouracil)	(VBICanLib238577_0183)	Pantothenate and CoA Biosynthesis
DMATT	Dimethylallyltranstransferase	(VBICanLib238577_1036) or (VBICanLib238577_0469)	Steroid biosynthesis
DNAMTn2r	DNA (cytosine-5-)-methyltransferase	(VBICanLib238577_0710) or (VBICanLib238577_0709)	Methionine Metabolism
DNTPPA	Dihydroneopterin triphosphate pyrophosphatase	(VBICanLib238577_0208)	Folate Biosynthesis
DPCOAK	Dephospho-CoA kinase	(VBICanLib238577_0401)	Pantothenate and CoA Biosynthesis
DPR	2-dehydropantoate 2-reductase	(VBICanLib238577_1024)	Pantothenate and CoA Biosynthesis
DTMPK	DTMP kinase	(VBICanLib238577_0094)	Pyrimidine Metabolism
DURAD	Dihydrouracil dehydrogenase (NADP)	(VBICanLib238577_0327)	Pantothenate and CoA Biosynthesis
DUTPDP	DUTP diphosphatase	(VBICanLib238577_0949)	Pyrimidine Metabolism
DXPS	1-deoxy-D-xylulose 5-phosphate synthase	(VBICanLib238577_0548)	Steroid biosynthesis
E4PD	Erythrose 4-phosphate dehydrogenase	(VBICanLib238577_0569)	Vitamin B6 Metabolism
EAR100y	Enoyl-[acyl-carrier-protein] reductase (NADPH) (n-C10:0)	(VBICanLib238577_0358) or (VBICanLib238577_1098)	Fatty Acid Biosynthesis
EAR120y	Enoyl-[acyl-carrier-protein] reductase (NADPH) (n-C12:0)	(VBICanLib238577_1098) or (VBICanLib238577_0358) or (VBICanLib238577_0692)	Fatty Acid Biosynthesis
EAR121y	Enoyl-[acyl-carrier-protein] reductase (NADPH) (n-C12:1)	((VBICanLib238577_0228) and (VBICanLib238577_0227)	Fatty Acid Biosynthesis
EAR160y	Enoyl-[acyl-carrier-protein] reductase (NADPH) (n-C16:0)	(VBICanLib238577_0358) or (VBICanLib238577_1098)	Fatty Acid Biosynthesis

EAR161y	Enoyl-[acyl-carrier-protein] reductase (NADPH) (n-C16:1)	((VBICanLib238577_0227) and (VBICanLib238577_0228))	Fatty Acid Biosynthesis
EAR180y	Enoyl-[acyl-carrier-protein] reductase (NADPH) (n-C18:0)	(VBICanLib238577_0358) or (VBICanLib238577_1098)	Fatty Acid Biosynthesis
EAR181y	Enoyl-[acyl-carrier-protein] reductase (NADPH) (n-C18:1)	(VBICanLib238577_0358) or (VBICanLib238577_1098)	Fatty Acid Biosynthesis
EAR40y	Enoyl-[acyl-carrier-protein] reductase (NADPH) (n-C4:0)	(VBICanLib238577_0358) or (VBICanLib238577_1098)	Fatty Acid Biosynthesis
EAR60y	Enoyl-[acyl-carrier-protein] reductase (NADPH) (n-C6:0)	(VBICanLib238577_0692)	Fatty Acid Biosynthesis
EAR80y	Enoyl-[acyl-carrier-protein] reductase (NADPH) (n-C8:0)	(VBICanLib238577_0371 and VBICanLib238577_0370 and VBICanLib238577_0368 and VBICanLib238577_0369)	Fatty Acid Biosynthesis
ETHSO3abcpp	Ethanesulfonate transport via ABC system (periplasm)	(VBICanLib238577_0629 and VBICanLib238577_0628 and VBICanLib238577_0760)	Transport
FDH	Formate dehydrogenase	(VBICanLib238577_0760 and VBICanLib238577_0759 and VBICanLib238577_0758)	Methane Metabolism
FE2abc	Iron (II) transport via ABC system	(VBICanLib238577_0227) or (VBICanLib238577_0667 and VBICanLib238577_0666 and VBICanLib238577_0663)	Transport
FERtex	FERtex	(VBICanLib238577_0481)	Transport
FERtpp	FERtpp	(VBICanLib238577_0481)	Transport
FTHFD	Formyltetrahydrofolate deformylase	(VBICanLib238577_0815)	One Carbon pool by folate
FUM	Fumarase	(VBICanLib238577_0061) or (VBICanLib238577_0886) or (VBICanLib238577_0260)	TCA Cycle
FUMt2_2pp	Fumarate transport via proton symport (2 H) (periplasm)	(VBICanLib238577_0277) or (VBICanLib238577_1103)	Transport
FUMtex	Fumarate transport via diffusion (extracellular to periplasm)	(VBICanLib238577_0061)	Transport
G1SAT	Glutamate-1-semialdehyde aminotransferase	(VBICanLib238577_0325) or (VBICanLib238577_0385)	Porphyrin and Chlorophyll Metabolism

G3PAT120	Glycerol-3-phosphate acyltransferase (C12:0)	(VBICanLib238577_0693)	Glycerophospholipid Metabolism
G3PAT160	Glycerol-3-phosphate acyltransferase (C16:0)	(VBICanLib238577_0693)	Glycerophospholipid Metabolism
G3PAT161	Glycerol-3-phosphate acyltransferase (C16:1)	(VBICanLib238577_0693)	Glycerophospholipid Metabolism
G3PAT180	Glycerol-3-phosphate acyltransferase (C18:0)	(VBICanLib238577_0693)	Glycerophospholipid Metabolism
G3PAT181	Glycerol-3-phosphate acyltransferase (C18:1)	(VBICanLib238577_0693)	Glycerophospholipid Metabolism
G6PDH2r	Glucose 6-phosphate dehydrogenase	(VBICanLib238577_0721)	Glycolysis
GART	GAR transformylase-T	(VBICanLib238577_0025)	Purine Metabolism
GF6PTA	Glutamine-fructose-6-phosphate transaminase	(VBICanLib238577_0850) or (VBICanLib238577_0222)	Glutamate Metabolism
GLCNt2rpp	D-gluconate transport via proton symport, reversible (periplasm)	(VBICanLib238577_0238)	Transport
GLCabcpp	D-glucose transport via ABC system (periplasm)	(VBICanLib238577_0042) or (VBICanLib238577_0227) or (VBICanLib238577_0667)	Transport
GLNS	Glutamine synthetase	(VBICanLib238577_0055) or (VBICanLib238577_0722)	Glutamate Metabolism
GLNTRAT	Glutamyl-tRNA(Gln) amidotransferase	(VBICanLib238577_0891 and VBICanLib238577_0893 and VBICanLib238577_0892)	Glutamate Metabolism
GLUSy	Glutamate synthase (NADPH)	(VBICanLib238577_0163)	Glutamate Metabolism
GLUabcpp	L-glutamate transport via ABC system (periplasm)	(VBICanLib238577_0040 and VBICanLib238577_1037 and VBICanLib238577_0042 and VBICanLib238577_0041) or (VBICanLib238577_0041) or (VBICanLib238577_1038) or (VBICanLib238577_0039)	Transport
GLUt4pp	Na+/glutamate symport (periplasm)	(VBICanLib238577_0877)	Transport
GLXCL	Glyoxalate carboligase	(VBICanLib238577_0177) or (VBICanLib238577_0549)	Glycolate metabolism

GLYALDtex	Glyceraldehyde transport via diffusion (extracellular to periplasm)	(VBICanLib238577_0389) or (VBICanLib238577_0041)	Transport
GLYBabcpp	Glycine betaine transport via ABC system (periplasm)	(VBICanLib238577_0227 and VBICanLib238577_0228)	Arginine and Proline Metabolism
GLYCK	Glycerate kinase	(VBICanLib238577_0822) or (VBICanLib238577_1138)	Glycine, Serine and threonine metabolism
GLYCL	Glycine Cleavage System	(VBICanLib238577_0546) or (VBICanLib238577_0975) or (VBICanLib238577_0977) or (VBICanLib238577_0546) or (VBICanLib238577_0975) or (VBICanLib238577_0977)	Glycine, Serine and threonine metabolism
GLYCLTt2rpp	Glycolate transport via proton symport, reversible (periplasm)	(VBICanLib238577_0546) or (VBICanLib238577_0975) or (VBICanLib238577_0977) or (VBICanLib238577_0546) or (VBICanLib238577_0975) or (VBICanLib238577_0977)	Transport
GLYCLTtex	Glycolate transport via diffusion (extracellular to periplasm)	(VBICanLib238577_0546) or (VBICanLib238577_0975) or (VBICanLib238577_0977) or (VBICanLib238577_0546) or (VBICanLib238577_0975) or (VBICanLib238577_0977)	Transport
GLYCL_2	Glycine cleavage system, cytosol	(VBICanLib238577_0546) or (VBICanLib238577_0975) or (VBICanLib238577_0977) or (VBICanLib238577_0546) or (VBICanLib238577_0975) or (VBICanLib238577_0977)	Nitrogen Metabolism
GLYCTO1	Glycolate oxidase	(VBICanLib238577_0463)	Glycolate metabolism
GLYTRS	Glycyl-tRNA synthetase	(VBICanLib238577_1019 and VBICanLib238577_0467)	Glycine, Serine and threonine metabolism
GMPS2	GMP synthase	(VBICanLib238577_0117) or (VBICanLib238577_0279)	Purine Metabolism
GND	Phosphogluconate dehydrogenase	(VBICanLib238577_0507) or (VBICanLib238577_0315)	Pentose Phosphate Pathway

GRTT	Geranyltranstransferase	(VBICanLib238577_1036) or (VBICanLib238577_0469)	Steroid biosynthesis
GTHPi	Glutathione peridoxase	(VBICanLib238577_1189)	Glutathione Metabolism
GTHS	Glutathione synthetase	(VBICanLib238577_0528)	Glutamate Metabolism
GTPCII	GTP cyclohydrolase II	(VBICanLib238577_0249)	Riboflavin Metabolism
HCO3_NAt	Bicarbonate transport (Na/HCO3 cotransport)	(VBICanLib238577_0830)	Transport
HEMEOS	Heme O synthase	(VBICanLib238577_0250)	Porphyrin and Chlorophyll Metabolism
HISabcpp	L-histidine transport via ABC system (periplasm)	(VBICanLib238577_0228 and VBICanLib238577_0932)	Transport
HMBS	Hydroxymethylbilane synthase	(VBICanLib238577_0959)	Porphyrin and Chlorophyll Metabolism
HMGL	Hydroxymethylglutaryl-CoA lyase	(VBICanLib238577_0065)	Synthesis and degradation of Ketones Bodies
HPPK	2-amino-4-hydroxy-6-hydroxymethyldihydropteridine diphosphokinase	(VBICanLib238577_1082)	Folate Biosynthesis
HXAtex	Hexanoate transport via diffusion (extracellular to periplasm)	(VBICanLib238577_0830)	Transport
ICDHyr	Isocitrate dehydrogenase (NADP)	(VBICanLib238577_0807)	TCA Cycle
ICITtex	Citrate transport via diffusion extracellular to periplasm	(VBICanLib238577_0879)	Transport
ICL	Isocitrate lyase	(VBICanLib238577_0375)	TCA Cycle
IGPS	Indole-3-glycerol-phosphate synthase	(VBICanLib238577_0003)	Phenylalanine Tyrosine Tryptophan Biosynthesis
ILEabcpp	L-isoleucine transport via ABC system (periplasm)	(VBICanLib238577_0879)	Transport
IPDPS	1-hydroxy-2-methyl-2-(E)-butenyl 4-diphosphate reductase (ipdp)	(VBICanLib238577_0952)	Steroid biosynthesis
IPMD	3-isopropylmalate dehydrogenase	(VBICanLib238577_0807)	Valine, Leucine, and Isoleucine Metabolism
IPPMIa	3-isopropylmalate dehydratase	(VBICanLib238577_0345) or (VBICanLib238577_0724)	Valine, Leucine, and Isoleucine Metabolism
IPPMIb	2-isopropylmalate hydratase	(VBICanLib238577_0345) or (VBICanLib238577_0724)	Valine, Leucine, and Isoleucine Metabolism
KARA1	Ketol-acid reductoisomerase (2,3-dihydroxy-3-methylbutanoate)	(VBICanLib238577_1203) or (VBICanLib238577_1163)	Valine, Leucine, and Isoleucine Metabolism
KARA2	Ketol-acid reductoisomerase (2-Acetolactate)	(VBICanLib238577_1203) or (VBICanLib238577_1163)	Valine, Leucine, and Isoleucine Metabolism
KAS14	Beta-ketoacyl-ACP synthase	(VBICanLib238577_0357)	Fatty Acid Biosynthesis

LEUTRS	Leucyl-tRNA synthetase	(VBICanLib238577_0418)	Valine, Leucine, and Isoleucine Metabolism
LEUabcpp	L-leucine transport via ABC system (periplasm)	(VBICanLib238577_0879)	Transport
LYSTRS	Lysyl-tRNA synthetase	(VBICanLib238577_1044)	Lysine Biosynthesis
LYSabcpp	L-lysine transport via ABC system (periplasm)	(VBICanLib238577_1180)	Lysine Metabolism
MACPD	Malonyl-ACP decarboxylase	(VBICanLib238577_0042)	Fatty Acid Biosynthesis
MALt2_2pp	Malate transport via proton symport (2 H) (periplasm)	(VBICanLib238577_0277)	Transport
MSO3abcpp	Methanesulfonate transport via ABC system (periplasm)	(VBICanLib238577_0628)	Transport
MTHFC	Methenyltetrahydrofolate cyclohydrolase	(VBICanLib238577_0719)	One Carbon pool by folate
MTHFD	Methylenetetrahydrofolate dehydrogenase (NADP)	(VBICanLib238577_0719)	One Carbon pool by folate
MHPGLUT	5 methyltetrahydropteroyltriglutamate homocysteine S methyltransferase	(VBICanLib238577_1007)	Methionine Metabolism
NADH16pp	NADH dehydrogenase (ubiquinone-8 & 3 protons) (periplasm)	(VBICanLib238577_0754 and VBICanLib238577_0755 and VBICanLib238577_0756 and VBICanLib238577_0757 and VBICanLib238577_0758 and VBICanLib238577_0759 and VBICanLib238577_0760 and VBICanLib238577_0761 and VBICanLib238577_0762 and VBICanLib238577_0763 and VBICanLib238577_0764 and VBICanLib238577_0765 and VBICanLib238577_0766 and VBICanLib238577_0767)	Oxidative Phosphorylation
NADK	NAD kinase	(VBICanLib238577_0470)	Nicotinate and Nicotinamide
NDPK1	Nucleoside-diphosphate kinase (ATP:GDP)	(VBICanLib238577_0279)	Purine Metabolism
NDPK2	Nucleoside-diphosphate kinase (ATP:UDP)	(VBICanLib238577_0279)	Pyrimidine Metabolism

NDPK3	Nucleoside-diphosphate kinase (ATP:CDP)	(VBICanLib238577_0279)	Pyrimidine Metabolism
NDPK4	Nucleoside-diphosphate kinase (ATP:dTDP)	(VBICanLib238577_0279)	Pyrimidine Metabolism
NDPK5	Nucleoside-diphosphate kinase (ATP:dGDP)	(VBICanLib238577_0279)	Purine Metabolism
NDPK6	Nucleoside-diphosphate kinase (ATP:dUDP)	(VBICanLib238577_0279)	Pyrimidine Metabolism
NDPK7	Nucleoside-diphosphate kinase (ATP:dCDP)	(VBICanLib238577_0279)	Pyrimidine Metabolism
NDPK8	Nucleoside-diphosphate kinase (ATP:dADP)	(VBICanLib238577_0279)	Purine Metabolism
NNATr	Nicotinate-nucleotide adenylyltransferase	(VBICanLib238577_0574)	Nicotinate and Nicotinamide
NO2t2rpp	Nitrite transport in via proton symport, reversible (periplasm)	(VBICanLib238577_0908)	Transport
OIVD1r	2-oxoisovalerate dehydrogenase (acylating; 4-methyl-2-oxopentaoate)	(VBICanLib238577_0973)	Valine, leucine and isoleucine degradation
OIVD2	2-oxoisovalerate dehydrogenase (acylating; 3-methyl-2-oxobutanoate)	(VBICanLib238577_0973)	Valine, leucine and isoleucine degradation
OIVD3	2-oxoisovalerate dehydrogenase (acylating; 3-methyl-2-oxopentanoate)	(VBICanLib238577_0973)	Valine, leucine and isoleucine degradation
ORNCD	Ornithine cyclodeaminase	(VBICanLib238577_1196) or (VBICanLib238577_0962)	Arginine and Proline Metabolism
ORNabcpp	Ornithine transport via ABC system (periplasm)	(VBICanLib238577_0042)	Lysine Metabolism
ORPT	Orotate phosphoribosyltransferase	(VBICanLib238577_0936)	Pyrimidine Metabolism
PANTS	Pantothenate synthase	(VBICanLib238577_0603)	Pantothenate and CoA Biosynthesis
PAPPT3	Phospho-N-acetylmuramoyl-pentapeptide-transferase (meso-2,6-diaminopimelate)	(VBICanLib238577_1025)	Peptidoglycan Biosynthesis
PDH	Pyruvate dehydrogenase	(VBICanLib238577_0546)	Glycolysis
PDX5POi	Pyridoxine 5-phosphate oxidase	(VBICanLib238577_0880)	Vitamin B6 Metabolism
PGM	Phosphoglycerate mutase	(VBICanLib238577_0158)	Gluconeogenesis
PGMT	Phosphoglucomutase	(VBICanLib238577_1032)	Glycolysis

PIuabcpp	Phosphate transport via ABC system (uptake, periplasm)	(VBICanLib238577_0519)	Transport
PMDPHT	Pyrimidine phosphatase	(VBICanLib238577_0034)	Riboflavin Metabolism
PNTK	Pantothenate kinase	(VBICanLib238577_0391)	Pantothenate and CoA Biosynthesis
PPA	Inorganic diphosphatase	(VBICanLib238577_0108)	Oxidative Phosphorylation
PPBNGS	Porphobilinogen synthase	(VBICanLib238577_0520)	Porphyrin and Chlorophyll Metabolism
PPPGO	Protoporphyrinogen oxidase (aerobic)	(VBICanLib238577_0996)	Porphyrin and Chlorophyll Metabolism
PRAGSr	Phosphoribosylglycinamide synthase	((VBICanLib238577_0108)	Purine Metabolism
PRAIi	Phosphoribosylanthranilate isomerase (irreversible)	(VBICanLib238577_0003)	Phenylalanine Tyrosine Tryptophan Biosynthesis
PRAIS	Phosphoribosylaminoimidazole synthase	(VBICanLib238577_0885)	Purine Metabolism
PRFGS	Phosphoribosylformylglycinamidine synthase	(VBICanLib238577_0882)	Purine Metabolism
PROTRS	Prolyl-tRNA synthetase	(VBICanLib238577_0771)	Arginine and Proline Metabolism
PROabcpp	L-proline transport via ABC system (periplasm)	(VBICanLib238577_1180)	Arginine and Proline Metabolism
PRPPS	Phosphoribosylpyrophosphate synthetase	(VBICanLib238577_0299)	Ribose Metabolism
PSP_L	Phosphoserine phosphatase (L-serine)	(VBICanLib238577_0870)	Glycine, Serine and threonine metabolism
PTRCabcpp	Putrescine transport via ABC system (periplasm)	(VBICanLib238577_0667) or (VBICanLib238577_0629) or (VBICanLib238577_0227)	Transport
RIBabcpp	D-ribose transport via ABC system (periplasm)	(VBICanLib238577_0667) or (VBICanLib238577_0629)	Transport
RNDR4	Ribonucleoside-diphosphate reductase (UDP)	(VBICanLib238577_0723 and VBICanLib238577_0059) or (VBICanLib238577_0749) or (VBICanLib238577_0876) or (VBICanLib238577_0930) or (VBICanLib238577_1220)	Pyrimidine Metabolism
RPE	Ribulose 5-phosphate 3-epimerase	(VBICanLib238577_0227)) or ((VBICanLib238577_0667)	Pentose Phosphate Pathway
RPI	Ribose-5-phosphate isomerase	(VBICanLib238577_0976)	Pentose Phosphate Pathway
SDPTA	Succinyldiaminopimelate transaminase	(VBICanLib238577_0832)	Lysine Biosynthesis
SERAT	Serine O-acetyltransferase	(VBICanLib238577_0540)	Cysteine Metabolism
SERTRS	Seryl-tRNA synthetase	(VBICanLib238577_0542)	Glycine, Serine and threonine metabolism

SHCHF	Sirohydrochlorin ferrochelatase	(VBICanLib238577_0613)	Porphyrin and Chlorophyll Metabolism
SOTA	Succinylornithine transaminase	(VBICanLib238577_0542)	Arginine and Proline Metabolism
SPMDabcpp	Spermidine transport via ABC system (periplasm)	(VBICanLib238577_0290)	Transport
SUCD1	Succinate dehydrogenase	(VBICanLib238577_0965 and VBICanLib238577_0966 and VBICanLib238577_0964)	TCA Cycle
SUCDi	Succinate dehydrogenase (irreversible)	(VBICanLib238577_0965 and VBICanLib238577_0966 and VBICanLib238577_0964)	TCA Cycle
SULR	Sulfite reductase (NADPH2)	(VBICanLib238577_0872)	Sulfur Metabolism
SULabcpp	Sulfate transport via ABC system (periplasm)	(VBICanLib238577_0797)	Sulfur Metabolism
TRPS3	Tryptophan synthase (indoleglycerol phosphate)	(VBICanLib238577_0507)	Phenylalanine Tyrosine Tryptophan Biosynthesis
TRSARr	Tartronate semialdehyde reductase	(VBICanLib238577_0667) or (VBICanLib238577_0227)	Glycolate metabolism
UAMAS	UDP-N-acetylmuramoyl-L-alanine synthetase	(VBICanLib238577_0632)	Glutamate metabolism
UGMDDS	UDP-N-acetylmuramoyl-L-alanyl-D-glutamyl-meso-2,6-diaminopimeloyl-D-alanyl-D-alanine synthetase	(VBICanLib238577_0444)	Peptidoglycan Biosynthesis
UPPN	B-ureidopropionase	(VBICanLib238577_0787)	Pantothenate and CoA Biosynthesis
VANKpp	VANKpp	(VBICanLib238577_0879)	Transport
VANLNtex	VANLNtex	(VBICanLib238577_0879)	Transport
VANLTtex	VANLTtex	(VBICanLib238577_0879)	Transport
VNLNpp	VNLNpp	(VBICanLib238577_0879)	Transport
pentso3abcpp	Pentso3abcpp	(VBICanLib238577_0629)	Transport
ACOLIPAabctex	Arabinose modified core oligosaccharide lipid A transport via ABC system (periplasm to extracellular)	((VBICanLib238577_0282 and VBICanLib238577_0481) or (VBICanLib238577_0689) or (VBICanLib238577_0667) or (VBICanLib238577_0002) or (VBICanLib238577_0520))	Lipopolysaccharide Biosynthesis / Recycling
ALATA_D2	D-alanine transaminase	((VBICanLib238577_0237) or (VBICanLib238577_0985))	Cofactor and Prosthetic Group Biosynthesis

ALATA_L2	Alanine transaminase	((VBICanLib238577_0237) or (VBICanLib238577_0985))	Cofactor and Prosthetic Group Biosynthesis
AMPTASECG	Alanyl aminopeptidase (cys-gly)	(VBICanLib238577_0281)	Cysteine Metabolism
AMPTASEPG	Aminopeptidase (pro-gly)	(VBICanLib238577_0985) or (VBICanLib238577_1016)	Arginine and Proline Metabolism
ASCBptspp	L-ascorbate transport via PEP:Pyr PTS (periplasm)	(VBICanLib238577_1100)	Transport, Inner Membrane
BUTSO3abcpp	Butanesulfonate transport via ABC system (periplasm)	(VBICanLib238577_0629 and VBICanLib238577_0628 and VBICanLib238577_0228)	Transport, Inner Membrane
CLIPAabctex	Cold lipid A transport via ABC system (periplasm to extracellular)	(VBICanLib238577_0282 and VBICanLib238577_0481) or (VBICanLib238577_0689)	Lipopolysaccharide Biosynthesis / Recycling
COLIPAPabctex	Core oligosaccharide lipid A diphosphate transport via ABC system (periplasm to extracellular)	(VBICanLib238577_0282 and VBICanLib238577_0481)	Lipopolysaccharide Biosynthesis / Recycling
COLIPAabctex	Core oligosaccharide lipid A transport via ABC system (periplasm to extracellular)	(VBICanLib238577_0282 and VBICanLib238577_0481)	Lipopolysaccharide Biosynthesis / Recycling
CRNDabcpp	D-carnitine transport via ABC system (periplasm)	(VBICanLib238577_0228 and VBICanLib238577_0227) or (VBICanLib238577_0520) or (VBICanLib238577_0629) or (VBICanLib238577_0667)	Transport, Inner Membrane
CYSSADS	L-cysteine sulfinic acid desulfurase	(VBICanLib238577_1016) or (VBICanLib238577_0985)	Cysteine Metabolism
ECA4COLIPAabctex	Enterobacterial common antigen (x4) core oligosaccharide lipid A transport via ABC system (periplasm to extracellular)	(VBICanLib238577_0480 and VBICanLib238577_0496 and VBICanLib238577_0481 and VBICanLib238577_0282)	Lipopolysaccharide Biosynthesis / Recycling
ENLIPAabctex	Phosphoethanolamine lipid A transport via ABC system (periplasm to extracellular)	(VBICanLib238577_0481 and VBICanLib238577_0282 and VBICanLib238577_0480)	Lipopolysaccharide Biosynthesis / Recycling
GRXR	Glutaredoxin reductase	(VBICanLib238577_0880)	Unassigned
K2L4Aabctex	KDO(2)-lipid IV A transport via ABC system (periplasm to extracellular)	(VBICanLib238577_0822)	Lipopolysaccharide Biosynthesis / Recycling
KAS15	Beta-ketoacyl-ACP synthase (2)	(VBICanLib238577_0694)	Membrane Lipid Metabolism

KDOPS	3-deoxy -D-manno-octulosonic -acid 8-phosphate synthase	(VBICanLib238577_0553)	Lipopolysaccharide Biosynthesis / Recycling
LIPAabctex	Lipid A transport via ABC system (periplasm to extracellular)	(VBICanLib238577_0822)	Lipopolysaccharide Biosynthesis / Recycling
NADH17pp	NADH dehydrogenase (menaquinone-8 & 3 protons) (periplasm)	(VBICanLib238577_0754 and VBICanLib238577_0755 and VBICanLib238577_0756 and VBICanLib238577_0757 and VBICanLib238577_0758 and VBICanLib238577_0759 and VBICanLib238577_0760 and VBICanLib238577_0761 and VBICanLib238577_0762 and VBICanLib238577_0763 and VBICanLib238577_0764 and VBICanLib238577_0765 and VBICanLib238577_0766 and VBICanLib238577_0767)	Oxidative Phosphorylation
NADH18pp	NADH dehydrogenase (demethylmenaquinone-8 & 3 protons) (periplasm)	(VBICanLib238577_0754 and VBICanLib238577_0755 and VBICanLib238577_0756 and VBICanLib238577_0757 and VBICanLib238577_0758 and VBICanLib238577_0759 and VBICanLib238577_0760 and VBICanLib238577_0761 and VBICanLib238577_0762 and VBICanLib238577_0763 and VBICanLib238577_0764 and VBICanLib238577_0765 and VBICanLib238577_0766 and VBICanLib238577_0767)	Oxidative Phosphorylation
NADHHR	NADH hydratase	(VBICanLib238577_0569)	Metabolite Repair
NADHHS	NADH hydratase	(VBICanLib238577_0569)	Metabolite Repair
NADPHHR	NADPH hydratase	(VBICanLib238577_0569)	Metabolite Repair
NADPHHS	NADPH hydratase	(VBICanLib238577_0569)	Metabolite Repair

O16A4COLIPAabctex	O16 antigen (x4) core oligosaccharide lipid A transport via ABC system (periplasm to extracellular)	(VBICanLib238577_0479 and VBICanLib238577_0480 and VBICanLib238577_0481)	Lipopolysaccharide Biosynthesis / Recycling
OCTDPS	Octaprenyl pyrophosphate synthase	(VBICanLib238577_0279))	Cofactor and Prosthetic Group Biosynthesis
OMBZLM	2-Octaprenyl-6-methoxy-benzoquinol methylase	(VBICanLib238577_0279))	Cofactor and Prosthetic Group Biosynthesis
RNDR4b	Ribonucleoside-diphosphate reductase (UDP) (glutaredoxin)	(VBICanLib238577_0457)	Nucleotide Salvage Pathway
S2FE2SR	SUF [2Fe-2S] regeneration	(VBICanLib238577_0918 and VBICanLib238577_0985 and VBICanLib238577_0984 and VBICanLib238577_0983)	Cofactor and Prosthetic Group Biosynthesis
S2FE2SS	SUF [2Fe-2S] Synthesis	(VBICanLib238577_0918 and VBICanLib238577_0985 and VBICanLib238577_0984 and VBICanLib238577_0983)	Cofactor and Prosthetic Group Biosynthesis
S2FE2SS2	SUF [2Fe-2S] Synthesis II	(VBICanLib238577_0918 and VBICanLib238577_0985 and VBICanLib238577_0984 and VBICanLib238577_0983)	Cofactor and Prosthetic Group Biosynthesis
S2FE2ST	SUF [2Fe-2S] Transfer	(VBICanLib238577_0918 and VBICanLib238577_0985 and VBICanLib238577_0984 and VBICanLib238577_0983)	Cofactor and Prosthetic Group Biosynthesis
S4FE4SR	SUF [4Fe-4S] Reduction	(VBICanLib238577_0918 and VBICanLib238577_0985 and VBICanLib238577_0984 and VBICanLib238577_0983)	Cofactor and Prosthetic Group Biosynthesis
S4FE4ST	SUF [4Fe-4S] Transfer	(VBICanLib238577_0918 and VBICanLib238577_0985 and VBICanLib238577_0984 and VBICanLib238577_0983)	Cofactor and Prosthetic Group Biosynthesis
SCYSDS	SUF Cysteine desulfuration	(VBICanLib238577_0985)	Cofactor and Prosthetic Group Biosynthesis
FEex	Iron transporter	(VBICanLib238577_0822 and VBICanLib238577_0649) or (VBICanLib238577_0065)	iron metabolism

FEpp	Iron transporter	(VBICanLib238577_0649 and VBICanLib238577_0822)	iron metabolism
RNDR3b	Ribonucleoside-diphosphate reductase (CDP) (glutaredoxin)	((VBICanLib238577_0723) and (VBICanLib238577_0059)) or (VBICanLib238577_0749) or (VBICanLib238577_0876) or (VBICanLib238577_0930) or (VBICanLib238577_1220)	Nucleotide Salvage Pathway
4HTHRS	4-Hydroxy-L-threonine synthase	(Ishi_364)	Vitamin B6 Metabolism
ACCOAC	Acetyl-CoA carboxylase	((Ishi_405) and (Ishi_147) and (Ishi_646) and (Ishi_148))	Fatty Acid Biosynthesis
ACGK	Acetylglutamate kinase	((Ishi_148) and (Ishi_405) and (Ishi_646) and (Ishi_147))	Urea cycle/amino group metabolism
ACGS	N-acetylglutamate synthase	(Ishi_384)	Urea cycle/amino group metabolism
ACHBS	2-aceto-2-hydroxybutanoate synthase	((Ishi_425) and (Ishi_972)) and (Ishi_845))	Valine, Leucine, and Isoleucine Metabolism
ACOATA	Acetyl-CoA ACP transacylase	(Ishi_738))	Fatty Acid Biosynthesis
ACONT	Aconitate hydratase	(Ishi_349)	TCA Cycle
ACONTa	Aconitase (half-reaction A, Citrate hydro-lyase)	((Ishi_349)	TCA Cycle
ACONTb	Aconitase (half-reaction B, Isocitrate hydro-lyase)	(Ishi_349)	TCA Cycle
ACOTA	Acetylornithine transaminase	(Ishi_349)	Urea cycle/amino group metabolism
ACt2rpp	Acetate reversible transport via proton symport (periplasm)	((Ishi_349)	Transport
ADSL1r	Adenylsuccinate lyase	(Ishi_897)	Purine Metabolism
ADSL2r	Adenylosuccinate lyase	(Ishi_897)	Purine Metabolism
AGPAT160	1-hexadecanoyl-sn-glycerol 3-phosphate O-acyltransferase (n-C16:0)	(Ishi_654)	Glycerophospholipid Metabolism
AGPAT161	1-hexadec-7-enoyl-sn-glycerol 3-phosphate O-acyltransferase (n-C16:1)	(Ishi_654)	Glycerophospholipid Metabolism
AKGDH	2-Oxogluterate dehydrogenase	(Ishi_982) and (Ishi_983))	TCA Cycle
ALAALAr	D-alanine-D-alanine ligase (reversible)	(Ishi_1117))	Peptidoglycan Biosynthesis
ANPRT	Anthranilate phosphoribosyltransferase	(Ishi_308)	Phenylalanine Tyrosine Tryptophan Biosynthesis
ANS	Anthranilate synthase	(Ishi_308) or (Ishi_316)	Phenylalanine Tyrosine Tryptophan Biosynthesis
ARGSS	Argininosuccinate synthase	(Ishi_113)	Arginine and Proline Metabolism

ARGabcpp	L-arginine transport via ABC system (periplasm)	(Ishi_47) or (Ishi_47)	Transport
ASAD	Aspartate-semialdehyde dehydrogenase	(Ishi_191)	Lysine Biosynthesis
ASPabcpp	L-aspartate transport via ABC system (periplasm)	((Ishi_46) and (Ishi_45) and (Ishi_1045)) or ((Ishi_263))	Transport
ATPPRT	ATP phosphoribosyltransferase	(Ishi_786)	Histidine Metabolism
ATPS4rpp	ATP synthase (four protons for one ATP) (periplasm)	((Ishi_1046) and (Ishi_1046)) or ((Ishi_46) and (Ishi_45) and (Ishi_44) and (Ishi_47))	Oxidative Phosphorylation
BZt1pp	Benzoate transport in via proton symport (periplasm)	(Ishi_280)	B-Ketoadipate pathway
CHLabcpp	Choline transport via ABC system (periplasm)	(Ishi_230)) or (Ishi_47) or ((Ishi_525) and (Ishi_231)	Transport
DAPE	Diaminopimelate epimerase	(Ishi_518))	Lysine Biosynthesis
CHOLSabc	CHOLSabc	((Ishi_232) or (Ishi_230)) or (Ishi_525) or (Ishi_638) or (Ishi_677)	Glycine, Serine and threonine metabolism
COBALT2tpp	Cobalt transport in via permease (no H+)	(Ishi_158)	Transport
CS	Citrate synthase	((Ishi_230) and (Ishi_231)	TCA Cycle
CTPS2	CTP synthase (glutamine)	((Ishi_230) and (Ishi_231)	Pyrimidine Metabolism
CYO1b	CYO1b	((Ishi_373) and (Ishi_374))	Oxidative Phosphorylation
CYSabcpp	L-cysteine uptake via ABC system (periplasm)	(Ishi_658)) or (Ishi_69) or ((Ishi_834) and (Ishi_658)) or (Ishi_69) or ((Ishi_834)	Transport
DAPDC	Diaminopimelate decarboxylase	(Ishi_264)	Lysine Biosynthesis
DASYN160	CDP-diacylglycerol synthetase (n-C16:0)	(Ishi_452)	Glycerophospholipid Metabolism
DASYN161	CDP-diacylglycerol synthetase (n-C16:1)	(Ishi_452)	Glycerophospholipid Metabolism
DB4PS	3,4-Dihydroxy-2-butanone-4-phosphate synthase	(Ishi_252)	Riboflavin Metabolism
DDPA	3-deoxy-D-arabino-heptulosonate 7-phosphate synthetase	(Ishi_560)	Phenylalanine Tyrosine Tryptophan Biosynthesis
DHFR	Dihydrofolate reductase	(Ishi_878)	One Carbon pool by folate
DHORTS	Dihydroorotase	(Ishi_947)	Pyrimidine Metabolism
DMATT	Dimethylallyltranstransferase	(Ishi_1044)	Steroid biosynthesis
DNTPPA	Dihydroneopterin triphosphate pyrophosphatase	(Ishi_211)	Folate Biosynthesis

DPCOAK	Dephospho-CoA kinase	(Ishi_407)	Pantothenate and CoA Biosynthesis
DPR	2-dehydropantoate 2-reductase	(Ishi_1032)	Pantothenate and CoA Biosynthesis
DTMPK	DTMP kinase	(Ishi_96)	Pyrimidine Metabolism
DXPS	1-deoxy-D-xylulose 5-phosphate synthase	(Ishi_555)	Steroid biosynthesis
E4PD	Erythrose 4-phosphate dehydrogenase	((Ishi_1046) and (Ishi_47)) or ((Ishi_525) and (Ishi_1045))	Vitamin B6 Metabolism
EAR100y	Enoyl-[acyl-carrier-protein] reductase (NADPH) (n-C10:0)	(Ishi_47)	Fatty Acid Biosynthesis
EAR120y	Enoyl-[acyl-carrier-protein] reductase (NADPH) (n-C12:0)	((Ishi_230) and (Ishi_231)	Fatty Acid Biosynthesis
EAR121y	Enoyl-[acyl-carrier-protein] reductase (NADPH) (n-C12:1)	(Ishi_230) and (Ishi_231))	Fatty Acid Biosynthesis
EAR160y	Enoyl-[acyl-carrier-protein] reductase (NADPH) (n-C16:0)	((Ishi_230) and (Ishi_231))	Fatty Acid Biosynthesis
EAR161y	Enoyl-[acyl-carrier-protein] reductase (NADPH) (n-C16:1)	(Ishi_47))	Fatty Acid Biosynthesis
EAR180y	Enoyl-[acyl-carrier-protein] reductase (NADPH) (n-C18:0)	(Ishi_891)	Fatty Acid Biosynthesis
EAR181y	Enoyl-[acyl-carrier-protein] reductase (NADPH) (n-C18:1)	(Ishi_638)	Fatty Acid Biosynthesis
EAR40y	Enoyl-[acyl-carrier-protein] reductase (NADPH) (n-C4:0)	((Ishi_231) and (Ishi_230)	Fatty Acid Biosynthesis
EAR60y	Enoyl-[acyl-carrier-protein] reductase (NADPH) (n-C6:0)	(Ishi_1105) or (Ishi_361)	Fatty Acid Biosynthesis
EAR80y	Enoyl-[acyl-carrier-protein] reductase (NADPH) (n-C8:0)	((Ishi_525)	Fatty Acid Biosynthesis
ENO	Enolase	((Ishi_372) and (Ishi_371) and (Ishi_374) and (Ishi_373))	Gluconeogenesis
ETHSO3abcpp	Ethanesulfonate transport via ABC system (periplasm)	((Ishi_638) and (Ishi_637) and (Ishi_770))	Transport
FDH	Formate dehydrogenase	(Ishi_770) and (Ishi_769)	Methane Metabolism
FE2abc	Iron (II) transport via ABC system	(Ishi_230) or ((Ishi_677)	Transport
FTHFD	Formyltetrahydrofolate deformylase	(Ishi_827) or (Ishi_666)	One Carbon pool by folate
FUM	Fumarase	(Ishi_577) or ((Ishi_578) or (Ishi_577)) or (Ishi_578)	TCA Cycle

FUMtex	Fumarate transport via diffusion (extracellular to periplasm)	(Ishi_547)	Transport
GLCabcpp	D-glucose transport via ABC system (periplasm)	(Ishi_65)	Transport
GLNS	Glutamine synthetase	(Ishi_47)) or (Ishi_638)	Glutamate Metabolism
GLNTRAT	Glutamyl-tRNA(Gln) amidotransferase	((Ishi_902) and (Ishi_904) and (Ishi_903))	Glutamate Metabolism
GLUSy	Glutamate synthase (NADPH)	(Ishi_164)	Glutamate Metabolism
GLYBabcpp	Glycine betaine transport via ABC system (periplasm)	((Ishi_280)	Arginine and Proline Metabolism
GLYCL	Glycine Cleavage System	(Ishi_553)) or (Ishi_984) or (Ishi_986) or (Ishi_553)) or (Ishi_984) or ((Ishi_986)	Glycine, Serine and threonine metabolism
GLYCLTt2rpp	Glycolate transport via proton symport, reversible (periplasm)	(Ishi_1030) and (Ishi_553)) or (Ishi_984) or ((Ishi_986)	Transport
GLYCLTtex	Glycolate transport via diffusion (extracellular to periplasm)	(Ishi_553)) or (Ishi_984) or (Ishi_986) or (Ishi_553)) or (Ishi_984) or ((Ishi_986)	Transport
GLYCL_2	Glycine cleavage system, cytosol	(Ishi_553)) or (Ishi_984) or (Ishi_986) or (Ishi_553)) or (Ishi_984) or ((Ishi_986)	Nitrogen Metabolism
GLYCTO1	Glycolate oxidase	((Ishi_468)	Glycolate metabolism
GLYTRS	Glycyl-tRNA synthetase	(Ishi_47))	Glycine, Serine and threonine metabolism
GMPS2	GMP synthase	(Ishi_471))	Purine Metabolism
GND	Phosphogluconate dehydrogenase	(Ishi_283))	Pentose Phosphate Pathway
GRTT	Geranyltranstransferase	(Ishi_1044)	Steroid biosynthesis
GTHS	Glutathione synthetase	(Ishi_891)	Glutamate Metabolism
GTPCII	GTP cyclohydrolase II	((Ishi_252) or (Ishi_252))	Riboflavin Metabolism
HCO3E	HCO3 equilibration reaction	(Ishi_567)	One Carbon pool by folate
HEMEOS	Heme O synthase	(Ishi_253)	Porphyrin and Chlorophyll Metabolism
HEX1	Hexokinase (D-glucose:ATP)	(Ishi_161)	Glycolysis
HISabcpp	L-histidine transport via ABC system (periplasm)	(Ishi_60)	Transport
HMBS	Hydroxymethylbilane synthase	(Ishi_969)	Porphyrin and Chlorophyll Metabolism
HSK	Homoserine kinase	(Ishi_431)	Glycine, Serine and threonine metabolism
Htex	Proton transport via diffusion (extracellular to periplasm)	(Ishi_69)	Transport
IGPS	Indole-3-glycerol-phosphate synthase	(Ishi_10)	Phenylalanine Tyrosine Tryptophan Biosynthesis

ILEabcpp	L-isoleucine transport via ABC system (periplasm)	(Ishi_810)	Transport
IMPC	IMP cyclohydrolase	(Ishi_1050)	Purine Metabolism
IMPD	IMP dehydrogenase	(Ishi_803)	Purine Metabolism
IPDDI	Isopentenyl-diphosphate D-isomerase	(Ishi_1006)	Steroid biosynthesis
IPDPS	1-hydroxy-2-methyl-2-(E)-butenyl 4-diphosphate reductase (ipdp)	(Ishi_962)	Steroid biosynthesis
IPMD	3-isopropylmalate dehydrogenase	(Ishi_819)	Valine, Leucine, and Isoleucine Metabolism
IPPMIa	3-isopropylmalate dehydratase	(Ishi_349) or (Ishi_735)	Valine, Leucine, and Isoleucine Metabolism
IPPMIb	2-isopropylmalate hydratase	(Ishi_349) or (Ishi_735)	Valine, Leucine, and Isoleucine Metabolism
ISETACabcpp	Isethionate transport via ABC system (periplasm)	(Ishi_638) or (Ishi_230)	Transport
KAS14	Beta-ketoacyl-ACP synthase	(Ishi_360)	Fatty Acid Biosynthesis
LEUTRS	Leucyl-tRNA synthetase	(Ishi_424)	Valine, Leucine, and Isoleucine Metabolism
LEUabcpp	L-leucine transport via ABC system (periplasm)	(Ishi_810)	Transport
LYSTRS	Lysyl-tRNA synthetase	(Ishi_1051)	Lysine Biosynthesis
LYSabcpp	L-lysine transport via ABC system (periplasm)	((Ishi_46) and (Ishi_44) and (Ishi_45) and (Ishi_47))	Lysine Metabolism
MACPD	Malonyl-ACP decarboxylase	(Ishi_308)	Fatty Acid Biosynthesis
MALt2_2pp	Malate transport via proton symport (2 H) (periplasm)	(Ishi_986)	Transport
MCITS	2-methylcitrate synthase	(Ishi_248)	Propanoate Metabolism
MCOATA	Malonyl-CoA-ACP transacylase	(Ishi_533)	Fatty Acid Biosynthesis
ME1	Malic enzyme (NAD)	(Ishi_7)	Glycolysis
METAT	Methionine adenosyltransferase	((Ishi_798) or (Ishi_798))	Methionine Metabolism
METabcpp	L-methionine transport via ABC system (periplasm)	(Ishi_47)	Transport
MICITDr	2-methylisocitrate dehydratase	(Ishi_349)	Propanoate Metabolism
MSO3abcpp	Methanesulfonate transport via ABC system (periplasm)	(Ishi_638)	Transport
MTHFC	Methenyltetrahydrofolate cyclohydrolase	(Ishi_730)	One Carbon pool by folate
MTHFD	Methylenetetrahydrofolate dehydrogenase (NADP)	(Ishi_730)	One Carbon pool by folate

NADH16pp	NADH dehydrogenase (ubiquinone-8 & 3 protons) (periplasm)	(Ishi_764) and (Ishi_765) and (Ishi_766) and (Ishi_767) and (Ishi_768) and (Ishi_769) and (Ishi_770) and (Ishi_771) and (Ishi_772) and (Ishi_773) and (Ishi_774) and (Ishi_775) and (Ishi_776) and (Ishi_777)	Oxidative Phosphorylation
NADK	NAD kinase	(Ishi_474)	Nicotinate and Nicotinamide
NADS2	Nicotinate-mononucleotide adenylyltransferase	(Ishi_930)	Nicotinate and Nicotinamide
NAMNPP	Nicotinic acid mononucleotide pyrophosphorylase	(Ishi_353)	Nicotinate and Nicotinamide
NAt3_2pp	Sodium proton antiporter (H:NA is 2) (periplasm)	(Ishi_255)	Transport
NDPK1	Nucleoside-diphosphate kinase (ATP:GDP)	(Ishi_283)	Purine Metabolism
NDPK2	Nucleoside-diphosphate kinase (ATP:UDP)	(Ishi_283)	Pyrimidine Metabolism
NDPK3	Nucleoside-diphosphate kinase (ATP:CDP)	(Ishi_283)	Pyrimidine Metabolism
NDPK4	Nucleoside-diphosphate kinase (ATP:dTDP)	(Ishi_283)	Pyrimidine Metabolism
NDPK5	Nucleoside-diphosphate kinase (ATP:dGDP)	(Ishi_283)	Purine Metabolism
NDPK6	Nucleoside-diphosphate kinase (ATP:dUDP)	(Ishi_283)	Pyrimidine Metabolism
NDPK7	Nucleoside-diphosphate kinase (ATP:dCDP)	(Ishi_283)	Pyrimidine Metabolism
NDPK8	Nucleoside-diphosphate kinase (ATP:dADP)	(Ishi_283)	Purine Metabolism
NNATr	Nicotinate-nucleotide adenylyltransferase	(Ishi_584)	Nicotinate and Nicotinamide
NO2t2rpp	Nitrite transport in via proton symport, reversible (periplasm)	(Ishi_920)	Transport
NTD10	5-nucleotidase (XMP)	(Ishi_548)	Purine Metabolism
NTD11	5-nucleotidase (IMP)	(Ishi_548)	Purine Metabolism
NTD2	5-nucleotidase (UMP)	(Ishi_548)	Pyrimidine Metabolism
NTD3	5-nucleotidase (dCMP)	(Ishi_548)	Pyrimidine Metabolism

NTD4	5-nucleotidase (CMP)	(Ishi_548)	Pyrimidine Metabolism
NTD5	5-nucleotidase (dTMP)	(Ishi_548)	Pyrimidine Metabolism
NTD6	5-nucleotidase (dAMP)	(Ishi_548)	Purine Metabolism
NTD7	5-nucleotidase (AMP)	(Ishi_548)	Purine Metabolism
NTD8	5-nucleotidase (dGMP)	(Ishi_548)	Purine Metabolism
NTD9	5-nucleotidase (GMP)	(Ishi_548)	Purine Metabolism
OCBT	Ornithine carbamoyltransferase	(Ishi_280)	Urea cycle/amino group metabolism
OIVD1r	2-oxoisovalerate dehydrogenase (acylating; 4-methyl-2-oxopentaoate)	(Ishi_554) or ((Ishi_983) and (Ishi_553)) or (Ishi_984) or ((Ishi_986) and (Ishi_555)))	Valine, leucine and isoleucine degradation
OIVD2	2-oxoisovalerate dehydrogenase (acylating; 3-methyl-2-oxobutanoate)	(Ishi_554) or ((Ishi_983) and (Ishi_553)) or (Ishi_984) or ((Ishi_986) and (Ishi_555)))	Valine, leucine and isoleucine degradation
OIVD3	2-oxoisovalerate dehydrogenase (acylating; 3-methyl-2-oxopentanoate)	(Ishi_554) or ((Ishi_983) and (Ishi_553)) or (Ishi_984) or ((Ishi_986) and (Ishi_555)))	Valine, leucine and isoleucine degradation
OMPDC	Orotidine-5-phosphate decarboxylase	(Ishi_351)	Pyrimidine Metabolism
ORNabcpp	Ornithine transport via ABC system (periplasm)	(Ishi_701)	Lysine Metabolism
ORPT	Orotate phosphoribosyltransferase	(Ishi_946)	Pyrimidine Metabolism
OXOADLR	OXOADLR	(Ishi_983)	Lysine Metabolism
PAPPT3	Phospho-N-acetylmuramoyl-pentapeptide-transferase (meso-2,6-diaminopimelate)	(Ishi_7)	Peptidoglycan Biosynthesis
PC	Pyruvate carboxylase	(Ishi_148)	TCA Cycle
PDH	Pyruvate dehydrogenase	(Ishi_319)	Glycolysis
PDX5POi	Pyridoxine 5-phosphate oxidase	(Ishi_1104)	Vitamin B6 Metabolism
PDX5PS	Pyridoxine 5-phosphate synthase	(Ishi_335) and (Ishi_291)	Vitamin B6 Metabolism
PE120abcpp	Phosphatidylethanolamine transport via ABC system (n-C12:0, periplasm)	(Ishi_658)) or (Ishi_69) or ((Ishi_834)	Glycerophospholipid Metabolism
PE160abcpp	Phosphatidylethanolamine transport via ABC system (n-C16:0, periplasm)	(Ishi_658)) or (Ishi_69) or ((Ishi_834)	Glycerophospholipid Metabolism
PE161abcpp	Phosphatidylethanolamine transport via ABC system (n-C16:1, periplasm)	(Ishi_658)) or (Ishi_69) or ((Ishi_834)	Glycerophospholipid Metabolism
PE180abcpp	Phosphatidylethanolamine transport via ABC system (n-C18:0, periplasm)	(Ishi_658)) or (Ishi_69) or ((Ishi_834)	Glycerophospholipid Metabolism
PE181abcpp	Phosphatidylethanolamine transport via ABC system (n-C18:1, periplasm)	(Ishi_658)) or (Ishi_69) or ((Ishi_834)	Glycerophospholipid Metabolism

PGAMT	Phosphoglucosamine mutase	((Ishi_46) and (Ishi_45) and (Ishi_44) and (Ishi_47))	Aminosugar Metabolism
PGL	6-phosphogluconolactonase	(Ishi_349)	Glycolysis
PGM	Phosphoglycerate mutase	(Ishi_159)	Gluconeogenesis
PGP120abcpp	Phosphatidylglycerophosphate transport via ABC system (n-C12:0, periplasm)	(Ishi_658)) or (Ishi_69) or ((Ishi_834)	Glycerophospholipid Metabolism
PGP160abcpp	Phosphatidylglycerophosphate transport via ABC system (n-C16:0, periplasm)	(Ishi_658)) or (Ishi_69) or ((Ishi_834)	Glycerophospholipid Metabolism
PGP161abcpp	Phosphatidylglycerophosphate transport via ABC system (n-C16:1, periplasm)	(Ishi_658)) or (Ishi_69) or ((Ishi_834)	Glycerophospholipid Metabolism
PGP180abcpp	Phosphatidylglycerophosphate transport via ABC system (n-C18:0, periplasm)	(Ishi_658)) or (Ishi_69) or ((Ishi_834)	Glycerophospholipid Metabolism
PGP181abcpp	Phosphatidylglycerophosphate transport via ABC system (n-C18:1, periplasm)	(Ishi_658)) or (Ishi_69) or ((Ishi_834)	Glycerophospholipid Metabolism
PGSA120	Phosphatidylglycerol synthase (n-C12:0)	(Ishi_634)	Glycerophospholipid Metabolism
PGSA160	Phosphatidylglycerol synthase (n-C16:0)	(Ishi_634)	Glycerophospholipid Metabolism
PGSA161	Phosphatidylglycerol synthase (n-C16:1)	(Ishi_634)	Glycerophospholipid Metabolism
PGSA180	Phosphatidylglycerol synthase (n-C18:0)	(Ishi_634)	Glycerophospholipid Metabolism
PGSA181	Phosphatidylglycerol synthase (n-C18:1)	(Ishi_634)	Glycerophospholipid Metabolism
PHETRS	Phenylalanyl-tRNA synthetase	((Ishi_730) or (Ishi_730))	Phenylalanine Tyrosine Tryptophan Biosynthesis
PIuabcpp	Phosphate transport via ABC system (uptake, periplasm)	((Ishi_730) or (Ishi_730))	Transport
PNTK	Pantothenate kinase	(Ishi_280)	Pantothenate and CoA Biosynthesis
PPA	Inorganic diphosphatase	(Ishi_111)	Oxidative Phosphorylation
PPBNGS	Porphobilinogen synthase	(Ishi_283)	Porphyrin and Chlorophyll Metabolism
PPCDC	Phosphopantothenoylcysteine decarboxylase	((Ishi_283) or (Ishi_283))	Pantothenate and CoA Biosynthesis
PPNCL	Phosphopantothenate-cysteine ligase	(Ishi_612)	Pantothenate and CoA Biosynthesis
PRAGSr	Phosphoribosylglycinamide synthase	((Ishi_283) or (Ishi_283))	Purine Metabolism
PRAIi	Phosphoribosylanthranilate isomerase (irreversible)	(Ishi_10)	Phenylalanine Tyrosine Tryptophan Biosynthesis
PRAIS	Phosphoribosylaminoimidazole synthase	(Ishi_283)	Purine Metabolism
PRASCSi	Phosphoribosylaminoimidazolesuccinocarboxamide synthase	(Ishi_896)	Purine Metabolism
PRFGS	Phosphoribosylformylglycinamidine synthase	(Ishi_893)	Purine Metabolism

PROTRS	Prolyl-tRNA synthetase	(Ishi_283)	Arginine and Proline Metabolism
PROabcpp	L-proline transport via ABC system (periplasm)	(Ishi_283)	Arginine and Proline Metabolism
PRPPS	Phosphoribosylpyrophosphate synthetase	(Ishi_283)	Ribose Metabolism
PSCVT	3-phosphoshikimate 1-carboxyvinyltransferase	(Ishi_357)	Phenylalanine Tyrosine Tryptophan Biosynthesis
PSD160	Phosphatidylserine decarboxylase (n-C16:0)	(Ishi_657)	Glycerophospholipid Metabolism
PSD161	Phosphatidylserine decarboxylase (n-C16:1)	(Ishi_657)	Glycerophospholipid Metabolism
PSP_L	Phosphoserine phosphatase (L-serine)	(Ishi_881)	Glycine, Serine and threonine metabolism
PTPATi	Pantetheine-phosphate adenylyltransferase	(Ishi_56)	Pantothenate and CoA Biosynthesis
PTRCabcpp	Putrescine transport via ABC system (periplasm)	(Ishi_283)	Transport
PYK	Pyruvate kinase	(Ishi_339)	Gluconeogenesis
RBFK	Riboflavin kinase	(Ishi_760)	Riboflavin Metabolism
RHACOAR100	RHACOAR100	(Ishi_702)	PHAs Metabolism
RHACOAR120	RHACOAR120	(Ishi_702)	PHAs Metabolism
RHACOAR140	RHACOAR140	(Ishi_702)	PHAs Metabolism
RHACOAR60	RHACOAR60	(Ishi_702)	PHAs Metabolism
RHACOAR80	RHACOAR80	(Ishi_702)	PHAs Metabolism
RNDR1	Ribonucleoside-diphosphate reductase (ADP)	(Ishi_734) and (Ishi_887) and (Ishi_759)	Purine Metabolism
RNDR4	Ribonucleoside-diphosphate reductase (UDP)	(Ishi_148)	Pyrimidine Metabolism
RPE	Ribulose 5-phosphate 3-epimerase	(Ishi_898)	Pentose Phosphate Pathway
RPI	Ribose-5-phosphate isomerase	(Ishi_985)	Pentose Phosphate Pathway
SDPDS	Succinyl-diaminopimelate desuccinylase	((Ishi_984) or (Ishi_984))	Lysine Biosynthesis
SDPTA	Succinyldiaminopimelate transaminase	(Ishi_577) or (Ishi_578)	Lysine Biosynthesis
SELMETAT	Selenomethionine adenosyltransferase	(Ishi_319)	Methionine Metabolism
SERAT	Serine O-acetyltransferase	(Ishi_547)	Cysteine Metabolism
SERTRS	Seryl-tRNA synthetase	(Ishi_693)	Glycine, Serine and threonine metabolism
SHCHF	Sirohydrochlorin ferrochelatase	(Ishi_622)	Porphyrin and Chlorophyll Metabolism

SOTA	Succinylornithine transaminase	(Ishi_731)	Arginine and Proline Metabolism
SPMDabcpp	Spermidine transport via ABC system (periplasm)	(Ishi_294)	Transport
SUCD1	Succinate dehydrogenase	(Ishi_975) and (Ishi_976) and (Ishi_974)	TCA Cycle
SUCDi	Succinate dehydrogenase (irreversible)	(Ishi_975) and (Ishi_976) and (Ishi_974)	TCA Cycle
SUCOAS	Succinyl-CoA synthetase (ADP-forming)	(Ishi_111)	TCA Cycle
SULabcpp	Sulfate transport via ABC system (periplasm)	(Ishi_809)	Sulfur Metabolism
T2DECAI	Trans-2-decenoyl-ACP isomerase	((Ishi_244) or (Ishi_244))	Fatty Acid Biosynthesis
TAURabcpp	Taurine transport via ABC system (periplasm)	(Ishi_47)	Transport
NADTRHD	NAD transhydrogenase	(Ishi_553)) or (Ishi_984) or ((Ishi_986)	Nicotinate and Nicotinamide
THRS	Threonine synthase	(Ishi_612)	Glycine, Serine and threonine metabolism
THRTRS	Threonyl-tRNA synthetase	(Ishi_612)	Glycine, Serine and threonine metabolism
TKT1	Transketolase	(Ishi_84)	Pentose Phosphate Pathway
TPI	Triose-phosphate isomerase	(Ishi_74)	Gluconeogenesis
TRDR	Thioredoxin reductase (NADPH)	(Ishi_781)	Pyrimidine Metabolism
TSULabcpp	Thiosulfate transport via ABC system (periplasm)	(Ishi_677) or (Ishi_230)	Sulfur Metabolism
UAGDP	UDP-N-acetylglucosamine diphosphorylase	(Ishi_862)	Aminosugar Metabolism
UAGPT3	UDP-N-acetylglucosamine-N-acetylmuramyl-(pentapeptide)pyrophosphoryl-undecaprenol N-acetylglucosamine transferase	(Ishi_1030)	Peptidoglycan Biosynthesis
UAMAGS	UDP-N-acetylmuramoyl-L-alanyl-D-glutamate synthetase	(Ishi_1032)	Glutamate Metabolism
UAMAS	UDP-N-acetylmuramoyl-L-alanine synthetase	(Ishi_303)	Glutamate metabolism
UDCPDP	Undecaprenyl-diphosphatase	(Ishi_641)	Peptidoglycan Biosynthesis
UGMDDS	UDP-N-acetylmuramoyl-L-alanyl-D-glutamyl-meso-2,6-diaminopimeloyl-D-alanyl-D-alanine synthetase	(Ishi_1034)	Peptidoglycan Biosynthesis
UMPK	UMP kinase	(Ishi_449)	Pyrimidine Metabolism

UPP3MT	Uroporphyrinogen methyltransferase	(Ishi_622)	Porphyrin and Chlorophyll Metabolism
UPPDC1	Uroporphyrinogen decarboxylase (uroporphyrinogen III)	(Ishi_413)	Porphyrin and Chlorophyll Metabolism
VALTRS	Valyl-tRNA synthetase	(Ishi_851)	Valine, Leucine, and Isoleucine Metabolism
pentso3abcpp	Pentso3abcpp	(Ishi_638) or ((Ishi_677) or (Ishi_230)) or (Ishi_638)	Transport
ACOLIPAabctex	Arabinose modified core oligosaccharide lipid A transport via ABC system (periplasm to extracellular)	((Ishi_286) and (Ishi_485)) or (Ishi_699) or (Ishi_677) or (Ishi_9) or (Ishi_525)	Lipopolysaccharide Biosynthesis / Recycling
ALATA_D2	D-alanine transaminase	(Ishi_240)	Cofactor and Prosthetic Group Biosynthesis
ALATA_L2	Alanine transaminase	(Ishi_240)	Cofactor and Prosthetic Group Biosynthesis
AMPTASECG	Alanyl aminopeptidase (cys-gly)	(Ishi_285)	Cysteine Metabolism
AMPTASEPG	Aminopeptidase (pro-gly)	(Ishi_285)	Arginine and Proline Metabolism
ASCBptspp	L-ascorbate transport via PEP:Pyr PTS (periplasm)	(Ishi_1107)	Transport, Inner Membrane
BUTSO3abcpp	Butanesulfonate transport via ABC system (periplasm)	((Ishi_637) and (Ishi_638) and (Ishi_770)) or (Ishi_230) or (Ishi_638) or ((Ishi_677) and (Ishi_231))	Transport, Inner Membrane
CLIPAabctex	Cold lipid A transport via ABC system (periplasm to extracellular)	(Ishi_501) and (Ishi_286) and (Ishi_485) and (Ishi_484) and (Ishi_288) and (Ishi_344)	Lipopolysaccharide Biosynthesis / Recycling
COLIPAPabctex	Core oligosaccharide lipid A diphosphate transport via ABC system (periplasm to extracellular)	((Ishi_286) and (Ishi_484) and (Ishi_485))	Lipopolysaccharide Biosynthesis / Recycling
COLIPAabctex	Core oligosaccharide lipid A transport via ABC system (periplasm to extracellular)	((Ishi_286) and (Ishi_484) and (Ishi_485) and (Ishi_699))	Lipopolysaccharide Biosynthesis / Recycling
CRNDabcpp	D-carnitine transport via ABC system (periplasm)	((Ishi_230) and (Ishi_231)	Transport, Inner Membrane
CYSSADS	L-cysteine sulfinic acid desulfurase	(Ishi_993))	Cysteine Metabolism
ECA4COLIPAabctex	Enterobacterial common antigen (x4) core oligosaccharide lipid A transport via ABC system (periplasm to extracellular)	((Ishi_484) and (Ishi_501) and (Ishi_485) and (Ishi_286))	Lipopolysaccharide Biosynthesis / Recycling
ENLIPAabctex	Phosphoethanolamine lipid A transport via ABC system (periplasm to extracellular)	((Ishi_485) and (Ishi_286) and (Ishi_484))	Lipopolysaccharide Biosynthesis / Recycling

FHL	Formate-hydrogen lyase	(Ishi_772) and (Ishi_771) and (Ishi_775) and (Ishi_765) and (Ishi_772) and (Ishi_776) and (Ishi_767) or (Ishi_777)	Pyruvate Metabolism
GRXR	Glutaredoxin reductase	((Ishi_981) and (Ishi_891))	Unassigned
HYD1pp	Hydrogenase (ubiquinone-8: 2 protons) (periplasm)	(Ishi_765) and (Ishi_772) and (Ishi_767) and (Ishi_766) and (Ishi_771) and (Ishi_775) and (Ishi_776) and (Ishi_777)	Oxidative Phosphorylation
K2L4Aabctex	KDO(2)-lipid IV A transport via ABC system (periplasm to extracellular)	(Ishi_834)	Lipopolysaccharide Biosynthesis / Recycling
KDOPS	3-deoxy -D-manno-octulosonic -acid 8-phosphate synthase	(Ishi_560)	Lipopolysaccharide Biosynthesis / Recycling
LIPAabctex	Lipid A transport via ABC system (periplasm to extracellular)	(Ishi_834)	Lipopolysaccharide Biosynthesis / Recycling
NADH17pp	NADH dehydrogenase (menaquinone-8 & 3 protons) (periplasm)	(Ishi_764) and (Ishi_765) and (Ishi_766) and (Ishi_767) and (Ishi_768) and (Ishi_769) and (Ishi_770) and (Ishi_771) and (Ishi_772) and (Ishi_773) and (Ishi_774) and (Ishi_775) and (Ishi_776) and (Ishi_777)	Oxidative Phosphorylation
NADH18pp	NADH dehydrogenase (demethylmenaquinone-8 & 3 protons) (periplasm)	(Ishi_764) and (Ishi_765) and (Ishi_766) and (Ishi_767) and (Ishi_768) and (Ishi_769) and (Ishi_770) and (Ishi_771) and (Ishi_772) and (Ishi_773) and (Ishi_774) and (Ishi_775) and (Ishi_776) and (Ishi_777)	Oxidative Phosphorylation
NADHHR	NADH hydratase	(Ishi_579)	Metabolite Repair
NADHHS	NADH hydratase	(Ishi_579)	Metabolite Repair
NADPHHR	NADPH hydratase	(Ishi_579)	Metabolite Repair
NADPHHS	NADPH hydratase	(Ishi_579)	Metabolite Repair
O16A4COLIPAabctex	O16 antigen (x4) core oligosaccharide lipid A transport via ABC system (periplasm to extracellular)	(Ishi_483) and (Ishi_484) and (Ishi_485)	Lipopolysaccharide Biosynthesis / Recycling
OCTDPS	Octaprenyl pyrophosphate synthase	(Ishi_473)	Cofactor and Prosthetic Group Biosynthesis
OMBZLM	2-Octaprenyl-6-methoxy-benzoquinol methylase	(Ishi_473)	Cofactor and Prosthetic Group Biosynthesis
RNDR4b	Ribonucleoside-diphosphate reductase (UDP) (glutaredoxin)	(Ishi_462)	Nucleotide Salvage Pathway
S2FE2SR	SUF [2Fe-2S] regeneration	(Ishi_990) and (Ishi_991) and (Ishi_992)	Cofactor and Prosthetic Group Biosynthesis

S2FE2SS	SUF [2Fe-2S] Synthesis	(Ishi_990) and (Ishi_991) and (Ishi_992)	Cofactor and Prosthetic Group Biosynthesis
S2FE2SS2	SUF [2Fe-2S] Synthesis II	(Ishi_990) and (Ishi_991) and (Ishi_992)	Cofactor and Prosthetic Group Biosynthesis
S2FE2ST	SUF [2Fe-2S] Transfer	(Ishi_990) and (Ishi_991) and (Ishi_992)	Cofactor and Prosthetic Group Biosynthesis
S4FE4SR	SUF [4Fe-4S] Reduction	(Ishi_991) and (Ishi_992) and (Ishi_990)	Cofactor and Prosthetic Group Biosynthesis
S4FE4ST	SUF [4Fe-4S] Transfer	(Ishi_990) and (Ishi_991) and (Ishi_992)	Cofactor and Prosthetic Group Biosynthesis
SCYSDS	SUF Cysteine desulfuration	(Ishi_993)	Cofactor and Prosthetic Group Biosynthesis
FEex	Iron transporter	((Ishi_834) and (Ishi_658)) or (Ishi_69)	iron metabolism
FEpp	Iron transporter	((Ishi_658) and (Ishi_834))	iron metabolism
Kt3pp	Potassium transport out via proton antiport (periplasm)	(Ishi_738)	Inorganic Ion Transport and Metabolism
2DHGLCNkt_tpp	2DHGLCNkt tpp	(VBICanLib64949_0763)	Transport
4HTHRS	4-Hydroxy-L-threonine synthase	(VBICanLib64949_0372)	Vitamin B6 Metabolism
ACCOAC	Acetyl-CoA carboxylase	((VBICanLib64949_0413) and (VBICanLib64949_0158) and (VBICanLib64949_0656) and (VBICanLib64949_0159))	Fatty Acid Biosynthesis
ACGS	N-acetylglutamate synthase	(VBICanLib64949_0394) or (VBICanLib64949_0221)	Urea cycle/amino group metabolism
ACHBS	2-aceto-2-hydroxybutanoate synthase	(VBICanLib64949_0019)	Valine, Leucine, and Isoleucine Metabolism
ACOATA	Acetyl-CoA ACP transacylase	((VBICanLib64949_0712) and (VBICanLib64949_0745))	Fatty Acid Biosynthesis
ACONT	Aconitate hydratase	(VBICanLib64949_0356)	TCA Cycle
ACONTa	Aconitase (half-reaction A, Citrate hydro-lyase)	(VBICanLib64949_0356)	TCA Cycle
ACONTb	Aconitase (half-reaction B, Isocitrate hydro-lyase)	(VBICanLib64949_0356)	TCA Cycle
ADSL1r	Adenylsuccinate lyase	(VBICanLib64949_0903)	Purine Metabolism
ADSL2r	Adenylosuccinate lyase	(VBICanLib64949_0903) or (VBICanLib64949_0077) or (VBICanLib64949_0272)	Purine Metabolism
AGPAT120	1-tetradecanoyl-sn-glycerol 3-phosphate O-acyltransferase (n-C12:0)	(VBICanLib64949_0664)	Glycerophospholipid Metabolism
AGPAT160	1-hexadecanoyl-sn-glycerol 3-phosphate O-acyltransferase (n-C16:0)	(VBICanLib64949_0664)	Glycerophospholipid Metabolism
AGPAT161	1-hexadec-7-enoyl-sn-glycerol 3-phosphate O-acyltransferase (n-C16:1)	(VBICanLib64949_0664)	Glycerophospholipid Metabolism

AGPAT180	1-octadecanoyl-sn-glycerol 3-phosphate O-acyltransferase (n-C18:0)	(VBICanLib64949_0664)	Glycerophospholipid Metabolism
AGPAT181	1-octadec-7-enoyl-sn-glycerol 3-phosphate O-acyltransferase (n-C18:1)	(VBICanLib64949_0664)	Glycerophospholipid Metabolism
AKGDH	2-Oxogluterate dehydrogenase	((VBICanLib64949_0988) and (VBICanLib64949_0986) and (VBICanLib64949_0987))	
ALAALAr	D-alanine-D-alanine ligase (reversible)	(VBICanLib64949_11176)	Peptidoglycan Biosynthesis
ANPRT	Anthranilate phosphoribosyltransferase	(VBICanLib64949_0317)	Phenylalanine Tyrosine Tryptophan Biosynthesis
ANS	Anthranilate synthase	(VBICanLib64949_0317)	Phenylalanine Tyrosine Tryptophan Biosynthesis
ARGSL	Argininosuccinate lyase	(VBICanLib64949_0272)	Arginine and Proline Metabolism
ARGSS	Argininosuccinate synthase	(VBICanLib64949_0124)	Arginine and Proline Metabolism
ARGabcpp	L-arginine transport via ABC system (periplasm)	((VBICanLib64949_1047) and (VBICanLib64949_0058)) or ((VBICanLib64949_1046) and (VBICanLib64949_1047) and (VBICanLib64949_0058) and (VBICanLib64949_0057))	Transport
ASAD	Aspartate-semialdehyde dehydrogenase	(VBICanLib64949_0200)	Lysine Biosynthesis
ASNS1	Asparagine synthase (glutamine-hydrolysing)	(VBICanLib64949_0234)	Alanine and Aspartate Metabolism
ASPabcpp	L-aspartate transport via ABC system (periplasm)	((VBICanLib64949_0057) and (VBICanLib64949_0056) and (VBICanLib64949_1046)) or (VBICanLib64949_1047)	Transport
ATPPRT	ATP phosphoribosyltransferase	(VBICanLib64949_0372)	Histidine Metabolism
ATPS4rpp	ATP synthase (four protons for one ATP) (periplasm)	((VBICanLib64949_0626) and (VBICanLib64949_0629) and (VBICanLib64949_1023) and (VBICanLib64949_0627) and (VBICanLib64949_0625) and (VBICanLib64949_0628))	Oxidative Phosphorylation
BZt1pp	Benzoate transport in via proton symport (periplasm)	(VBICanLib64949_0289)	B-Ketoadipate pathway

CHLabcpp	Choline transport via ABC system (periplasm)	((VBICanLib64949_0240) and (VBICanLib64949_0239)) or (VBICanLib64949_0538) or (VBICanLib64949_0648) or (VBICanLib64949_0686)	Transport
CHOLS_ex	CHOLS LPAREN ex RPAREN	(VBICanLib64949_0372)	Transport
DAPE	Diaminopimelate epimerase	(VBICanLib64949_0531)	Lysine Biosynthesis
CHOLSabc	CHOLSabc	((VBICanLib64949_0241) and (VBICanLib64949_0240) and (VBICanLib64949_0239)) or (VBICanLib64949_0538) or (VBICanLib64949_0648) or (VBICanLib64949_0686)	Glycine, Serine and threonine metabolism
CITtex	Citrate transport via diffusion (extracellular to periplasm)	(VBICanLib64949_0828)	Transport
COBALT2tex	Cobalt (Co+2) transport via diffusion (extracellular to periplasm)	(VBICanLib64949_0647)	Transport
COBALT2tpp	Cobalt transport in via permease (no H+)	(VBICanLib64949_0647)	Transport
CS	Citrate synthase	((VBICanLib64949_0239) and (VBICanLib64949_0240))	TCA Cycle
CTPS2	CTP synthase (glutamine)	((VBICanLib64949_0239) and (VBICanLib64949_0240))	Pyrimidine Metabolism
CYO1b	CYO1b	((VBICanLib64949_0381) and (VBICanLib64949_0382))	Oxidative Phosphorylation
CYSabcpp	L-cysteine uptake via ABC system (periplasm)	((VBICanLib64949_0712) and (VBICanLib64949_0745))	Transport
DAPDC	Diaminopimelate decarboxylase	(VBICanLib64949_0273) or (VBICanLib64949_0273)	Lysine Biosynthesis
DASYN120	CDP-diacylglycerol synthetase (n-C12:0)	(VBICanLib64949_0461)	Glycerophospholipid Metabolism
DASYN160	CDP-diacylglycerol synthetase (n-C16:0)	(VBICanLib64949_0461)	Glycerophospholipid Metabolism
DASYN161	CDP-diacylglycerol synthetase (n-C16:1)	(VBICanLib64949_0461)	Glycerophospholipid Metabolism
DASYN180	CDP-diacylglycerol synthetase (n-C18:0)	(VBICanLib64949_0461)	Glycerophospholipid Metabolism

DASYN181	CDP-diacylglycerol synthetase (n-C18:1)	(VBICanLib64949_0461)	Glycerophospholipid Metabolism
DB4PS	3,4-Dihydroxy-2-butanone-4-phosphate synthase	(VBICanLib64949_0261)	Riboflavin Metabolism
DDPA	3-deoxy-D-arabino-heptulosonate 7-phosphate synthetase	(VBICanLib64949_0573)	Phenylalanine Tyrosine Tryptophan Biosynthesis
DHPM1	Dihydropyrimidinase (5,6-dihydrouracil)	(VBICanLib64949_0195)	Pantothenate and CoA Biosynthesis
DMATT	Dimethylallyltranstransferase	(VBICanLib64949_1045) or (VBICanLib64949_0484)	Steroid biosynthesis
DNAMTn2r	DNA (cytosine-5-)-methyltransferase	(VBICanLib64949_0729) or (VBICanLib64949_0728)	Methionine Metabolism
DNTPPA	Dihydroneopterin triphosphate pyrophosphatase	(VBICanLib64949_0220)	Folate Biosynthesis
DPCOAK	Dephospho-CoA kinase	(VBICanLib64949_0415)	Pantothenate and CoA Biosynthesis
DPR	2-dehydropantoate 2-reductase	(VBICanLib64949_1033)	Pantothenate and CoA Biosynthesis
DTMPK	DTMP kinase	(VBICanLib64949_0107)	Pyrimidine Metabolism
DURAD	Dihydrouracil dehydrogenase (NADP)	(VBICanLib64949_0338)	Pantothenate and CoA Biosynthesis
DUTPDP	DUTP diphosphatase	(VBICanLib64949_0960)	Pyrimidine Metabolism
DXPS	1-deoxy-D-xylulose 5-phosphate synthase	(VBICanLib64949_0569)	Steroid biosynthesis
EAR100y	Enoyl-[acyl-carrier-protein] reductase (NADPH) (n-C10:0)	(VBICanLib64949_0369) or (VBICanLib64949_1104)	Fatty Acid Biosynthesis
EAR120y	Enoyl-[acyl-carrier-protein] reductase (NADPH) (n-C12:0)	((VBICanLib64949_0239) and (VBICanLib64949_0240))	Fatty Acid Biosynthesis
EAR121y	Enoyl-[acyl-carrier-protein] reductase (NADPH) (n-C12:1)	(VBICanLib64949_0239) and (VBICanLib64949_0240)	Fatty Acid Biosynthesis
EAR160y	Enoyl-[acyl-carrier-protein] reductase (NADPH) (n-C16:0)	((VBICanLib64949_0538) and (VBICanLib64949_0240)) or (VBICanLib64949_0239)	Fatty Acid Biosynthesis
EAR161y	Enoyl-[acyl-carrier-protein] reductase (NADPH) (n-C16:1)	(VBICanLib64949_0369) or (VBICanLib64949_1104)	Fatty Acid Biosynthesis
EAR180y	Enoyl-[acyl-carrier-protein] reductase (NADPH) (n-C18:0)	(VBICanLib64949_0369) or (VBICanLib64949_1104)	Fatty Acid Biosynthesis
EAR181y	Enoyl-[acyl-carrier-protein] reductase (NADPH) (n-C18:1)	(VBICanLib64949_0369) or (VBICanLib64949_1104)	Fatty Acid Biosynthesis

EAR40y	Enoyl-[acyl-carrier-protein] reductase (NADPH) (n-C4:0)	(VBICanLib64949_0369) or (VBICanLib64949_1104)	Fatty Acid Biosynthesis
EAR60y	Enoyl-[acyl-carrier-protein] reductase (NADPH) (n-C6:0)	(VBICanLib64949_0369) or (VBICanLib64949_1104)	Fatty Acid Biosynthesis
EAR80y	Enoyl-[acyl-carrier-protein] reductase (NADPH) (n-C8:0)	(VBICanLib64949_0369) or (VBICanLib64949_1104)	Fatty Acid Biosynthesis
ETHSO3abcpp	Ethanesulfonate transport via ABC system (periplasm)	((VBICanLib64949_0648) and (VBICanLib64949_0647) and (VBICanLib64949_0776))	Transport
FDH	Formate dehydrogenase	((VBICanLib64949_0776) and (VBICanLib64949_0775) and (VBICanLib64949_0774))	Methane Metabolism
FE2abc	Iron (II) transport via ABC system	(VBICanLib64949_0239)) or ((VBICanLib64949_0686) and (VBICanLib64949_0685) and (VBICanLib64949_0681))	Transport
FTHFD	Formyltetrahydrofolate deformylase	(VBICanLib64949_0588)	One Carbon pool by folate
FUM	Fumarase	((VBICanLib64949_0131) or (VBICanLib64949_0292))	TCA Cycle
FUMtex	Fumarate transport via diffusion (extracellular to periplasm)	(VBICanLib64949_0077)	Transport
G1SAT	Glutamate-1-semialdehyde aminotransferase	(VBICanLib64949_0336)	Porphyrin and Chlorophyll Metabolism
G3PAT120	Glycerol-3-phosphate acyltransferase (C12:0)	(VBICanLib64949_0712)	Glycerophospholipid Metabolism
G3PAT160	Glycerol-3-phosphate acyltransferase (C16:0)	(VBICanLib64949_0712)	Glycerophospholipid Metabolism
G3PAT161	Glycerol-3-phosphate acyltransferase (C16:1)	(VBICanLib64949_0712)	Glycerophospholipid Metabolism
G3PAT180	Glycerol-3-phosphate acyltransferase (C18:0)	(VBICanLib64949_0712)	Glycerophospholipid Metabolism
G3PAT181	Glycerol-3-phosphate acyltransferase (C18:1)	(VBICanLib64949_0712)	Glycerophospholipid Metabolism
GART	GAR transformylase-T	(VBICanLib64949_0041)	Purine Metabolism
GLCNt2rpp	D-gluconate transport via proton symport, reversible (periplasm)	(VBICanLib64949_0250)	Transport

GLCabcpp	D-glucose transport via ABC system (periplasm)	(VBICanLib64949_0058) or (VBICanLib64949_0239) or (VBICanLib64949_0686)	Transport
GLNS	Glutamine synthetase	((VBICanLib64949_1046) and (VBICanLib64949_1047) and (VBICanLib64949_0058)) or (VBICanLib64949_0648)	Glutamate Metabolism
GLNTRAT	Glutamyl-tRNA(Gln) amidotransferase	((VBICanLib64949_0907) and (VBICanLib64949_0909) and (VBICanLib64949_0908))	Glutamate Metabolism
GLUSy	Glutamate synthase (NADPH)	(VBICanLib64949_0175)	Glutamate Metabolism
GLUt4pp	Na+/glutamate symport (periplasm)	(VBICanLib64949_0894)	Transport
GLXCL	Glyoxalate carboligase	(VBICanLib64949_0189) or (VBICanLib64949_0570)	Glycolate metabolism
GLYALDtex	Glyceraldehyde transport via diffusion (extracellular to periplasm)	(VBICanLib64949_0402) or (VBICanLib64949_0057)	Transport
GLYBabcpp	Glycine betaine transport via ABC system (periplasm)	(VBICanLib64949_0289)	Arginine and Proline Metabolism
GLYCK	Glycerate kinase	(VBICanLib64949_0837)	Glycine, Serine and threonine metabolism
GLYCL	Glycine Cleavage System	(VBICanLib64949_0566)) or (VBICanLib64949_0988) or (VBICanLib64949_0990)	Glycine, Serine and threonine metabolism
GLYCLTt2rpp	Glycolate transport via proton symport, reversible (periplasm)	(VBICanLib64949_0566)) or (VBICanLib64949_0988) or (VBICanLib64949_0990)	Transport
GLYCLTtex	Glycolate transport via diffusion (extracellular to periplasm)	(VBICanLib64949_0566)) or (VBICanLib64949_0988) or (VBICanLib64949_0990)	Transport
GLYCL_2	Glycine cleavage system, cytosol	(VBICanLib64949_0566)) or (VBICanLib64949_0988) or (VBICanLib64949_0990)	Nitrogen Metabolism
GLYCTO1	Glycolate oxidase	(VBICanLib64949_0478)	Glycolate metabolism
GLYTRS	Glycyl-tRNA synthetase	((VBICanLib64949_0058) and (VBICanLib64949_0240))	Glycine, Serine and threonine metabolism
GMPS2	GMP synthase	((VBICanLib64949_1028) and (VBICanLib64949_0482))	Purine Metabolism

GND	Phosphogluconate dehydrogenase	(VBICanLib64949_0525) or (VBICanLib64949_0292)	Pentose Phosphate Pathway
GRTT	Geranyltranstransferase	(VBICanLib64949_1045) or (VBICanLib64949_0484)	Steroid biosynthesis
GTHPi	Glutathione peridoxase	(VBICanLib64949_1154)	Glutathione Metabolism
GTHS	Glutathione synthetase	(VBICanLib64949_0547)	Glutamate Metabolism
GTPCII	GTP cyclohydrolase II	(VBICanLib64949_0261)	Riboflavin Metabolism
HCO3_NAt	Bicarbonate transport (Na/HCO3 cotransport)	(VBICanLib64949_0845)	Transport
HEMEOS	Heme O synthase	(VBICanLib64949_0262)	Porphyrin and Chlorophyll Metabolism
HMBS	Hydroxymethylbilane synthase	(VBICanLib64949_0972)	Porphyrin and Chlorophyll Metabolism
HMGL	Hydroxymethylglutaryl-CoA lyase	(VBICanLib64949_0081)	Synthesis and degradation of Ketones Bodies
HPPK	2-amino-4-hydroxy-6-hydroxymethyldihydropteridine diphosphokinase	(VBICanLib64949_1086)	Folate Biosynthesis
HXAtex	Hexanoate transport via diffusion (extracellular to periplasm)	(VBICanLib64949_0845)	Transport
ICDHyr	Isocitrate dehydrogenase (NADP)	(VBICanLib64949_0822)	TCA Cycle
ICITtex	Citrate transport via diffusion extracellular to periplasm	(VBICanLib64949_0896)	Transport
ICL	Isocitrate lyase	(VBICanLib64949_0386)	TCA Cycle
IGPS	Indole-3-glycerol-phosphate synthase	(VBICanLib64949_0019)	Phenylalanine Tyrosine Tryptophan Biosynthesis
ILEabcpp	L-isoleucine transport via ABC system (periplasm)	(VBICanLib64949_0896)	Transport
IPDDI	Isopentenyl-diphosphate D-isomerase	(VBICanLib64949_1010)	Steroid biosynthesis
IPDPS	1-hydroxy-2-methyl-2-(E)-butenyl 4-diphosphate reductase (ipdp)	(VBICanLib64949_0965)	Steroid biosynthesis
IPMD	3-isopropylmalate dehydrogenase	(VBICanLib64949_0822)	Valine, Leucine, and Isoleucine Metabolism
IPPMIa	3-isopropylmalate dehydratase	(VBICanLib64949_0356) or (VBICanLib64949_0742)	Valine, Leucine, and Isoleucine Metabolism
IPPMIb	2-isopropylmalate hydratase	(VBICanLib64949_0356) or (VBICanLib64949_0742)	Valine, Leucine, and Isoleucine Metabolism
KARA1	Ketol-acid reductoisomerase (2,3-dihydroxy-3-methylbutanoate)	(VBICanLib64949_1167)	Valine, Leucine, and Isoleucine Metabolism
KARA2	Ketol-acid reductoisomerase (2-Acetolactate)	(VBICanLib64949_1167)	Valine, Leucine, and Isoleucine Metabolism

KAS14	Beta-ketoacyl-ACP synthase	(VBICanLib64949_0368)	Fatty Acid Biosynthesis
LEUTRS	Leucyl-tRNA synthetase	(VBICanLib64949_0432)	Valine, Leucine, and Isoleucine Metabolism
LEUabcpp	L-leucine transport via ABC system (periplasm)	(VBICanLib64949_0896) and (VBICanLib64949_1093)	Transport
LYSTRS	Lysyl-tRNA synthetase	(VBICanLib64949_1052)	Lysine Biosynthesis
LYSabcpp	L-lysine transport via ABC system (periplasm)	(VBICanLib64949_0058)	Lysine Metabolism
MACPD	Malonyl-ACP decarboxylase	(VBICanLib64949_0058)	Fatty Acid Biosynthesis
MSO3abcpp	Methanesulfonate transport via ABC system (periplasm)	(VBICanLib64949_0647)	Transport
MTHFC	Methenyltetrahydrofolate cyclohydrolase	(VBICanLib64949_0737)	One Carbon pool by folate
MTHFD	Methylenetetrahydrofolate dehydrogenase (NADP)	(VBICanLib64949_0737)	One Carbon pool by folate
MHPGLUT	5 methyltetrahydropteroyltriglutamate homocysteine S methyltransferase	(VBICanLib64949_1018)	Methionine Metabolism
NADH16pp	NADH dehydrogenase (ubiquinone-8 & 3 protons) (periplasm)	(VBICanLib64949_0770) and (VBICanLib64949_0771) and (VBICanLib64949_0772) and (VBICanLib64949_0773) and (VBICanLib64949_0774) and (VBICanLib64949_0775) and (VBICanLib64949_0776) and (VBICanLib64949_0777) and (VBICanLib64949_0778) and (VBICanLib64949_0779) and (VBICanLib64949_0780) and (VBICanLib64949_0781) and (VBICanLib64949_0782) and (VBICanLib64949_0783)	Oxidative Phosphorylation
NADK	NAD kinase	(VBICanLib64949_0485)	Nicotinate and Nicotinamide
NDPK1	Nucleoside-diphosphate kinase (ATP:GDP)	(VBICanLib64949_0292)	Purine Metabolism
NDPK2	Nucleoside-diphosphate kinase (ATP:UDP)	(VBICanLib64949_0292)	Pyrimidine Metabolism

NDPK3	Nucleoside-diphosphate kinase (ATP:CDP)	(VBICanLib64949_0292)	Pyrimidine Metabolism
NDPK4	Nucleoside-diphosphate kinase (ATP:dTDP)	(VBICanLib64949_0292)	Pyrimidine Metabolism
NDPK5	Nucleoside-diphosphate kinase (ATP:dGDP)	(VBICanLib64949_0292)	Purine Metabolism
NDPK6	Nucleoside-diphosphate kinase (ATP:dUDP)	(VBICanLib64949_0292)	Pyrimidine Metabolism
NDPK7	Nucleoside-diphosphate kinase (ATP:dCDP)	(VBICanLib64949_0292)	Pyrimidine Metabolism
NDPK8	Nucleoside-diphosphate kinase (ATP:dADP)	(VBICanLib64949_0292)	Purine Metabolism
NNATr	Nicotinate-nucleotide adenylyltransferase	(VBICanLib64949_0594)	Nicotinate and Nicotinamide
NO2t2rpp	Nitrite transport in via proton symport, reversible (periplasm)	(VBICanLib64949_0920)	Transport
OIVD1r	2-oxoisovalerate dehydrogenase (acylating; 4-methyl-2-oxopentaoate)	(VBICanLib64949_0986)	Valine, leucine and isoleucine degradation
OIVD2	2-oxoisovalerate dehydrogenase (acylating; 3-methyl-2-oxobutanoate)	(VBICanLib64949_0986)	Valine, leucine and isoleucine degradation
OIVD3	2-oxoisovalerate dehydrogenase (acylating; 3-methyl-2-oxopentanoate)	(VBICanLib64949_0986)	Valine, leucine and isoleucine degradation
ORNCD	Ornithine cyclodeaminase	(VBICanLib64949_1160) or (VBICanLib64949_0975)	Arginine and Proline Metabolism
ORNabcpp	Ornithine transport via ABC system (periplasm)	(VBICanLib64949_0058)	Lysine Metabolism
ORPT	Orotate phosphoribosyltransferase	(VBICanLib64949_0946)	Pyrimidine Metabolism
PANTS	Pantothenate synthase	(VBICanLib64949_0621)	Pantothenate and CoA Biosynthesis
PDH	Pyruvate dehydrogenase	(VBICanLib64949_0566)	Glycolysis
PDX5POi	Pyridoxine 5-phosphate oxidase	(VBICanLib64949_1103)	Vitamin B6 Metabolism
PGM	Phosphoglycerate mutase	(VBICanLib64949_0170)	Gluconeogenesis
PGMT	Phosphoglucomutase	(VBICanLib64949_1041)	Glycolysis
PIuabcpp	Phosphate transport via ABC system (uptake, periplasm)	(VBICanLib64949_0537)	Transport
PMDPHT	Pyrimidine phosphatase	(VBICanLib64949_0050)	Riboflavin Metabolism
PPA	Inorganic diphosphatase	(VBICanLib64949_0122)	Oxidative Phosphorylation

PPBNGS	Porphobilinogen synthase	(VBICanLib64949_0253)	Porphyrin and Chlorophyll Metabolism
PPPGO	Protoporphyrinogen oxidase (aerobic)	(VBICanLib64949_1008)	Porphyrin and Chlorophyll Metabolism
PRAGSr	Phosphoribosylglycinamide synthase	(VBICanLib64949_0095)	Purine Metabolism
PRAIi	Phosphoribosylanthranilate isomerase (irreversible)	(VBICanLib64949_0019)	Phenylalanine Tyrosine Tryptophan Biosynthesis
PRFGS	Phosphoribosylformylglycinamidine synthase	(VBICanLib64949_0899)	Purine Metabolism
PSP_L	Phosphoserine phosphatase (L-serine)	(VBICanLib64949_0886)	Glycine, Serine and threonine metabolism
RPE	Ribulose 5-phosphate 3-epimerase	(VBICanLib64949_0904)	Pentose Phosphate Pathway
RPI	Ribose-5-phosphate isomerase	(VBICanLib64949_0989)	Pentose Phosphate Pathway
SDPTA	Succinyldiaminopimelate transaminase	(VBICanLib64949_0336)	Lysine Biosynthesis
SERAT	Serine O-acetyltransferase	(VBICanLib64949_0559)	Cysteine Metabolism
SERTRS	Seryl-tRNA synthetase	(VBICanLib64949_0562)	Glycine, Serine and threonine metabolism
SHCHF	Sirohydrochlorin ferrochelatase	(VBICanLib64949_0631)	Porphyrin and Chlorophyll Metabolism
SOTA	Succinylornithine transaminase	(VBICanLib64949_0336)	Arginine and Proline Metabolism
SUCD1	Succinate dehydrogenase	(VBICanLib64949_0978) and (VBICanLib64949_0979) and (VBICanLib64949_0977)	TCA Cycle
SULabcpp	Sulfate transport via ABC system (periplasm)	(VBICanLib64949_0811)	Sulfur Metabolism
TRSARr	Tartronate semialdehyde reductase	(VBICanLib64949_0525)	Glycolate metabolism
TSULabcpp	Thiosulfate transport via ABC system (periplasm)	(VBICanLib64949_0686) or (VBICanLib64949_0239)	Sulfur Metabolism
UDCPDP	Undecaprenyl-diphosphatase	(VBICanLib64949_0651)	Peptidoglycan Biosynthesis
UMPK	UMP kinase	(VBICanLib64949_045)	Pyrimidine Metabolism
UPPN	B-ureidopropionase	(VBICanLib64949_1031)	Pantothenate and CoA Biosynthesis
UREA	Urease	(VBICanLib64949_0803)	Urea cycle/amino group metabolism
VALTRS	Valyl-tRNA synthetase	(VBICanLib64949_0854)	Valine, Leucine, and Isoleucine Metabolism
VANKpp	VANKpp	(VBICanLib64949_0896)	Transport
VANLNtex	VANLNtex	(VBICanLib64949_0896)	Transport
VANLTtex	VANLTtex	(VBICanLib64949_0896)	Transport
VNLNpp	VNLNpp	(VBICanLib64949_0896)	Transport
pentso3abcpp	Pentso3abcpp	(VBICanLib64949_0648)	Transport

ACOLIPAabctex	Arabinose modified core oligosaccharide lipid A transport via ABC system (periplasm to extracellular)	(VBICanLib64949_0295) and (VBICanLib64949_0496) or (VBICanLib64949_0708) or (VBICanLib64949_0686) or (VBICanLib64949_0018) or (VBICanLib64949_0538)	Lipopolysaccharide Biosynthesis / Recycling
ALATA_D2	D-alanine transaminase	(VBICanLib64949_0249)	Cofactor and Prosthetic Group Biosynthesis
ALATA_L2	Alanine transaminase	(VBICanLib64949_0249)	Cofactor and Prosthetic Group Biosynthesis
AMPTASECG	Alanyl aminopeptidase (cys-gly)	(VBICanLib64949_0294)	Cysteine Metabolism
AMPTASEPG	Aminopeptidase (pro-gly)	(VBICanLib64949_0294)	Arginine and Proline Metabolism
ASCBptspp	L-ascorbate transport via PEP:Pyr PTS (periplasm)	((VBICanLib64949_1106) and (VBICanLib64949_0057) and (VBICanLib64949_0056) and (VBICanLib64949_1046) and (VBICanLib64949_1046))	Transport, Inner Membrane
BUTSO3abcpp	Butanesulfonate transport via ABC system (periplasm)	((VBICanLib64949_0648) and (VBICanLib64949_0647) and (VBICanLib64949_0240))	Transport, Inner Membrane
CLIPAabctex	Cold lipid A transport via ABC system (periplasm to extracellular)	((VBICanLib64949_0513) and (VBICanLib64949_0295) and (VBICanLib64949_0496) and (VBICanLib64949_0495) and (VBICanLib64949_0297) and (VBICanLib64949_0351))	Lipopolysaccharide Biosynthesis / Recycling
COLIPAPabctex	Core oligosaccharide lipid A diphosphate transport via ABC system (periplasm to extracellular)	((VBICanLib64949_0295) and (VBICanLib64949_0495) and (VBICanLib64949_0496))	Lipopolysaccharide Biosynthesis / Recycling
COLIPAabctex	Core oligosaccharide lipid A transport via ABC system (periplasm to extracellular)	((VBICanLib64949_0295) and (VBICanLib64949_0495) and (VBICanLib64949_0496))	Lipopolysaccharide Biosynthesis / Recycling
CRNDabcpp	D-carnitine transport via ABC system (periplasm)	((VBICanLib64949_0239) and (VBICanLib64949_0240))	Transport, Inner Membrane
CYSSADS	L-cysteine sulfinic acid desulfurase	((VBICanLib64949_1026) or (VBICanLib64949_0997))	Cysteine Metabolism

ECA4COLIPAabctex	Enterobacterial common antigen (x4) core oligosaccharide lipid A transport via ABC system (periplasm to extracellular)	((VBICanLib64949_0495) and (VBICanLib64949_0513) and (VBICanLib64949_0496) and (VBICanLib64949_0295))	Lipopolysaccharide Biosynthesis / Recycling
ENLIPAabctex	Phosphoethanolamine lipid A transport via ABC system (periplasm to extracellular)	((VBICanLib64949_0496) and (VBICanLib64949_0295) and (VBICanLib64949_0495))	Lipopolysaccharide Biosynthesis / Recycling
GRXR	Glutaredoxin reductase	(VBICanLib64949_0897)	Unassigned
K2L4Aabctex	KDO(2)-lipid IV A transport via ABC system (periplasm to extracellular)	(VBICanLib64949_0837)	Lipopolysaccharide Biosynthesis / Recycling
KAS15	Beta-ketoacyl-ACP synthase (2)	(VBICanLib64949_0713)	Membrane Lipid Metabolism
KDOPS	3-deoxy -D-manno-octulosonic -acid 8-phosphate synthase	(VBICanLib64949_0745)	Lipopolysaccharide Biosynthesis / Recycling
LIPAabctex	Lipid A transport via ABC system (periplasm to extracellular)	(VBICanLib64949_0496)	Lipopolysaccharide Biosynthesis / Recycling
NADH17pp	NADH dehydrogenase (menaquinone-8 & 3 protons) (periplasm)	(VBICanLib64949_0770) and (VBICanLib64949_0771) and (VBICanLib64949_0772) and (VBICanLib64949_0773) and (VBICanLib64949_0774) and (VBICanLib64949_0775) and (VBICanLib64949_0776) and (VBICanLib64949_0777) and (VBICanLib64949_0778) and (VBICanLib64949_0779) and (VBICanLib64949_0780) and (VBICanLib64949_0781) and (VBICanLib64949_0782) and (VBICanLib64949_0783)	Oxidative Phosphorylation

NADH18pp	NADH dehydrogenase (demethylmenaquinone-8 & 3 protons) (periplasm)	(VBICanLib64949_0770) and (VBICanLib64949_0771) and (VBICanLib64949_0772) and (VBICanLib64949_0773) and (VBICanLib64949_0774) and (VBICanLib64949_0775) and (VBICanLib64949_0776) and (VBICanLib64949_0777) and (VBICanLib64949_0778) and (VBICanLib64949_0779) and (VBICanLib64949_0780) and (VBICanLib64949_0781) and (VBICanLib64949_0782) and (VBICanLib64949_0783)	Oxidative Phosphorylation
NADHHR	NADH hydratase	(VBICanLib64949_0589)	Metabolite Repair
NADHHS	NADH hydratase	(VBICanLib64949_0589)	Metabolite Repair
NADPHHR	NADPH hydratase	(VBICanLib64949_0589)	Metabolite Repair
NADPHHS	NADPH hydratase	(VBICanLib64949_0589)	Metabolite Repair
O16A4COLIPAabctex	O16 antigen (x4) core oligosaccharide lipid A transport via ABC system (periplasm to extracellular)	(VBICanLib64949_0494) and (VBICanLib64949_0495) and (VBICanLib64949_0496)	Lipopolysaccharide Biosynthesis / Recycling
OCTDPS	Octaprenyl pyrophosphate synthase	(VBICanLib64949_0484)	Cofactor and Prosthetic Group Biosynthesis
OMBZLM	2-Octaprenyl-6-methoxy-benzoquinol methylase	(VBICanLib64949_0484)	Cofactor and Prosthetic Group Biosynthesis
RNDR3b	Ribonucleoside-diphosphate reductase (CDP) (glutaredoxin)	(VBICanLib64949_0741) and (VBICanLib64949_0014)) or (VBICanLib64949_0074) or (VBICanLib64949_0075) or (VBICanLib64949_0765) or (VBICanLib64949_0893) or ((VBICanLib64949_0939)	Nucleotide Salvage Pathway
RNDR4b	Ribonucleoside-diphosphate reductase (UDP) (glutaredoxin)	(VBICanLib64949_0471)	Nucleotide Salvage Pathway
S2FE2SR	SUF [2Fe-2S] regeneration	(VBICanLib64949_0928) and (VBICanLib64949_0996) and (VBICanLib64949_0997) and (VBICanLib64949_0995)	Cofactor and Prosthetic Group Biosynthesis

S2FE2SS	SUF [2Fe-2S] Synthesis	(VBICanLib64949_0928) and (VBICanLib64949_0996) and (VBICanLib64949_0997) and (VBICanLib64949_0995)	Cofactor and Prosthetic Group Biosynthesis
S2FE2SS2	SUF [2Fe-2S] Synthesis II	(VBICanLib64949_0928) and (VBICanLib64949_0996) and (VBICanLib64949_0997) and (VBICanLib64949_0995)	Cofactor and Prosthetic Group Biosynthesis
S2FE2ST	SUF [2Fe-2S] Transfer	(VBICanLib64949_0928) and (VBICanLib64949_0996) and (VBICanLib64949_0997) and (VBICanLib64949_0995)	Cofactor and Prosthetic Group Biosynthesis
S4FE4SR	SUF [4Fe-4S] Reduction	(VBICanLib64949_0928) and (VBICanLib64949_0996) and (VBICanLib64949_0997) and (VBICanLib64949_0995)	Cofactor and Prosthetic Group Biosynthesis
S4FE4ST	SUF [4Fe-4S] Transfer	(VBICanLib64949_0928) and (VBICanLib64949_0996) and (VBICanLib64949_0997) and (VBICanLib64949_0995)	Cofactor and Prosthetic Group Biosynthesis
SCYSDS	SUF Cysteine desulfuration	(VBICanLib64949_0997)	Cofactor and Prosthetic Group Biosynthesis
FEex	Iron transporter	((VBICanLib64949_0837) and (VBICanLib64949_0668)) or (VBICanLib64949_0081)	iron metabolism
FEpp	Iron transporter	((VBICanLib64949_0668) and (VBICanLib64949_0837))	iron metabolism
Kt3pp	Potassium transport out via proton antiport (periplasm)	(VBICanLib64949_0844) or (VBICanLib64949_0896)	Inorganic Ion Transport and Metabolism
2DHGLCNkt_tpp	2DHGLCNkt tpp	(AL011_03585)	Transport
3HAD100	3-hydroxyacyl-[acyl-carrier-protein] dehydratase (n-C10:0)	((AL011_01705) and (AL011_01700) and (AL011_01690) and (AL011_01695))	Fatty Acid Biosynthesis
3HAD120	3-hydroxyacyl-[acyl-carrier-protein] dehydratase (n-C12:0)	((AL011_02125) and (AL011_01630))	Fatty Acid Biosynthesis
3HAD121	3-hydroxyacyl-[acyl-carrier-protein] dehydratase (n-C12:1)	((AL011_01035) and (AL011_01040))	Fatty Acid Biosynthesis
3HAD141	3-hydroxyacyl-[acyl-carrier-protein] dehydratase (n-C14:1)	((AL011_02125) and (AL011_01630))	Fatty Acid Biosynthesis

3HAD161	3-hydroxyacyl-[acyl-carrier-protein] dehydratase (n-C16:1)	((AL011_01630) and (AL011_02125))	Fatty Acid Biosynthesis
3HAD80	3-hydroxyacyl-[acyl-carrier-protein] dehydratase (n-C8:0)	((AL011_01630) and (AL011_02125))	Fatty Acid Biosynthesis
3OAR120	3-oxoacyl-[acyl-carrier-protein] reductase (n-C12:0)	(AL011_03290) or (AL011_01020) or (AL011_01640)	Fatty Acid Biosynthesis
3OAR121	3-oxoacyl-[acyl-carrier-protein] reductase (n-C12:1)	(AL011_03290) or (AL011_01020) or (AL011_01640)	Fatty Acid Biosynthesis
3OAR141	3-oxoacyl-[acyl-carrier-protein] reductase (n-C14:1)	(AL011_03290) or (AL011_01020) or (AL011_01640)	Fatty Acid Biosynthesis
3OAR181	3-oxoacyl-[acyl-carrier-protein] reductase (n-C18:1)	(AL011_03290) or (AL011_01020) or (AL011_01640)	Fatty Acid Biosynthesis
3OAR40	3-oxoacyl-[acyl-carrier-protein] reductase (n-C4:0)	(AL011_03290) or (AL011_01020) or (AL011_01640)	Fatty Acid Biosynthesis
3OAS141	3-oxoacyl-[acyl-carrier-protein] synthase (n-C14:1)	(AL011_01635) or (AL011_03300)	Fatty Acid Biosynthesis
3OAS181	3-oxoacyl-[acyl-carrier-protein] synthase (n-C18:1)	((AL011_03300) and (AL011_03470) and (AL011_01780)) or (AL011_01635) or (AL011_01780)	Fatty Acid Biosynthesis
3OAS60	3-oxoacyl-[acyl-carrier-protein] synthase (n-C6:0)	((AL011_04825) or (AL011_04680))	Fatty Acid Biosynthesis
4HTHRS	4-Hydroxy-L-threonine synthase	(AL011_01655)	Vitamin B6 Metabolism
ACCOAC	Acetyl-CoA carboxylase	((AL011_01875) and (AL011_00660) and (AL011_03040) and (AL011_00665))	Fatty Acid Biosynthesis
ACGS	N-acetylglutamate synthase	((AL011_01755) or (AL011_00965))	Urea cycle/amino group metabolism
ACHBS	2-aceto-2-hydroxybutanoate synthase	(AL011_00030)	Valine, Leucine, and Isoleucine Metabolism
ACOATA	Acetyl-CoA ACP transacylase	((AL011_01610) or (AL011_02090))	Fatty Acid Biosynthesis
ACONT	Aconitate hydratase	(AL011_01575)	TCA Cycle
ACONTa	Aconitase (half-reaction A, Citrate hydro-lyase)	(AL011_01080) or (AL011_04680)	TCA Cycle
ACONTb	Aconitase (half-reaction B, Isocitrate hydro-lyase)	(AL011_01575))	TCA Cycle
ACOTA	Acetylornithine transaminase	((AL011_01485) and (AL011_01775))	Urea cycle/amino group metabolism
ADSL1r	Adenylsuccinate lyase	(AL011_04250)	Purine Metabolism
ADSL2r	Adenylosuccinate lyase	(AL011_04250) or (AL011_00280) or (AL011_01180)	Purine Metabolism

AGPAT120	1-tetradecanoyl-sn-glycerol 3-phosphate O-acyltransferase (n-C12:0)	(AL011_03075)	Glycerophospholipid Metabolism
AGPAT160	1-hexadecanoyl-sn-glycerol 3-phosphate O-acyltransferase (n-C16:0)	(AL011_03075)	Glycerophospholipid Metabolism
AGPAT161	1-hexadec-7-enoyl-sn-glycerol 3-phosphate O-acyltransferase (n-C16:1)	(AL011_03075)	Glycerophospholipid Metabolism
AGPAT180	1-octadecanoyl-sn-glycerol 3-phosphate O-acyltransferase (n-C18:0)	(AL011_03075)	Glycerophospholipid Metabolism
AGPAT181	1-octadec-7-enoyl-sn-glycerol 3-phosphate O-acyltransferase (n-C18:1)	(AL011_03075)	Glycerophospholipid Metabolism
AICART	Phosphoribosylaminoimidazolecarboxamide formyltransferase	(AL011_02645)	Purine Metabolism
AKGDH	2-Oxogluterate dehydrogenase	((AL011_00525) or (AL011_01275))	TCA Cycle
ALAALAr	D-alanine-D-alanine ligase (reversible)	((AL011_05235))	Peptidoglycan Biosynthesis
ANPRT	Anthranilate phosphoribosyltransferase	(AL011_01395)	Phenylalanine Tyrosine Tryptophan Biosynthesis
ANS	Anthranilate synthase	(AL011_01395)	Phenylalanine Tyrosine Tryptophan Biosynthesis
ARGSL	Argininosuccinate lyase	((AL011_00745) and (AL011_01865)) or (AL011_00745)	Arginine and Proline Metabolism
ARGSS	Argininosuccinate synthase	(AL011_00495)	Arginine and Proline Metabolism
ARGabcpp	L-arginine transport via ABC system (periplasm)	(AL011_00015)	Transport
ASAD	Aspartate-semialdehyde dehydrogenase	(AL011_00860)	Lysine Biosynthesis
ASNS1	Asparagine synthase (glutamine-hydrolysing)	(AL011_01015)	Alanine and Aspartate Metabolism
ASPabcpp	L-aspartate transport via ABC system (periplasm)	((AL011_00185) and (AL011_00180) and (AL011_04930) and (AL011_04930)) or (AL011_01180)	Transport
ATPPRT	ATP phosphoribosyltransferase	(AL011_01655)	Histidine Metabolism
ATPS4rpp	ATP synthase (four protons for one ATP) (periplasm)	((AL011_02880) and (AL011_02895) and (AL011_04810) and (AL011_02885) and (AL011_02875) and (AL011_02890)) or (AL011_03480)	Oxidative Phosphorylation
BZt1pp	Benzoate transport in via proton symport (periplasm)	(AL011_01265)	B-Ketoadipate pathway

CHLabcpp	Choline transport via ABC system (periplasm)	((AL011_01040) and (AL011_01035)) or (AL011_02465) or (AL011_03000) or (AL011_03175)	Transport
CHOLS_ex	CHOLS LPAREN ex RPAREN	(AL011_01655)	Transport
DAPE	Diaminopimelate epimerase	(AL011_02430)	Lysine Biosynthesis
CHOLSabc	CHOLSabc	((AL011_01045) and (AL011_01040) and (AL011_01035)) or (AL011_02465) or (AL011_03000) or (AL011_03175)	Glycine, Serine and threonine metabolism
CITtex	Citrate transport via diffusion (extracellular to periplasm)	(AL011_03930)	Transport
COBALT2tpp	Cobalt transport in via permease (no H+)	(AL011_02995)	Transport
CS	Citrate synthase	((AL011_01035) and (AL011_01040))	TCA Cycle
CTPS2	CTP synthase (glutamine)	((AL011_01035) and (AL011_01040))	Pyrimidine Metabolism
CYO1b	CYO1b	((AL011_01700) and (AL011_01705))	Oxidative Phosphorylation
CYSabcpp	L-cysteine uptake via ABC system (periplasm)	(AL011_03470))	Transport
CYTBO3_4pp	Cytochrome oxidase bo3 (ubiquinol-8: 4 protons) (periplasm)	((AL011_01705) and (AL011_01695) and (AL011_01690) and (AL011_01700))	Oxidative Phosphorylation
DAPDC	Diaminopimelate decarboxylase	(AL011_01185)	Lysine Biosynthesis
DASYN120	CDP-diacylglycerol synthetase (n-C12:0)	(AL011_02105)	Glycerophospholipid Metabolism
DASYN160	CDP-diacylglycerol synthetase (n-C16:0)	(AL011_02105)	Glycerophospholipid Metabolism
DASYN161	CDP-diacylglycerol synthetase (n-C16:1)	(AL011_02105)	Glycerophospholipid Metabolism
DASYN180	CDP-diacylglycerol synthetase (n-C18:0)	(AL011_02105)	Glycerophospholipid Metabolism
DASYN181	CDP-diacylglycerol synthetase (n-C18:1)	(AL011_02105)	Glycerophospholipid Metabolism
DB4PS	3,4-Dihydroxy-2-butanone-4-phosphate synthase	(AL011_04735)	Riboflavin Metabolism
DDPA	3-deoxy-D-arabino-heptulosonate 7-phosphate synthetase	(AL011_02630)	Phenylalanine Tyrosine Tryptophan Biosynthesis
DGK1	Deoxyguanylate kinase (dGMP:ATP)	((AL011_01875) and (AL011_00660) and (AL011_00665) and (AL011_03040))	Purine Metabolism

DHPM1	Dihydropyrimidinase (5,6-dihydrouracil)	(AL011_00835)	Pantothenate and CoA Biosynthesis
DMATT	Dimethylallyltranstransferase	(AL011_04925) or (AL011_02220)	Steroid biosynthesis
DNAMTn2r	DNA (cytosine-5-)-methyltransferase	(AL011_03385) or (AL011_03380)	Methionine Metabolism
DNTPPA	Dihydroneopterin triphosphate pyrophosphatase	(AL011_00960)	Folate Biosynthesis
DPCOAK	Dephospho-CoA kinase	(AL011_01880)	Pantothenate and CoA Biosynthesis
DPR	2-dehydropantoate 2-reductase	(AL011_04865)	Pantothenate and CoA Biosynthesis
DTMPK	DTMP kinase	(AL011_00415)	Pyrimidine Metabolism
DURAD	Dihydrouracil dehydrogenase (NADP)	(AL011_01495)	Pantothenate and CoA Biosynthesis
DUTPDP	DUTP diphosphatase	(AL011_04510)	Pyrimidine Metabolism
DXPS	1-deoxy-D-xylulose 5-phosphate synthase	(AL011_02610)	Steroid biosynthesis
E4PD	Erythrose 4-phosphate dehydrogenase	((AL011_02690) and (AL011_02690))	Vitamin B6 Metabolism
EAR100y	Enoyl-[acyl-carrier-protein] reductase (NADPH) (n-C10:0)	(AL011_01640) or (AL011_05175)	Fatty Acid Biosynthesis
EAR120y	Enoyl-[acyl-carrier-protein] reductase (NADPH) (n-C12:0)	((AL011_01035) and (AL011_01040))	Fatty Acid Biosynthesis
EAR121y	Enoyl-[acyl-carrier-protein] reductase (NADPH) (n-C12:1)	((AL011_01035) and (AL011_01040))	Fatty Acid Biosynthesis
EAR160y	Enoyl-[acyl-carrier-protein] reductase (NADPH) (n-C16:0)	((AL011_02465) and (AL011_01040)) or ((AL011_01035) and (AL011_01040))	Fatty Acid Biosynthesis
EAR161y	Enoyl-[acyl-carrier-protein] reductase (NADPH) (n-C16:1)	(AL011_01640) or (AL011_05175)	Fatty Acid Biosynthesis
EAR180y	Enoyl-[acyl-carrier-protein] reductase (NADPH) (n-C18:0)	(AL011_01640) or (AL011_05175)	Fatty Acid Biosynthesis
EAR181y	Enoyl-[acyl-carrier-protein] reductase (NADPH) (n-C18:1)	(AL011_01640) or (AL011_05175)	Fatty Acid Biosynthesis
EAR40y	Enoyl-[acyl-carrier-protein] reductase (NADPH) (n-C4:0)	(AL011_01640) or (AL011_05175)	Fatty Acid Biosynthesis
EAR60y	Enoyl-[acyl-carrier-protein] reductase (NADPH) (n-C6:0)	(AL011_01640) or (AL011_05175)	Fatty Acid Biosynthesis
EAR80y	Enoyl-[acyl-carrier-protein] reductase (NADPH) (n-C8:0)	(AL011_01640) or (AL011_05175)	Fatty Acid Biosynthesis
ETHSO3abcpp	Ethanesulfonate transport via ABC system (periplasm)	((AL011_02995) and (AL011_03000) and (AL011_03665))	Transport

FDH	Formate dehydrogenase	((AL011_03665) and (AL011_03660) and (AL011_03655))	Methane Metabolism
FE2abc	Iron (II) transport via ABC system	(AL011_01035) or ((AL011_03175) and (AL011_03170) and (AL011_03155))	Transport
FERtex	FERtex	(AL011_02275)	Transport
FERtpp	FERtpp	(AL011_02275)	Transport
FTHFD	Formyltetrahydrofolate deformylase	((AL011_02685) or (AL011_02685))	One Carbon pool by folate
FUM	Fumarase	((AL011_00525) or (AL011_01275))	TCA Cycle
FUMtex	Fumarate transport via diffusion (extracellular to periplasm)	(AL011_00280)	Transport
G1SAT	Glutamate-1-semialdehyde aminotransferase	(AL011_01485)	Porphyrin and Chlorophyll Metabolism
G3PAT120	Glycerol-3-phosphate acyltransferase (C12:0)	(AL011_03295)	Glycerophospholipid Metabolism
G3PAT160	Glycerol-3-phosphate acyltransferase (C16:0)	(AL011_03295)	Glycerophospholipid Metabolism
G3PAT161	Glycerol-3-phosphate acyltransferase (C16:1)	(AL011_03295)	Glycerophospholipid Metabolism
G3PAT180	Glycerol-3-phosphate acyltransferase (C18:0)	(AL011_03295)	Glycerophospholipid Metabolism
G3PAT181	Glycerol-3-phosphate acyltransferase (C18:1)	(AL011_03295)	Glycerophospholipid Metabolism
GART	GAR transformylase-T	(AL011_00125)	Purine Metabolism
GLCNt2rpp	D-gluconate transport via proton symport, reversible (periplasm)	(AL011_01085)	Transport
GLCabcpp	D-glucose transport via ABC system (periplasm)	(AL011_00190) or (AL011_01035) or (AL011_03175)	Transport
GLNS	Glutamine synthetase	(AL011_00190)) or (AL011_03000)	Glutamate Metabolism
GLNTRAT	Glutamyl-tRNA(Gln) amidotransferase	((AL011_04265) and (AL011_04275) and (AL011_04270))	Glutamate Metabolism
GLUSy	Glutamate synthase (NADPH)	(AL011_00745)	Glutamate Metabolism
GLUt4pp	Na+/glutamate symport (periplasm)	(AL011_04210)	Transport
GLXCL	Glyoxalate carboligase	(AL011_00810) or (AL011_02615)	Glycolate metabolism
GLYALDtex	Glyceraldehyde transport via diffusion (extracellular to periplasm)	(AL011_01825) or (AL011_00185)	Transport

GLYBabcpp	Glycine betaine transport via ABC system (periplasm)	(AL011_01265)	Arginine and Proline Metabolism
GLYCK	Glycerate kinase	(AL011_03970)	Glycine, Serine and threonine metabolism
GLYCL	Glycine Cleavage System	(AL011_02600)) or (AL011_04640) or (AL011_04650) or (AL011_02600) or (AL011_04640) or (AL011_04650)	Glycine, Serine and threonine metabolism
GLYCLTt2rpp	Glycolate transport via proton symport, reversible (periplasm)	(AL011_02600)) or (AL011_04640) or (AL011_04650) or (AL011_02600) or (AL011_04640) or (AL011_04650)	Transport
GLYCLTtex	Glycolate transport via diffusion (extracellular to periplasm)	(AL011_02600)) or (AL011_04640) or (AL011_04650) or (AL011_02600) or (AL011_04640) or (AL011_04650)	Transport
GLYCL_2	Glycine cleavage system, cytosol	(AL011_02600)) or (AL011_04640) or (AL011_04650) or (AL011_02600) or (AL011_04640) or (AL011_04650)	Nitrogen Metabolism
GLYCTO1	Glycolate oxidase	(AL011_02185)	Glycolate metabolism
GLYTRS	Glycyl-tRNA synthetase	((AL011_04835) and (AL011_02210))	Glycine, Serine and threonine metabolism
GMPS2	GMP synthase	(AL011_02210))	Purine Metabolism
GND	Phosphogluconate dehydrogenase	(AL011_02400)	Pentose Phosphate Pathway
GRTT	Geranyltranstransferase	(AL011_04925) or (AL011_02220)	Steroid biosynthesis
GTHPi	Glutathione peridoxase	(AL011_05400)	Glutathione Metabolism
GTHS	Glutathione synthetase	((AL011_04935) and (AL011_04930) and (AL011_00190))	Glutamate Metabolism
GTPCII	GTP cyclohydrolase II	(AL011_01125)	Riboflavin Metabolism
HCO3_NAt	Bicarbonate transport (Na/HCO3 cotransport)	(AL011_04010)	Transport
HEMEOS	Heme O synthase	(AL011_01130)	Porphyrin and Chlorophyll Metabolism
HISabcpp	L-histidine transport via ABC system (periplasm)	(AL011_01040) and (AL011_02155)	Transport
HMBS	Hydroxymethylbilane synthase	(AL011_04565)	Porphyrin and Chlorophyll Metabolism
HMGL	Hydroxymethylglutaryl-CoA lyase	(AL011_00295)	Synthesis and degradation of Ketones Bodies
HPPK	2-amino-4-hydroxy-6-hydroxymethyldihydropteridine diphosphokinase	(34021.12.peg.1110)	Folate Biosynthesis
HXAtex	Hexanoate transport via diffusion (extracellular to periplasm)	(AL011_04010)	Transport

ICDHyr	Isocitrate dehydrogenase (NADP)	(AL011_03900)	TCA Cycle
ICITtex	Citrate transport via diffusion extracellular to periplasm	(AL011_04215)	Transport
ICL	Isocitrate lyase	(AL011_01725)	TCA Cycle
IGPS	Indole-3-glycerol-phosphate synthase	(AL011_00030)	Phenylalanine Tyrosine Tryptophan Biosynthesis
ILEabcpp	L-isoleucine transport via ABC system (periplasm)	(AL011_04215)	Transport
IMPD	IMP dehydrogenase	(AL011_03830)	Purine Metabolism
IPDDI	Isopentenyl-diphosphate D-isomerase	(AL011_04745)	Steroid biosynthesis
IPDPS	1-hydroxy-2-methyl-2-(E)-butenyl 4-diphosphate reductase (ipdp)	(AL011_04530)	Steroid biosynthesis
IPMD	3-isopropylmalate dehydrogenase	(AL011_03900)	Valine, Leucine, and Isoleucine Metabolism
IPPMIa	3-isopropylmalate dehydratase	(AL011_01575) or (AL011_03455)	Valine, Leucine, and Isoleucine Metabolism
IPPMIb	2-isopropylmalate hydratase	(AL011_01575) or (AL011_03455)	Valine, Leucine, and Isoleucine Metabolism
KARA1	Ketol-acid reductoisomerase (2,3-dihydroxy-3-methylbutanoate)	(34021.12.peg.1195)	Valine, Leucine, and Isoleucine Metabolism
KARA2	Ketol-acid reductoisomerase (2-Acetolactate)	(34021.12.peg.1195)	Valine, Leucine, and Isoleucine Metabolism
KAS14	Beta-ketoacyl-ACP synthase	(AL011_01635)	Fatty Acid Biosynthesis
LEUTRS	Leucyl-tRNA synthetase	(AL011_01965)	Valine, Leucine, and Isoleucine Metabolism
LEUabcpp	L-leucine transport via ABC system (periplasm)	(AL011_04215) and (AL011_05135)	Transport
LYSTRS	Lysyl-tRNA synthetase	(AL011_04960)	Lysine Biosynthesis
LYSabcpp	L-lysine transport via ABC system (periplasm)	(AL011_05355)	Lysine Metabolism
MACPD	Malonyl-ACP decarboxylase	(AL011_00190)	Fatty Acid Biosynthesis
MCOATA	Malonyl-CoA-ACP transacylase	(AL011_03285)	Fatty Acid Biosynthesis
MSO3abcpp	Methanesulfonate transport via ABC system (periplasm)	(AL011_02995)	Transport
MTHFC	Methenyltetrahydrofolate cyclohydrolase	(AL011_03430)	One Carbon pool by folate
MTHFD	Methylenetetrahydrofolate dehydrogenase (NADP)	(AL011_03430)	One Carbon pool by folate
MHPGLUT	5 methyltetrahydropteroyltriglutamate homocysteine S methyltransferase	(AL011_04785)	Methionine Metabolism

NADH16pp	NADH dehydrogenase (ubiquinone-8 & 3 protons) (periplasm)	(AL011_03635) and (AL011_03640) and (AL011_03645) and (AL011_03650) and (AL011_03655) and (AL011_03660) and (AL011_03665) and (AL011_03670) and (AL011_03675) and (AL011_03680) and (AL011_03685) and (AL011_03690) and (AL011_03695) and (AL011_03700)	Oxidative Phosphorylation
NADK	NAD kinase	(AL011_02225)	Nicotinate and Nicotinamide
NDPK1	Nucleoside-diphosphate kinase (ATP:GDP)	(AL011_01275)	Purine Metabolism
NDPK2	Nucleoside-diphosphate kinase (ATP:UDP)	(AL011_01275)	Pyrimidine Metabolism
NDPK3	Nucleoside-diphosphate kinase (ATP:CDP)	(AL011_01275)	Pyrimidine Metabolism
NDPK4	Nucleoside-diphosphate kinase (ATP:dTDP)	(AL011_01275)	Pyrimidine Metabolism
NDPK5	Nucleoside-diphosphate kinase (ATP:dGDP)	(AL011_01275)	Purine Metabolism
NDPK6	Nucleoside-diphosphate kinase (ATP:dUDP)	(AL011_01275)	Pyrimidine Metabolism
NDPK7	Nucleoside-diphosphate kinase (ATP:dCDP)	(AL011_01275)	Pyrimidine Metabolism
NDPK8	Nucleoside-diphosphate kinase (ATP:dADP)	(AL011_01275)	Purine Metabolism
NNATr	Nicotinate-nucleotide adenylyltransferase	(AL011_02715)	Nicotinate and Nicotinamide
NO2t2rpp	Nitrite transport in via proton symport, reversible (periplasm)	(AL011_04330)	Transport
OIVD1r	2-oxoisovalerate dehydrogenase (acylating; 4-methyl-2-oxopentaoate)	(AL011_04630)	Valine, leucine and isoleucine degradation
OIVD2	2-oxoisovalerate dehydrogenase (acylating; 3-methyl-2-oxobutanoate)	(AL011_04630)	Valine, leucine and isoleucine degradation
OIVD3	2-oxoisovalerate dehydrogenase (acylating; 3-methyl-2-oxopentanoate)	(AL011_04630)	Valine, leucine and isoleucine degradation
ORNCD	Ornithine cyclodeaminase	(AL011_04580)	Arginine and Proline Metabolism
ORNabcpp	Ornithine transport via ABC system (periplasm)	(AL011_00190)	Lysine Metabolism

ORPT	Orotate phosphoribosyltransferase	(AL011_04445)	Pyrimidine Metabolism
PANTS	Pantothenate synthase	(AL011_02855)	Pantothenate and CoA Biosynthesis
PDH	Pyruvate dehydrogenase	(AL011_02600)	Glycolysis
PDX5POi	Pyridoxine 5-phosphate oxidase	(AL011_04220)	Vitamin B6 Metabolism
PGAMT	Phosphoglucosamine mutase	(AL011_03250)	Aminosugar Metabolism
PGM	Phosphoglycerate mutase	(AL011_00720)	Gluconeogenesis
PGMT	Phosphoglucomutase	(AL011_04905)	Glycolysis
PMDPHT	Pyrimidine phosphatase	(AL011_00160)	Riboflavin Metabolism
PPA	Inorganic diphosphatase	(AL011_00485)	Oxidative Phosphorylation
PPBNGS	Porphobilinogen synthase	(AL011_01095)	Porphyrin and Chlorophyll Metabolism
PPPGO	Protoporphyrinogen oxidase (aerobic)	(AL011_04735)	Porphyrin and Chlorophyll Metabolism
PRAGSr	Phosphoribosylglycinamide synthase	(AL011_00375)	Purine Metabolism
PRAIi	Phosphoribosylanthranilate isomerase (irreversible)	(AL011_00030)	Phenylalanine Tyrosine Tryptophan Biosynthesis
PRAIS	Phosphoribosylaminoimidazole synthase	(AL011_04245)	Purine Metabolism
PRFGS	Phosphoribosylformylglycinamidine synthase	(AL011_04230)	Purine Metabolism
PSP_L	Phosphoserine phosphatase (L-serine)	((AL011_01035) and (AL011_01040))	Glycine, Serine and threonine metabolism
RIBabcpp	D-ribose transport via ABC system (periplasm)	(AL011_03175) or (AL011_03000)	Transport
RPE	Ribulose 5-phosphate 3-epimerase	(AL011_04220)	Pentose Phosphate Pathway
RPI	Ribose-5-phosphate isomerase	(AL011_04645)	Pentose Phosphate Pathway
SDPTA	Succinyldiaminopimelate transaminase	(AL011_01485)	Lysine Biosynthesis
SERAT	Serine O-acetyltransferase	(AL011_02565)	Cysteine Metabolism
SERTRS	Seryl-tRNA synthetase	(AL011_04220)	Glycine, Serine and threonine metabolism
SHCHF	Sirohydrochlorin ferrochelatase	(AL011_02910)	Porphyrin and Chlorophyll Metabolism
SOTA	Succinylornithine transaminase	(AL011_01485)	Arginine and Proline Metabolism
SUCD1	Succinate dehydrogenase	(AL011_04595) and (AL011_04600) and (AL011_04590)	TCA Cycle
SUCDi	Succinate dehydrogenase (irreversible)	(AL011_04595) and (AL011_04600) and (AL011_04590)	TCA Cycle
SUCOAS	Succinyl-CoA synthetase (ADP-forming)	(AL011_04620)	TCA Cycle
SULabcpp	Sulfate transport via ABC system (periplasm)	(AL011_03860)	Sulfur Metabolism

TKT1	Transketolase	(AL011_02685)	Pentose Phosphate Pathway
TRSARr	Tartronate semialdehyde reductase	(AL011_02400)	Glycolate metabolism
TSULabcpp	Thiosulfate transport via ABC system (periplasm)	(AL011_03175) or (AL011_01035)	Sulfur Metabolism
UDCPDP	Undecaprenyl-diphosphatase	(AL011_03015)	Peptidoglycan Biosynthesis
UMPK	UMP kinase	(AL011_02090)	Pyrimidine Metabolism
UPPN	B-ureidopropionase	(AL011_04855)	Pantothenate and CoA Biosynthesis
UREA	Urease	(AL011_03815)	Urea cycle/amino group metabolism
VALTRS	Valyl-tRNA synthetase	(AL011_04050)	Valine, Leucine, and Isoleucine Metabolism
VANKpp	VANKpp	(AL011_04215)	Transport
VANLNtex	VANLNtex	(AL011_04215)	Transport
VANLTtex	VANLTtex	(AL011_04215)	Transport
VNLNpp	VNLNpp	(AL011_04215)	Transport
pentso3abcpp	Pentso3abcpp	(AL011_03000)	Transport
ACOLIPAabctex	Arabinose modified core oligosaccharide lipid A transport via ABC system (periplasm to extracellular)	((AL011_01290) and (AL011_02275)) or (AL011_03275) or (AL011_03175) or (AL011_00025) or (AL011_02465)	Lipopolysaccharide Biosynthesis / Recycling
ALATA_D2	D-alanine transaminase	(AL011_01080))	Cofactor and Prosthetic Group Biosynthesis
ALATA_L2	Alanine transaminase	((AL011_04680) or (AL011_01080))	Cofactor and Prosthetic Group Biosynthesis
AMPTASECG	Alanyl aminopeptidase (cys-gly)	(AL011_01285)	Cysteine Metabolism
AMPTASEPG	Aminopeptidase (pro-gly)	(AL011_01285)	Arginine and Proline Metabolism
ASCBptspp	L-ascorbate transport via PEP:Pyr PTS (periplasm)	(AL011_05185)	Transport, Inner Membrane
BUTSO3abcpp	Butanesulfonate transport via ABC system (periplasm)	((AL011_03000) and (AL011_02995) and (AL011_01040))	Transport, Inner Membrane
CLIPAabctex	Cold lipid A transport via ABC system (periplasm to extracellular)	((AL011_02350) and (AL011_01290) and (AL011_02275) and (AL011_02270) and (AL011_01300) and (AL011_01550))	Lipopolysaccharide Biosynthesis / Recycling
COLIPAPabctex	Core oligosaccharide lipid A diphosphate transport via ABC system (periplasm to extracellular)	((AL011_01290) and (AL011_02270) and (AL011_02275))	Lipopolysaccharide Biosynthesis / Recycling
COLIPAabctex	Core oligosaccharide lipid A transport via ABC system (periplasm to extracellular)	((AL011_01290) and (AL011_02270) and (AL011_02275))	Lipopolysaccharide Biosynthesis / Recycling
CRNDabcpp	D-carnitine transport via ABC system (periplasm)	((AL011_01035) and (AL011_01040))	Transport, Inner Membrane

CYSSADS	L-cysteine sulfinic acid desulfurase	((AL011_04825) or (AL011_04680))	Cysteine Metabolism
ECA4COLIPAabctex	Enterobacterial common antigen (x4) core oligosaccharide lipid A transport via ABC system (periplasm to extracellular)	((AL011_02270) and (AL011_02350) and (AL011_02275) and (AL011_01290))	Lipopolysaccharide Biosynthesis / Recycling
ENLIPAabctex	Phosphoethanolamine lipid A transport via ABC system (periplasm to extracellular)	((AL011_02275) and (AL011_01290) and (AL011_02270))	Lipopolysaccharide Biosynthesis / Recycling
GRXR	Glutaredoxin reductase	(AL011_04220)	Unassigned
K2L4Aabctex	KDO(2)-lipid IV A transport via ABC system (periplasm to extracellular)	(AL011_03970)	Lipopolysaccharide Biosynthesis / Recycling
KAS15	Beta-ketoacyl-ACP synthase (2)	(AL011_03300)	Membrane Lipid Metabolism
KDOPS	3-deoxy -D-manno-octulosonic -acid 8-phosphate synthase	(AL011_03470)	Lipopolysaccharide Biosynthesis / Recycling
LIPAabctex	Lipid A transport via ABC system (periplasm to extracellular)	(AL011_03970)	Lipopolysaccharide Biosynthesis / Recycling
NADH17pp	NADH dehydrogenase (menaquinone-8 & 3 protons) (periplasm)	(AL011_03635) and (AL011_03640) and (AL011_03645) and (AL011_03650) and (AL011_03655) and (AL011_03660) and (AL011_03665) and (AL011_03670) and (AL011_03675) and (AL011_03680) and (AL011_03685) and (AL011_03690) and (AL011_03695) and (AL011_03700)	Oxidative Phosphorylation
NADH18pp	NADH dehydrogenase (demethylmenaquinone-8 & 3 protons) (periplasm)	(AL011_03635) and (AL011_03640) and (AL011_03645) and (AL011_03650) and (AL011_03655) and (AL011_03660) and (AL011_03665) and (AL011_03670) and (AL011_03675) and (AL011_03680) and (AL011_03685) and (AL011_03690) and (AL011_03695) and (AL011_03700)	Oxidative Phosphorylation
NADHHR	NADH hydratase	(AL011_02690)	Metabolite Repair
NADHHS	NADH hydratase	(AL011_02690)	Metabolite Repair
NADPHHR	NADPH hydratase	(AL011_02690)	Metabolite Repair
NADPHHS	NADPH hydratase	(AL011_02690)	Metabolite Repair

O16A4COLIPAabctex	O16 antigen (x4) core oligosaccharide lipid A transport via ABC system (periplasm to extracellular)	(AL011_02265) and (AL011_02270) and (AL011_02275)	Lipopolysaccharide Biosynthesis / Recycling
OCTDPS	Octaprenyl pyrophosphate synthase	(AL011_01275))	Cofactor and Prosthetic Group Biosynthesis
OMBZLM	2-Octaprenyl-6-methoxy-benzoquinol methylase	(AL011_01275))	Cofactor and Prosthetic Group Biosynthesis
RNDR3b	Ribonucleoside-diphosphate reductase (CDP) (glutaredoxin)	((AL011_03450) and (AL011_03600)) or (AL011_04205)	Nucleotide Salvage Pathway
RNDR4b	Ribonucleoside-diphosphate reductase (UDP) (glutaredoxin)	(AL011_02155)	Nucleotide Salvage Pathway
S2FE2SR	SUF [2Fe-2S] regeneration	(AL011_04375) and (AL011_04680) and (AL011_04675) and (AL011_04670)	Cofactor and Prosthetic Group Biosynthesis
S2FE2SS	SUF [2Fe-2S] Synthesis	(AL011_04375) and (AL011_04680) and (AL011_04675) and (AL011_04670)	Cofactor and Prosthetic Group Biosynthesis
S2FE2SS2	SUF [2Fe-2S] Synthesis II	(AL011_04375) and (AL011_04680) and (AL011_04675) and (AL011_04670)	Cofactor and Prosthetic Group Biosynthesis
S2FE2ST	SUF [2Fe-2S] Transfer	(AL011_04375) and (AL011_04680) and (AL011_04675) and (AL011_04670)	Cofactor and Prosthetic Group Biosynthesis
S4FE4SR	SUF [4Fe-4S] Reduction	(AL011_04375) and (AL011_04680) and (AL011_04675) and (AL011_04670)	Cofactor and Prosthetic Group Biosynthesis
S4FE4ST	SUF [4Fe-4S] Transfer	(AL011_04375) and (AL011_04680) and (AL011_04675) and (AL011_04670)	Cofactor and Prosthetic Group Biosynthesis
SCYSDS	SUF Cysteine desulfuration	(AL011_04680)	Cofactor and Prosthetic Group Biosynthesis
FEex	Iron transporter	((AL011_03280) and (AL011_03275) and (AL011_03265)) or (AL011_03970)	iron metabolism
FEpp	Iron transporter	((AL011_03280) and (AL011_03275) and (AL011_03265)) or (AL011_03970)	iron metabolism
Kt3pp	Potassium transport out via proton antiport (periplasm)	(AL011_04005)	Inorganic Ion Transport and Metabolism
2DHGLCNkt_tpp	2DHGLCNkt tpp	(B488_10160) or (B488_02930) or (B488_00560) or (B488_08370) or (B488_11610)	Transport
4HBZtex	4-Hydroxybenzoate transport (extracellular)	(B488_01170) or (B488_06670)	Transport
4HTHRS	4-Hydroxy-L-threonine synthase	(B488_05900) or (B488_00990)	Vitamin B6 Metabolism
ACCOAC	Acetyl-CoA carboxylase	((B488_13670) and (B488_09100) and (B488_11350) and (B488_09110))	Fatty Acid Biosynthesis

ACGS	N-acetylglutamate synthase	(B488_13230) or (B488_03310)	Urea cycle/amino group metabolism
ACHBS	2-aceto-2-hydroxybutanoate synthase	(B488_06340)	Valine, Leucine, and Isoleucine Metabolism
ACOATA	Acetyl-CoA ACP transacylase	((B488_10110) and (B488_03640))	Fatty Acid Biosynthesis
ACONT	Aconitate hydratase	(B488_01200)	TCA Cycle
ACONTa	Aconitase (half-reaction A, Citrate hydro-lyase)	(B488_01200)	TCA Cycle
ACONTb	Aconitase (half-reaction B, Isocitrate hydro-lyase)	(B488_01200)	TCA Cycle
ADSL1r	Adenylsuccinate lyase	(B488_05360) or (B488_04530)	Purine Metabolism
ADSL2r	Adenylosuccinate lyase	(B488_05360) or (B488_02110) or (B488_04530) or (B488_04420)	Purine Metabolism
AGPAT120	1-tetradecanoyl-sn-glycerol 3-phosphate O-acyltransferase (n-C12:0)	(B488_01290)	Glycerophospholipid Metabolism
AGPAT160	1-hexadecanoyl-sn-glycerol 3-phosphate O-acyltransferase (n-C16:0)	(B488_01290)	Glycerophospholipid Metabolism
AGPAT161	1-hexadec-7-enoyl-sn-glycerol 3-phosphate O-acyltransferase (n-C16:1)	(B488_01290)	Glycerophospholipid Metabolism
AGPAT180	1-octadecanoyl-sn-glycerol 3-phosphate O-acyltransferase (n-C18:0)	(B488_01290)	Glycerophospholipid Metabolism
AGPAT181	1-octadec-7-enoyl-sn-glycerol 3-phosphate O-acyltransferase (n-C18:1)	(B488_01290)	Glycerophospholipid Metabolism
AKGDH	2-Oxogluterate dehydrogenase	((B488_07400) and (B488_07420) and (B488_07410))	TCA Cycle
AKGt2rpp	2-oxoglutarate reversible transport via symport (periplasm)	(B488_01170) or (B488_02930)	Transport
ALAALAr	D-alanine-D-alanine ligase (reversible)	((B488_05940) or (B488_05940))	Peptidoglycan Biosynthesis
ANPRT	Anthranilate phosphoribosyltransferase	(B488_13100) or (B488_13110) or (B488_10810)	Phenylalanine Tyrosine Tryptophan Biosynthesis
ANS	Anthranilate synthase	((B488_13100) and (B488_13120))	Phenylalanine Tyrosine Tryptophan Biosynthesis
ARGSS	Argininosuccinate synthase	(B488_10220)	Arginine and Proline Metabolism
ARGabcpp	L-arginine transport via ABC system (periplasm)	((B488_06120) and (B488_06080)) or (B488_08860) or ((B488_10540) and (B488_06120)) or ((B488_06130) and (B488_06120) and (B488_06080)) or ((B488_08860) and (B488_08850))	Transport
ASNS1	Asparagine synthase (glutamine-hydrolysing)	(B488_03130)	Alanine and Aspartate Metabolism

ASPabcpp	L-aspartate transport via ABC system (periplasm)	((B488_08850) and (B488_08840) and (B488_08830) and (B488_08830)) or ((B488_06120) and (B488_06060) or (B488_05640)) or (B488_06080) or (B488_08860)	Transport
ATPPRT	ATP phosphoribosyltransferase	(B488_09910) or (B488_00990) or (B488_05900)	Histidine Metabolism
ATPS4rpp	ATP synthase (four protons for one ATP) (periplasm)	((B488_11790) and (B488_11760) and (B488_07350) and (B488_11780) and (B488_11800) and (B488_11770))	Oxidative Phosphorylation
BZt1pp	Benzoate transport in via proton symport (periplasm)	(B488_03690) or (B488_08370)	B-Ketoadipate pathway
CATECHOLtex	CATECHOLtex	(B488_06090)	Transport
CATECHOLtpp	CATECHOLtpp	(B488_06090)	Transport
CHLabcpp	Choline transport via ABC system (periplasm)	((B488_10780) and (B488_03240)) or (B488_10540) or (B488_10770) or (B488_11480) or (B488_12610) or (B488_13000)	Transport
CHOLS_ex	CHOLS LPAREN ex RPAREN	(B488_05900)	Transport
DAPE	Diaminopimelate epimerase	(B488_12670)	Lysine Biosynthesis
CHOLSabc	CHOLSabc	(B488_10780) or (B488_03240)) or (B488_10540) or (B488_10770) or (B488_11480) or (B488_12610) or (B488_13000)	Glycine, Serine and threonine metabolism
CITt_kt	Citrate proton symport periplasm	(B488_01170) or (B488_02930)	Transport
COBALT2tex	Cobalt (Co+2) transport via diffusion (extracellular to periplasm)	(B488_11490)	Transport
CRNabcpp	L-carnitine transport via ABC system (periplasm)	((B488_10770) and (B488_10540) and (B488_10780)) or (B488_11480) or (B488_08860)	Transport
CS	Citrate synthase	(B488_10770) or ((B488_11480) and (B488_10780))	TCA Cycle
CTPS2	CTP synthase (glutamine)	(B488_02930)	Pyrimidine Metabolism
CYO1b	CYO1b	((B488_00820) and (B488_00810))	Oxidative Phosphorylation
CYSS	Cysteine synthase	((B488_05900) and (B488_02930))	Cysteine Metabolism
CYSTL	Cystathionine b-lyase	((B488_11550) and (B488_00630))	Methionine Metabolism
CYSabcpp	L-cysteine uptake via ABC system (periplasm)	(B488_01620)	Transport
DADK	Deoxyadenylate kinase	(B488_03690)	Purine Metabolism

DAPDC	Diaminopimelate decarboxylase	(B488_02090)) or ((B488_09190)	Lysine Biosynthesis
DASYN120	CDP-diacylglycerol synthetase (n-C12:0)	(B488_00450)	Glycerophospholipid Metabolism
DASYN160	CDP-diacylglycerol synthetase (n-C16:0)	(B488_00450)	Glycerophospholipid Metabolism
DASYN161	CDP-diacylglycerol synthetase (n-C16:1)	(B488_00450)	Glycerophospholipid Metabolism
DASYN180	CDP-diacylglycerol synthetase (n-C18:0)	(B488_00450)	Glycerophospholipid Metabolism
DASYN181	CDP-diacylglycerol synthetase (n-C18:1)	(B488_00450)	Glycerophospholipid Metabolism
DB4PS	3,4-Dihydroxy-2-butanone-4-phosphate synthase	(B488_05900)	Riboflavin Metabolism
DDPA	3-deoxy-D-arabino-heptulosonate 7-phosphate synthetase	(B488_07360)	Phenylalanine Tyrosine Tryptophan Biosynthesis
DHPM1	Dihydropyrimidinase (5,6-dihydrouracil)	(B488_09560)	Pantothenate and CoA Biosynthesis
DMATT	Dimethylallyltranstransferase	(B488_00690)	Steroid biosynthesis
DNAMTn2r	DNA (cytosine-5-)-methyltransferase	(B488_06600)	Methionine Metabolism
DNTPPA	Dihydroneopterin triphosphate pyrophosphatase	(B488_03320)	Folate Biosynthesis
DPCOAK	Dephospho-CoA kinase	(B488_13730)	Pantothenate and CoA Biosynthesis
DPR	2-dehydropantoate 2-reductase	(B488_01360) or (B488_11240)	Pantothenate and CoA Biosynthesis
DTMPK	DTMP kinase	(B488_08030)	Pyrimidine Metabolism
DURAD	Dihydrouracil dehydrogenase (NADP)	(B488_01480)	Pantothenate and CoA Biosynthesis
DUTPDP	DUTP diphosphatase	(B488_05450) or (B488_09830)	Pyrimidine Metabolism
DXPS	1-deoxy-D-xylulose 5-phosphate synthase	(B488_11020)	Steroid biosynthesis
EAR100y	Enoyl-[acyl-carrier-protein] reductase (NADPH) (n-C10:0)	((B488_02950) and (B488_01060))	Fatty Acid Biosynthesis
EAR120y	Enoyl-[acyl-carrier-protein] reductase (NADPH) (n-C12:0)	((B488_10280) and (B488_10270) and (B488_00950) and (B488_10290) and (B488_10260))	Fatty Acid Biosynthesis
EAR121y	Enoyl-[acyl-carrier-protein] reductase (NADPH) (n-C12:1)	((B488_10270) and (B488_10290) and (B488_10260))	Fatty Acid Biosynthesis
EAR160y	Enoyl-[acyl-carrier-protein] reductase (NADPH) (n-C16:0)	(B488_11480)	Fatty Acid Biosynthesis

EAR161y	Enoyl-[acyl-carrier-protein] reductase (NADPH) (n-C16:1)	((B488_11480) and (B488_10780))	Fatty Acid Biosynthesis
EAR180y	Enoyl-[acyl-carrier-protein] reductase (NADPH) (n-C18:0)	(B488_02950) or (B488_01060)	Fatty Acid Biosynthesis
EAR181y	Enoyl-[acyl-carrier-protein] reductase (NADPH) (n-C18:1)	(B488_02950) or (B488_01060)	Fatty Acid Biosynthesis
EAR40y	Enoyl-[acyl-carrier-protein] reductase (NADPH) (n-C4:0)	(B488_02950) or (B488_01060)	Fatty Acid Biosynthesis
EAR60y	Enoyl-[acyl-carrier-protein] reductase (NADPH) (n-C6:0)	((B488_10890)	Fatty Acid Biosynthesis
EAR80y	Enoyl-[acyl-carrier-protein] reductase (NADPH) (n-C8:0)	(B488_05290))	Fatty Acid Biosynthesis
ETHSO3abcpp	Ethanesulfonate transport via ABC system (periplasm)	((B488_11480) and (B488_10550) and (B488_05980))	Transport
FBA	Fructose-bisphosphate aldolase	(B488_12160)	Glycolysis
FBP	Fructose-bisphosphatase	(B488_05190)	Glycolysis
FDH	Formate dehydrogenase	((B488_02630) and (B488_02620) and (B488_02610))	Methane Metabolism
FE2abc	Iron (II) transport via ABC system	(B488_08860)) or (B488_10770) or (B488_11190) or (B488_11480)	Transport
FERtex	FERtex	(B488_10270) or (B488_13000)	Transport
FERtpp	FERtpp	(B488_10270) or (B488_13000)	Transport
FRUpts	D-fructose transport via PEP:Pyr PTS	(B488_02780)	Fructose Metabolism
FTHFD	Formyltetrahydrofolate deformylase	((B488_09590) or (B488_09590))	One Carbon pool by folate
FUM	Fumarase	(B488_06010)	TCA Cycle
FUMtex	Fumarate transport via diffusion (extracellular to periplasm)	(B488_04530)	Transport
G1SAT	Glutamate-1-semialdehyde aminotransferase	(B488_01500)	Porphyrin and Chlorophyll Metabolism
G3PAT120	Glycerol-3-phosphate acyltransferase (C12:0)	(B488_10110) or (B488_09760)	Glycerophospholipid Metabolism
G3PAT160	Glycerol-3-phosphate acyltransferase (C16:0)	(B488_10110) or (B488_09760)	Glycerophospholipid Metabolism
G3PAT161	Glycerol-3-phosphate acyltransferase (C16:1)	(B488_10110) or (B488_09760)	Glycerophospholipid Metabolism

G3PAT180	Glycerol-3-phosphate acyltransferase (C18:0)	(B488_10110) or (B488_09760)	Glycerophospholipid Metabolism
G3PAT181	Glycerol-3-phosphate acyltransferase (C18:1)	(B488_10110) or (B488_09760)	Glycerophospholipid Metabolism
G6PDH2r	Glucose 6-phosphate dehydrogenase	(B488_03930) or (B488_11480)	Glycolysis
GARFT	Phosphoribosylglycinamide formyltransferase	(B488_10740) or (B488_10070) or (B488_13310)	One Carbon pool by folate
GF6PTA	Glutamine-fructose-6-phosphate transaminase	(B488_03130) or (B488_06870)	Glutamate Metabolism
GLCNt2rpp	D-gluconate transport via proton symport, reversible (periplasm)	(B488_10790)	Transport
GLCabcpp	D-glucose transport via ABC system (periplasm)	(B488_08860)) or (B488_10770)	Transport
GLNS	Glutamine synthetase	(B488_12180)	Glutamate Metabolism
GLNTRAT	Glutamyl-tRNA(Gln) amidotransferase	(B488_09770) and (B488_09780)	Glutamate Metabolism
GLUSy	Glutamate synthase (NADPH)	(B488_07400)	Glutamate Metabolism
GLUt4pp	Na+/glutamate symport (periplasm)	(B488_05430)	Transport
GLXCL	Glyoxalate carboligase	(B488_06150) or (B488_02470) or (B488_11010)	Glycolate metabolism
GLYALDDr	D-Glyceraldehyde dehydrogenase	(B488_08390)	Glycerophospholipid Metabolism
GLYALDtex	Glyceraldehyde transport via diffusion (extracellular to periplasm)	(B488_11250) or (B488_12030)	Transport
GLYBabcpp	Glycine betaine transport via ABC system (periplasm)	(B488_08590)	Arginine and Proline Metabolism
GLYCK	Glycerate kinase	(B488_11520)	Glycine, Serine and threonine metabolism
GLYCL	Glycine Cleavage System	(B488_07370) or (B488_07400) or (B488_11040) or (B488_07370)) or (B488_07400) or (B488_11040)	Glycine, Serine and threonine metabolism
GLYCLTt2rpp	Glycolate transport via proton symport, reversible (periplasm)	(B488_07370) or (B488_07400) or (B488_11040) or (B488_07370)) or (B488_07400) or (B488_11040)	Transport
GLYCLTtex	Glycolate transport via diffusion (extracellular to periplasm)	(B488_07370) or (B488_07400) or (B488_11040) or (B488_07370)) or (B488_07400) or (B488_11040)	Transport
GLYCL_2	Glycine cleavage system, cytosol	(B488_07370) or (B488_07400) or (B488_11040) or (B488_07370)) or (B488_07400) or (B488_11040)	Nitrogen Metabolism

GLYCTO1	Glycolate oxidase	((B488_00600) and (B488_07480))	Glycolate metabolism
GLYTRS	Glycyl-tRNA synthetase	(B488_08160)	Glycine, Serine and threonine metabolism
GLYabcpp	Glycine transport via ABC system	(B488_08850) and (B488_06060)) or ((B488_08830) and (B488_08840) and (B488_02750)) or (B488_03240) or (B488_05640) or (B488_06080) or (B488_08860) or (B488_10540) or (B488_11190) or ((B488_12610)	Transport
GMPS2	GMP synthase	(B488_06870)	Purine Metabolism
GRTT	Geranyltranstransferase	(B488_00690)	Steroid biosynthesis
GTHPi	Glutathione peridoxase	(B488_04630)	Glutathione Metabolism
GTHS	Glutathione synthetase	(B488_10080)	Glutamate Metabolism
GTPCII	GTP cyclohydrolase II	(B488_02240) or (B488_02240)	Riboflavin Metabolism
GTPDPDP	Guanosine-5-triphosphate,3-diphosphate diphosphatase	(B488_10590)	Purine Metabolism
GUAPRT	Guanine phosphoribosyltransferase	(B488_06500)	Purine Metabolism
HCO3_NAt	Bicarbonate transport (Na/HCO3 cotransport)	(B488_12660)	Transport
HEMEOS	Heme O synthase	(B488_02230)	Porphyrin and Chlorophyll Metabolism
HISabcpp	L-histidine transport via ABC system (periplasm)	(B488_08850) and (B488_10550) and (B488_08840)	Transport
HIStex	L-histidine transport via diffusion (extracellular to periplasm)	(B488_07290)	Transport
HMBS	Hydroxymethylbilane synthase	(B488_07550)	Porphyrin and Chlorophyll Metabolism
HMGL	Hydroxymethylglutaryl-CoA lyase	(B488_01190)	Synthesis and degradation of Ketones Bodies
HXAtex	Hexanoate transport via diffusion (extracellular to periplasm)	(B488_09870) and (B488_13370) and (B488_09950) and (B488_12880)	Transport
ICDHyr	Isocitrate dehydrogenase (NADP)	(B488_04640)	TCA Cycle
ICITtex	Citrate transport via diffusion extracellular to periplasm	(B488_01170) and (B488_0528)	Transport
ICL	Isocitrate lyase	(B488_01740)	TCA Cycle
IGPS	Indole-3-glycerol-phosphate synthase	(B488_03020) or (B488_13490) or (B488_03030) or (B488_05410)	Phenylalanine Tyrosine Tryptophan Biosynthesis
ILEabcpp	L-isoleucine transport via ABC system (periplasm)	(B488_07550)	Transport
IMPC	IMP cyclohydrolase	(B488_12320)	Purine Metabolism

IPDDI	Isopentenyl-diphosphate D-isomerase	(B488_07730)	Steroid biosynthesis
IPMD	3-isopropylmalate dehydrogenase	(B488_04640) or (B488_06680)	Valine, Leucine, and Isoleucine Metabolism
IPPMIa	3-isopropylmalate dehydratase	(B488_01200)	Valine, Leucine, and Isoleucine Metabolism
IPPMIb	2-isopropylmalate hydratase	(B488_01200)	Valine, Leucine, and Isoleucine Metabolism
ISETACabcpp	Isethionate transport via ABC system (periplasm)	(B488_11480)	Transport
KARA1	Ketol-acid reductoisomerase (2,3-dihydroxy-3-methylbutanoate)	(B488_01360)	Valine, Leucine, and Isoleucine Metabolism
KARA2	Ketol-acid reductoisomerase (2-Acetolactate)	(B488_01360)	Valine, Leucine, and Isoleucine Metabolism
KAS14	Beta-ketoacyl-ACP synthase	(B488_01070)	Fatty Acid Biosynthesis
LEUTRS	Leucyl-tRNA synthetase	(B488_00070)	Valine, Leucine, and Isoleucine Metabolism
LEUabcpp	L-leucine transport via ABC system (periplasm)	(B488_10380)	Transport
LYSTRS	Lysyl-tRNA synthetase	(B488_06400)	Lysine Biosynthesis
LYSabcpp	L-lysine transport via ABC system (periplasm)	(B488_07380) and (B488_06670)	Lysine Metabolism
MACPD	Malonyl-ACP decarboxylase	((B488_01070) or (B488_10100))	Fatty Acid Biosynthesis
MCOATA	Malonyl-CoA-ACP transacylase	(B488_10130)	Fatty Acid Biosynthesis
METAT	Methionine adenosyltransferase	(B488_01620)	Methionine Metabolism
METS	Methionine synthase	(B488_04950)	Methionine Metabolism
METabcpp	L-methionine transport via ABC system (periplasm)	((B488_10540) and (B488_10550) and (B488_10560)) or (B488_08860)	Transport
MSO3abcpp	Methanesulfonate transport via ABC system (periplasm)	(B488_10130))	Transport
MTHFC	Methenyltetrahydrofolate cyclohydrolase	(B488_03910)	One Carbon pool by folate
MTHFD	Methylenetetrahydrofolate dehydrogenase (NADP)	(B488_03910)	One Carbon pool by folate
MHPGLUT	5 methyltetrahydropteroyltriglutamate homocysteine S methyltransferase	(B488_03820) or (B488_08800)	Methionine Metabolism

NADH16pp	NADH dehydrogenase (ubiquinone-8 & 3 protons) (periplasm)	(B488_02570) and (B488_02580) and (B488_02590) and (B488_02600) and (B488_02610) and (B488_02620) and (B488_02630) and (B488_02640) and (B488_02650) and (B488_02660) and (B488_02670) and (B488_02680) and (B488_02690) and (B488_02700)	Oxidative Phosphorylation
NADK	NAD kinase	(B488_00710)	Nicotinate and Nicotinamide
NAt3_1p5pp	Sodium proton antiporter (H:NA is 1.5) (periplasm)	(B488_06670) or (B488_09960)	Transport
NDPK1	Nucleoside-diphosphate kinase (ATP:GDP)	(B488_02460)	Purine Metabolism
NDPK2	Nucleoside-diphosphate kinase (ATP:UDP)	(B488_02460)	Pyrimidine Metabolism
NDPK3	Nucleoside-diphosphate kinase (ATP:CDP)	(B488_02460)	Pyrimidine Metabolism
NDPK4	Nucleoside-diphosphate kinase (ATP:dTDP)	(B488_02460)	Pyrimidine Metabolism
NDPK5	Nucleoside-diphosphate kinase (ATP:dGDP)	(B488_02460)	Purine Metabolism
NDPK6	Nucleoside-diphosphate kinase (ATP:dUDP)	(B488_02460)	Pyrimidine Metabolism
NDPK7	Nucleoside-diphosphate kinase (ATP:dCDP)	(B488_02460)	Pyrimidine Metabolism
NDPK8	Nucleoside-diphosphate kinase (ATP:dADP)	(B488_02460)	Purine Metabolism
NI2uabcpp	Nickel transport via ABC system (uptake, periplasm)	(B488_03250)	Transport
NNATr	Nicotinate-nucleotide adenylyltransferase	(B488_12100)	Nicotinate and Nicotinamide
NO2t2rpp	Nitrite transport in via proton symport, reversible (periplasm)	(B488_06750)	Transport
OIVD1r	2-oxoisovalerate dehydrogenase (acylating; 4-methyl-2-oxopentaoate)	(B488_07420)	Valine, leucine and isoleucine degradation
OIVD2	2-oxoisovalerate dehydrogenase (acylating; 3-methyl-2-oxobutanoate)	(B488_07420)	Valine, leucine and isoleucine degradation

OIVD3	2-oxoisovalerate dehydrogenase (acylating; 3-methyl-2-oxopentanoate)	(B488_07420)	Valine, leucine and isoleucine degradation
OMPDC	Orotidine-5-phosphate decarboxylase	(B488_01160)	Pyrimidine Metabolism
ORNabcpp	Ornithine transport via ABC system (periplasm)	(B488_08860)	Lysine Metabolism
ORPT	Orotate phosphoribosyltransferase	(B488_06500)	Pyrimidine Metabolism
PANTS	Pantothenate synthase	(B488_11870)	Pantothenate and CoA Biosynthesis
PAPPT3	Phospho-N-acetylmuramoyl-pentapeptide-transferase (meso-2,6-diaminopimelate)	(B488_07140)	Peptidoglycan Biosynthesis
PDH	Pyruvate dehydrogenase	(B488_07400) or (B488_11040)	Glycolysis
PDX5POi	Pyridoxine 5-phosphate oxidase	(B488_02960)	Vitamin B6 Metabolism
PENTSOtex	PENTSOtex	(B488_11480)	Transport
PHETRS	Phenylalanyl-tRNA synthetase	(B488_10630) and (B488_10640)	Phenylalanine Tyrosine Tryptophan Biosynthesis
PMDPHT	Pyrimidine phosphatase	(B488_08800)	Riboflavin Metabolism
PPA	Inorganic diphosphatase	(B488_10200)	Oxidative Phosphorylation
PPBNGS	Porphobilinogen synthase	(B488_03540)	Porphyrin and Chlorophyll Metabolism
PPPGO	Protoporphyrinogen oxidase (aerobic)	(B488_08410)	Porphyrin and Chlorophyll Metabolism
PRAGSr	Phosphoribosylglycinamide synthase	(B488_08180)	Purine Metabolism
PRAIi	Phosphoribosylanthranilate isomerase (irreversible)	(B488_13090) or (B488_00850) or (B488_03030) or (B488_03020)	Phenylalanine Tyrosine Tryptophan Biosynthesis
PRASCSi	Phosphoribosylaminoimidazolesuccinocarboxamide synthase	(B488_05350)	Purine Metabolism
PRFGS	Phosphoribosylformylglycinamidine synthase	(B488_05310)	Purine Metabolism
PSP_L	Phosphoserine phosphatase (L-serine)	(B488_06380)	Glycine, Serine and threonine metabolism
RIBabcpp	D-ribose transport via ABC system (periplasm)	(B488_11480) or (B488_05640) or (B488_10540)	Transport
RPE	Ribulose 5-phosphate 3-epimerase	(B488_02730)	Pentose Phosphate Pathway
RPI	Ribose-5-phosphate isomerase	(B488_07390)	Pentose Phosphate Pathway
SDPTA	Succinyldiaminopimelate transaminase	(B488_01500)	Lysine Biosynthesis
SERAT	Serine O-acetyltransferase	(B488_03850)	Cysteine Metabolism
SERTRS	Seryl-tRNA synthetase	(B488_11110)	Glycine, Serine and threonine metabolism
SHCHF	Sirohydrochlorin ferrochelatase	(B488_09610)	Porphyrin and Chlorophyll Metabolism
SHK3Dr	Shikimate dehydrogenase	(B488_13740)	Phenylalanine Tyrosine Tryptophan Biosynthesis

SOTA	Succinylornithine transaminase	(B488_01500)	Arginine and Proline Metabolism
SUCD1	Succinate dehydrogenase	(B488_07490) and (B488_07480) and (B488_07500)	TCA Cycle
SUCDi	Succinate dehydrogenase (irreversible)	(B488_07490) and (B488_07480) and (B488_07500)	TCA Cycle
SULabcpp	Sulfate transport via ABC system (periplasm)	(B488_00260)	Sulfur Metabolism
TALA	Transaldolase	(B488_09590) or (B488_05910)	Pentose Phosphate Pathway
TAURabcpp	Taurine transport via ABC system (periplasm)	(B488_11480)	Transport
THRTRS	Threonyl-tRNA synthetase	(B488_06460)	Glycine, Serine and threonine metabolism
TPI	Triose-phosphate isomerase	(B488_04610)	Gluconeogenesis
TRPS1	Tryptophan synthase (indoleglycerol phosphate)	(B488_13070) or (B488_00380)	Phenylalanine Tyrosine Tryptophan Biosynthesis
TRPS2	Tryptophan synthase (indole)	(B488_13080) or (B488_09990)	Phenylalanine Tyrosine Tryptophan Biosynthesis
TSULabcpp	Thiosulfate transport via ABC system (periplasm)	(B488_10770)	Sulfur Metabolism
UPPN	B-ureidopropionase	(B488_04780)	Pantothenate and CoA Biosynthesis
VANKpp	VANKpp	(B488_06670) or (B488_05280)	Transport
VANLNtex	VANLNtex	(B488_06670) or (B488_05280)	Transport
VANLTtex	VANLTtex	(B488_06670) or (B488_05280)	Transport
VNLNpp	VNLNpp	(B488_06670) or (B488_05280)	Transport
pentso3abcpp	Pentso3abcpp	(B488_11480)	Transport
3PEPTabcpp	Tripeptide (LalaDgluMdap) transport via ABC system (periplasm)	((B488_03240) and (B488_03270) and (B488_03250) and (B488_03240) and (B488_03260)) or ((B488_10540) and (B488_10540)) or ((B488_11480) and (B488_08860))	Murein Recycling
4PEPTabcpp	Tetrapeptide (LalaDgluMdapDala) transport via ABC system (periplasm)	((B488_03260) and (B488_03240) and (B488_03240) and (B488_03270) and (B488_03250)) or ((B488_10540) and (B488_08860)) or ((B488_08860) and (B488_11480))	Murein Recycling

ACOLIPAabctex	Arabinose modified core oligosaccharide lipid A transport via ABC system (periplasm to extracellular)	((B488_02420) and (B488_02430) and (B488_06930) and (B488_13000) and (B488_13010) and (B488_02410) and (B488_11650))	Lipopolysaccharide Biosynthesis / Recycling
ADCS	4-amino-4-deoxychorismate synthase	((B488_05820) and (B488_10810)) or (B488_13110)	Cofactor and Prosthetic Group Biosynthesis
AGM3PApp	N-Acetyl-D-glucosamine(anhydrous)N-Acetylmuramyl-tripeptide amidase (periplasm)	(B488_00740)	Murein Recycling
AGM4PApp	N-Acetyl-D-glucosamine(anhydrous)N-Acetylmuramyl-tetrapeptide amidase (periplasm)	(B488_00740)	Murein Recycling
ALAALAabcpp	D-alanyl-D-alanine (DalaDala) transport via ABC system (periplasm)	((B488_03270) and (B488_03250) and (B488_03260) and (B488_03240)) or ((B488_03260) and (B488_10540)) or (B488_06080) or (B488_08860)	Transport, Inner Membrane
ALATA_D2	D-alanine transaminase	(B488_10890)	Cofactor and Prosthetic Group Biosynthesis
ALATA_L2	Alanine transaminase	(B488_10890)	Cofactor and Prosthetic Group Biosynthesis
ALAabcpp	L-alanine transport via ABC system (periplasm)	((B488_10260) and (B488_10280) and (B488_10270) and (B488_00950)) or (B488_00980) or ((B488_05380) and (B488_10290))	Transport, Inner Membrane
ASCBptspp	L-ascorbate transport via PEP:Pyr PTS (periplasm)	((B488_13600) and (B488_02780) and (B488_06020))	Transport, Inner Membrane
CGLYabcpp	L-Cysteinylglycine (Cys-Gly) transport via ABC system (periplasm)	((B488_03250) and (B488_03270) and (B488_03240) and (B488_10540)) or ((B488_03260) and (B488_10540))	Transport, Inner Membrane
CLIPAabctex	Cold lipid A transport via ABC system (periplasm to extracellular)	(B488_02430 and B488_13000 and B488_02420) or (B488_10260) or (B488_08860) or (B488_10150) or (B488_11480)	Lipopolysaccharide Biosynthesis / Recycling
CMtpp	Chloramphenicol transport via TolC system	((B488_09880) and (B488_09870) and (B488_09860)) or (B488_13370) or (B488_09950) or (B488_05630) or (B488_13390)	Transport, Outer Membrane
COLIPAPabctex	Core oligosaccharide lipid A diphosphate transport via ABC system (periplasm to extracellular)	((B488_02430) and (B488_06930) and (B488_13010) and (B488_13000) and (B488_02420))	Lipopolysaccharide Biosynthesis / Recycling

COLIPAabctex	Core oligosaccharide lipid A transport via ABC system (periplasm to extracellular)	((B488_02430) and (B488_13010) and (B488_06930) and (B488_13000) and (B488_02420))	Lipopolysaccharide Biosynthesis / Recycling
CRNDabcpp	D-carnitine transport via ABC system (periplasm)	(B488_02250)	Transport, Inner Membrane
CYSSADS	L-cysteine sulfinic acid desulfurase	(B488_08710))	Cysteine Metabolism
CYTBD2pp	Cytochrome oxidase bd (menaquinol-8: 2 protons) (periplasm)	(B488_10770) or ((B488_11480) and (B488_10780))	Oxidative Phosphorylation
DOXRBCNtpp	Doxorubicin transport via TolC system	((B488_09860) and (B488_09870) and (B488_09880)) or ((B488_05630) and (B488_13370)) or ((B488_13390) and (B488_09950)) or (B488_13380)	Transport, Outer Membrane
ECA4COLIPAabctex	Enterobacterial common antigen (x4) core oligosaccharide lipid A transport via ABC system (periplasm to extracellular)	((B488_13010) and (B488_02420) and (B488_06930) and (B488_13000) and (B488_02430))	Lipopolysaccharide Biosynthesis / Recycling
ENLIPAabctex	Phosphoethanolamine lipid A transport via ABC system (periplasm to extracellular)	((B488_06930) and (B488_13000) and (B488_02420) and (B488_02430) and (B488_13010))	Lipopolysaccharide Biosynthesis / Recycling
FUSAtpp	Fusidic acid transport via TolC system	((B488_09880) and (B488_09860) and (B488_09870)) or (B488_13370)	Transport, Outer Membrane
GRXR	Glutaredoxin reductase	(B488_05290)	Unassigned
GTHRDabcpp	Reduced glutathione via ABC system (periplasm)	((B488_03260) and (B488_03250) and (B488_03270) and (B488_03240)) or (B488_10540) or (B488_08860)	Transport, Inner Membrane
HPPK2	6-hydroxymethyl-dihydropterin pyrophosphokinase	(B488_08090)	Cofactor and Prosthetic Group Biosynthesis
INDOLEt2pp	Indole transport via proton symport, irreversible (periplasm)	(B488_09880)	Transport, Inner Membrane
K2L4Aabctex	KDO(2)-lipid IV A transport via ABC system (periplasm to extracellular)	(B488_05650)	Lipopolysaccharide Biosynthesis / Recycling
KAS15	Beta-ketoacyl-ACP synthase (2)	(B488_10100) or (B488_09730) or (B488_09740)	Membrane Lipid Metabolism
KDOPS	3-deoxy -D-manno-octulosonic -acid 8-phosphate synthase	(B488_10980)	Lipopolysaccharide Biosynthesis / Recycling
LIPAabctex	Lipid A transport via ABC system (periplasm to extracellular)	(B488_05650)	Lipopolysaccharide Biosynthesis / Recycling

MALTHXabcpp	Maltohexaose transport via ABC system (periplasm)	(B488_10770) and (B488_10780) and (B488_10790)	Transport, Inner Membrane
MALTPTabcpp	Maltopentaose transport via ABC system (periplasm)	(B488_10770) and (B488_10780) and (B488_10790)	Transport, Inner Membrane
MALTTRabcpp	Maltotriose transport via ABC system (periplasm)	(B488_10770) and (B488_10780) and (B488_10790)	Transport, Inner Membrane
MALTTTRabcpp	Maltotetraose transport via ABC system (periplasm)	(B488_10770) and (B488_10780) and (B488_10790)	Transport, Inner Membrane
MALTabcpp	Maltose transport via ABC system (periplasm)	(B488_10770) and (B488_10780) and (B488_10790)	Transport, Inner Membrane
MINCYCtpp	Minocycline transport via TolC system	(B488_09870)	Transport, Outer Membrane
NADH17pp	NADH dehydrogenase (menaquinone-8 & 3 protons) (periplasm)	(B488_02570) and (B488_02580) and (B488_02590) and (B488_02600) and (B488_02610) and (B488_02620) and (B488_02630) and (B488_02640) and (B488_02650) and (B488_02660) and (B488_02670) and (B488_02680) and (B488_02690) and (B488_02700)	Oxidative Phosphorylation
NADH18pp	NADH dehydrogenase (demethylmenaquinone-8 & 3 protons) (periplasm)	(B488_02570) and (B488_02580) and (B488_02590) and (B488_02600) and (B488_02610) and (B488_02620) and (B488_02630) and (B488_02640) and (B488_02650) and (B488_02660) and (B488_02670) and (B488_02680) and (B488_02690) and (B488_02700)	Oxidative Phosphorylation
NADHHR	NADH hydratase	(B488_12180)	Metabolite Repair
NADHHS	NADH hydratase	(B488_12180)	Metabolite Repair
NADPHHR	NADPH hydratase	(B488_12180)	Metabolite Repair
NADPHHS	NADPH hydratase	(B488_12180)	Metabolite Repair
NADPHQR3	NADPH Quinone Reductase (Menaquinone-8)	(B488_05890)	Oxidative Phosphorylation
NOVBCNtpp	Novobiocin transport via TolC system	(B488_09870)	Transport, Outer Membrane
O16A4COLIPAabctex	O16 antigen (x4) core oligosaccharide lipid A transport via ABC system (periplasm to extracellular)	(B488_13000) and (B488_13010) and (B488_13020)	Lipopolysaccharide Biosynthesis / Recycling
OCTDPS	Octaprenyl pyrophosphate synthase	(B488_00690)	Cofactor and Prosthetic Group Biosynthesis

OMBZLM	2-Octaprenyl-6-methoxy-benzoquinol methylase	(B488_00690)	Cofactor and Prosthetic Group Biosynthesis
RFAMPtpp	Rifampin transport via TolC system	(B488_09870) or (B488_13370)	Transport, Outer Membrane
RNDR3b	Ribonucleoside-diphosphate reductase (CDP) (glutaredoxin)	(B488_03580) and (B488_03570)	Nucleotide Salvage Pathway
RNDR4b	Ribonucleoside-diphosphate reductase (UDP) (glutaredoxin)	(B488_02930)	Nucleotide Salvage Pathway
S2FE2SR	SUF [2Fe-2S] regeneration	(B488_08720) and (B488_02490) and (B488_08730) and (B488_08710)	Cofactor and Prosthetic Group Biosynthesis
S2FE2SS	SUF [2Fe-2S] Synthesis	(B488_08720) and (B488_02490) and (B488_08730) and (B488_08710)	Cofactor and Prosthetic Group Biosynthesis
S2FE2SS2	SUF [2Fe-2S] Synthesis II	(B488_08720) and (B488_02490) and (B488_08730) and (B488_08710)	Cofactor and Prosthetic Group Biosynthesis
S2FE2ST	SUF [2Fe-2S] Transfer	(B488_08720) and (B488_02490) and (B488_08730) and (B488_08710)	Cofactor and Prosthetic Group Biosynthesis
S4FE4SR	SUF [4Fe-4S] Reduction	(B488_08720) and (B488_02490) and (B488_08730) and (B488_08710)	Cofactor and Prosthetic Group Biosynthesis
S4FE4ST	SUF [4Fe-4S] Transfer	(B488_08720) and (B488_02490) and (B488_08730) and (B488_08710)	Cofactor and Prosthetic Group Biosynthesis
SCYSDS	SUF Cysteine desulfuration	(B488_08710)	Cofactor and Prosthetic Group Biosynthesis
TTRCYCtpp	Tetracycline transport via TolC system	(B488_09880)	Transport, Outer Membrane
UDCPDPS	Undecaprenyl diphosphate synthase	(B488_00440)	Cofactor and Prosthetic Group Biosynthesis
BUTSO3abcpp	Butanesulfonate transport via ABC system (periplasm)	((B488_11480) and (B488_10550) and (B488_10780))	Transport; Inner Membrane
FEex	Iron transporter	((B488_04500) and (B488_11150)) or (B488_10030)	iron metabolism
FEpp	Iron transporter	((B488_11150) and (B488_04500)) or (B488_10030) or (B488_08420)	iron metabolism
Kt3pp	Potassium transport out via proton antiport (periplasm)	(B488_06670) or (B488_09960)	Inorganic Ion Transport and Metabolism
SULFACabcpp	Sulfoacetate transport via ABC system (periplasm)	(B488_11480)	Transport; Inner Membrane
THZPSN	Thiazole phosphate synthesis	(B488_01030)	Cofactor and Prosthetic Group Biosynthesis

Supplementary Table 2. Reactions present in BT-1 and absent in *C* Las strains

Reaction ID	Reaction Name	Subsystem	General Subsystem
AASAD3	L-aminoadipate-semialdehyde dehydrogenase (NADH	Lysine Metabolism	Amino acid metabolism
AHSERL2	O acetylhomoserine thiol lyase	Methionine Metabolism	Amino acid metabolism
ALATA_L	L-alanine transaminase	Alanine and Aspartate Metabolism	Amino acid metabolism
ARGTRS	Arginyl-tRNA synthetase	Arginine and Proline Metabolism	Amino acid metabolism
BETALDHx	Betaine-aldehyde dehydrogenase	Glycine, Serine and threonine metabolism	Amino acid metabolism
CHOLSH	CHOLSH	Glycine, Serine and threonine metabolism	Amino acid metabolism
CYSTGL	Cystathionine g lyase	Methionine Metabolism	Amino acid metabolism
CYSTL	Cystathionine b-lyase	Methionine Metabolism	Amino acid metabolism
G2PP	Glycerol-2-phosphate phosphatase	Alternate Carbon Metabolism	Amino acid metabolism
G5SD	Glutamate-5-semialdehyde dehydrogenase	Arginine and Proline Metabolism	Amino acid metabolism
GLUR	Glutamate racemase	Glutamate Metabolism	Amino acid metabolism
HPYRRx	Hydroxypyruvate reductase (NADH)	Glycine, Serine and threonine metabolism	Amino acid metabolism
HSERTA	Homoserine O trans acetylase	Methionine Metabolism	Amino acid metabolism
LYSMO	LYSMO	Lysine Metabolism	Amino acid metabolism
METGL	Methionine g lyase	Methionine Metabolism	Amino acid metabolism
METS	Methionine synthase	Methionine Metabolism	Amino acid metabolism
METSOXR1	L-methionine-S-oxide reductase	Methionine Metabolism	Amino acid metabolism
METSOXR2	L-methionine-R-sulfoxide reductase	Methionine Metabolism	Amino acid metabolism
MMTSAO	(S)-Methylmalonate semialdehyde:NAD+ oxidoreduct	Valine, leucine and isoleucine degradation	Amino acid metabolism
MTRPOX	N-methyltryptophan oxidase	Glycine and Serine Metabolism	Amino acid metabolism
OXPTNDH	Glutarate-semialdehyde:NAD+ oxidoreductase	Lysine Metabolism	Amino acid metabolism
P5CD	1-pyrroline-5-carboxylate dehydrogenase	Arginine and Proline Metabolism	Amino acid metabolism
P5CR	Pyrroline-5-carboxylate reductase	Arginine and Proline Metabolism	Amino acid metabolism
PGCD	Phosphoglycerate dehydrogenase	Glycine, Serine and threonine metabolism	Amino acid metabolism
PROD2	Proline dehydrogenase	Arginine and Proline Metabolism	Amino acid metabolism
PROt2rpp	L-proline reversible transport via proton symport (peri	Arginine and Proline Metabolism	Amino acid metabolism
SARCOX	Sarcosine oxidase	Glycine and Serine Metabolism	Amino acid metabolism
SGSAD	Succinylglutamic semialdehyde dehydrogenase	Arginine and Proline Metabolism	Amino acid metabolism
SHSL1	O-succinylhomoserine lyase (L-cysteine)	Methionine Metabolism	Amino acid metabolism
SLCYSS	O-acetyl-L-serine sulfhydrylase	Cysteine Metabolism	Amino acid metabolism
TRPS1	Tryptophan synthase (indoleglycerol phosphate)	Phenylalanine Tyrosine Tryptophan Biosynthe	Amino acid metabolism
TRPTRS	Tryptophanyl-tRNA synthetase	tRNA Charging	Amino acid metabolism

FBA	Fructose-bisphosphate aldolase	Glycolysis	Carbohydrate metabolism
FBP	Fructose-bisphosphatase	Glycolysis	Carbohydrate metabolism
FRD2	Fumarate reductase	Citric Acid Cycle	Carbohydrate metabolism
FRD3	Fumarate reductase	Citric Acid Cycle	Carbohydrate metabolism
FRUpts	D-fructose transport via PEP:Pyr PTS	Fructose Metabolism	Carbohydrate metabolism
G6PI	Glucose 6 phosphate isomerase	Pentose Phosphate Pathway	Carbohydrate metabolism
GADktpp	GADktpp	Glycolysis	Carbohydrate metabolism
LDH_D	D-lactate dehydrogenase	Pyruvate Met	Carbohydrate metabolism
PGLCNDH	PGLCNDH	Glycolysis	Carbohydrate metabolism
14GLUCANabcpp	1,4-alpha-D-glucan transport via ABC system (peripla	Transport, Inner Membrane	Cell envelope
ALAALAabcpp	D-alanyl-D-alanine (DalaDala) transport via ABC syst	Transport, Inner Membrane	Cell envelope
ALAabcpp	L-alanine transport via ABC system (periplasm)	Transport, Inner Membrane	Cell envelope
CGLYabcpp	L-Cysteinylglycine (Cys-Gly) transport via ABC syste	Transport, Inner Membrane	Cell envelope
CMtpp	Chloramphenicol transport via TolC system	Transport, Outer Membrane	Cell envelope
CRNDt2rpp	D-carnitine outward transport (H+ antiport)	Transport; Inner Membrane	Cell envelope
CRNt2rpp	L-carnitine outward transport (H+ antiport)	Transport; Inner Membrane	Cell envelope
CTBTt2rpp	Cronobetaine outward transport (H+ antiport)	Transport; Inner Membrane	Cell envelope
DOXRBCNtpp	Doxorubicin transport via TolC system	Transport, Outer Membrane	Cell envelope
EAR100x	Enoyl-[acyl-carrier-protein] reductase (NADH) (n-C1(	Cell Envelope Biosynthesis	Cell envelope
EAR121x	Enoyl-[acyl-carrier-protein] reductase (NADH) (n-C12	Cell Envelope Biosynthesis	Cell envelope
EAR180x	Enoyl-[acyl-carrier-protein] reductase (NADH) (n-C18	Cell Envelope Biosynthesis	Cell envelope
FUSAtpp	Fusidic acid transport via TolC system	Transport, Outer Membrane	Cell envelope
GTHRDabcpp	Reduced glutathione via ABC system (periplasm)	Transport, Inner Membrane	Cell envelope
INDOLEt2pp	Indole transport via proton symport, irreversible (perip	Transport, Inner Membrane	Cell envelope
MALTHXabcpp	Maltohexaose transport via ABC system (periplasm)	Transport, Inner Membrane	Cell envelope
MALTPTabcpp	Maltopentaose transport via ABC system (periplasm)	Transport, Inner Membrane	Cell envelope
MALTTRabcpp	Maltotriose transport via ABC system (periplasm)	Transport, Inner Membrane	Cell envelope
MALTTTRabcpp	Maltotetraose transport via ABC system (periplasm)	Transport, Inner Membrane	Cell envelope
MALTabcpp	Maltose transport via ABC system (periplasm)	Transport, Inner Membrane	Cell envelope
METDabcpp	D-methionine transport via ABC system (periplasm)	Transport, Inner Membrane	Cell envelope
MI1PP	Myo-inositol 1-phosphatase	Cell Envelope Biosynthesis	Cell envelope
MINCYCtpp	Minocycline transport via TolC system	Transport, Outer Membrane	Cell envelope
NOVBCNtpp	Novobiocin transport via TolC system	Transport, Outer Membrane	Cell envelope
PROGLYabcpp	L-Prolinylglycine (Pro-Gly) transport via ABC system	Transport, Inner Membrane	Cell envelope

RFAMPtpp	Rifampin transport via TolC system	Transport, Outer Membrane	Cell envelope
SULFACabcpp	Sulfoacetate transport via ABC system (periplasm)	Transport; Inner Membrane	Cell envelope
TTRCYCtpp	Tetracycline transport via TolC system	Transport, Outer Membrane	Cell envelope
CYTBD2pp	Cytochrome oxidase bd (menaquinol-8: 2 protons) (pe	Oxidative Phosphorylation	Energy metabolism
CYTBDpp	Cytochrome oxidase bd (ubiquinol-8: 2 protons) (perip	Oxidative Phosphorylation	Energy metabolism
GLU5K	Glutamate 5-kinase	Urea cycle/amino group metabolism	Energy metabolism
NADPHQR3	NADPH Quinone Reductase (Menaquinone-8)	Oxidative Phosphorylation	Energy metabolism
NADPHQR4	NADPH Quinone Reductase (2-Demethylmenaquinon	Oxidative Phosphorylation	Energy metabolism
GLYALDDr	D-Glyceraldehyde dehydrogenase	Glycerophospholipid Metabolism	Fatty acid metabolism
GLYALDtpp	Glyceraldehyde facilitated diffusion (periplasm)	Glycerophospholipid Metabolism	Fatty acid metabolism
3PEPTabcpp	Tripeptide (LalaDgluMdap) transport via ABC system	Murein Recycling	Glycan biosynthesis and metabolism
4PEPTabcpp	Tetrapeptide (LalaDgluMdapDala) transport via ABC	Murein Recycling	Glycan biosynthesis and metabolism
AGM3PApp	N-Acetyl-D-glucosamine(anhydrous)N-Acetylmuramy	Murein Recycling	Glycan biosynthesis and metabolism
AGM4PApp	N-Acetyl-D-glucosamine(anhydrous)N-Acetylmuramy	Murein Recycling	Glycan biosynthesis and metabolism
KDOCT2	3-deoxy-manno-octulosonate cytidylyltransferase	Lipopolysaccharide Biosynthesis / Recycling	Glycan biosynthesis and metabolism
UHGADA	UDP-3-O-acetylglucosamine deacetylase	Lipopolysaccharide Biosynthesis / Recycling	Glycan biosynthesis and metabolism
ADCS	4-amino-4-deoxychorismate synthase	Cofactor and Prosthetic Group Biosynthesis	Metabolism of cofactors and vitamins
AMPMS2	4-amino-2-methyl-5-phosphomethylpyrimidine synthe	Thiamine Metabolism	Metabolism of cofactors and vitamins
ASP1DC	Aspartate 1-decarboxylase	Cofactor and Prosthetic Group Biosynthesis	Metabolism of cofactors and vitamins
DHPS	Dihydropteroate synthase	Folate Biosynthesis	Metabolism of cofactors and vitamins
DHPS2	Dihydropteroate synthase	Cofactor and Prosthetic Group Biosynthesis	Metabolism of cofactors and vitamins
HMPK1	Hydroxymethylpyrimidine kinase (ATP)	Thiamine Metabolism	Metabolism of cofactors and vitamins

HPPK2	6-hydroxymethyl-dihydropterin pyrophosphokinase	Cofactor and Prosthetic Group Biosynthesis	Metabolism of cofactors and vitamins
NADDP	NAD diphosphatase	Cofactor and Prosthetic Group Biosynthesis	Metabolism of cofactors and vitamins
NMNDA	Nicotinamide-nucleotide amidase	Cofactor and Prosthetic Group Biosynthesis	Metabolism of cofactors and vitamins
NNAM	Nicotinamidase	Cofactor and Prosthetic Group Biosynthesis	Metabolism of cofactors and vitamins
THZPSN	Thiazole phosphate synthesis	Cofactor and Prosthetic Group Biosynthesis	Metabolism of cofactors and vitamins
UDCPDPS	Undecaprenyl diphosphate synthase	Cofactor and Prosthetic Group Biosynthesis	Metabolism of cofactors and vitamins
NADPHQR2	NADPH Quinone Reductase (Ubiquinone-8)	others	Metabolism of other amino acids
ADK3	Adentylate kinase (GTP)	Nucleotide Salvage Pathway	Nucleotide metabolism
ADK4	Adentylate kinase (ITP)	Nucleotide Salvage Pathway	Nucleotide metabolism
ADNK1	Adenosine kinase	Nucleotide Salvage Pathway	Nucleotide metabolism
GTPDPDP	Guanosine-5-triphosphate,3-diphosphate diphosphatas	Purine Metabolism	Nucleotide metabolism
GTPDPK	GTP diphosphokinase	Purine Metabolism	Nucleotide metabolism
GUAPRT	Guanine phosphoribosyltransferase	Purine Metabolism	Nucleotide metabolism
PPGPPDP	Guanosine-3,5-bis(diphosphate) 3-diphosphatase	Purine Metabolism	Nucleotide metabolism
3OXOADPt_pp	3OXOADPt LPAREN pp RPAREN	B-Ketoadipate pathway	Xenobiotics biodegradation and metabolism
COALDDH	COALDDH	B-Ketoadipate pathway	Xenobiotics biodegradation and metabolism
VNDH	VNDH	B-Ketoadipate pathway	Xenobiotics biodegradation and metabolism
VNDH_2	4-hydroxybenzaldehyde dehydrogenase	B-Ketoadipate pathway	Xenobiotics biodegradation and metabolism
VNDH_3	VNDH 3	B-Ketoadipate pathway	Xenobiotics biodegradation and metabolism

Supplementary Table 3. Culture media compositions and imposed constraints

Components	Reaction ID	BM-7 (mg/L)	M13 (mg/L)	M14 (mg/L)	M15 (mg/L)
**Inorganic salts**					
Calcium chloride dehydrate		165	1320	1320	1320
Magnesium chloride anhydrous		1068.2	1068.2	1068.2	1068.2
Ammonium sulfate anhydrous		98	1356.7	1356.7	1356.7
Potassium chloride	EX_so4_e	3750	2240	2240	2240
Sodium phosphate monobasic monohydrate	EX_pi_e	125	1007	1007	1007
**Amino acids**					
L/β-alanine	EX_ala__L_e	447.25	447.25	447.25	447.25
L-arginine-HCl	EX_arg__L_e	1777	1777	1777	1777
L-asparagine monohydrate	EX_asp__L_e	1075.45	1075.45	1075.45	1075.45
L-aspartic acid	EX_asn__L_e	818.6	818.6	818.6	818.6
L-cystine-2HCl	EX_cys__L_e	56.38	56.38	56.38	56.38
L-glutamic acid	EX_glu__L_e	1502.2	1502.2	1502.2	1502.2
L-glutamine	EX_glu__L_e	358.04	358.04	358.04	358.04
Glycine	EX_gly_e	859.512	859.512	859.512	859.512
L-histidine hydrochloride monohydrate	EX_his__L_e	2366.11	2366.11	2366.11	2366.11
L-isoleucine	EX_ile__L_e	687.36	687.36	687.36	687.36
L-leucine	EX_leu__L_e	592.89	592.89	592.89	592.89
L-lysine-HCl	EX_lys__L_e	1464.85	1464.85	1464.85	1464.85
L-methionine	EX_met__L_e	678.9	678.9	678.9	678.9
L-phenylalanine	EX_phe__L_e	789.62	789.62	789.62	789.62
L-proline	EX_pro__L_e	940.61	940.61	940.61	940.61
DL-serine	EX_ser__L_e	944.76	944.76	944.76	944.76
L-threonine	EX_thr__L_e	459.8	459.8	459.8	459.8
L-tryptophan	EX_trp__L_e	373.73	373.73	373.73	373.73
L-tyrosine disodium salt	EX_tyr__L_e	391.37	391.37	391.37	391.37
L-valine	EX_val__L_e	644.31	644.31	644.31	644.31
Betaine	EX_glyb_e	0.31	0.31	0.31	0.31
Ornithine	EX_orn_e	229.96	229.96	229.96	229.96
Methionine sulfoxide		18.2	18.2	18.2	18.2
**Vitamins**					
Biotin	EX_btn_e	0	0.1	0.1	0.1
Choline Chloride		0	1000	1000	1000
Folic Acid		0	0.2	0.2	0.2
Myo-Inositol	EX_ins_e	0	0.2	0.2	0.2
Niacin	EX_nac_e	0	0.2	0.2	0.2
D-Calcium pantothenate	EX_pnto__R_e	0	0.2	0.2	0.2

Para-aminobenzoic acid (PABA)	EX_4abz_e	0	0.2	0.2	0.2
Pyrodoxine-HCl		0	0.2	0.2	0.2
Riboflavin	EX_ribflv_e	0	0.2	0.2	0.2
Thiamine-HCl	EX_thm_e	0	0.2	0.2	0.2
Urea	EX_urea_e	290	0	0	0
**Organic acids**					
Citric acid	EX_cit_e	0	0	0	2500
a-ketoglutarate	EX_akg_e	2000	0	0	0
ACES		10000	0	0	0
malate	EX_mal__L_e	0	0	4.64	4.64
fumarate	EX_fum_e	0	0	4.8	4.8
maltose		0	0	616	616
fructose	EX_fru_e	0	0	293	293
succinate	EX_succ_e	0	0	8.27	8.27
Glucose	EX_glc__D_e	1250	0	5667	5667

Reaction ID*	BM-7 (mmol/gDW/h)	M13 (mmol/gDW/h)	M14 (mmol/gDW/h)	M15 (mmol/gDW/h)	Roots (mmol/gDW/h)
EX_glc__D_e	-1.500	0.000	-1.500	-1.500	-10
EX_so4_e	-100.000	-100.000	-100.000	-100.000	-100
EX_pi_e	-100.000	-100.000	-100.000	-100.000	-100
EX_ala__L_e	-0.130	-0.519	-0.130	-0.130	0
EX_arg__L_e	-0.516	-2.063	-0.516	-0.516	0
EX_asp__L_e	-0.212	-0.212	-0.212	-0.212	0
EX_asn__L_e	-0.238	-0.950	-0.238	-0.238	0
EX_cys__L_e	-0.016	-0.065	-0.016	-0.016	0
EX_glu__L_e	-0.436	-1.744	-0.436	-0.436	0
EX_gln__L_e	-0.104	-0.104	-0.104	-0.104	0
EX_gly_e	-0.249	-0.998	-0.249	-0.249	0
EX_his__L_e	-0.687	-2.747	-0.687	-0.687	0
EX_ile__L_e	-0.199	-0.798	-0.199	-0.199	0
EX_leu__L_e	-0.172	-0.688	-0.172	-0.172	0
EX_lys__L_e	-0.425	-1.700	-0.425	-0.425	0
EX_met__L_e	-0.197	-0.788	-0.197	-0.197	0
EX_phe__L_e	-0.229	-0.917	-0.229	-0.229	0
EX_pro__L_e	-0.273	-1.092	-0.273	-0.273	0
EX_ser__L_e	-0.274	-0.174	-0.274	-0.274	0
EX_thr__L_e	-0.133	-0.534	-0.133	-0.133	0
EX_trp__L_e	-0.108	-0.434	-0.108	-0.108	0
EX_tyr__L_e	-0.114	-0.454	-0.114	-0.114	0
EX_val__L_e	-0.187	-0.748	-0.187	-0.187	0
EX_glyb_e	-0.050	-0.200	-0.050	-0.050	0
EX_orn_e	-0.250	-0.250	-0.250	-0.250	0
EX_btn_e	0.000	-0.010	-0.010	-0.010	0
EX_ins_e	0.000	-0.020	-0.020	-0.020	0
EX_nac_e	0.000	-0.020	-0.020	-0.020	0
EX_pnto__R_e	0.000	-0.020	-0.020	-0.020	0
EX_4abz_e	0.000	-0.020	-0.020	-0.020	0
EX_ribflv_e	0.000	-0.020	-0.020	-0.020	0
EX_thm_e	0.000	-0.020	-0.020	-0.020	-10
EX_urea_e	-0.200	0.000	0.000	0.000	0
EX_cit_e	0.000	0.000	0.000	-0.150	0
EX_akg_e	-0.350	0.000	0.000	0.000	0
EX_mal__L_e	0.000	0.000	-0.010	-0.010	0
EX_fum_e	0.000	0.000	-0.010	-0.010	0

EX_fru_e	0.000	0.000	-0.200	-0.200	-10
EX_succ_e	0.000	0.000	-0.020	-0.020	0
EX_cobalt2_e	-100.000	-100.000	-100.000	-100.000	-100
EX_fe2_e	-100.000	-100.000	-100.000	-100.000	-100
EX_h2_e	-100.000	-100.000	-100.000	-100.000	-100
EX_h2o_e	-100.000	-100.000	-100.000	-100.000	-100
EX_h_e	-100.000	-100.000	-100.000	-100.000	-100
EX_na1_e	-100.000	-100.000	-100.000	-100.000	-100
EX_nh4_e	-100.000	-100.000	-100.000	-100.000	-5
EX_o2_e	-0.015	-0.015	-0.015	-0.015	-100
EX_no3_e	0	0	0	0	-100

*Uptake rates were calculated based on experimentally observed maximum citrate consumption rates of 0.15 mmol/gDW/h. This uptake rate calculated for 13 mM of citrate in M15 culture media, when the culture achieved OD=0.65 in 285 h. Each metabolite uptake rate was adjusted based on their concentration in the culture media and molecular weight

$$13\ mM\ = \frac{13\ mmol}{L} \cdot \frac{192\ mg}{1\ mmol} \cdot \frac{1}{0.3\ \frac{gDW}{L}} \cdot \frac{1}{285\ h} = 30\ \frac{mg}{gDW\ h} = 0.15\ \frac{mmol}{gDW\ h}$$

Supplementary Table 4. Connectivity analysis by metabolite in the culture medium

	Degree of connectivity							Pricted beneficial fraction by metabolite						
**Metabolite**	**CLas A4**	**CLas FL17**	**CLas gxpsy**	**CLas Ishi-1**	**CLas psy62**	**CLas YCPsy**	***L. crescens* BT-1**	**CLas A4**	**CLas FL17**	**CLas gxpsy**	**CLas Ishi-1**	**CLas psy62**	**CLas YCPsy**	***L. crescens* BT-1**
Glc-D	2	2	2	2	2	2	4	1.00	1.00	1.00	1.00	1.00	1.00	0.50
Pi	149	127	127	127	127	127	134	0.26	0.31	0.31	0.31	0.31	0.31	0.26
Ala-L	8	8	8	8	8	8	11	0.25	0.25	0.25	0.25	0.25	0.25	0.09
Arg-L	5	5	5	5	5	5	6	0.60	0.60	0.60	0.60	0.60	0.60	0.33
Asp-L	14	14	14	14	14	14	15	0.64	0.64	0.64	0.64	0.64	0.64	0.53
Asn-L	3	3	3	3	3	3	3	0.67	0.67	0.67	0.67	0.67	0.67	0.67
Cys-L	8	8	8	8	8	8	10	0.25	0.25	0.25	0.25	0.25	0.25	0.20
Glu-L	35	35	35	35	35	35	39	0.66	0.66	0.71	0.66	0.66	0.66	0.56
Gln-L	17	17	17	17	17	17	18	0.53	0.53	0.65	0.53	0.53	0.53	0.56
Gly	12	12	12	12	12	12	12	0.50	0.50	0.50	0.50	0.50	0.50	0.92
His-L	3	3	3	3	3	3	3	0.67	0.67	0.67	0.67	0.67	0.67	0.67
Ile-L	4	4	4	4	4	4	4	0.50	0.50	0.50	0.50	0.50	0.50	0.50
Leu-L	4	4	4	4	4	4	4	0.50	0.50	0.50	0.50	0.50	0.50	0.50
Lys-L	4	4	4	4	4	4	5	0.50	0.50	0.50	0.50	0.50	0.50	0.40
Met-L	8	8	7	8	8	8	12	0.25	0.25	0.29	0.25	0.25	0.25	0.25
Phe-L	4	4	4	4	4	4	4	0.50	0.50	0.50	0.50	0.50	0.50	0.50
Pro-L	5	5	6	5	5	5	7	0.40	0.40	0.33	0.40	0.40	0.40	0.29
Ser-L	14	14	14	14	14	14	15	0.71	0.71	0.71	0.71	0.71	0.71	0.60
Thr-L	6	6	6	6	6	6	6	0.67	0.67	0.67	0.67	0.67	0.67	0.50
Trp-L	3	3	3	3	3	3	6	0.67	0.67	0.67	0.67	0.67	0.67	0.17
Tyr-L	4	4	4	4	4	4	5	0.50	0.50	0.50	0.50	0.50	0.50	0.40
Val-L	4	4	4	4	4	4	4	0.50	0.50	0.50	0.50	0.50	0.50	0.50
Glyb	2	2	2	2	2	2	2	0.00	0.00	0.00	0.00	0.00	0.00	0.00
Orn	6	6	6	6	6	6	6	0.67	0.67	0.67	0.67	0.67	0.67	0.67
Btn	3	3	3	3	3	3	4	0.00	0.00	0.00	0.00	0.00	0.00	0.00
Ins	2	2	2	2	2	2	1	0.00	0.00	0.00	0.00	0.00	0.00	0.00
Nac	2	2	2	2	2	2	2	0.00	0.00	0.00	0.00	0.00	0.00	0.00

Pnto-R	3	3	3	3	3	3	4	0.67	0.67	0.67	0.67	0.67	0.67	0.50
4abz	3	3	3	3	3	3	5	0.67	0.67	0.67	0.67	0.67	0.67	0.40
Ribflv	3	3	3	3	3	3	2	0.67	0.67	0.67	0.67	0.67	0.67	0.00
Thm	2	2	2	2	2	2	2	0.00	0.00	0.00	0.00	0.00	0.00	0.00
Urea	3	3	3	3	3	3	3	0.33	0.33	0.33	0.33	0.33	0.33	0.00
Cit	4	4	4	4	4	4	4	0.25	0.25	0.25	0.25	0.25	0.25	0.00
Akg	19	19	19	19	19	19	19	0.68	0.68	0.68	0.68	0.68	0.68	0.63
Mal-L	5	5	3	5	5	5	5	0.20	0.20	0.67	0.20	0.20	0.20	0.00
Fum	8	8	8	8	8	8	10	0.50	0.50	0.50	0.50	0.50	0.50	0.30
Fru	1	1	1	1	1	1	2	0.00	0.00	0.00	0.00	0.00	0.00	0.00
Succ	9	9	9	9	9	9	12	0.67	0.67	0.67	0.67	0.67	0.67	0.33

Supplementary Table 5. Preprocessing results of RNA-sequencing data (metadata)

Sample	Sample ID	BioProject number	BioSample number	Raw Reads	After_Trim	Bacterial rRNA abundance	After Trim rRNA
Phloem from cultivar seven	S2	PRJNA509215	SAMN1057417	30672168	30631057	81.08%	5794478
	S23	PRJNA509215	SAMN1057418	1908993688	1903100504	80.10%	94670523
Phloem from cultivar washington	S7	PRJNA509215	SAMN1057418	24922041	22780749	56.80%	9840815
	S8	PRJNA509215	SAMN1057418	8456409	8372787	30.36%	5831076
	S9	PRJNA509215	SAMN1057418	14144915	14086749	58.66%	5823961
Phloem from cultivar tango	S21	PRJNA509215	SAMN1057418	1829659200	1767973740	50.23%	219961284
	S22	PRJNA509215	SAMN1057418	508233228	475811112	25.99%	88042503
ACP alimentary canals	S19	PRJNA509215	SAMN1057418	47653173	47205209	72.15%	13146872
	S20	PRJNA509215	SAMN1057418	38361432	36064109	26.52%	26499381

Citrus (%)	After Trim Citrus	After Trim psyllid	CLas psy62 (%)	Reads CLas psy62	CLas A4 (%)	Reads CLas A4	CLas FL17 (%)	Reads CLas FL17	CLas gxpsy (%)
78.32%	1256133	1256133	0.39%	4853	0.38%	4761	0.38%	4811	0.40%
85.19%	14017480	14017480	0.52%	72858	0.51%	71088	0.51%	72050	0.53%
83.97%	1577319	1577319	0.12%	1961	0.13%	1985	0.12%	1956	0.13%
80.60%	1131307	1131307	0.16%	1769	0.16%	1780	0.16%	1767	0.16%
83.09%	984932	984932	0.14%	1338	0.14%	1351	0.14%	1332	0.14%
91.47%	18761011	18761011	0.13%	24434	0.13%	24456	0.13%	24296	0.13%
92.75%	6380258	6380258	0.48%	30726	0.48%	30902	0.48%	30640	0.49%
0.39%	13095939	4245182	1.69%	71842	1.69%	71917	1.69%	71695	1.70%
0.33%	26412346	6014036	3.33%	200151	3.33%	200487	3.32%	199679	3.35%

Reads CLas gxpsy	CLas YCPsy (%)	Reads CLas YCPsy	CLas Ishi-1 (%)	Reads CLas Ishi-1	CLas BT-1 (%)	Reads CLas BT-1
6288	0.38%	4792	0.33%	4100	0.08%	1036
74516	0.51%	71756	0.44%	62255	0.12%	16447
2854	0.12%	1959	0.12%	1903	0.04%	626
3048	0.16%	1774	0.15%	1724	0.03%	354
2250	0.14%	1343	0.13%	1306	0.03%	305
24792	0.13%	24261	0.12%	22803	0.04%	7835
31101	0.48%	30705	0.47%	30241	0.08%	4983
72363	1.69%	71717	1.64%	69789	0.23%	9879
201433	3.32%	199945	3.21%	193218	0.40%	23758

Supplementary Table 6. Analysis of RNA-sequencing data

Unique essential genes			Unique no essential genes		
**Gene**	**Subsystem**	**Reaction ID**	**Gene**	**Subsystem**	**Reaction ID**
***C* Las A4**			***C* Las A4**		
VBICanLib130745_0017	Glycolysis	ME1	VBICanLib130745_0192	Methionine Metabolism	FMETTRS
VBICanLib130745_0058	Transport	ARGabcpp	VBICanLib130745_0396	TCA Cycle	ICL
VBICanLib130745_0085	Oxidative Phosphorylation	CYTBO3_4pp	VBICanLib130745_0528	Pentose Phosphate Pathway	GND
VBICanLib130745_0171	Purine and Pyrimidine Biosynthesis	DHORD2	VBICanLib130745_0564	Glycine, Serine and threonine metabolism	SERTRS
VBICanLib130745_0173	Gluconeogenesis	PGM	VBICanLib130745_0850	Glycine, Serine and threonine metabolism	GLYCK
VBICanLib130745_0190	Urea cycle/amino group metabolism	AGPR	VBICanLib130745_1000	TCA Cycle	AKGDH
VBICanLib130745_0225	Urea cycle/amino group metabolism	ACGS	***C* Las FL17**		
VBICanLib130745_0319	Nucleotide Salvage Pathway	RNDR1b	RH08_00340	Purine Metabolism	ADSL2r
VBICanLib130745_0325	Phenylalanine Tyrosine Tryptophan [illegible]	ANPRT	RH08_01630	TCA Cycle	ACONT
VBICanLib130745_0355	Gluconeogenesis	PYK	RH08_01780	TCA Cycle	ICL
VBICanLib130745_0371	Nicotinate and Nicotinamide	NAMNPP	RH08_02935	Oxidative Phosphorylation	ATPS4rpp
VBICanLib130745_0402	Urea cycle/amino group metabolism	ACGK	RH08_02940	Oxidative Phosphorylation	ATPS4rpp
VBICanLib130745_0464	Nucleotide Salvage Pathway	URIDK2r	RH08_02945	Oxidative Phosphorylation	ATPS4rpp
VBICanLib130745_0477	Nucleotide Salvage Pathway	RNDR4b	RH08_02950	Oxidative Phosphorylation	ATPS4rpp
VBICanLib130745_0501	Transport	FERtex	RH08_02955	Oxidative Phosphorylation	ATPS4rpp
VBICanLib130745_0549	Glutamate Metabolism	GTHS	RH08_03970	TCA Cycle	ICDHyr
VBICanLib130745_0592	Gluconeogenesis	ENO	RH08_04000	Transport	CITtex
VBICanLib130745_0593	Gluconeogenesis	PGK	RH08_04040	Glycine, Serine and threonine metabolism	GLYCK
VBICanLib130745_0689	Pentose Phosphate Pathway	TKT1	RH08_04645	TCA Cycle	SUCD1
VBICanLib130745_0690	Transport	FE2abc	RH08_04675	TCA Cycle	SUCOAS
VBICanLib130745_0691	Transport	CHLabcpp	RH08_04685	TCA Cycle	AKGDa
VBICanLib130745_0745	Fatty Acid Biosynthesis	EAR141y	RH08_04855	Oxidative Phosphorylation	ATPS4rpp
VBICanLib130745_0746	Glycolysis	PGL	***C* Las gxpsy**		
VBICanLib130745_0747	Glycolysis	G6PBDH	VBICanLib238577_0375	TCA Cycle	ICL
VBICanLib130745_0753	Fatty Acid Biosynthesis	ACOATA	VBICanLib238577_0964	TCA Cycle	SUCD1
VBICanLib130745_0785	Methane Metabolism	FDH	VBICanLib238577_0965	Alanine and Aspartate Metabolism	ASPO1
VBICanLib130745_0786	Methane Metabolism	FDH	VBICanLib238577_0966	TCA Cycle	SUCD1
VBICanLib130745_0787	Transport	ETHSO3abcpp	VBICanLib238577_0973	TCA Cycle	AKGDa
VBICanLib130745_0814	Urea cycle/amino group metabolism	UREA	***C* Las Ishi-1**		
VBICanLib130745_0823	Transport	THRabcpp	Ishi_547	Transport	FUMtex

VBICanLib130745_0855	Glycolysis	G6PDH2r
VBICanLib130745_0879	Glycine, Serine and threonine metabo	GHMT2r
VBICanLib130745_0900	Glycine, Serine and threonine metabo	PSP_L
VBICanLib130745_0909	Transport	ICITtex
VBICanLib130745_0910	Unassigned	GRXR
VBICanLib130745_0946	Nicotinate and Nicotinamide	NADS1
VBICanLib130745_0989	Arginine and Proline Metabolism	ORNCD
VBICanLib130745_0997	TCA Cycle	MDH
VBICanLib130745_1112	Transport	LEUabcpp
***C* Las FL17**		
***C* Las gxpsy**		
VBICanLib238577_0068	Oxidative Phosphorylation	CYTBO3_4pp
VBICanLib238577_0156	Purine and Pyrimidine Biosynthesis	DHORD2
VBICanLib238577_0158	Gluconeogenesis	PGM
VBICanLib238577_0175	Urea cycle/amino group metabolism	AGPR
VBICanLib238577_0209	Urea cycle/amino group metabolism	ACGS
VBICanLib238577_0325	Glutamate Metabolism	ABTA
VBICanLib238577_0335	Gluconeogenesis	PYK
VBICanLib238577_0350	Nicotinate and Nicotinamide	NAMNPP
VBICanLib238577_0381	Urea cycle/amino group metabolism	ACGK
VBICanLib238577_0439	Pentose Phosphate Pathway	TKT2
VBICanLib238577_0444	Peptidoglycan Biosynthesis	UGMDDS
VBICanLib238577_0457	Nucleotide Salvage Pathway	RNDR4b
VBICanLib238577_0569	Vitamin B6 Metabolism	E4PD
VBICanLib238577_0570	Gluconeogenesis	PGK
VBICanLib238577_0719	One Carbon pool by folate	MTHFC
VBICanLib238577_0720	Glycolysis	PGL
VBICanLib238577_0727	Fatty Acid Biosynthesis	ACOATA
VBICanLib238577_0758	Methane Metabolism	FDH
VBICanLib238577_0759	Methane Metabolism	FDH
VBICanLib238577_0760	Transport	ETHSO3abcpp
VBICanLib238577_0825	Pentose Phosphate Pathway	TKT1
VBICanLib238577_0839	Valine, Leucine, and Isoleucine Meta	ILETRS
VBICanLib238577_0850	Glutamate Metabolism	GF6PTA
Ishi_559	Transport	FUMt2_2pp
Ishi_974	TCA Cycle	SUCD1
Ishi_975	Alanine and Aspartate Metabolism	ASPO1
Ishi_976	TCA Cycle	SUCD1
Ishi_982	TCA Cycle	AKGDH
Ishi_986	Glycine, Serine and threonine metabolism	GLYCL
**CLas psy62**		
VBICanLib64949_0386	TCA Cycle	ICL
VBICanLib64949_0525	Pentose Phosphate Pathway	GND
VBICanLib64949_0562	Glycine, Serine and threonine metabolism	SERTRS
VBICanLib64949_0837	Glycine, Serine and threonine metabolism	GLYCK
VBICanLib64949_0986	TCA Cycle	AKGDH
***C* Las YCPsy**		
AL011_00280	Purine Metabolism	ADSL2r
AL011_00345	Transport	FUMt2_2pp
AL011_01575	TCA Cycle	ACONT
AL011_01725	TCA Cycle	ICL
AL011_03900	TCA Cycle	ICDHyr
AL011_03930	Transport	CITtex
AL011_03970	Glycine, Serine and threonine metabolism	GLYCK
AL011_04590	TCA Cycle	SUCD1
AL011_04620	TCA Cycle	SUCOAS
AL011_04630	TCA Cycle	AKGDa

VBICanLib238577_0879	Transport	ICITtex
VBICanLib238577_0880	Vitamin B6 Metabolism	PDX5POi
VBICanLib238577_0932	Transport	HISabcpp
VBICanLib238577_1180	Lysine Metabolism	LYSabcpp
VBICanLib238577_1189	Glutathione Metabolism	GTHPi
***C* Las Ishi-1**		
Ishi_157	Purine and Pyrimidine Biosynthesis	DHORD2
Ishi_159	Gluconeogenesis	PGM
Ishi_176	Urea cycle/amino group metabolism	AGPR
Ishi_212	Urea cycle/amino group metabolism	ORNTAC
Ishi_308	Phenylalanine Tyrosine Tryptophan I	ANPRT
Ishi_331	Pyrimidine Metabolism	DHORDi
Ishi_339	Gluconeogenesis	PYK
Ishi_349	TCA Cycle	ACONT
Ishi_353	Nicotinate and Nicotinamide	NAMNPP
Ishi_371	Gluconeogenesis	ENO
Ishi_372	Gluconeogenesis	ENO
Ishi_373	Oxidative Phosphorylation	CYO1b
Ishi_374	Oxidative Phosphorylation	CYO1b
Ishi_397	Nucleotide Salvage Pathway	RNDR1b
Ishi_462	Nucleotide Salvage Pathway	RNDR4b
Ishi_525	Transport	CHLabcpp
Ishi_579	One Carbon pool by folate	GARFT
Ishi_580	Gluconeogenesis	PGK
Ishi_60	Transport	GLUabcpp
Ishi_667	Pentose Phosphate Pathway	TKT2
Ishi_69	Transport	CYSabcpp
Ishi_7	Fatty Acid Biosynthesis	3OAR100
Ishi_72	Oxidative Phosphorylation	CYTBO3_4pp
Ishi_730	Fatty Acid Biosynthesis	EAR141y
Ishi_732	Glycolysis	G6PBDH
Ishi_738	Fatty Acid Biosynthesis	ACOATA
Ishi_810	Transport	ILEabcpp
Ishi_839	Glycolysis	G6PDH2r

Ishi_84	Valine, Leucine, and Isoleucine Meta	ACHBS
Ishi_863	Glycine, Serine and threonine metabo	GHMT2r
Ishi_898	Pentose Phosphate Pathway	RPE
Ishi_938	Nicotinate and Nicotinamide	NADS1
Ishi_979	TCA Cycle	MDH
***C* Las psy62**		
VBICanLib64949_0016	Glycolysis	ME1
VBICanLib64949_0058	Transport	ARGabcpp
VBICanLib64949_0084	Oxidative Phosphorylation	CYTBO3_4pp
VBICanLib64949_0168	Purine and Pyrimidine Biosynthesis	DHORD2
VBICanLib64949_0170	Gluconeogenesis	PGM
VBICanLib64949_0187	Urea cycle/amino group metabolism	AGPR
VBICanLib64949_0221	Urea cycle/amino group metabolism	ACGS
VBICanLib64949_0312	Nucleotide Salvage Pathway	RNDR1b
VBICanLib64949_0317	Phenylalanine Tyrosine Tryptophan I	ANPRT
VBICanLib64949_0346	Gluconeogenesis	PYK
VBICanLib64949_0361	Nicotinate and Nicotinamide	NAMNPP
VBICanLib64949_0458	Nucleotide Salvage Pathway	URIDK2r
VBICanLib64949_0471	Nucleotide Salvage Pathway	RNDR4b
VBICanLib64949_0547	Glutamate Metabolism	GTHS
VBICanLib64949_0589	Gluconeogenesis	ENO
VBICanLib64949_0590	Gluconeogenesis	PGK
VBICanLib64949_0684	Pentose Phosphate Pathway	TKT1
VBICanLib64949_0685	Transport	FE2abc
VBICanLib64949_0686	Transport	CHLabcpp
VBICanLib64949_0737	Fatty Acid Biosynthesis	EAR141y
VBICanLib64949_0738	Glycolysis	PGL
VBICanLib64949_0739	Glycolysis	G6PBDH
VBICanLib64949_0745	Fatty Acid Biosynthesis	ACOATA
VBICanLib64949_0774	Methane Metabolism	FDH
VBICanLib64949_0775	Methane Metabolism	FDH
VBICanLib64949_0776	Transport	ETHSO3abcpp
VBICanLib64949_0803	Urea cycle/amino group metabolism	UREA
VBICanLib64949_0842	Glycolysis	G6PDH2r

VBICanLib64949_0864	Glycine, Serine and threonine metab	GHMT2r
VBICanLib64949_0886	Glycine, Serine and threonine metab	PSP_L
VBICanLib64949_0896	Transport	ICITtex
VBICanLib64949_0897	Unassigned	GRXR
VBICanLib64949_0929	Nicotinate and Nicotinamide	NADS1
VBICanLib64949_0983	TCA Cycle	MDH
VBICanLib64949_1093	Transport	LEUabcpp
VBICanLib64949_1154	Glutathione Metabolism	GTHPi
***C* Las YCPsy**		
AL011_00015	Transport	ARGabcpp
AL011_00190	Transport	GLCabcpp
AL011_00710	Purine and Pyrimidine Biosynthesis	DHORD2
AL011_00720	Gluconeogenesis	PGM
AL011_00800	Urea cycle/amino group metabolism	AGPR
AL011_00965	Urea cycle/amino group metabolism	ACGS
AL011_01265	Transport	ACt2rpp
AL011_01395	Phenylalanine Tyrosine Tryptophan I	ANPRT
AL011_01535	Gluconeogenesis	PYK
AL011_01595	Nicotinate and Nicotinamide	NAMNPP
AL011_01755	Urea cycle/amino group metabolism	ACGK
AL011_01775	Glutamate Metabolism	ABTA
AL011_02155	Transport	HISabcpp
AL011_02400	Pentose Phosphate Pathway	GND
AL011_02685	One Carbon pool by folate	FTHFD
AL011_02690	Vitamin B6 Metabolism	E4PD
AL011_02695	Gluconeogenesis	PGK
AL011_03430	Fatty Acid Biosynthesis	EAR141y
AL011_03435	Glycolysis	PGL
AL011_03440	Glycolysis	G6PBDH
AL011_03470	Fatty Acid Biosynthesis	3OAS181
AL011_03655	Methane Metabolism	FDH
AL011_03660	Methane Metabolism	FDH
AL011_03665	Transport	ETHSO3abcpp
AL011_03815	Urea cycle/amino group metabolism	UREA

AL011_03995	Glycolysis	G6PDH2r
AL011_04105	Glycine, Serine and threonine metabo	GHMT2r
AL011_04215	Transport	ICITtex
AL011_04220	Vitamin B6 Metabolism	PDX5POi
AL011_04380	Nicotinate and Nicotinamide	NADS1
AL011_04580	Arginine and Proline Metabolism	ORNCD
AL011_04615	TCA Cycle	MDH
AL011_05135	Transport	LEUabcpp
AL011_05400	Glutathione Metabolism	GTHPi

Supplementary Table 7. Gene essentiality by strain and subsystem

Subsystem	Essential genes/no-growth						No-essential genes/ wild type growth					
	**A4**	**FL-17**	**gxpsy**	**Ishi-1**	**psy62**	**YCPsy**	**A4**	**FL-17**	**gxpsy**	**Ishi-1**	**psy62**	**YCPsy**
Vitamin b6 metabolism	0	0	2	0	0	2	0	0	0	0	0	0
Valine, leucine, and isoleucine metaboli	0	0	1	1	0	0	0	0	0	0	0	0
Urea cycle/amino group metabolism	4	0	3	2	3	4	0	0	0	0	0	0
Unassigned	1	0	0	0	1	0	0	0	0	0	0	0
Transport	8	0	3	4	6	7	0	1	0	2	0	2
Tca cycle	1	0	0	2	1	1	2	6	4	3	2	6
Pyrimidine metabolism	0	0	0	1	0	0	0	0	0	0	0	0
Purine and pyrimidine biosynthesis	1	0	1	1	1	1	0	0	0	0	0	0
Purine metabolism	0	0	0	0	0	0	0	1	0	0	0	1
Phenylalanine tyrosine tryptophan biosy	1	0	0	1	1	1	0	0	0	0	0	0
Peptidoglycan biosynthesis	0	0	1	0	0	0	0	0	0	0	0	0
Pentose phosphate pathway	1	0	2	2	1	1	1	0	0	0	1	0
Oxidative phosphorylation	1	0	1	3	1	0	0	6	0	0	0	0
One carbon pool by folate	0	0	1	1	0	1	0	0	0	0	0	0
Nucleotide salvage pathway	3	0	1	2	3	0	0	0	0	0	0	0
Nicotinate and nicotinamide	2	0	1	2	2	2	0	0	0	0	0	0
Methionine metabolism	0	0	0	0	0	0	1	0	0	0	0	0
Methane metabolism	2	0	2	0	2	2	0	0	0	0	0	0
Lysine metabolism	0	0	1	0	0	0	0	0	0	0	0	0
Glycolysis	4	0	1	2	4	3	0	0	0	0	0	0
Glycine, serine and threonine metabolis	2	0	0	1	2	1	2	1	0	1	2	1
Glutathione metabolism	0	0	1	0	1	1	0	0	0	0	0	0
Glutamate metabolism	1	0	2	0	1	1	0	0	0	0	0	0
Gluconeogenesis	4	0	3	5	4	3	0	0	0	0	0	0
Fatty acid biosynthesis	2	0	1	3	2	2	0	0	0	0	0	0
Arginine and proline metabolism	1	0	0	0	0	1	0	0	0	0	0	0
Alanine and aspartate metabolism	0	0	0	0	0	0	0	0	1	1	0	0

Supplementary Table 8. Gene essentiality benchmarking

Gene	Experimental (Lai et al., 2016)	Probability of having 1 insertion (TnS)	Predicted growth	Phenotype
B488_00070	No essential	0	0.909658	TP
B488_00180	No essential	0	1	TP
B488_00190	No essential	0	1	TP
B488_00210	No essential	0	1	TP
B488_00260	No essential	0	1	TP
B488_00370	No essential	0	1	TP
B488_00380	No essential	0	0	FN
B488_00420	No essential	0	0	FN
B488_00440	Essential	0.942804928	0	TN
B488_00450	Essential	0.950782957	0	TN
B488_00490	Essential	0.841889893	0	TN
B488_00500	Essential	0.958615955	0	TN
B488_00540	No essential	0	0	FN
B488_00550	Essential	0.945388514	0	TN
B488_00560	No essential	0	0	FN
B488_00600	No essential	0	0	FN
B488_00630	No essential	0	0	FN
B488_00690	No essential	0	0	FN
B488_00710	No essential	0	0	FN
B488_00740	No essential	0	0	FN
B488_00810	No essential	0	1	TP
B488_00820	No essential	0	0	FN
B488_00830	No essential	0	0	FN
B488_00840	Essential	0.765815875	0	TN
B488_00850	No essential	0	0	FN
B488_00950	No essential	0	0	FN
B488_00970	No essential	0	0	FN
B488_00980	No essential	0	0	FN
B488_00990	No essential	0	0	FN
B488_01030	No essential	0	0	FN
B488_01060	Essential	0.949046764	0	TN
B488_01070	No essential	0	0	FN
B488_01080	Essential	0.860760087	0	TN
B488_01100	No essential	0	0	FN
B488_01110	No essential	0	0	FN
B488_01130	Essential	0.993180375	0	TN
B488_01160	No essential	0	1	TP
B488_01170	No essential	0	0	FN
B488_01190	No essential	0	0	FN
B488_01200	No essential	0	0	FN
B488_01240	No essential	0	0	FN
B488_01270	No essential	0	0	FN
B488_01280	No essential	0	0	FN

B488_01290	No essential	0	0	FN
B488_01310	No essential	0	0	FN
B488_01360	No essential	0	0.997544	TP
B488_01370	Essential	0.944753768	0	TN
B488_01400	No essential	0	0	FN
B488_01440	No essential	0	0	FN
B488_01450	No essential	0	0	FN
B488_01480	Essential	0.984859176	0	TN
B488_01490	No essential	0	0	FN
B488_01500	No essential	0	0	FN
B488_01620	No essential	0.991406541	0	TN
B488_01670	No essential	0	0	FN
B488_01700	No essential	0	0	FN
B488_01740	No essential	0	0	FN
B488_01750	No essential	0	0	FN
B488_01920	No essential	0	0	FN
B488_02090	No essential	0	0	FN
B488_02110	Essential	0.995502027	0	TN
B488_02160	No essential	0	0	FN
B488_02180	No essential	0	0	FN
B488_02230	No essential	0	0	FN
B488_02240	No essential	0	0	FN
B488_02250	No essential	0	0	FN
B488_02350	Essential	0.980699757	0	TN
B488_02390	Essential	0.98047539	0	TN
B488_02410	No essential	0	0	FN
B488_02420	Essential	0.97858399	0	TN
B488_02430	No essential	0	0	FN
B488_02460	Essential	0.822525214	0	TN
B488_02470	No essential	0	0	FN
B488_02490	No essential	0	0	FN
B488_02530	No essential	0	0	FN
B488_02570	No essential	0	0	FN
B488_02580	No essential	0	0	FN
B488_02590	No essential	0	0	FN
B488_02600	No essential	0	1	TP
B488_02610	No essential	0	1	TP
B488_02620	Essential	0.992344656	0	TN
B488_02630	No essential	0	0	FN
B488_02640	No essential	0	0	FN
B488_02650	No essential	0	0	FN
B488_02660	Essential	0.903797358	0	TN
B488_02670	No essential	0	0	FN
B488_02680	No essential	0	0	FN
B488_02690	No essential	0	0	FN
B488_02700	No essential	0	0	FN
B488_02730	No essential	0.994331978	0	TN

B488_02750	Essential	0.927934188	0	TN
B488_02760	Essential	0.945388514	0	TN
B488_02770	No essential	0	0	FN
B488_02780	No essential	0	0	FN
B488_02850	Essential	0.902679311	0	TN
B488_02930	No essential	0	0	FN
B488_02950	Essential	0.960485436	2.08E-11	FP
B488_02960	No essential	0	0.134067	TP
B488_02970	Essential	0.994950775	0.440686	FP
B488_03020	Essential	0.636744737	0.463523	FP
B488_03030	No essential	0	0.712066	TP
B488_03110	No essential	0	0.712066	TP
B488_03130	No essential	0	0.712066	TP
B488_03160	No essential	0	0.712066	TP
B488_03240	No essential	0	0.712066	TP
B488_03250	No essential	0	0.712066	TP
B488_03260	No essential	0	0.719179	TP
B488_03270	No essential	0	0.7371	TP
B488_03310	No essential	0	0.77091	TP
B488_03320	Essential	0.811971015	0.787909	FP
B488_03410	No essential	0	0.853169	TP
B488_03540	No essential	0	0.908908	TP
B488_03570	No essential	0	0.909658	TP
B488_03580	No essential	0	0.946485	TP
B488_03630	Essential	0.983771887	0.946762	FP
B488_03640	No essential	0	0.955232	TP
B488_03690	No essential	0	0.958038	TP
B488_03820	No essential	0	0.958038	TP
B488_03850	No essential	0	0.958759	TP
B488_03910	No essential	0	0.963355	TP
B488_03920	No essential	0	0.964767	TP
B488_03930	No essential	0	0.971943	TP
B488_03980	No essential	0	0.971943	TP
B488_04000	Essential	0.768505843	0.971943	FP
B488_04010	No essential	0	0.971943	TP
B488_04310	Essential	0.89569286	0.985692	FP
B488_04410	No essential	0	0.985692	TP
B488_04420	No essential	0	0.985692	TP
B488_04500	No essential	0	0.985692	TP
B488_04520	No essential	0.999943844	0.985808	FP
B488_04530	No essential	0	0.987744	TP
B488_04610	No essential	0	0.987968	TP
B488_04630	Essential	0.998216317	0.987968	FP
B488_04640	Essential	0.990573947	0.987968	FP
B488_04780	No essential	0	0.989873	TP
B488_04950	No essential	0	0.991914	TP
B488_05190	No essential	0	0.991914	TP

B488_05250	Essential	0.959561495	0.99697	FP
B488_0528	No essential	0	0.997269	TP
B488_05280	No essential	0	0.997621	TP
B488_05290	No essential	0	0.999142	TP
B488_05310	No essential	0	0.999703	TP
B488_05350	No essential	0	0.999977	TP
B488_05360	No essential	0	1	TP
B488_05380	No essential	0	1	TP
B488_05410	No essential	0	1	TP
B488_05430	No essential	0	1	TP
B488_05450	Essential	0.82857125	1	FP
B488_05510	No essential	0	1	TP
B488_05630	No essential	0	1	TP
B488_05640	No essential	0	1	TP
B488_05650	No essential	0	1	TP
B488_05690	No essential	0	1	TP
B488_05700	No essential	0.991982367	1	FP
B488_05820	No essential	0	1	TP
B488_05870	No essential	0	1	TP
B488_05890	No essential	0	1	TP
B488_05900	No essential	0	1	TP
B488_05910	No essential	0	1	TP
B488_05940	No essential	0	1	TP
B488_05980	No essential	0	1	TP
B488_06010	No essential	0	1	TP
B488_06020	No essential	0	1	TP
B488_06060	No essential	0	1	TP
B488_06080	No essential	0	1	TP
B488_06090	No essential	0	1	TP
B488_06120	Essential	0.920954631	1	FP
B488_06130	No essential	0	1	TP
B488_06150	No essential	0	1	TP
B488_06340	Essential	0.953006219	1	FP
B488_06350	No essential	0	1	TP
B488_06380	Essential	0.967156257	1	FP
B488_06400	No essential	0	1	TP
B488_06410	No essential	0	1	TP
B488_06460	No essential	0	1	TP
B488_06500	Essential	0.929580588	1	FP
B488_06510	Essential	0.981783506	1	FP
B488_06600	No essential	0	1	TP
B488_06670	No essential	0.91127225	1	FP
B488_06680	No essential	0	1	TP
B488_06750	No essential	0	1	TP
B488_06870	No essential	0	1	TP
B488_06880	Essential	0.994892064	1	FP
B488_06930	No essential	0	1	TP

B488_06970	Essential	0.969000339	1	FP
B488_06980	No essential	0	1	TP
B488_07080	Essential	0.91024106	1	FP
B488_07100	No essential	0	1	TP
B488_07110	No essential	0	1	TP
B488_07130	No essential	0	1	TP
B488_07140	No essential	0	1	TP
B488_07150	No essential	0	1	TP
B488_07160	No essential	0	1	TP
B488_07290	No essential	0	1	TP
B488_07350	No essential	0	1	TP
B488_07360	Essential	0.994711804	1	FP
B488_07370	No essential	0	1	TP
B488_07380	No essential	0	1	TP
B488_07390	Essential	0.927096612	1	FP
B488_07400	No essential	0	1	TP
B488_07410	No essential	0	1	TP
B488_07420	Essential	0.999986932	1	FP
B488_07430	No essential	0	1	TP
B488_07440	No essential	0	1	TP
B488_07450	No essential	0	1	TP
B488_07480	Essential	0.950210899	1	FP
B488_07490	No essential	0	1	TP
B488_07500	Essential	0.768505843	1	FP
B488_07530	No essential	0	1	TP
B488_07550	No essential	0	1	TP
B488_07620	No essential	0	1	TP
B488_07630	No essential	0	1	TP
B488_07730	No essential	0	1	TP
B488_07750	No essential	0	1	TP
B488_08000	No essential	0	1	TP
B488_08030	Essential	0.92364784	1	FP
B488_08090	Essential	0.82857125	1	FP
B488_08110	Essential	0.965202496	1	FP
B488_08160	No essential	0	1	TP
B488_08180	No essential	0	1	TP
B488_08260	No essential	0	1	TP
B488_08280	No essential	0	1	TP
B488_08370	No essential	0	1	TP
B488_08390	No essential	0	1	TP
B488_08410	Essential	0.971077104	1	FP
B488_08420	No essential	0	1	TP
B488_08540	No essential	0	1	TP
B488_08590	No essential	0	1	TP
B488_08630	No essential	0	1	TP
B488_08640	No essential	0	1	TP
B488_08710	No essential	0	1	TP

B488_08720	Essential	0.989896988	1	FP
B488_08730	Essential	0.950782957	1	FP
B488_08800	No essential	0	1	TP
B488_08830	No essential	0	1	TP
B488_08840	No essential	0	1	TP
B488_08850	No essential	0	1	TP
B488_08860	No essential	0	1	TP
B488_09100	No essential	0	1	TP
B488_09110	No essential	0	1	TP
B488_09190	No essential	0	1	TP
B488_09260	Essential	0.936536224	1	FP
B488_09550	Essential	0.971299092	1	FP
B488_09560	No essential	0	1	TP
B488_09590	No essential	0	1	TP
B488_09610	No essential	0	1	TP
B488_09630	No essential	0	1	TP
B488_09680	No essential	0	1	TP
B488_09730	No essential	0	1	TP
B488_09740	No essential	0	1	TP
B488_09750	Essential	0.836313506	1	FP
B488_09760	No essential	0	1	TP
B488_09770	No essential	0	1	TP
B488_09780	No essential	0	1	TP
B488_09830	No essential	0.999340189	1	FP
B488_09860	No essential	0	1	TP
B488_09870	No essential	0	1	TP
B488_09880	No essential	0.995655355	1	FP
B488_09910	No essential	0	1	TP
B488_09920	No essential	0	1	TP
B488_09930	No essential	0	1	TP
B488_09950	No essential	0	1	TP
B488_09960	No essential	0	1	TP
B488_09970	No essential	0	1	TP
B488_09990	No essential	0	1	TP
B488_10000	No essential	0	1	TP
B488_10030	No essential	0	1	TP
B488_10070	No essential	0	1	TP
B488_10080	No essential	0	1	TP
B488_10100	No essential	0	1	TP
B488_10110	Essential	0.596944545	1	FP
B488_10120	No essential	0	1	TP
B488_10130	Essential	0.973934362	1	FP
B488_10150	Essential	0.954079907	1	FP
B488_10160	No essential	0	1	TP
B488_10200	Essential	0.868576115	1	FP
B488_10220	No essential	0	1	TP
B488_10230	Essential	0.875953473	1	FP

B488_10260	No essential	0	1	TP
B488_10270	No essential	0	1	TP
B488_10280	No essential	0	1	TP
B488_10290	No essential	0	1	TP
B488_10380	Essential	0.996963995	1	FP
B488_10420	No essential	0	1	TP
B488_10430	No essential	0	1	TP
B488_10530	No essential	0	1	TP
B488_10540	No essential	0	1	TP
B488_10550	No essential	0	1	TP
B488_10560	No essential	0	1	TP
B488_10590	No essential	0	1	TP
B488_10630	No essential	0	1	TP
B488_10640	No essential	0.984683156	1	FP
B488_10730	Essential	0.983958377	1	FP
B488_10740	No essential	0	1	TP
B488_10770	Essential	0.981783506	1	FP
B488_10780	No essential	0	1	TP
B488_10790	No essential	0	1	TP
B488_10810	No essential	0	1	TP
B488_10860	No essential	0.905995074	1	FP
B488_10870	No essential	0	1	TP
B488_10890	Essential	0.993180375	1	FP
B488_10950	Essential	0.993020868	1	FP
B488_10980	Essential	0.961388269	1	FP
B488_10990	No essential	0	1	TP
B488_11010	No essential	0	1	TP
B488_11020	No essential	0	1	TP
B488_11040	No essential	0	1	TP
B488_11050	No essential	0	1	TP
B488_11070	No essential	0	1	TP
B488_11110	No essential	0	1	TP
B488_11140	No essential	0	1	TP
B488_11150	No essential	0	1	TP
B488_11190	No essential	0	1	TP
B488_11240	Essential	0.971737973	1	FP
B488_11250	No essential	0	1	TP
B488_11320	Essential	0.986355079	1	FP
B488_11330	No essential	0	1	TP
B488_11350	Essential	0.974529955	1	FP
B488_11450	No essential	0	1	TP
B488_11480	No essential	0	1	TP
B488_11490	No essential	0	1	TP
B488_11520	No essential	0	1	TP
B488_11550	Essential	0.990241335	1	FP
B488_11560	Essential	0.972383744	1	FP
B488_11610	No essential	0	1	TP

B488_11640	Essential	0.9896607	1	FP
B488_11650	No essential	0	1	TP
B488_11750	No essential	0	1	TP
B488_11760	No essential	0	1	TP
B488_11770	No essential	0	1	TP
B488_11780	No essential	0	1	TP
B488_11790	No essential	0	1	TP
B488_11800	Essential	0.884261819	1	FP
B488_11870	No essential	0	1	TP
B488_11890	Essential	0.949632194	1	FP
B488_12030	No essential	0	1	TP
B488_12080	No essential	0	1	TP
B488_12090	No essential	0	1	TP
B488_12100	No essential	0	1	TP
B488_12160	No essential	0	1	TP
B488_12170	No essential	0	1	TP
B488_12180	No essential	0	1	TP
B488_12310	No essential	0	1	TP
B488_12320	No essential	0	1	TP
B488_12360	No essential	0	1	TP
B488_12440	No essential	0	1	TP
B488_12550	No essential	0	1	TP
B488_12610	Essential	0.955129068	1	FP
B488_12620	No essential	0	1	TP
B488_12630	No essential	0	1	TP
B488_12660	No essential	0	1	TP
B488_12670	No essential	0	1	TP
B488_12780	No essential	0	1	TP
B488_12880	No essential	0.610673531	1	FP
B488_12960	Essential	0.9857094	1	FP
B488_13000	Essential	0.92364784	1	FP
B488_13010	No essential	0	1	TP
B488_13020	No essential	0	1	TP
B488_13070	No essential	0	1	TP
B488_13080	No essential	0	1	TP
B488_13090	No essential	0	1	TP
B488_13100	No essential	0	1	TP
B488_13110	No essential	0	1	TP
B488_13120	No essential	0	1	TP
B488_13180	Essential	0.983958377	1	FP
B488_13230	Essential	0.967156257	1	FP
B488_13240	No essential	0	1	TP
B488_13270	No essential	0	1	TP
B488_13310	No essential	0	1	TP
B488_13360	No essential	0	1	TP
B488_13370	No essential	0	1	TP
B488_13380	No essential	0	1	TP

B488_13390	No essential	0	1	TP
B488_13490	No essential	0	1	TP
B488_13510	No essential	0	1	TP
B488_13520	No essential	0	1	TP
B488_13530	Essential	0.724703779	1	FP
B488_13540	Essential	0.975680632	1	FP
B488_13600	Essential	0.653148398	1	FP
B488_13650	No essential	0	1	TP
B488_13660	No essential	0	1	TP
B488_13670	No essential	0	1	TP
B488_13730	No essential	0	1	TP
B488_13740	No essential	0	1	TP
B488_13760	No essential	0	1	TP

Supplementary Table 9. Predicted strain-specific *C* Las genes potentially lethal useful for biocontrol

Culture Media			RNA-sequencing Citrus		
**Gene**	**Subsystem**	**Reaction**	**Gene**	**Subsystem**	**Reaction**
**A4**			**A4**		
CD16_RS00145	Purine Metabolism	AIRCr	CD16_RS0	Purine Metabolism	AIRCr
CD16_RS00505	Oxidative Phosphorylation	PPA	CD16_RS0	Oxidative Phosphorylation	PPA
CD16_RS00680	Fatty Acid Biosynthesis	ACCOAC	CD16_RS0	Fatty Acid Biosynthesis	ACCOAC
CD16_RS00750	Glycolysis	HEX1	CD16_RS0	Purine and Pyrimidine Biosynthesis	DHORD2
CD16_RS00855	Pantothenate and CoA Biosynthesis	DHPM1	CD16_RS0	Gluconeogenesis	PGM
CD16_RS01035	Alanine and Aspartate Metabolism	ASNS1	CD16_RS0	Glycolysis	HEX1
CD16_RS01090	Riboflavin Metabolism	RBFSb	CD16_RS0	Pantothenate and CoA Biosynthesis	DHPM1
CD16_RS01515	Pyrimidine Metabolism	DHORDi	CD16_RS0	Alanine and Aspartate Metabolism	ASNS1
CD16_RS01890	Folate Biosynthesis	DHFS	CD16_RS0	Riboflavin Metabolism	RBFSb
CD16_RS02925	Purine Metabolism	ADSS	CD16_RS0	Pyrimidine Metabolism	DHORDi
CD16_RS02930	Porphyrin and Chlorophyll Metabolism	SHCHF	CD16_RS0	Folate Biosynthesis	DHFS
CD16_RS03955	Lysine Biosynthesis	DHDPRy	CD16_RS0	Nucleotide Salvage Pathway	RNDR4b
CD16_RS04265	Purine Metabolism	PRASCSi	CD16_RS0	Purine Metabolism	ADSS
CD16_RS04880	Peptidoglycan Biosynthesis	UAGPT3	CD16_RS0	Porphyrin and Chlorophyll Metabolism	SHCHF
**FL-17**			CD16_RS0	Pentose Phosphate Pathway	TKT1
RH08_RS00180	Purine Metabolism	AIRCr	CD16_RS0	Lysine Biosynthesis	DHDPRy
RH08_RS00535	Oxidative Phosphorylation	PPA	CD16_RS0	Unassigned	GRXR
RH08_RS00710	Fatty Acid Biosynthesis	ACCOAC	CD16_RS0	Purine Metabolism	PRASCSi
RH08_RS00780	Glycolysis	HEX1	CD16_RS0	Arginine and Proline Metabolism	ORNCD
RH08_RS00885	Pantothenate and CoA Biosynthesis	DHPM1	CD16_RS0	Peptidoglycan Biosynthesis	UAGPT3
RH08_RS01065	Alanine and Aspartate Metabolism	ASNS1	CD16_RS0	Transport	LEUabcpp
RH08_RS01120	Riboflavin Metabolism	RBFSb	**FL-17**		
RH08_RS01545	Pyrimidine Metabolism	DHORDi	**gxpsy**		
RH08_RS01885	Pantothenate and CoA Biosynthesis	PNTK	WSI_RS00	Purine Metabolism	AIRCr
RH08_RS01920	Folate Biosynthesis	DHFS	WSI_RS00	Oxidative Phosphorylation	PPA
RH08_RS02965	Purine Metabolism	ADSS	WSI_RS00	Fatty Acid Biosynthesis	ACCOAC
RH08_RS02970	Porphyrin and Chlorophyll Metabolism	SHCHF	WSI_RS00	Purine and Pyrimidine Biosynthesis	DHORD2
RH08_RS04005	Lysine Biosynthesis	DHDPRy	WSI_RS00	Gluconeogenesis	PGM
RH08_RS04315	Purine Metabolism	PRAIS	WSI_RS00	Glycolysis	HEX1
RH08_RS04905	Peptidoglycan Biosynthesis	UAGPT3	WSI_RS00	Pantothenate and CoA Biosynthesis	DHPM1

**gxpsy**		
WSI_RS00110	Purine Metabolism	AIRCr
WSI_RS00480	Oxidative Phosphorylation	PPA
WSI_RS00655	Fatty Acid Biosynthesis	ACCOAC
WSI_RS00725	Glycolysis	HEX1
WSI_RS00830	Pantothenate and CoA Biosynthesis	DHPM1
WSI_RS01010	Alanine and Aspartate Metabolism	ASNS1
WSI_RS01070	Riboflavin Metabolism	RBFSb
WSI_RS01490	Pyrimidine Metabolism	DHORDi
WSI_RS01830	Pantothenate and CoA Biosynthesis	PNTK
WSI_RS01865	Folate Biosynthesis	DHFS
WSI_RS02900	Purine Metabolism	ADSS
WSI_RS02905	Porphyrin and Chlorophyll Metabolism	SHCHF
WSI_RS03160	Pyrimidine Metabolism	TMDS
WSI_RS03930	Lysine Biosynthesis	DHDPRy
WSI_RS04240	Purine Metabolism	PRAIS
**Ishi-1**		
CGUJ_RS00135	Purine Metabolism	AIRCr
CGUJ_RS00490	Oxidative Phosphorylation	PPA
CGUJ_RS00665	Fatty Acid Biosynthesis	ACCOAC
CGUJ_RS00735	Glycolysis	HEX1
CGUJ_RS01015	Purine metabolism	GLUPRT
CGUJ_RS01070	Riboflavin Metabolism	RBFSa
CGUJ_RS01870	Folate Biosynthesis	DHFS
CGUJ_RS02655	Nitrogen Metabolism	H2CO3D
CGUJ_RS02910	Purine Metabolism	ADSS
CGUJ_RS02915	Porphyrin and Chlorophyll Metabolism	SHCHD2
CGUJ_RS03950	Lysine Biosynthesis	DHDPRy
CGUJ_RS04230	Fatty Acid Biosynthesis	EAR180y
CGUJ_RS04255	Purine Metabolism	PRASCSi
**psy62**		
CLIBASIA_RS00180	Purine Metabolism	AIRCr
CLIBASIA_RS00540	Oxidative Phosphorylation	PPA
CLIBASIA_RS00715	Fatty Acid Biosynthesis	ACCOAC

WSI_RS01	Alanine and Aspartate Metabolism	ASNS1
WSI_RS01	Riboflavin Metabolism	RBFSb
WSI_RS01	Pyrimidine Metabolism	DHORDi
WSI_RS01	Pantothenate and CoA Biosynthesis	PNTK
WSI_RS01	Folate Biosynthesis	DHFS
WSI_RS02	Pentose Phosphate Pathway	TKT2
WSI_RS02	Nucleotide Salvage Pathway	RNDR4b
WSI_RS02	Purine Metabolism	ADSS
WSI_RS02	Porphyrin and Chlorophyll Metabolisn	SHCHF
WSI_RS03	Pyrimidine Metabolism	TMDS
WSI_RS03	TCA Cycle	ICDHyr
WSI_RS03	Lysine Biosynthesis	DHDPRy
WSI_RS04	Vitamin B6 Metabolism	PDX5POi
WSI_RS04	Purine Metabolism	PRAIS
**Ishi-1**		
CGUJ_RS(	Purine Metabolism	AIRCr
CGUJ_RS(	Oxidative Phosphorylation	PPA
CGUJ_RS(	Fatty Acid Biosynthesis	ACCOAC
CGUJ_RS(	Gluconeogenesis	PGM
CGUJ_RS(	Glycolysis	HEX1
CGUJ_RS(	Purine metabolism	GLUPRT
CGUJ_RS(	Riboflavin Metabolism	RBFSa
CGUJ_RS(	Pyrimidine Metabolism	DHORDi
CGUJ_RS(	Gluconeogenesis	ENO
CGUJ_RS(	Folate Biosynthesis	DHFS
CGUJ_RS(	Nucleotide Salvage Pathway	RNDR4b
CGUJ_RS(	Nitrogen Metabolism	H2CO3D
CGUJ_RS(	Purine Metabolism	ADSS
CGUJ_RS(	Porphyrin and Chlorophyll Metabolisn	SHCHD2
CGUJ_RS(	Lysine Biosynthesis	DHDPRy
CGUJ_RS(	Fatty Acid Biosynthesis	EAR180y
CGUJ_RS(	Purine Metabolism	PRASCSi
**psy62**		
CLIBASIA	Purine Metabolism	AIRCr

CLIBASIA_RS00785	Glycolysis	HEX1
CLIBASIA_RS00890	Pantothenate and CoA Biosynthesis	DHPM1
CLIBASIA_RS01070	Alanine and Aspartate Metabolism	ASNS1
CLIBASIA_RS01125	Riboflavin Metabolism	RBFSb
CLIBASIA_RS01550	Pyrimidine Metabolism	DHORDi
CLIBASIA_RS01925	Folate Biosynthesis	DHFS
CLIBASIA_RS02965	Purine Metabolism	ADSS
CLIBASIA_RS02970	Porphyrin and Chlorophyll Metabolism	SHCHF
CLIBASIA_RS04005	Lysine Biosynthesis	DHDPRy
CLIBASIA_RS04310	Purine Metabolism	PRASCSi
CLIBASIA_RS04895	Peptidoglycan Biosynthesis	UAGPT3
**YCPsy**		
AL011_RS00120	Purine Metabolism	AIRCr
AL011_RS00480	Oxidative Phosphorylation	PPA
AL011_RS00655	Fatty Acid Biosynthesis	ACCOAC
AL011_RS00725	Glycolysis	HEX1
AL011_RS00830	Pantothenate and CoA Biosynthesis	DHPM1
AL011_RS01010	Alanine and Aspartate Metabolism	ASNS1
AL011_RS01065	Riboflavin Metabolism	RBFSb
AL011_RS01490	Pyrimidine Metabolism	DHORDi
AL011_RS01685	Fatty Acid Biosynthesis	3HAD100
AL011_RS01865	Folate Biosynthesis	DHFS
AL011_RS02120	Fatty Acid Biosynthesis	3HAD120
AL011_RS02900	Purine Metabolism	ADSS
AL011_RS02905	Porphyrin and Chlorophyll Metabolism	SHCHF
AL011_RS03930	Lysine Biosynthesis	DHDPRy
AL011_RS04235	Purine Metabolism	PRAIS
AL011_RS04850	Peptidoglycan Biosynthesis	UAGPT3
**Summary**		

CLIBASIA	Oxidative Phosphorylation	PPA
CLIBASIA	Fatty Acid Biosynthesis	ACCOAC
CLIBASIA	Purine and Pyrimidine Biosynthesis	DHORD2
CLIBASIA	Gluconeogenesis	PGM
CLIBASIA	Glycolysis	HEX1
CLIBASIA	Pantothenate and CoA Biosynthesis	DHPM1
CLIBASIA	Alanine and Aspartate Metabolism	ASNS1
CLIBASIA	Riboflavin Metabolism	RBFSb
CLIBASIA	Pyrimidine Metabolism	DHORDi
CLIBASIA	Folate Biosynthesis	DHFS
CLIBASIA	Nucleotide Salvage Pathway	RNDR4b
CLIBASIA	Purine Metabolism	ADSS
CLIBASIA	Porphyrin and Chlorophyll Metabolism	SHCHF
CLIBASIA	Pentose Phosphate Pathway	TKT1
CLIBASIA	Lysine Biosynthesis	DHDPRy
CLIBASIA	Unassigned	GRXR
CLIBASIA	Purine Metabolism	PRASCSi
CLIBASIA	Peptidoglycan Biosynthesis	UAGPT3
CLIBASIA	Transport	LEUabcpp
**YCPsy**		
AL011_RS	Purine Metabolism	AIRCr
AL011_RS	Oxidative Phosphorylation	PPA
AL011_RS	Fatty Acid Biosynthesis	ACCOAC
AL011_RS	Purine and Pyrimidine Biosynthesis	DHORD2
AL011_RS	Gluconeogenesis	PGM
AL011_RS	Glycolysis	HEX1
AL011_RS	Pantothenate and CoA Biosynthesis	DHPM1
AL011_RS	Alanine and Aspartate Metabolism	ASNS1
AL011_RS	Riboflavin Metabolism	RBFSb
AL011_RS	Pyrimidine Metabolism	DHORDi
AL011_RS	Fatty Acid Biosynthesis	3HAD100
AL011_RS	Folate Biosynthesis	DHFS
AL011_RS	Fatty Acid Biosynthesis	3HAD120
AL011_RS	Transport	HISabcpp

AL011_RS	Purine Metabolism	ADSS
AL011_RS	Porphyrin and Chlorophyll Metabolism	SHCHF
AL011_RS	Lysine Biosynthesis	DHDPRy
AL011_RS	Vitamin B6 Metabolism	PDX5POi
AL011_RS	Purine Metabolism	PRAIS
AL011_RS	Arginine and Proline Metabolism	ORNCD
AL011_RS	Peptidoglycan Biosynthesis	UAGPT3
AL011_RS	Transport	LEUabcpp

Supplementary Table 10. List of reactions without Gene-Protein-Reaction associations

Common reactions across *C* Las models

Reaction ID	Reaction Name	Subsystem	Flux M15	Reaction Notes
GDBTALDH	GDBTALDH	Arginine and Proline Metabolism	0.01	Reaction added during gap filling
GUDBUTNAH	GUDBUTNAH	Arginine and Proline Metabolism	0.01	Reaction added during gap filling
OARGDC	OARGDC	Arginine and Proline Metabolism	0.01	Reaction added during gap filling
CYSS	Cysteine synthase	Cysteine Metabolism	0.00	Reaction added during gap filling
CFAS160E	Cyclopropane fatty acid synthase (Phosphatidylethanolamine, n-C16:0)	Fatty Acid Biosynthesis	0.00	Reaction added during gap filling, reaccion associated with pathogenic traits
CFAS160G	Cyclopropane fatty acid synthase (Phosphatidylglycerol, n-C16:0)	Fatty Acid Biosynthesis	0.00	Reaction added during gap filling, reaccion associated with pathogenic traits
CFAS180E	Cyclopropane fatty acid synthase (Phosphatidylethanolamine, n-C18:0)	Fatty Acid Biosynthesis	0.00	Reaction added during gap filling, reaccion associated with pathogenic traits
CFAS180G	Cyclopropane fatty acid synthase (Phosphatidylglycerol, n-C18:0)	Fatty Acid Biosynthesis	0.00	Reaction added during gap filling, reaccion associated with pathogenic traits
PABB	PABB	Folate Biosynthesis	0.00	Reaction found for L. BT-1 [GEM for BT-1 present work], not reference for Clas
DHNPA	Dihydroneopterin aldolase	Folate Biosynthesis	0.00	Reaction found for L. BT-1 [Lai etal., 2016/GEM for BT-1]
DNMPPA	Dihydroneopterin monophosphate dephosphorylase	Folate Biosynthesis	0.00	Reaction added during gap filling
FOLD3	Dihydropteroate synthase	Folate Biosynthesis	0.00	Reaction found for L. BT-1 [Lai etal., 2016/GEM for BT-1]
GCALDD	Glycolaldehyde dehydrogenase	Folate Biosynthesis	0.00	Reaction found for L. BT-1 [GEM for BT-1 present work], not reference for Clas
GLUDy	Glutamate dehydrogenase (NADP)	Glutamate Metabolism	0.00	Reaction added during gap filling
SSALy	Succinate-semialdehyde dehydrogenase (NADP)	Glutamate Metabolism	0.01	Reaction added during gap filling
PGPP120	Phosphatidylglycerol phosphate phosphatase (n-C14:0)	Glycerophospholipid Metabolism	0.00	Reaction added during gap filling, reaccion associated with pathogenic traits
PGPP160	Phosphatidylglycerol phosphate phosphatase (n-C16:0)	Glycerophospholipid Metabolism	0.00	Reaction added during gap filling, reaccion associated with pathogenic traits

PGPP161	Phosphatidylglycerol phosphate phosphatase (n-C16:1)	Glycerophospholipid Metabolism	0.00	Reaction added during gap filling, reaccion associated with pathogenic traits
PGPP180	Phosphatidylglycerol phosphate phosphatase (n-C18:0)	Glycerophospholipid Metabolism	0.00	Reaction added during gap filling, reaccion associated with pathogenic traits
PGPP181	Phosphatidylglycerol phosphate phosphatase (n-C18:1)	Glycerophospholipid Metabolism	0.00	Reaction added during gap filling, reaccion associated with pathogenic traits
PSD120	Phosphatidylserine decarboxylase (n-C12:0)	Glycerophospholipid Metabolism	0.00	Reaction added during gap filling, reaccion associated with pathogenic traits
PSD180	Phosphatidylserine decarboxylase (n-C18:0)	Glycerophospholipid Metabolism	0.00	Reaction added during gap filling, reaccion associated with pathogenic traits
PSSA120	Phosphatidylserine syntase (n-C12:0)	Glycerophospholipid Metabolism	0.00	Reaction found for L. BT-1 [GEM for BT-1 present work], not reference for Clas
PSSA180	Phosphatidylserine syntase (n-C18:0)	Glycerophospholipid Metabolism	0.00	Reaction found for L. BT-1 [GEM for BT-1 present work], not reference for Clas
PSD181	Phosphatidylserine decarboxylase (n-C18:1)	Glycerophospholipid Metabolism	0.00	Reaction added during gap filling, reaccion associated with pathogenic traits
PSSA181	Phosphatidylserine syntase (n-C18:1)	Glycerophospholipid Metabolism	0.00	Reaction found for L. BT-1 [GEM for BT-1 present work], not reference for Clas
SERD_L	L-serine deaminase	Glycine, Serine and threonine metabolism	-0.31	Reaction added during gap filling
THRD_L	L-threonine deaminase	Glycine, Serine and threonine metabolism	0.00	Reaction added during gap filling
PGI	Glucose-6-phosphate isomerase	Glycolysis	-0.06	Reaction added during gap filling
ALDD2x_copy2	Aldehyde dehydrogenase (acetaldehyde, NAD)	Glycolysis	0.00	Reaction added during gap filling
GLCtex	Glucose transport via diffusion (extracellular to periplasm)	Glycolysis	0.04	Reaction added during gap filling
HISTD	Histidinol dehydrogenase	Histidine Metabolism	0.00	Reaction found for L. BT-1 [GEM for BT-1 present work], not reference for Clas
HISTP	Histidinol-phosphatase	Histidine Metabolism	0.00	Reaction found for L. BT-1 [GEM for BT-1 present work], not reference for Clas
HSTPT	Histidinol-phosphate transaminase	Histidine Metabolism	0.00	Reaction found for L. BT-1 [GEM for BT-1 present work], not reference for Clas
IG3PS	Imidazole-glycerol-3-phosphate synthase	Histidine Metabolism	0.00	Reaction found for L. BT-1 [GEM for BT-1 present work], not reference for Clas

IGPDH	Imidazoleglycerol-phosphate dehydratase	Histidine Metabolism	0.00	Reaction found for L. BT-1 [GEM for BT-1 present work], not reference for Clas
PRAMPC	Phosphoribosyl-AMP cyclohydrolase	Histidine Metabolism	0.00	Reaction found for L. BT-1 [GEM for BT-1 present work], not reference for Clas
PRATPP	Phosphoribosyl-ATP pyrophosphatase	Histidine Metabolism	0.00	Reaction found for L. BT-1 [GEM for BT-1 present work], not reference for Clas
PRMICI	1-(5-phosphoribosyl)-5-[(5-phosphoribosylamino)methylideneamino)imidazole-4-carboxamide isomerase	Histidine Metabolism	0.00	Reaction found for L. BT-1 [GEM for BT-1 present work], not reference for Clas
HGNTOR	Homogentisateoxygen 1 2 oxidoreductase decyclizing	Homogentisate Pathway	0.00	Reaction added during gap filling
TYRTA	Tyrosine transaminase	Homogentisate Pathway	0.00	Reaction added during gap filling
FALDDH	FALDDH	Methane Metabolism	0.00	Reaction added during gap filling
SHSL2r	O-succinylhomoserine lyase (H2S)	Methionine Metabolism	0.00	Reaction added during gap filling
SHSL4r	O succinylhomoserine lyase elimination reversible	Methionine Metabolism	0.00	Reaction found for L. BT-1 [GEM for BT-1 present work], not reference for Clas
NNDPR	Nicotinate-nucleotide diphosphorylase (carboxylating)	Nicotinate and Nicotinamide	0.00	Reaction added during gap filling
QULNS	Quinolinate synthase	Nicotinate and Nicotinamide	0.00	Reaction added during gap filling
MTHFR3	5 10 methylenetetrahydrofolatereductase NADPH	One Carbon pool by folate	0.00	Homologous gene was not found, however expression data showed existance of this protein [Yan et al, 2013]
ACLS	Acetolactate synthase	Pantothenate and CoA Biosynthesis	0.00	Reaction added during gap filling
DHAD1	Dihydroxy-acid dehydratase (2,3-dihydroxy-3-methylbutanoate)	Pantothenate and CoA Biosynthesis	0.00	Reaction added during gap filling
MOHMT	3-methyl-2-oxobutanoate hydroxymethyltransferase	Pantothenate and CoA Biosynthesis	0.00	Reaction found for L. BT-1 [GEM for BT-1 present work], not reference for Clas
TALA	Transaldolase	Pathway	0.03	Reaction added during gap filling
HADPCOADH	3-hydroxyadipyl-CoA dehydrogenase	pathway	0.00	Reaction added during gap filling
CHORM	Chorismate mutase	Phenylalanine Tyrosine Tryptophan Biosynthesis	0.00	Reaction added during gap filling
CHORS	Chorismate synthase	Phenylalanine Tyrosine Tryptophan Biosynthesis	0.00	Reaction added during gap filling

DHQTi	3-dehydroquinate dehydratase, irreversible	Phenylalanine Tyrosine Tryptophan Biosynthesis	0.00	Reaction added during gap filling
DHQS	3-dehydroquinate synthase	Phenylalanine Tyrosine Tryptophan Biosynthesis	0.00	Reaction found for L. BT-1 [Lai etal., 2016/GEM for BT-1]
PHETA1	Phenylalanine transaminase	Phenylalanine Tyrosine Tryptophan Biosynthesis	0.00	Reaction added during gap filling
SHK3Dr	Shikimate dehydrogenase	Phenylalanine Tyrosine Tryptophan Biosynthesis	0.00	Reaction added during gap filling
SHKK	Shikimate kinase	Phenylalanine Tyrosine Tryptophan Biosynthesis	0.00	Reaction added during gap filling
TRPS2	Tryptophan synthase (indole)	Phenylalanine Tyrosine Tryptophan Biosynthesis	0.00	Reaction added during gap filling
GLUTRR	Glutamyl-tRNA reductase	Porphyrin and Chlorophyll Metabolism	0.00	Reaction added during gap filling
PYNP2r	Pyrimidine-nucleoside phosphorylase (uracil)	Pyrimidine Metabolism	0.00	Reaction added during gap filling
R15BPK	Ribose-1,5-bisphosphokinase	Ribose Metabolism	0.00	Reaction added during gap filling
R1PK	Ribose 1-phosphokinase	Ribose Metabolism	0.00	Reaction added during gap filling
CDPMEK	4-(cytidine 5-diphospho)-2-C-methyl-D-erythritol kinase	Steroid biosynthesis	0.00	Reaction added during gap filling
DXPRIi	1-deoxy-D-xylulose reductoisomerase	Steroid biosynthesis	0.00	Reaction added during gap filling
MECDPS	2-C-methyl-D-erythritol 2,4-cyclodiphosphate synthase	Steroid biosynthesis	0.00	Reaction added during gap filling
MEPCT	2-C-methyl-D-erythritol 4-phosphate cytidylyltransferase	Steroid biosynthesis	0.00	Reaction added during gap filling
APSR	Adenosine 5-phosphosulfate reductase;	Sulfur Metabolism	0.00	Reaction added during gap filling
FDMO2	FMNH2-dependent monooxygenase (methanesulfonate)	Sulfur Metabolism	0.00	Reaction added during gap filling
SADT	Sulfate adenylyltransferase	Sulfur Metabolism	0.00	Reaction added during gap filling
GLYtex	Glycine transport via diffusion (extracellular to periplasm)	Transport	-1.14	Modeling reaction added to simulate culture media
H2Otpp	H2O transport via diffusion (periplasm)	Transport	-0.48	Modeling reaction added to simulate culture media
AKGt2rpp	2-oxoglutarate reversible transport via symport (periplasm)	Transport	-0.33	Modeling reaction added to simulate culture media

AKGtex	Alpha-ketoglutarate transport via diffusion (extracellular to periplasm)	Transport	-0.33	Modeling reaction added to simulate culture media
ORNtex	Ornithine transport via diffusion (extracellular to periplasm)	Transport	-0.01	Modeling reaction added to simulate culture media
2DHGLCNtex	2DHGLCNtex	Transport	0.00	Modeling reaction with boundaries lb=0 and up= 1000
34DHCINMtex	34DHCINMtex	Transport	0.00	Modeling reaction with boundaries lb=0 and up= 1000
34DHCINMtpp	34DHCINMtpp	Transport	0.00	Modeling reaction with boundaries lb=0 and up= 1000
3OXOADPt_ex	3OXOADPt LPAREN ex RPAREN	Transport	0.00	Modeling reaction with boundaries lb=0 and up= 1000
4HBZtex	4-Hydroxybenzoate transport (extracellular)	Transport	0.00	Modeling reaction with boundaries lb=0 and up= 1000
ABUTt2pp	4-aminobutyrate transport in via proton symport (periplasm)	Transport	0.00	Modeling reaction with boundaries lb=0 and up= 1000
ABUTtex	4-aminobutyrate transport via diffusion (extracellular to periplasm)	Transport	0.00	Modeling reaction with boundaries lb=0 and up= 1000
ACONCtex	Cisaconitat transport via diffusion extracellular to periplasm	Transport	0.00	Modeling reaction with boundaries lb=0 and up= 1000
ACtex	Acetate transport via diffusion (extracellular to periplasm)	Transport	0.00	Modeling reaction with boundaries lb=0 and up= 1000
ALAtex	L-alanine transport via diffusion (extracellular to periplasm)	Transport	0.00	Modeling reaction with boundaries lb=0 and up= 1000
CATECHOLtex	CATECHOLtex	Transport	0.00	Modeling reaction with boundaries lb=0 and up= 1000
CATECHOLtpp	CATECHOLtpp	Transport	0.00	Modeling reaction with boundaries lb=0 and up= 1000
CHLtex	Choline transport via diffusion (extracellular to periplasm)	Transport	0.00	Modeling reaction with boundaries lb=0 and up= 1000
CO2tex	CO2 transport via diffusion (extracellular to periplasm)	Transport	0.00	Modeling reaction with boundaries lb=0 and up= 1000
CO2tpp	CO2 transporter via diffusion (periplasm)	Transport	0.00	Modeling reaction with boundaries lb=0 and up= 1000

CONFRLtex	CONFRLtex	Transport	0.00	Modeling reaction with boundaries lb=0 and up= 1000
CONFRLtpp	CONFRLtpp	Transport	0.00	Modeling reaction with boundaries lb=0 and up= 1000
DCAtex	Decanoate transport via diffusion (extracellular to periplasm)	Transport	0.00	Modeling reaction with boundaries lb=0 and up= 1000
DDCAtexi	Fatty acid (dodecanoate) transport via facilitated irreversible diffusion (extracellular to periplasm)	Transport	0.00	Modeling reaction with boundaries lb=0 and up= 1000
D_LACt2pp	D-lactate transport via proton symport (periplasm)	Transport	0.00	Modeling reaction with boundaries lb=0 and up= 1000
D_LACtex	D-lactate transport via diffusion (extracellular to periplasm)	Transport	0.00	Modeling reaction with boundaries lb=0 and up= 1000
ETHSO3tex	Ethanesulfonate transport via diffusion (extracellular to periplasm)	Transport	0.00	Modeling reaction with boundaries lb=0 and up= 1000
GAt1	GAt1	Transport	0.00	Modeling reaction with boundaries lb=0 and up= 1000
GAt2pp	GAt2pp	Transport	0.00	Modeling reaction with boundaries lb=0 and up= 1000
GLCNtex	D-gluconate transport via diffusion (extracellular to periplasm)	Transport	0.00	Modeling reaction with boundaries lb=0 and up= 1000
GLUtex	L-glutamate transport via diffusion (extracellular to periplasm)	Transport	0.00	Modeling reaction with boundaries lb=0 and up= 1000
GLYBtex	Glycine betaine transport via diffusion (extracellular to periplasm)	Transport	0.00	Modeling reaction with boundaries lb=0 and up= 1000
GLYCtex	Glycerol transport via diffusion (extracellular to periplasm)	Transport	0.00	Modeling reaction with boundaries lb=0 and up= 1000
H2tex	Hydrogen transport via diffusion (extracellular to periplasm)	Transport	0.00	Modeling reaction with boundaries lb=0 and up= 1000
H2tpp	Hydrogen transport diffusion (periplasm)	Transport	0.00	Modeling reaction with boundaries lb=0 and up= 1000
HDCAtexi	Hexadecanoate transport via facilitated irreversible diffusion (extracellular to periplasm)	Transport	0.00	Modeling reaction with boundaries lb=0 and up= 1000

ICITt_kt	ICITt kt	Transport	0.00	Modeling reaction with boundaries lb=0 and up= 1000
L_LACt2rpp	L-lactate reversible transport via proton symport (periplasm)	Transport	0.00	Modeling reaction with boundaries lb=0 and up= 1000
L_LACtex	L-lactate transport via diffusion (extracellular to periplasm)	Transport	0.00	Modeling reaction with boundaries lb=0 and up= 1000
MSO3tex	Methanesulfonate transport via diffusion (extracellular to periplasm)	Transport	0.00	Modeling reaction with boundaries lb=0 and up= 1000
M_XYLtpp	XYLtpp	Transport	0.00	Modeling reaction with boundaries lb=0 and up= 1000
M_Xylt1	Xylt1	Transport	0.00	Modeling reaction with boundaries lb=0 and up= 1000
NAt3_1p5pp	Sodium proton antiporter (H:NA is 1.5) (periplasm)	Transport	0.00	Modeling reaction with boundaries lb=0 and up= 1000
NAtex	Sodium transport via diffusion (extracellular to periplasm)	Transport	0.00	Modeling reaction with boundaries lb=0 and up= 1000
NI2tex	Nickel transport via diffusion (extracellular to periplasm)	Transport	0.00	Modeling reaction with boundaries lb=0 and up= 1000
NI2uabcpp	Nickel transport via ABC system (uptake, periplasm)	Transport	0.00	Modeling reaction with boundaries lb=0 and up= 1000
NO2tex	Nitrite transport via diffusion (extracellular to periplasm)	Transport	0.00	Modeling reaction with boundaries lb=0 and up= 1000
OCTAtex	Octanoate transport via diffusion (extracellular to periplasm)	Transport	0.00	Modeling reaction with boundaries lb=0 and up= 1000
PENTSOtex	PENTSOtex	Transport	0.00	Modeling reaction with boundaries lb=0 and up= 1000
PROtex	L-proline transport via diffusion (extracellular to periplasm)	Transport	0.00	Modeling reaction with boundaries lb=0 and up= 1000
PTRCtex	Putrescine transport via diffusion (extracellular to periplasm)	Transport	0.00	Modeling reaction with boundaries lb=0 and up= 1000
P_XYLtpp	P XYLtpp	Transport	0.00	Modeling reaction with boundaries lb=0 and up= 1000
P_Xylt1	P Xylt1	Transport	0.00	Modeling reaction with boundaries lb=0 and up= 1000

QUIN2tex	Quinate transport via diffusion (extracellular to periplasm)	Transport	0.00	Modeling reaction with boundaries lb=0 and up= 1000
QUINtpp	QUINtpp	Transport	0.00	Modeling reaction with boundaries lb=0 and up= 1000
RBK	Ribokinase	Transport	0.00	Modeling reaction with boundaries lb=0 and up= 1000
RIBtex	Ribose transport via diffusion (extracellular to periplasm)	Transport	0.00	Modeling reaction with boundaries lb=0 and up= 1000
SO4t4_2	Sulfate transport via sodium symport	Transport	0.00	Modeling reaction with boundaries lb=0 and up= 1000
SO4tex	Sulfate transport via diffusion (extracellular to periplasm)	Transport	0.00	Modeling reaction with boundaries lb=0 and up= 1000
SPMDtex	Spermidine transport via diffusion (extracellular to periplasm)	Transport	0.00	Modeling reaction with boundaries lb=0 and up= 1000
SUCCtex	Succinate transport via diffusion (extracellular to periplasm)	Transport	0.00	Modeling reaction with boundaries lb=0 and up= 1000
T4HCINNMtex	T4HCINNMtex	Transport	0.00	Modeling reaction with boundaries lb=0 and up= 1000
T4HCINNMtpp	T4HCINNMtpp	Transport	0.00	Modeling reaction with boundaries lb=0 and up= 1000
TAURtex	Taurine transport via diffusion (extracellular to periplasm)	Transport	0.00	Modeling reaction with boundaries lb=0 and up= 1000
TOLtex	Toluene outer membrane porin transport	Transport	0.00	Modeling reaction with boundaries lb=0 and up= 1000
TSULtex	Thiosulfate transport via diffusion (extracellular to periplasm)	Transport	0.00	Modeling reaction with boundaries lb=0 and up= 1000
TTDCAtexi	Tetradecanoate transport via facilitated irreversible diffusion (extracellular to periplasm)	Transport	0.00	Modeling reaction with boundaries lb=0 and up= 1000
FE2t	Iron II transport	Transport	0.00	Modeling reaction with boundaries lb=0 and up= 1000
NACt1pp	NACt1pp	Transport	0.00	Modeling reaction with boundaries lb=0 and up= 1000
NACtex	Nicotinic acid transport via diffusion (extracellular to periplasm)	Transport	0.00	Modeling reaction with boundaries lb=0 and up= 1000

CYStex	L-cysteine transport via diffusion (extracellular to periplasm)	Transport	0.00	Modeling reaction with boundaries lb=0 and up= 1000
HIStex	L-histidine transport via diffusion (extracellular to periplasm)	Transport	0.00	Modeling reaction with boundaries lb=0 and up= 1000
TYRt2rpp	L-tyrosine reversible transport via proton symport (periplasm)	Transport	0.00	Modeling reaction with boundaries lb=0 and up= 1000
TYRtex	L-tyrosine transport via diffusion (extracellular to periplasm)	Transport	0.00	Modeling reaction with boundaries lb=0 and up= 1000
METtex	L-methionine transport via diffusion (extracellular to periplasm)	Transport	0.00	Modeling reaction with boundaries lb=0 and up= 1000
PHEt2rpp	L-phenylalanine reversible transport via proton symport (periplasm)	Transport	0.00	Modeling reaction with boundaries lb=0 and up= 1000
PHEtex	L-phenylalanine transport via diffusion (extracellular to periplasm)	Transport	0.00	Modeling reaction with boundaries lb=0 and up= 1000
THRtex	L-threonine transport via diffusion (extracellular to periplasm)	Transport	0.00	Modeling reaction with boundaries lb=0 and up= 1000
ILEtex	L-isoleucine transport via diffusion (extracellular to periplasm)	Transport	0.00	Modeling reaction with boundaries lb=0 and up= 1000
LEUtex	L-leucine transport via diffusion (extracellular to periplasm)	Transport	0.01	Modeling reaction with boundaries lb=0 and up= 1000
MALtex	Malate transport via diffusion (extracellular to periplasm)	Transport	0.01	Modeling reaction with boundaries lb=0 and up= 1000
PIt2rpp	Phosphate reversible transport via symport (periplasm)	Transport	0.01	Modeling reaction with boundaries lb=0 and up= 1000
PItex	Phosphate transport via diffusion (extracellular to periplasm)	Transport	0.01	Modeling reaction with boundaries lb=0 and up= 1000
ARGORNt7pp	Arginine/ornithine antiporter (periplasm)	Transport	0.01	Modeling reaction with boundaries lb=0 and up= 1000
ARGtex	L-arginine transport via diffusion (extracellular to periplasm)	Transport	0.01	Modeling reaction with boundaries lb=0 and up= 1000
O2tex	Oxygen transport via diffusion (extracellular to periplasm)	Transport	0.01	Modeling reaction with boundaries lb=0 and up= 1000
O2tpp	O2 transport via diffusion (periplasm)	Transport	0.01	Modeling reaction with boundaries lb=0 and up= 1000

ASPtex	L-aspartate transport via diffusion (extracellular to periplasm)	Transport	0.21	Modeling reaction with boundaries lb=0 and up= 1000
SERt2rpp	L-serine reversible transport via proton symport (periplasm)	Transport	0.27	Modeling reaction with boundaries lb=0 and up= 1000
SERtex	L-serine transport via diffusion (extracellular to periplasm)	Transport	0.27	Modeling reaction with boundaries lb=0 and up= 1000
CITt_kt	Citrate proton symport periplasm	Transport	0.35	Modeling reaction with boundaries lb=0 and up= 1000
NH4tex	Ammonia transport via diffusion (extracellular to periplasm)	Transport	0.69	Modeling reaction with boundaries lb=0 and up= 1000
NH4tpp	Ammonia reversible transport (periplasm)	Transport	0.69	Modeling reaction with boundaries lb=0 and up= 1000
btn_etex	btn_etransport via diffusion	Transport	0.00	Modeling reaction with boundaries lb=0 and up= 1000
ins_etex	ins_etransport via diffusion	Transport	0.00	Modeling reaction with boundaries lb=0 and up= 1000
thm_etex	thm_etransport via diffusion	Transport	0.00	Modeling reaction with boundaries lb=0 and up= 1000
urea_etex	urea_etransport via diffusion	Transport	0.00	Modeling reaction with boundaries lb=0 and up= 1000
ribflv_etex	ribflv_etransport via diffusion	Transport	0.00	Modeling reaction with boundaries lb=0 and up= 1000
pnto__R_etex	pnto__R_etransport via diffusion	Transport	0.00	Modeling reaction with boundaries lb=0 and up= 1000
4abz_etex	4abz_etransport via diffusion	Transport	0.00	Modeling reaction with boundaries lb=0 and up= 1000
trp__L_etex	trp__L_etransport via diffusion	Transport	0.00	Modeling reaction with boundaries lb=0 and up= 1000
asn__L_etex	asn__L_etransport via diffusion	Transport	0.00	Modeling reaction with boundaries lb=0 and up= 1000
gln__L_etex	gln__L_etransport via diffusion	Transport	0.03	Modeling reaction with boundaries lb=0 and up= 1000
ORNDC	Ornithine Decarboxylase	Urea cycle/amino group metabolism	0.00	Reaction added during gap filling

ILETA	Isoleucine transaminase	Valine, leucine and isoleucine degradation	0.00	Reaction added during gap filling
LEUTA	Leucine transaminase	Valine, leucine and isoleucine degradation	0.00	Reaction added during gap filling
VALTA	Valine transaminase	Valine, leucine and isoleucine degradation	0.00	Reaction added during gap filling
DHAD2	Dihydroxy-acid dehydratase (2,3-dihydroxy-3-methylpentanoate)	Valine, Leucine, and Isoleucine Metabolism	0.00	Reaction added during gap filling
IPPS	2-isopropylmalate synthase	Valine, Leucine, and Isoleucine Metabolism	0.00	Reaction added during gap filling
OMCDC	2-Oxo-4-methyl-3-carboxypentanoate decarboxylation	Valine, Leucine, and Isoleucine Metabolism	0.00	Reaction added during gap filling
**Reactions in A4**				
ADCL	4-aminobenzoate synthase	Folate Biosynthesis	0	Reaction added during gap filling
GTHPi	Glutathione peridoxase	Glutathione Metabolism	-0.001534133	Reaction added during gap filling
H2Otex	H2O transport via diffusion (extracellular to periplasm)	Transport	-0.482132396	Modeling reaction with boundaries lb=0 and up= 1000
Htex	Proton transport via diffusion (extracellular to periplasm)	Transport	-0.039844523	Modeling reaction with boundaries lb=0 and up= 1000
IPDDI	Isopentenyl-diphosphate D-isomerase	Steroid biosynthesis	7.66E-06	Reaction added during gap filling, reaccion associated with pathogenic traits
PGSA120	Phosphatidylglycerol synthase (n-C12:0)	Glycerophospholipid Metabolism	7.66E-06	Reaction added during gap filling, reaccion associated with pathogenic traits
PGSA160	Phosphatidylglycerol synthase (n-C16:0)	Glycerophospholipid Metabolism	7.66E-06	Reaction added during gap filling, reaccion associated with pathogenic traits
PGSA161	Phosphatidylglycerol synthase (n-C16:1)	Glycerophospholipid Metabolism	7.66E-06	Reaction added during gap filling, reaccion associated with pathogenic traits
PGSA180	Phosphatidylglycerol synthase (n-C18:0)	Glycerophospholipid Metabolism	7.66E-06	Reaction added during gap filling, reaccion associated with pathogenic traits
PGSA181	Phosphatidylglycerol synthase (n-C18:1)	Glycerophospholipid Metabolism	7.66E-06	Reaction added during gap filling, reaccion associated with pathogenic traits
PSD160	Phosphatidylserine decarboxylase (n-C16:0)	Glycerophospholipid Metabolism	7.66E-06	Reaction added during gap filling, reaccion associated with pathogenic traits
PSD161	Phosphatidylserine decarboxylase (n-C16:1)	Glycerophospholipid Metabolism	1.53E-05	Reaction added during gap filling, reaccion associated with pathogenic traits

PSSA160	Phosphatidylserine syntase (n-C16:0)	Glycerophospholipid Metabolism	7.66E-06	Reaction found for L. BT-1 [GEM for BT-1 present work], not reference for Clas
PSSA161	Phosphatidylserine syntase (n-C16:1)	Glycerophospholipid Metabolism	1.53E-05	Reaction found for L. BT-1 [GEM for BT-1 present work], not reference for Clas
RBFSa	Riboflavin synthase	Riboflavin Metabolism	0	"Homologous gene was not found, however the enzyme was predicted [Subhraveti et al,]
SHCHD2	Sirohydrochlorin dehydrogenase (NAD)	Porphyrin and Chlorophyll Metabolism	7.66E-06	Reaction added during gap filling
TRPS3	Tryptophan synthase (indoleglycerol phosphate)	Phenylalanine Tyrosine Tryptophan Biosynthesis	0	Reaction added during gap filling
UPP3MT	Uroporphyrinogen methyltransferase	Porphyrin and Chlorophyll Metabolism	7.66E-06	Reaction added during gap filling
UPP3S	Uroporphyrinogen-III synthase	Porphyrin and Chlorophyll Metabolism	1.53E-05	Reaction found for L. asiaticus [Yan et al. 2013/GEM for L. asiaticus]
VALtex	L-valine transport via diffusion (extracellular to periplasm)	Transport	0.006154903	Homologous gene was not found, however the activity was predicted in L. BT1 [Subhraveti et al,]
VNLNpp	VNLNpp	Transport	0	Modeling reaction with boundaries lb=0 and up= 1000
**Reactions in FL17**				
ADCL	4-aminobenzoate synthase	Folate Biosynthesis	0	Reaction added during gap filling
PGSA120	Phosphatidylglycerol synthase (n-C12:0)	Glycerophospholipid Metabolism	7.66E-06	Reaction added during gap filling, reaccion associated with pathogenic traits
PGSA160	Phosphatidylglycerol synthase (n-C16:0)	Glycerophospholipid Metabolism	7.66E-06	Reaction added during gap filling, reaccion associated with pathogenic traits
PGSA161	Phosphatidylglycerol synthase (n-C16:1)	Glycerophospholipid Metabolism	7.66E-06	Reaction added during gap filling, reaccion associated with pathogenic traits
PGSA180	Phosphatidylglycerol synthase (n-C18:0)	Glycerophospholipid Metabolism	7.66E-06	Reaction added during gap filling, reaccion associated with pathogenic traits
PGSA181	Phosphatidylglycerol synthase (n-C18:1)	Glycerophospholipid Metabolism	7.66E-06	Reaction added during gap filling, reaccion associated with pathogenic traits
PSD160	Phosphatidylserine decarboxylase (n-C16:0)	Glycerophospholipid Metabolism	7.66E-06	Reaction added during gap filling, reaccion associated with pathogenic traits
PSD161	Phosphatidylserine decarboxylase (n-C16:1)	Glycerophospholipid Metabolism	1.53E-05	Reaction added during gap filling, reaccion associated with pathogenic traits

PSSA160	Phosphatidylserine syntase (n-C16:0)	Glycerophospholipid Metabolism	7.66E-06	Reaction found for L. BT-1 [GEM for BT-1 present work], not reference for Clas
PSSA161	Phosphatidylserine syntase (n-C16:1)	Glycerophospholipid Metabolism	1.53E-05	Reaction found for L. BT-1 [GEM for BT-1 present work], not reference for Clas
TRPS3	Tryptophan synthase (indoleglycerol phosphate)	Phenylalanine Tyrosine Tryptophan Biosynthesis	0	Reaction added during gap filling
SHCHD2	Sirohydrochlorin dehydrogenase (NAD)	Porphyrin Metabolism	7.66E-06	Reaction added during gap filling
UPP3MT	Uroporphyrinogen methyltransferase	Porphyrin Metabolism	7.66E-06	Reaction added during gap filling
UPP3S	Uroporphyrinogen-III synthase	Porphyrin Metabolism	1.53E-05	Reaction found for L. asiaticus [Yan et al. 2013/GEM for L. asiaticus]
RBFSa	Riboflavin synthase	Riboflavin Metabolism	0	"Homologous gene was not found, however the enzyme was predicted [Subhraveti et al,]
SULR	Sulfite reductase (NADPH2)	Sulfur Metabolism	0	Reaction added during gap filling
H2Otex	H2O transport via diffusion (extracellular to periplasm)	Transport	-0.482132396	Modeling reaction added to simulate culture media
Htex	Proton transport via diffusion (extracellular to periplasm)	Transport	-0.039844523	Modeling reaction added to simulate culture media
ISETACtex	Isethionate transport via diffusion (extracellular to periplasm)	Transport	0	Modeling reaction with boundaries lb=0 and up= 1000
NO2t2rpp	Nitrite transport in via proton symport, reversible (periplasm)	Transport	0	Modeling reaction with boundaries lb=0 and up= 1000
TOLtpp	TOLtpp	Transport	0	Modeling reaction with boundaries lb=0 and up= 1000
VALtex	L-valine transport via diffusion (extracellular to periplasm)	Transport	0.006154903	Homologous gene was not found, however the activity was predicted in L. BT1 [Subhraveti et al,]
**Reactions in gxpsy**				
P5CR	Pyrroline-5-carboxylate reductase	Arginine and Proline Metabolism	0	Reaction added during gap filling
ADCL	4-aminobenzoate synthase	Folate Biosynthesis	0	Reaction added during gap filling
PGSA120	Phosphatidylglycerol synthase (n-C12:0)	Glycerophospholipid Metabolism	7.65E-06	Reaction added during gap filling, reaccion associated with pathogenic traits
PGSA160	Phosphatidylglycerol synthase (n-C16:0)	Glycerophospholipid Metabolism	7.65E-06	Reaction added during gap filling, reaccion associated with pathogenic traits

PGSA161	Phosphatidylglycerol synthase (n-C16:1)	Glycerophospholipid Metabolism	7.65E-06	Reaction added during gap filling, reaccion associated with pathogenic traits
PGSA180	Phosphatidylglycerol synthase (n-C18:0)	Glycerophospholipid Metabolism	7.65E-06	Reaction added during gap filling, reaccion associated with pathogenic traits
PGSA181	Phosphatidylglycerol synthase (n-C18:1)	Glycerophospholipid Metabolism	7.65E-06	Reaction added during gap filling, reaccion associated with pathogenic traits
PSD160	Phosphatidylserine decarboxylase (n-C16:0)	Glycerophospholipid Metabolism	7.65E-06	Reaction added during gap filling, reaccion associated with pathogenic traits
PSD161	Phosphatidylserine decarboxylase (n-C16:1)	Glycerophospholipid Metabolism	1.53E-05	Reaction added during gap filling, reaccion associated with pathogenic traits
PSSA160	Phosphatidylserine syntase (n-C16:0)	Glycerophospholipid Metabolism	7.65E-06	Reaction found for L. BT-1 [GEM for BT-1 present work], not reference for Clas
PSSA161	Phosphatidylserine syntase (n-C16:1)	Glycerophospholipid Metabolism	1.53E-05	Reaction found for L. BT-1 [GEM for BT-1 present work], not reference for Clas
SHCHD2	Sirohydrochlorin dehydrogenase (NAD)	Porphyrin Metabolism	7.65E-06	Reaction added during gap filling
UPP3MT	Uroporphyrinogen methyltransferase	Porphyrin Metabolism	7.65E-06	Reaction added during gap filling
UPP3S	Uroporphyrinogen-III synthase	Porphyrin Metabolism	1.53E-05	Reaction found for L. asiaticus [Yan et al. 2013/GEM for L. asiaticus]
RBFSa	Riboflavin synthase	Riboflavin Metabolism	0	"Homologous gene was not found, however the enzyme was predicted [Subhraveti et al,]
IPDDI	Isopentenyl-diphosphate D-isomerase	Steroid biosynthesis	7.65E-06	Reaction added during gap filling
H2Otex	H2O transport via diffusion (extracellular to periplasm)	Transport	-0.473461935	Modeling reaction added to simulate culture media
Htex	Proton transport via diffusion (extracellular to periplasm)	Transport	-0.035536121	Modeling reaction added to simulate culture media
ISETACtex	Isethionate transport via diffusion (extracellular to periplasm)	Transport	0	Modeling reaction with boundaries lb=0 and up= 1000
TOLtpp	TOLtpp	Transport	0	Modeling reaction with boundaries lb=0 and up= 1000
UREA	Urease	Urea cycle/amino group metabolism	0.00746609	Reaction added during gap filling
**Reactions in Ishi-1**				
ORNCD	Ornithine cyclodeaminase	Arginine and Proline Metabolism	0.003209552	Reaction added during gap filling

HPPK	2-amino-4-hydroxy-6-hydroxymethyldihydropteridine diphosphokinase	Folate Biosynthesis	0.000764179	Reaction found for L. BT-1 [Lai etal., 2016/GEM for BT-1]
GTHPi	Glutathione peridoxase	Glutathione Metabolism	-0.001143212	Reaction added during gap filling
AGPAT120	1-tetradecanoyl-sn-glycerol 3-phosphate O-acyltransferase (n-C12:0)	Glycerophospholipid Metabolism	1.53E-05	Reaction added during gap filling, reaccion associated with pathogenic traits
AGPAT180	1-octadecanoyl-sn-glycerol 3-phosphate O-acyltransferase (n-C18:0)	Glycerophospholipid Metabolism	1.53E-05	Reaction added during gap filling, reaccion associated with pathogenic traits
AGPAT181	1-octadec-7-enoyl-sn-glycerol 3-phosphate O-acyltransferase (n-C18:1)	Glycerophospholipid Metabolism	2.29E-05	Reaction added during gap filling, reaccion associated with pathogenic traits
DASYN120	CDP-diacylglycerol synthetase (n-C12:0)	Glycerophospholipid Metabolism	1.53E-05	Reaction added during gap filling, reaccion associated with pathogenic traits
DASYN180	CDP-diacylglycerol synthetase (n-C18:0)	Glycerophospholipid Metabolism	1.53E-05	Reaction added during gap filling, reaccion associated with pathogenic traits
DASYN181	CDP-diacylglycerol synthetase (n-C18:1)	Glycerophospholipid Metabolism	2.29E-05	Reaction added during gap filling, reaccion associated with pathogenic traits
G3PAT120	Glycerol-3-phosphate acyltransferase (C12:0)	Glycerophospholipid Metabolism	1.53E-05	Reaction added during gap filling, reaccion associated with pathogenic traits
G3PAT160	Glycerol-3-phosphate acyltransferase (C16:0)	Glycerophospholipid Metabolism	1.53E-05	Reaction added during gap filling, reaccion associated with pathogenic traits
G3PAT161	Glycerol-3-phosphate acyltransferase (C16:1)	Glycerophospholipid Metabolism	2.29E-05	Reaction added during gap filling, reaccion associated with pathogenic traits
G3PAT180	Glycerol-3-phosphate acyltransferase (C18:0)	Glycerophospholipid Metabolism	1.53E-05	Reaction added during gap filling, reaccion associated with pathogenic traits
G3PAT181	Glycerol-3-phosphate acyltransferase (C18:1)	Glycerophospholipid Metabolism	2.29E-05	Reaction added during gap filling, reaccion associated with pathogenic traits
GLYCK	Glycerate kinase	Glycine, Serine and threonine metabolism	0.052919297	Reaction added during gap filling
GLXCL	Glyoxalate carboligase	Glycolate metabolism	0.052919297	Reaction added during gap filling
TRSARr	Tartronate semialdehyde reductase	Glycolate metabolism	0.052919297	Reaction added during gap filling
PGMT	Phosphoglucomutase	Glycolysis	-4.59E-05	Reaction found for L. asiaticus [Yan et al. 2013/GEM for L. asiaticus]
DNAMTn2r	DNA (cytosine-5-)-methyltransferase	Methionine Metabolism	-7.64E-05	Reaction added during gap filling
MHPGLUT	5 methyltetrahydropteroyltriglutamate homocysteine S methyltransferase	Methionine Metabolism	0	Reaction added during gap filling

DHPM1	Dihydropyrimidinase (5,6-dihydrouracil)	Pantothenate and CoA Biosynthesis	0	Reaction added during gap filling
DURAD	Dihydrouracil dehydrogenase (NADP)	Pantothenate and CoA Biosynthesis	0	Reaction added during gap filling
PANTS	Pantothenate synthase	Pantothenate and CoA Biosynthesis	0	Reaction added during gap filling
UPPN	B-ureidopropionase	Pantothenate and CoA Biosynthesis	0	Reaction added during gap filling
TRPS3	Tryptophan synthase (indoleglycerol phosphate)	Phenylalanine Tyrosine Tryptophan Biosynthesis	0	Reaction added during gap filling
G1SAT	Glutamate-1-semialdehyde aminotransferase	Porphyrin Metabolism	0.000122269	Reaction added during gap filling
PPPGO	Protoporphyrinogen oxidase (aerobic)	Porphyrin Metabolism	7.64E-06	Reaction added during gap filling
GART	GAR transformylase-T	Purine Metabolism	0.000764179	Reaction added during gap filling
DUTPDP	DUTP diphosphatase	Pyrimidine Metabolism	0	Reaction added during gap filling
PMDPHT	Pyrimidine phosphatase	Riboflavin Metabolism	0	Reaction added during gap filling
SULR	Sulfite reductase (NADPH2)	Sulfur Metabolism	0	Reaction added during gap filling
HMGL	Hydroxymethylglutaryl-CoA lyase	Synthesis and degradation of Ketones Bodies	0	Reaction added during gap filling
ICDHyr	Isocitrate dehydrogenase (NADP)	TCA Cycle	0.244161406	Reaction added during gap filling
ICL	Isocitrate lyase	TCA Cycle	0.105838594	Reaction added during gap filling
2DHGLCNkt_tpp	2DHGLCNkt tpp	Transport	0	Modeling reaction with boundaries lb=0 and up= 1000
CHOLS_ex	CHOLS LPAREN ex RPAREN	Transport	0	Modeling reaction with boundaries lb=0 and up= 1000
CITtex	Citrate transport via diffusion (extracellular to periplasm)	Transport	0.35	Modeling reaction with boundaries lb=0 and up= 1000
COBALT2tex	Cobalt (Co+2) transport via diffusion (extracellular to periplasm)	Transport	0	Modeling reaction with boundaries lb=0 and up= 1000
FERtex	FERtex	Transport	0	Modeling reaction with boundaries lb=0 and up= 1000
FERtpp	FERtpp	Transport	0	Modeling reaction with boundaries lb=0 and up= 1000
GLCNt2rpp	D-gluconate transport via proton symport, reversible (periplasm)	Transport	0	Modeling reaction with boundaries lb=0 and up= 1000

GLUt4pp	Na+/glutamate symport (periplasm)	Transport	0	Modeling reaction with boundaries lb=0 and up= 1000
GLYALDtex	Glyceraldehyde transport via diffusion (extracellular to periplasm)	Transport	0	Modeling reaction with boundaries lb=0 and up= 1000
HCO3_NAt	Bicarbonate transport (Na/HCO3 cotransport)	Transport	0	Modeling reaction with boundaries lb=0 and up= 1000
HXAtex	Hexanoate transport via diffusion (extracellular to periplasm)	Transport	0	Modeling reaction with boundaries lb=0 and up= 1000
ICITtex	Citrate transport via diffusion extracellular to periplasm	Transport	0	Modeling reaction with boundaries lb=0 and up= 1000
ISETACtex	Isethionate transport via diffusion (extracellular to periplasm)	Transport	0	Modeling reaction with boundaries lb=0 and up= 1000
RIBabcpp	D-ribose transport via ABC system (periplasm)	Transport	0	Modeling reaction with boundaries lb=0 and up= 1000
TOLtpp	TOLtpp	Transport	0	Modeling reaction with boundaries lb=0 and up= 1000
VALtex	L-valine transport via diffusion (extracellular to periplasm)	Transport	0.006144	Homologous gene was not found, however the activity was predicted in L. BT1 [Subhraveti et al,]
VANKpp	VANKpp	Transport	0	Modeling reaction with boundaries lb=0 and up= 1000
VANLNtex	VANLNtex	Transport	0	Modeling reaction with boundaries lb=0 and up= 1000
VANLTtex	VANLTtex	Transport	0	Modeling reaction with boundaries lb=0 and up= 1000
VNLNpp	VNLNpp	Transport	0	Modeling reaction with boundaries lb=0 and up= 1000
UREA	Urease	Urea cycle/amino group metabolism	0.007458388	Reaction added during gap filling
KARA1	Ketol-acid reductoisomerase (2,3-dihydroxy-3-methylbutanoate)	Valine, Leucine, and Isoleucine Metabolism	0	Reaction added during gap filling
KARA2	Ketol-acid reductoisomerase (2-Acetolactate)	Valine, Leucine, and Isoleucine Metabolism	0	Reaction added during gap filling
**Reactions in psy62**				

ADCL	4-aminobenzoate synthase	Folate Biosynthesis	0	Reaction added during gap filling
PGSA120	Phosphatidylglycerol synthase (n-C12:0)	Glycerophospholipid Metabolism	7.66E-06	Reaction added during gap filling, reaccion associated with pathogenic traits
PGSA160	Phosphatidylglycerol synthase (n-C16:0)	Glycerophospholipid Metabolism	7.66E-06	Reaction added during gap filling, reaccion associated with pathogenic traits
PGSA161	Phosphatidylglycerol synthase (n-C16:1)	Glycerophospholipid Metabolism	7.66E-06	Reaction added during gap filling, reaccion associated with pathogenic traits
PGSA180	Phosphatidylglycerol synthase (n-C18:0)	Glycerophospholipid Metabolism	7.66E-06	Reaction added during gap filling, reaccion associated with pathogenic traits
PGSA181	Phosphatidylglycerol synthase (n-C18:1)	Glycerophospholipid Metabolism	7.66E-06	Reaction added during gap filling, reaccion associated with pathogenic traits
PSD160	Phosphatidylserine decarboxylase (n-C16:0)	Glycerophospholipid Metabolism	7.66E-06	Reaction added during gap filling, reaccion associated with pathogenic traits
PSD161	Phosphatidylserine decarboxylase (n-C16:1)	Glycerophospholipid Metabolism	1.53E-05	Reaction added during gap filling, reaccion associated with pathogenic traits
PSSA160	Phosphatidylserine syntase (n-C16:0)	Glycerophospholipid Metabolism	7.66E-06	Reaction found for L. BT-1 [GEM for BT-1 present work], not reference for Clas
PSSA161	Phosphatidylserine syntase (n-C16:1)	Glycerophospholipid Metabolism	1.53E-05	Reaction found for L. BT-1 [GEM for BT-1 present work], not reference for Clas
TRPS3	Tryptophan synthase (indoleglycerol phosphate)	Phenylalanine Tyrosine Tryptophan Biosynthesis	0	Reaction added during gap filling
SHCHD2	Sirohydrochlorin dehydrogenase (NAD)	Porphyrin Metabolism	7.66E-06	Reaction added during gap filling
UPP3MT	Uroporphyrinogen methyltransferase	Porphyrin Metabolism	7.66E-06	Reaction added during gap filling
UPP3S	Uroporphyrinogen-III synthase	Porphyrin Metabolism	1.53E-05	Reaction found for L. asiaticus [Yan et al. 2013/GEM for L. asiaticus]
RBFSa	Riboflavin synthase	Riboflavin Metabolism	0	"Homologous gene was not found, however the enzyme was predicted [Subhraveti et al,]
SULR	Sulfite reductase (NADPH2)	Sulfur Metabolism	0	Reaction added during gap filling
FERtex	FERtex	Transport	0	Modeling reaction with boundaries lb=0 and up= 1000
FERtpp	FERtpp	Transport	0	Modeling reaction with boundaries lb=0 and up= 1000
H2Otex	H2O transport via diffusion (extracellular to periplasm)	Transport	-0.482132396	Modeling reaction added to simulate culture media

Htex	Proton transport via diffusion (extracellular to periplasm)	Transport	-0.039844523	Modeling reaction added to simulate culture media
ISETACtex	Isethionate transport via diffusion (extracellular to periplasm)	Transport	0	Modeling reaction with boundaries lb=0 and up= 1000
RIBabcpp	D-ribose transport via ABC system (periplasm)	Transport	0	Modeling reaction with boundaries lb=0 and up= 1000
TOLtpp	TOLtpp	Transport	0	Modeling reaction with boundaries lb=0 and up= 1000
VALtex	L-valine transport via diffusion (extracellular to periplasm)	Transport	0.006154903	Homologous gene was not found, however the activity was predicted in L. BT1 [Subhraveti et al,]
**Reactions in YCPsy**				
ADCL	4-aminobenzoate synthase	Folate Biosynthesis	0	Reaction added during gap filling
PGSA120	Phosphatidylglycerol synthase (n-C12:0)	Glycerophospholipid Metabolism	7.66E-06	Reaction added during gap filling, reaccion associated with pathogenic traits
PGSA160	Phosphatidylglycerol synthase (n-C16:0)	Glycerophospholipid Metabolism	7.66E-06	Reaction added during gap filling, reaccion associated with pathogenic traits
PGSA161	Phosphatidylglycerol synthase (n-C16:1)	Glycerophospholipid Metabolism	7.66E-06	Reaction added during gap filling, reaccion associated with pathogenic traits
PGSA180	Phosphatidylglycerol synthase (n-C18:0)	Glycerophospholipid Metabolism	7.66E-06	Reaction added during gap filling, reaccion associated with pathogenic traits
PGSA181	Phosphatidylglycerol synthase (n-C18:1)	Glycerophospholipid Metabolism	7.66E-06	Reaction added during gap filling, reaccion associated with pathogenic traits
PSD160	Phosphatidylserine decarboxylase (n-C16:0)	Glycerophospholipid Metabolism	7.66E-06	Reaction added during gap filling, reaccion associated with pathogenic traits
PSD161	Phosphatidylserine decarboxylase (n-C16:1)	Glycerophospholipid Metabolism	1.53E-05	Reaction added during gap filling, reaccion associated with pathogenic traits
PSSA160	Phosphatidylserine syntase (n-C16:0)	Glycerophospholipid Metabolism	7.66E-06	Reaction found for L. BT-1 [GEM for BT-1 present work], not reference for Clas
PSSA161	Phosphatidylserine syntase (n-C16:1)	Glycerophospholipid Metabolism	1.53E-05	Reaction found for L. BT-1 [GEM for BT-1 present work], not reference for Clas
TRPS3	Tryptophan synthase (indoleglycerol phosphate)	Phenylalanine Tyrosine Tryptophan Biosynthesis	0	Reaction added during gap filling
SHCHD2	Sirohydrochlorin dehydrogenase (NAD)	Porphyrin Metabolism	7.66E-06	Reaction added during gap filling

UPP3MT	Uroporphyrinogen methyltransferase	Porphyrin Metabolism	7.66E-06	Reaction added during gap filling
UPP3S	Uroporphyrinogen-III synthase	Porphyrin Metabolism	1.53E-05	Reaction found for L. asiaticus [Yan et al. 2013/GEM for L. asiaticus]
RBFSa	Riboflavin synthase	Riboflavin Metabolism	0	"Homologous gene was not found, however the enzyme was predicted [Subhraveti et al,]
SULR	Sulfite reductase (NADPH2)	Sulfur Metabolism	0	Reaction added during gap filling
H2Otex	H2O transport via diffusion (extracellular to periplasm)	Transport	-0.482132396	Modeling reaction added to simulate culture media
Htex	Proton transport via diffusion (extracellular to periplasm)	Transport	-0.039844523	Modeling reaction added to simulate culture media
ISETACtex	Isethionate transport via diffusion (extracellular to periplasm)	Transport	0	Modeling reaction with boundaries lb=0 and up= 1000
TOLtpp	TOLtpp	Transport	0	Modeling reaction with boundaries lb=0 and up= 1000
VALtex	L-valine transport via diffusion (extracellular to periplasm)	Transport	0.006154903	Homologous gene was not found, however the activity was predicted in L. BT1 [Subhraveti et al,]
**Reactions in BT-1**				
ADCL	4-aminobenzoate synthase	Folate Biosynthesis	0	Reaction added during gap filling
GLYALDtpp	Glyceraldehyde facilitated diffusion (periplasm)	Glycerophospholipid Metabolism	-0.018285759	Reaction added during gap filling, reaccion associated with pathogenic traits
PGSA120	Phosphatidylglycerol synthase (n-C12:0)	Glycerophospholipid Metabolism	7.77E-06	Reaction added during gap filling, reaccion associated with pathogenic traits
PGSA160	Phosphatidylglycerol synthase (n-C16:0)	Glycerophospholipid Metabolism	7.77E-06	Reaction added during gap filling, reaccion associated with pathogenic traits
PGSA161	Phosphatidylglycerol synthase (n-C16:1)	Glycerophospholipid Metabolism	7.77E-06	Reaction added during gap filling, reaccion associated with pathogenic traits
PGSA180	Phosphatidylglycerol synthase (n-C18:0)	Glycerophospholipid Metabolism	7.77E-06	Reaction added during gap filling, reaccion associated with pathogenic traits
PGSA181	Phosphatidylglycerol synthase (n-C18:1)	Glycerophospholipid Metabolism	7.77E-06	Reaction added during gap filling, reaccion associated with pathogenic traits
PSD160	Phosphatidylserine decarboxylase (n-C16:0)	Glycerophospholipid Metabolism	7.77E-06	Reaction added during gap filling, reaccion associated with pathogenic traits
PSD161	Phosphatidylserine decarboxylase (n-C16:1)	Glycerophospholipid Metabolism	1.55E-05	Reaction added during gap filling, reaccion associated with pathogenic traits

PSSA160	Phosphatidylserine syntase (n-C16:0)	Glycerophospholipid Metabolism	7.77E-06	Reaction found for L. BT-1 [GEM for BT-1 present work], not reference for Clas
PSSA161	Phosphatidylserine syntase (n-C16:1)	Glycerophospholipid Metabolism	1.55E-05	Reaction found for L. BT-1 [GEM for BT-1 present work], not reference for Clas
TRSARr	Tartronate semialdehyde reductase	Glycolate metabolism	2.26E-02	Reaction added during gap filling
GADktpp	GADktpp	Glycolysis	0.00E+00	Reaction added during gap filling
PGMT	Phosphoglucomutase	Glycolysis	-4.66E-05	Manually curated grRule/Reaction found for L. asiaticus [Yan et al. 2013/GEM for L. asiaticus]
HSERTA	Homoserine O trans acetylase	Methionine Metabolism	-5.07E-03	Reaction added during gap filling
TKT1	Transketolase	Pathway	-4.12E-03	Reaction added during gap filling
TKT2	Transketolase	Pentose Phosphate Pathway	-4.12E-03	Reaction found for L. asiaticus [Yan et al. 2013/GEM for L. asiaticus]
SHCHD2	Sirohydrochlorin dehydrogenase (NAD)	Porphyrin and Chlorophyll Metabolism	7.77E-06	Reaction added during gap filling
UPP3MT	Uroporphyrinogen methyltransferase	Porphyrin and Chlorophyll Metabolism	7.77E-06	Reaction added during gap filling
UPP3S	Uroporphyrinogen-III synthase	Porphyrin and Chlorophyll Metabolism	1.55E-05	Reaction found for L. asiaticus [Yan et al. 2013/GEM for L. asiaticus]
IPDPS	1-hydroxy-2-methyl-2-(E)-butenyl 4-diphosphate reductase (ipdp)	Steroid biosynthesis	2.33E-05	Reaction added during gap filling, reaccion associated with pathogenic traits
COBALT2tpp	Cobalt transport in via permease (no H+)	Transport	0	Modeling reaction with boundaries lb=0 and up= 1000
H2Otex	H2O transport via diffusion (extracellular to periplasm)	Transport	-0.415341788	Modeling reaction added to simulate culture media
Htex	Proton transport via diffusion (extracellular to periplasm)	Transport	0.032495886	Modeling reaction with boundaries lb=0 and up= 1000
ISETACtex	Isethionate transport via diffusion (extracellular to periplasm)	Transport	0.00E+00	Modeling reaction with boundaries lb=0 and up= 1000
TOLtpp	TOLtpp	Transport	0.00E+00	Modeling reaction with boundaries lb=0 and up= 1000
VALtex	L-valine transport via diffusion (extracellular to periplasm)	Transport	0.00624689	Homologous gene was not found, however the activity was predicted in L. BT1 [Subhraveti et al,]

Breakdown of reactions by general subsystem

Transport	Modeling reactions
Metabolic	Reference/gap filling (30:70)

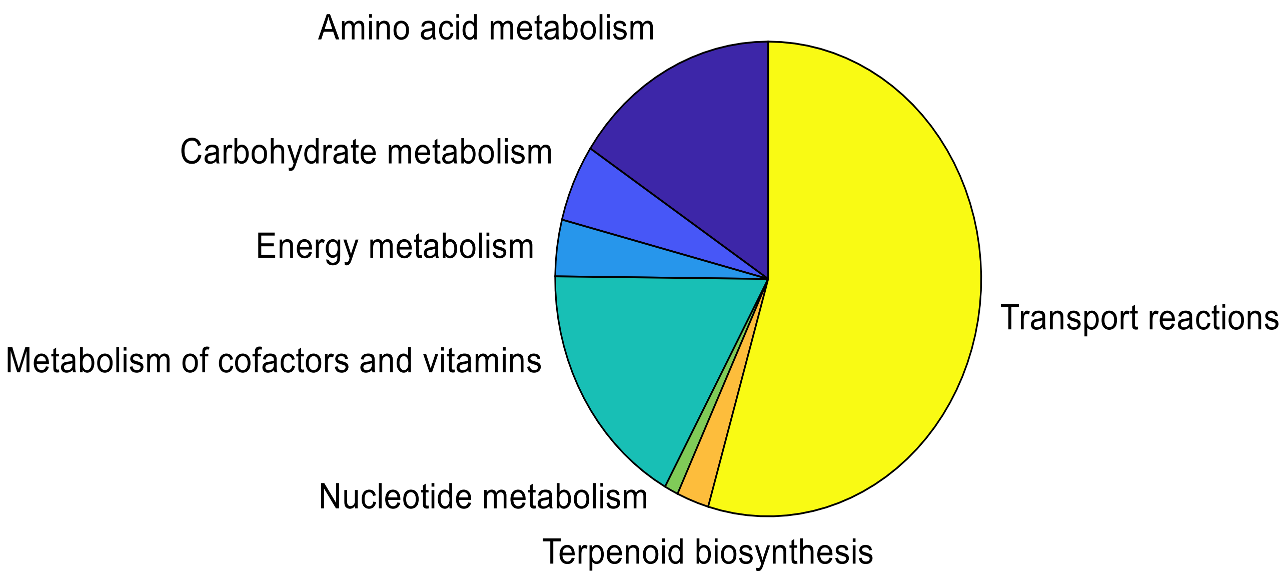

Supplement: Supplementary file 1 — Supplementary Information [file 41540_2020_142_MOESM1_ESM.pdf]
