# Supplementary material for: Linking metabolic phenotypes to pathogenic traits among “Candidatus Liberibacter asiaticus” and its hosts
Source: NPJ Syst Biol Appl. 2020 Aug 4;6:24. doi: 10.1038/s41540-020-00142-w (PMC7403731; doi:10.1038/s41540-020-00142-w)
Supplement: Supplementary file 2 — Dataset 1 [file 41540_2020_142_MOESM2_ESM.zip › Liberibacter-master/SupplementalText_Figures.docx]

**Supplementary Information**

Linking metabolic phenotypes to pathogenic traits among ‘*Candidatus* Liberibacter asiaticus’ and its hosts

Cristal Zuniga^1^, Beth Peacock^2*^, Bo Liang^1,3*^, Greg McCollum^4^, Sonia C. Irigoyen^5^, Diego Tec^1,6^, Clarisse Marotz^1^, Nien-Chen Weng^1^, Alejandro Zepeda^6^, Georgios Vidalakis^2^, Kranthi K. Mandadi^5,7^, James Borneman^2,#^, Karsten Zengler^1,8,9,#^

^1^Department of Pediatrics, University of California, San Diego, 9500 Gilman Drive, La Jolla, CA 92093-0760, USA

^2^Department of Microbiology and Plant Pathology, University of California, 900 University Ave, Riverside, CA 92521

^3^State Key Laboratory of Bioreactor Engineering and Institute of Applied Chemistry, East China University of Science and Technology, Shanghai, P.R. China

^4^USDA, ARS, US Horticultural Research Laboratory, 2001 S. Rock Road, Ft. Pierce, FL 34945

^5^Texas A&M AgriLife Research and Extension Center, Texas A&M University System, Weslaco, Texas, USA

^6^Facultad de Ingeniería Química, Universidad Autónoma de Yucatán, Campus de Ciencias Exactas e Ingenierías, Mérida 97203, Yucatán, México

^7^Department of Plant Pathology and Microbiology, Texas A&M University, College Station, TX, USA

^8^Department of Bioengineering, University of California, San Diego, La Jolla CA 92093-0412, USA

^9^Center for Microbiome Innovation, University of California, San Diego, 9500 Gilman Drive, La Jolla, CA 92093-0403, USA

*Equal contribution

# Correspondence to James Borneman (borneman@ucr.edu) or Karsten Zengler (kzengler@ucsd.edu).

**This PDF file includes:**

Supplementary Figures 1 to 7

Captions for Supplementary Tables 1 to 9

Captions for Supplementary Dataset 1

References

Table of contents

[Supplemental Figures 3](#_Toc26798957)

[Supplementary Fig. 1. Phylogenetic *C*Las properties. 3](#_Toc26798958)

[Supplementary Fig. 2. Genome-scale models comparison and auxotrophies prediction. 4](#_Toc26798959)

[Supplementary Fig. 3. Model simulations by culture medium and metabolic model. 5](#_Toc26798960)

[Supplementary Fig. 4. RNA-sequencing data profiles. 6](#_Toc26798961)

[Supplementary Fig. 5. Quality control and analysis of RNA-sequencing data. 7](#_Toc26798962)

[Supplementary Fig. 6. Correlation matrix of predicted flux distributions. 8](#_Toc26798963)

[Supplementary Fig. 7. Gene essentiality analysis by strain. 9](#_Toc26798964)

[Captions of Supplementary Tables 10](#_Toc26798965)

[Supplementary Table 1. Manually curated gene-protein-reactions associations 10](#_Toc26798966)

[Supplementary Table 2. Reactions present in *Liberibacter crescens* BT-1 and absent in *C*Las strains 10](#_Toc26798967)

[Supplementary Table 3. Culture media compositions and imposed constraints 10](#_Toc26798968)

[Supplementary Table 4. Connectivity analysis by metabolite in the culture medium 10](#_Toc26798969)

[Supplementary Table 5. Preprocessing results and analysis of RNA-sequencing data (metadata) 10](#_Toc26798970)

[Supplementary Table 6. Analysis of RNA-sequencing data 10](#_Toc26798971)

[Supplementary Table 7. Gene essentiality by strain and subsystem 10](#_Toc26798972)

[Supplementary Table 8. Comparison among predicted gene essentiality in BT-1 and experimentally determined essential genes by Lai et al., 2016 10](#_Toc26798973)

[Supplementary Table 9. Predicted strain-specific *C*Las genes potentially lethal useful for biocontrol 10](#_Toc26798974)

[Captions of Supplementary Dataset 1 11](#_Toc26798975)

[Available models ID 11](#_Toc26798976)

# Supplemental Figures


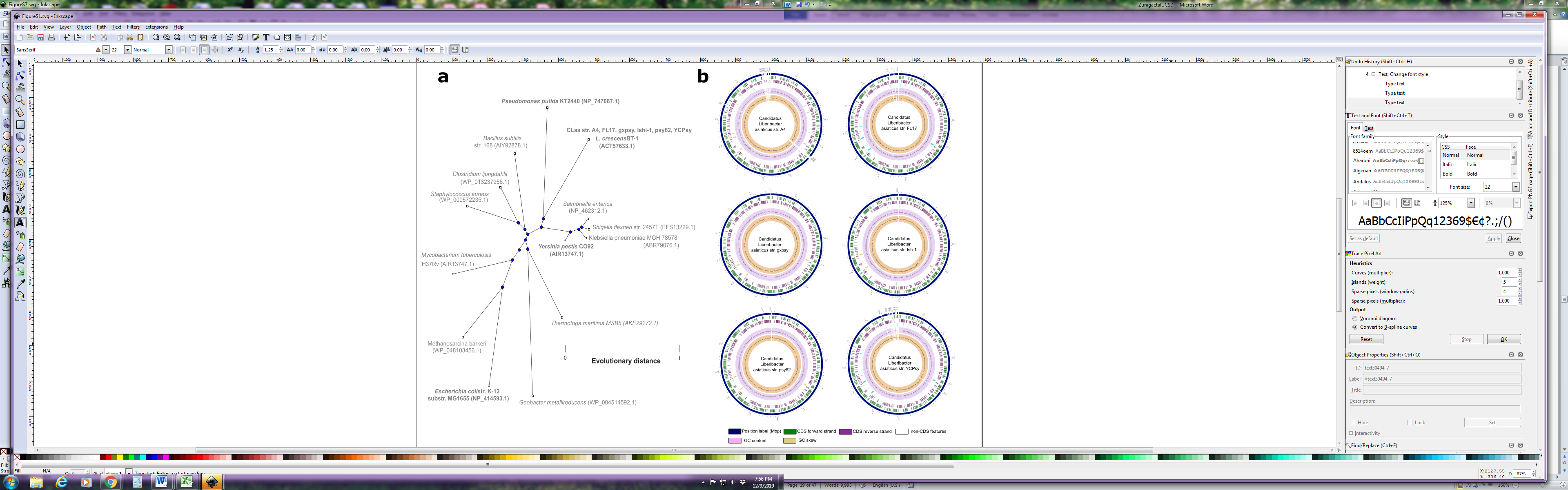


Supplementary Fig. 1. Phylogenetic *C*Las properties. **a**, Neighbor-joining tree based on almost full-length 16S rRNA gene sequences, showing phylogenetic relationships among *Liberibacter* strains and bacteria with available reconstructions. GenBank accession numbers are given in parentheses. **b**, Genomes feature of *Candidatus* Liberibacter asiaticus strains.


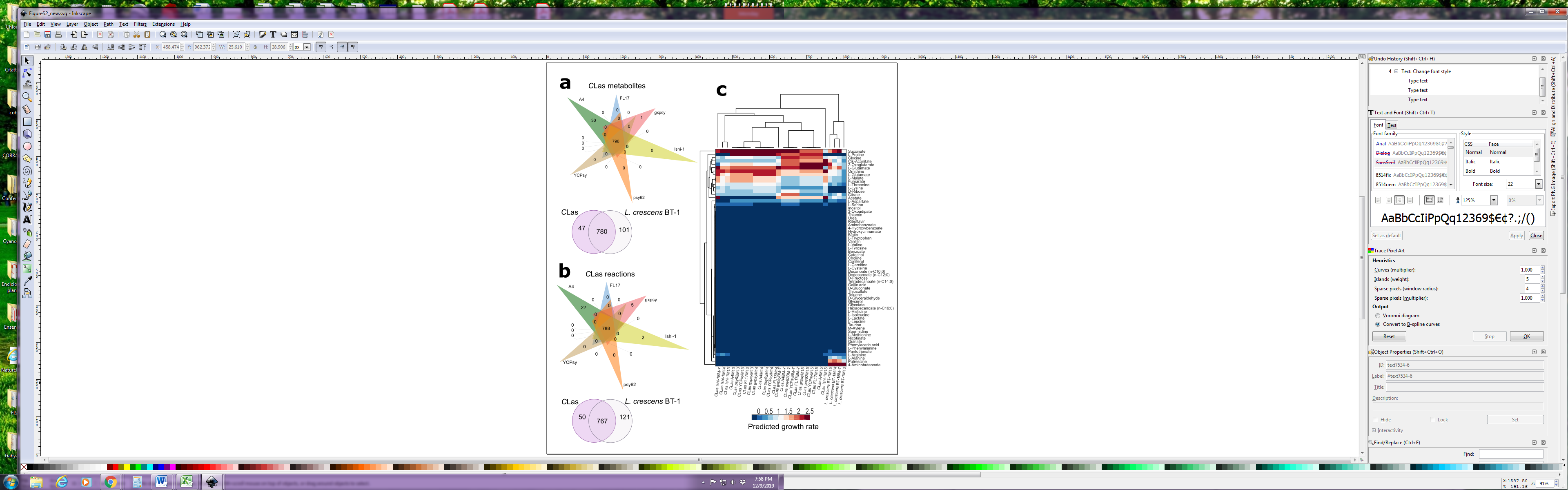


Supplementary Fig. 2. Genome-scale models comparison and auxotrophies prediction. **a-b**, Strain-specific metabolites and reactions across in models of *Candidatus* Liberibacter asiaticus (*C*Las) and *Liberibacter crescens* BT-1. **c,** Auxotrophies predictions for all *C*Las strains and BT-1 under different culture media: BM7, M13, M14, and M15.


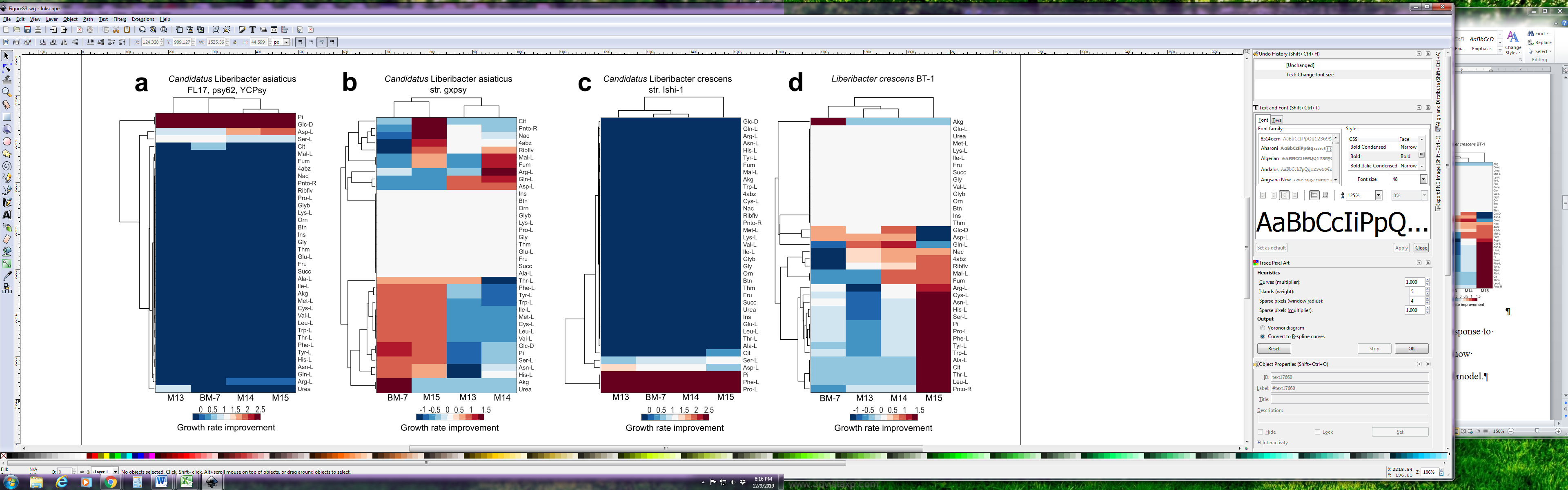


Supplementary Fig. 3. Model simulations by culture medium and metabolic model. The response to individual additions of carbon substrates were evaluated for the seven models. Results show different outcomes depending on the overall culture medium composition and evaluated model.


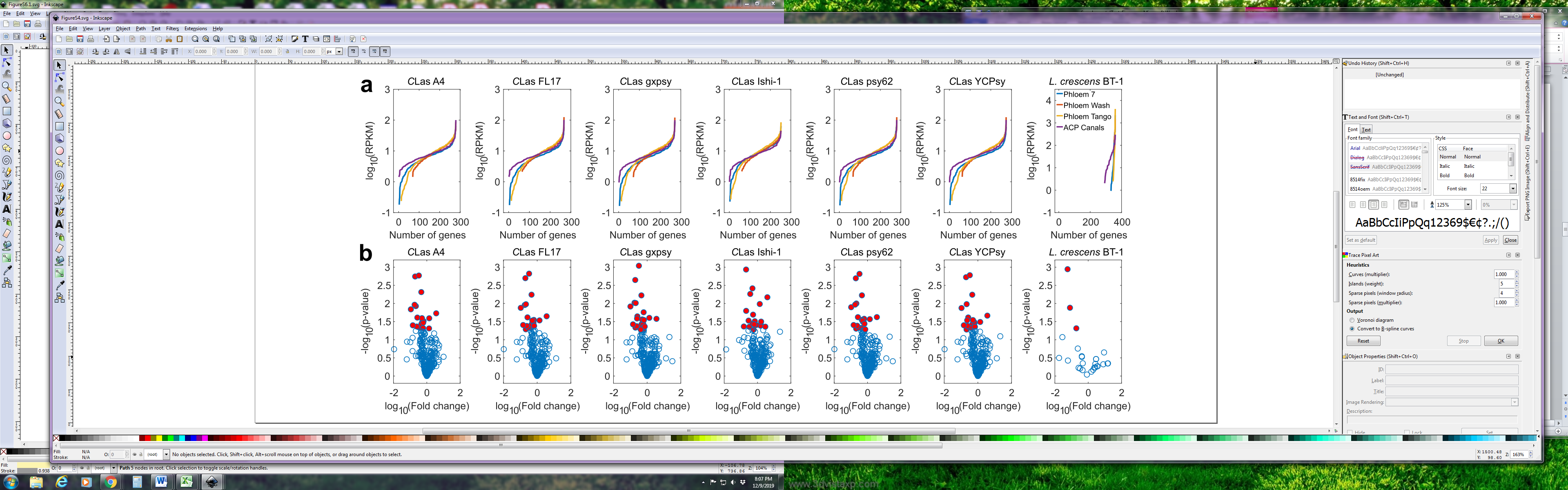


Supplementary Fig. 4. RNA-sequencing data profiles. **a**, Normalized data by *Liberibacter* strain and sample. Samples were taken from citrus phloem from the cultivars Valencia orange, Washington navel orange, and Tango mandarin as well as from Asian Citrus Psyllid (ACP) alimentary canals. **b**, Differential expression analysis by *Liberibacter* strain. Dots highlighted in red have a p-value<0.05 (t-test).


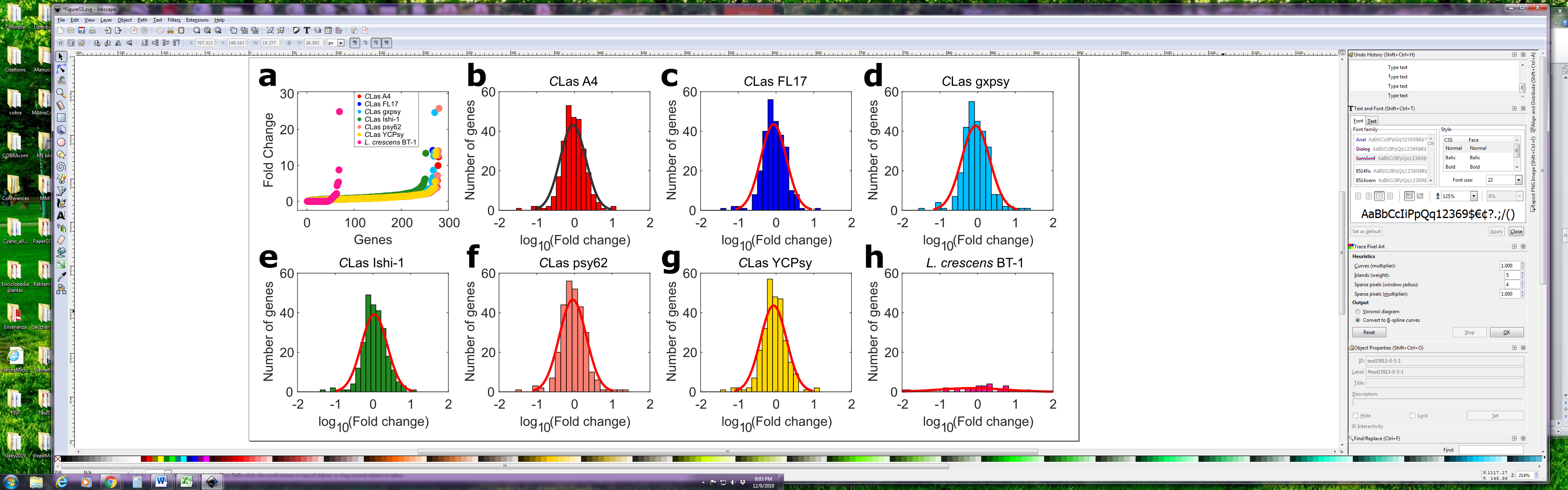


Supplementary Fig. 5. Quality control and analysis of RNA-sequencing data. **a**, Fold change (phloem versus psyllid) by gene in the *Liberibacter* models. **b-g**, Fold change is normally distributed. The parameters estimation to fit the distributions was performed using the Probability Distributions Toolbox for MATLAB. The mean of the *C*Las distributions was around -0.0597±0.024 and the sigma parameter describing the square root of the unbiased estimate of the variance was 0.3378± 0.0150. This value was used as a threshold to define up and downregulated genes in addition to p-values obtained from the t-test.


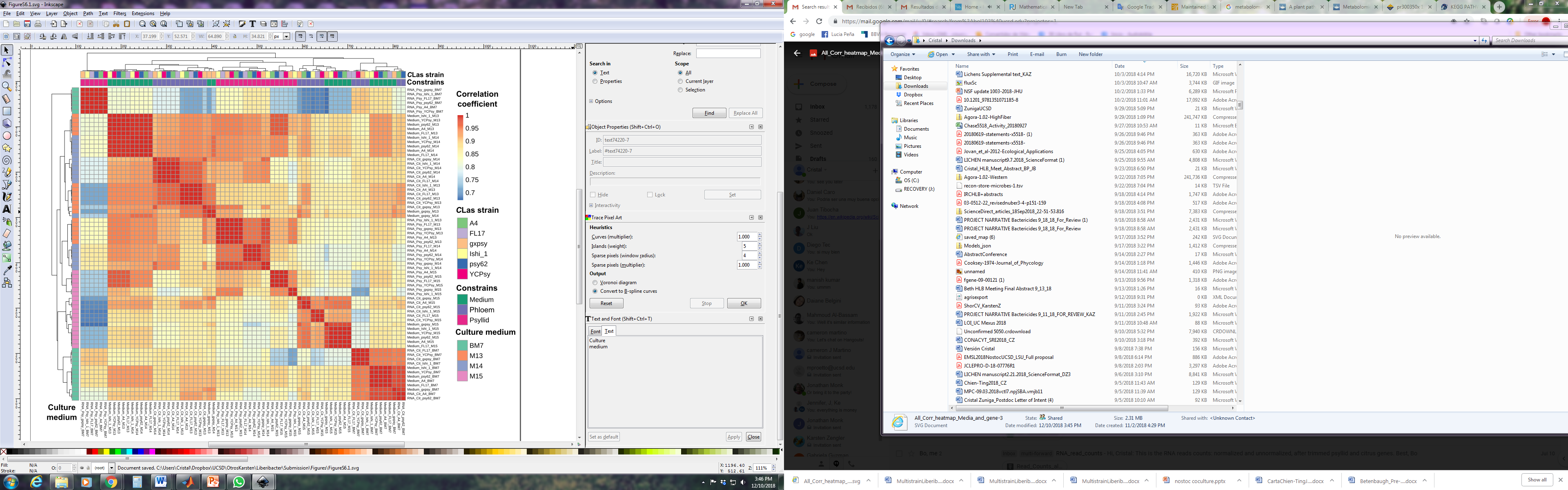


Supplementary Fig. 6. Correlation matrix of predicted flux distributions. Flux distributions for all *Candidatus* Liberibacter asisticus (*C*Las) strains were simulated under four culture media conditions (BM7, M13, M14, and M15) and different RNA-sequencing constraints. Predicted flux distributions cluster by culture medium and host, containing all *C*Las strains by cluster.


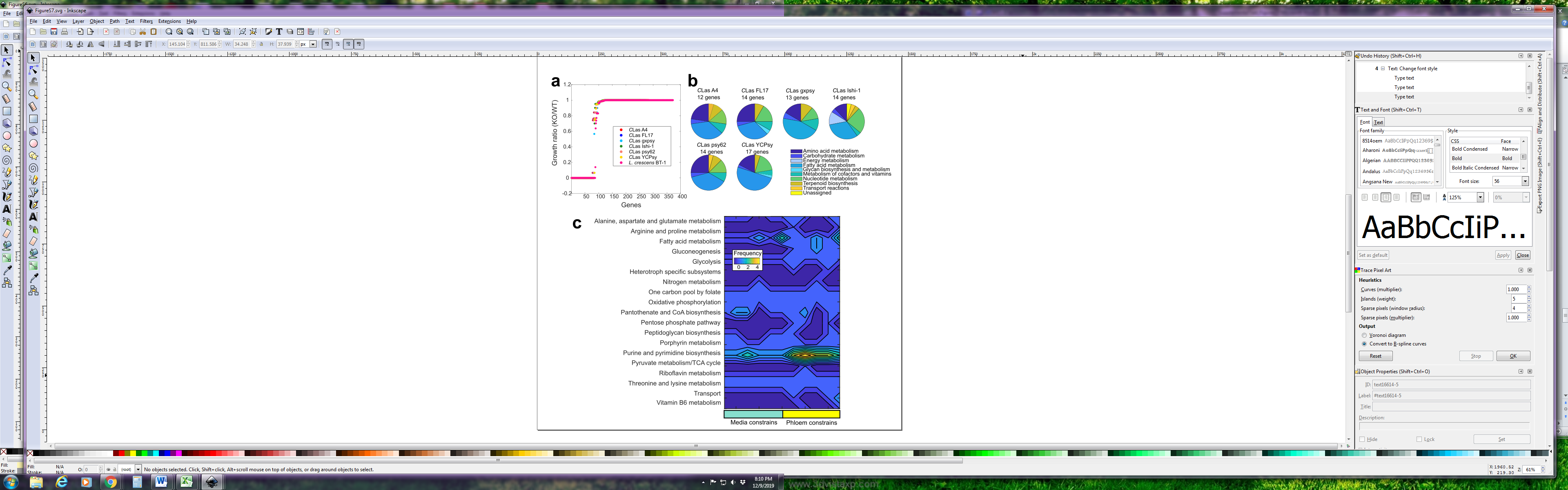


Supplementary Fig. 7. Gene essentiality analysis by strain. **a**, Growth rate ratio *in silico* knock-out of each gene in the *Liberibacter* models. **b**, General subsystems breakdown of genes reducing the growth rate to less than 0.3 and with a significant differential expression between the plant and psyllid (t-test, p-value<0.05). **c**, Essential genes highly expressed (t-test, p-value<0.05, fold change>10) during *C*Las infection of trees.

# Captions of Supplementary Tables

## Supplementary Table 1. Manually curated gene-protein-reactions associations

## Supplementary Table 2. Reactions present in *Liberibacter crescens* BT-1 and absent in *C*Las strains

## Supplementary Table 3. Culture media compositions and imposed constraints

## Supplementary Table 4. Connectivity analysis by metabolite in the culture medium

## Supplementary Table 5. Preprocessing results and analysis of RNA-sequencing data (metadata)

## Supplementary Table 6. Analysis of RNA-sequencing data

## Supplementary Table 7. Gene essentiality by strain and subsystem

## Supplementary Table 8. Comparison among predicted gene essentiality in BT-1 and experimentally determined essential genes by Lai et al., 2016

## Supplementary Table 9. Predicted strain-specific *C*Las genes potentially lethal useful for biocontrol

# Captions of Supplementary Dataset 1

Properly constrained metabolic models (7 strains total) using four culture medium (BG-7, M13, M15) are provided in Dataset 1. This Dataset can be also found at <https://github.com/cristalzucsd/Liberibacter>.

## Available models ID

| **Model ID** | **Microorganism name** | **Culture medium** | **Available formats** |
| --- | --- | --- | --- |
| CLasA4_BM7 | Candidatus Liberibacter asiaticus strain A4 | B7 | .mat/.json/.xml |
| CLasA4_M13 | Candidatus Liberibacter asiaticus strain A4 | M13 | .mat/.json/.xml |
| CLasA4_M14 | Candidatus Liberibacter asiaticus strain A4 | M14 | .mat/.json/.xml |
| CLasA4_M15 | Candidatus Liberibacter asiaticus strain A4 | M15 | .mat/.json/.xml |
| CLasFL17_BM7 | Candidatus Liberibacter asiaticus strain FL17 | B7 | .mat/.json/.xml |
| CLasFL17_M13 | Candidatus Liberibacter asiaticus strain FL17 | M13 | .mat/.json/.xml |
| CLasFL17_M14 | Candidatus Liberibacter asiaticus strain FL17 | M14 | .mat/.json/.xml |
| CLasFL17_M15 | Candidatus Liberibacter asiaticus strain FL17 | M15 | .mat/.json/.xml |
| CLasIshi_BM7 | Candidatus Liberibacter asiaticus str. gxpsy | B7 | .mat/.json/.xml |
| CLasIshi_M13 | Candidatus Liberibacter asiaticus str. gxpsy | M13 | .mat/.json/.xml |
| CLasIshi_M14 | Candidatus Liberibacter asiaticus str. gxpsy | M14 | .mat/.json/.xml |
| CLasIshi_M15 | Candidatus Liberibacter asiaticus str. gxpsy | M15 | .mat/.json/.xml |
| CLasYCPsy_BM7 | Candidatus Liberibacter asiaticus str. Ishi | B7 | .mat/.json/.xml |
| CLasYCPsy_M13 | Candidatus Liberibacter asiaticus str. Ishi | M13 | .mat/.json/.xml |
| CLasYCPsy_M14 | Candidatus Liberibacter asiaticus str. Ishi | M14 | .mat/.json/.xml |
| CLasYCPsy_M15 | Candidatus Liberibacter asiaticus str. Ishi | M15 | .mat/.json/.xml |
| CLasgxpsy_BM7 | Candidatus Liberibacter asiaticus strain psy62 | B7 | .mat/.json/.xml |
| CLasgxpsy_M13 | Candidatus Liberibacter asiaticus strain psy62 | M13 | .mat/.json/.xml |
| CLasgxpsy_M14 | Candidatus Liberibacter asiaticus strain psy62 | M14 | .mat/.json/.xml |
| CLasgxpsy_M15 | Candidatus Liberibacter asiaticus strain psy62 | M15 | .mat/.json/.xml |
| CLaspsy62_BM7 | Candidatus Liberibacter asiaticus strain YCPsy | B7 | .mat/.json/.xml |
| CLaspsy62_M13 | Candidatus Liberibacter asiaticus strain YCPsy | M13 | .mat/.json/.xml |
| CLaspsy62_M14 | Candidatus Liberibacter asiaticus strain YCPsy | M14 | .mat/.json/.xml |
| CLaspsy62_M15 | Candidatus Liberibacter asiaticus strain YCPsy | M15 | .mat/.json/.xml |
| LcBT1_BM7 | Liberibacter crescens BT-1 | B7 | .mat/.json/.xml |
| LcBT1_M13 | Liberibacter crescens BT-1 | M13 | .mat/.json/.xml |
| LcBT1_M14 | Liberibacter crescens BT-1 | M14 | .mat/.json/.xml |
| LcBT1_M15 | Liberibacter crescens BT-1 | M15 | .mat/.json/.xml |
